# Conservation of HLA Spike Protein Epitopes Supports T Cell Cross-Protection in SARS-CoV-2 Vaccinated Individuals against the Potentially Zoonotic Coronavirus Khosta-2

**DOI:** 10.3390/ijms25116087

**Published:** 2024-05-31

**Authors:** Antonio J. Martín-Galiano, Daniel López

**Affiliations:** 1 Core Scientific and Technical Units, Instituto de Salud Carlos III, Majadahonda, 28220 Madrid, Spain; mgaliano@isciii.es; 2 Centro Nacional de Microbiología, Instituto de Salud Carlos III, Majadahonda, 28220 Madrid, Spain

**Keywords:** coronaviruses, cross-protection, escape mutant, HLA, T cells, SARS-CoV-2, vaccines

## Abstract

Heterologous vaccines, which induce immunity against several related pathogens, can be a very useful and rapid way to deal with new pandemics. In this study, the potential impact of licensed COVID-19 vaccines on cytotoxic and helper cell immune responses against Khosta-2, a novel sarbecovirus that productively infects human cells, was analyzed for the 567 and 41 most common HLA class I and II alleles, respectively. Computational predictions indicated that most of these 608 alleles, covering more than 90% of the human population, contain sufficient fully conserved T-cell epitopes between the Khosta-2 and SARS-CoV-2 spike-in proteins. Ninety percent of these fully conserved peptides for class I and 93% for class II HLA molecules were verified as epitopes recognized by CD8^+^ or CD4^+^ T lymphocytes, respectively. These results show a very high correlation between bioinformatic prediction and experimental assays, which strongly validates this study. This immunoinformatics analysis allowed a broader assessment of the alleles that recognize these peptides, a global approach at the population level that is not possible with experimental assays. In summary, these findings suggest that both cytotoxic and helper cell immune protection elicited by currently licensed COVID-19 vaccines should be effective against Khosta-2 virus infection. Finally, by being rapidly adaptable to future coronavirus pandemics, this study has potential public health implications.

## 1. Introduction

Genome-level immunologic surveillance of coronavirus variants with pandemic potential is relevant in virulence and cross-immunoprotective terms. SARS-CoV-2, the etiologic agent of COVID-19, is responsible for a devastating pandemic that has resulted in more than 774 million confirmed cases and 7 million deaths worldwide to date (https://www.who.int/emergencies/diseases/novel-coronavirus-2019/situation-reports, accessed on 1 April 2024). SARS-CoV-2 is the third betacoronavirus, and the second within the sarbecovirus subgenus, to cause zoonoses in the last two decades. Thus, vaccine prophylaxis against COVID-19 and future pandemics, with special attention to sarbecoviruses, is a key issue in today’s globalized world.

Effective activation of the three branches of adaptive immunity: (i) cytotoxic CD8^+^ T lymphocytes, (ii) helper CD4^+^ T cells and (iii) neutralizing antibodies are key to surviving natural SARS-CoV-2 infection or generating a protective immune response after vaccination [1]. The specific interaction of CD4^+^ or CD8^+^ T-cell receptors with short viral peptides bound to human leukocyte antigen (HLA) class I or II molecules, respectively, triggers diverse functions of these T lymphocytes. Such activities include activation, regulation, or suppression of multiple components of adaptive immune responses [2]. In the absence of adequate recognition by HLA-restricted class I and II T lymphocytes, both humoral and cellular immune responses are not properly activated, resulting in viral spread throughout the body with potentially fatal outcomes for the host. The extremely complex set of immune events, triggered by HLA-restricted viral peptide recognition, can be altered or even suppressed by single changes in viral epitope sequences leading to a complete loss of antigen recognition by CD4^+^ or CD8^+^ T cells. These immune evasion events have been previously described for influenza [3], HCV [4], HIV [5], LCMV [6], SIV [5], coronavirus [7], and SARS-CoV-2 itself [8]. This very low tolerance to amino acid changes in antigen recognition may render lymphocytes previously activated by vaccine administration ineffective against new viral variants that harbor multiple non-synonymous mutations [9]. Therefore, in general, it is unlikely that a vaccine developed against a given virus can generate a cross-protective response against another related virus.

In contrast, cross-protective immunity has been observed between non-pathogenic poxviruses in humans, such as cowpox, horsepox or vaccinia, and pandemic smallpox [10]. This allowed the eradication of this terrible pandemic after worldwide immunization with the vaccinia virus [10]. In a recent study, and given the high similarity between the spike proteins of SARS-CoV-1 and SARS-CoV-2 sarbecoviruses, we proposed that the cytotoxic and helper cellular immune responses elicited by currently licensed COVID-19 vaccines should generate sufficient cross-protection against SARS-CoV-1 infection [11]. Furthermore, vaccination against SARS-CoV-1 in mice induced cross-reactive B and T lymphocytes against SARS-CoV-2, protecting against heterologous coronavirus challenges [12], strengthening our reasoning.

In this context, Khosta-2, a new sarbecovirus identified in horseshoe bats from the southern regions of Russia [13] has raised global concern about the possibility of this virus becoming a new pandemic. The Khosta-2 spike protein can effectively interact with the human ACE2 receptor in a trypsin-independent manner and productively infect human cells [14], suggesting potential anthropozoonotic behavior. However, laboratory experiments with the monoclonal bamlanivimab antibody and vaccinated patient antisera demonstrated that Khosta-2 is able to escape the humoral response elicited from individuals vaccinated with SARS-CoV-2 vaccines [15]. Based on the above, may SARS-CoV-2 vaccines provide any protection against Khosta-2 infection through the two T cellular branches of the adaptive response? Importantly, these cellular responses can remain operative even when the humoral response elicited by vaccines declines with time [16].

In this study, we have computationally addressed this aspect by focusing on the HLA class I and II-restricted epitopes shared between SARS-CoV-2 and Khosta-2 spike proteins. Although a significant loss of HLA-restricted epitopes derived from COVID-19 vaccines was detected, a still relevant number of conserved epitopes involving the bulk of the human population remained intact between the spike proteins of both sarbecoviruses. It is, therefore, expected that this cross-epitope pool in currently licensed vaccines could globally generate sufficient cytotoxic and helper cross-reactive immune responses against Khosta-2.

## 2. Results and Discussion

The striking polymorphism of HLA class I and II molecules, with more than 26,000 and 10,000 alleles identified to date [17], respectively, makes experimental study of cellular immune responses at the human population level extremely difficult. However, based on the structure and functionality of HLA class I and II molecules many of these antigen-presenting proteins are classified into three levels: families, superfamilies, and finally into twelve and ten canonical HLA class I and II supertypes. Thus, the HLA alleles included in each supertype share strong functional similarities at the peptide-ligand specificity level. Furthermore, the 551 HLA-A and -B class I alleles and another 41 HLA class II alleles more abundantly spread in humans included in these twenty-two HLA class I and II supertypes comprise more than 90% [18] and more than 95% [19,20] of the world’s population, regardless of ethnicity. Thus, the selection of abundant alleles of these supertypes significantly reduces data complexity, facilitates computational analysis, and allows the study of herd immunity at a global level.

**Figure 1:**
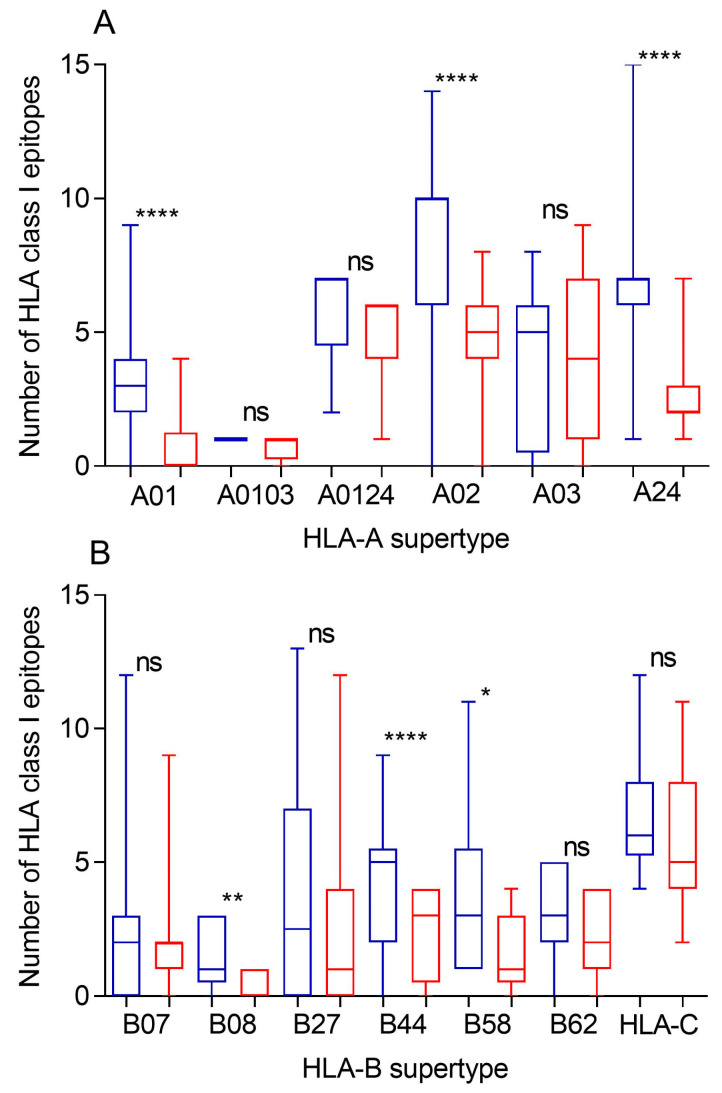
Conservation of Khosta-2 or SARS-CoV-1 epitopes with the SARS-CoV-2 spike protein sequence predicted for the most frequent HLA class I alleles. The number of fully conserved epitopes in Khosta-2 (red), and SARS-CoV-1 (blue) predicted for HLA class I molecules including in the 6 HLA-A supertypes (panel (**A**)) and the 6 HLA-B supertypes and the 16 most frequent HLA-C alleles (panel (**B**)). The median value is indicated. Box limits indicate the interquartile range. Whiskers are adjusted to maximal and minimal values. Significant *p* values: *, *p* < 0.05; **, *p* < 0.01; ****, *p* < 0.001; ns, non significant.

Based on the accuracy of current immunoinformatic tools, a predictive analysis of the impact of changes described in the Khosta-2 spike protein versus the theoretical epitopes from the SARS-CoV-2 spike protein was conducted. Similar studies involving SARS-CoV-1 and SARS-CoV-2 variants of concern were previously carried out [11,21,22]. The spike surface protein is the only viral product included in internationally licensed vaccines against the COVID-19 disease [23]. Thus, the influence of Khosta-2 spike protein mutations for each of the 551 HLA class I alleles associated with the twelve canonical HLA-A and -B class I supertypes was scrutinized (Figure 1). In a previous study, theoretical SARS-CoV-2 spike proteins with 304 random changes, the number of changes among spike proteins from SARS-CoV-1 and SARS-CoV-2, showed almost complete destruction of all class I HLA-restricted epitopes generated on the SARS-CoV-2 spike protein [11]. However, expectedly, as the 351 changes and 32 gaps between Khosta-2 and SARS-CoV-2 spike protein sequences are not randomly distributed, a significant but not total loss of HLA-A-restricted epitopes derived from SARS-CoV-2 vaccines was detected in Khosta-2 spike protein (Figure 1A, Table 1 and Table S1). Strikingly, up to 107 (19.4% of total) HLA-A class I alleles from all supertypes, except A0103, had more than three predicted epitopes fully conserved among the spike proteins from both sarbecoviruses (Table 2). Importantly, HLA-A*02:01 (the most prevalent HLA allele in humans) and eight different HLA-A alleles from the A02 supertype (A*02:02, A*02:03, A*02:11, A*02:12, A*02:16, A*02:22, A*02:47, and A*02:63) retained seven or more conserved epitopes on the Khosta-2 spike protein (Table S1). Other examples are, HLA-A*26:02 and -A*26:12 alleles from the A01 supertype, which could bind four conserved epitopes and seven HLA-A alleles (A*29:01, A*29:02, A*29:06, A*29:09, A*29:10, A*29:11, and A*29:12) from A0124 supertype that retained six unchanged epitopes between both spike proteins. In addition, another 22 HLA alleles from the A03 supertype retained seven or more conserved epitopes among sarbecovirus spike proteins (Table S1). Finally, HLA-A*23:04 and -A*24:03 with seven and six conserved epitopes, respectively, were the alleles from the A24 supertype with higher epitope conservation between Khosta-2 and SARS-CoV-2 spike proteins (Table S1).

For comparison, Figure 1 also includes the average number of epitopes conserved between SARS-CoV-1 and SARS-CoV-2 spike protein sequences (blue bins) predicted for HLA class I alleles including in the six HLA-A class I supertypes [11]. For most HLA-A alleles, the 304 changes between SARS-CoV-1 and SARS-CoV-2 spike-in proteins actually destroyed fewer HLA-A-restricted SARS2-CoV-2 epitopes than the 351 changes associated with Khosta-2 (Figure 1A). This suggests a lower cross-protective effect for the latter. The supertypes A0103, A0104, and A03 were the least affected. Unexpectedly, 32 HLA-A alleles, all of them of supertype A03 (HLA-A*03:01, -A*03:02, -A*03:04, -A*03:05, -A*03:06, -A*03:07, -A*03:08, -A*03:10, -A*03:12, -A*03:13, -A*03:14, -A*03:16, -A*03:17, -A*11:01, -A*11:02, -A*11:05, -A*11:07, -A*11:08, -A*11:09, -A*11:12, -A*11:13, -A*11:14, -A*11:15, -A*11:16, -A*11:20, -A*11:23, -A*31:06, -A*31:09, -A*31:11, -A* 33:06, -A*74:05, and -A*74:07) retained more conserved epitopes in the spike protein of Khosta-2 than in SARS-CoV-1. Thus, although the median depicted in Figure 1A for the A03 supertype is higher for conserved epitopes in SARS-CoV-1 (5.0) versus Khosta-2 (4.0), the mean of those data is higher for Khosta-2 (4.1) than for SARS-CoV-1 (3.8), which is not the case for any other HLA-A supertype.

Likewise, to the HLA-A class I alleles, the changes in the spike protein from Khosta-2 generated a significant but not total loss of HLA-B-restricted epitopes associated with the SARS-CoV-2 vaccines (Figure 1B, Table 1 and Table S2). However, up to 82 HLA-B class I alleles (29% of all analyzed) from all supertypes, except from the B08 supertype, remained more than three predicted epitopes conserved among the spike proteins from Khosta-2 and SARS-CoV-2 sarbecoviruses (Table 2). For example, HLA-B*35:21, -B*35:32, -B*35:35, and -B*35:41 alleles from B07 supertype could still bind 7, 7, 8, and 9 fully conserved epitopes among both spike proteins, respectively (Table S2). Similarly, HLA-B*15:62 and -B*15:98, and -B*27:17 from the B27 supertype could bind 12, 11, and 9 fully conserved epitopes among sarbecovirus spike proteins, respectively (Table S2). The supertypes least affected by the 47 additional changes between Khosta-2 and SARS-CoV-1 were B07, B27, and B62 (Figure 1B). Similarly to A03 described above, 30 alleles from the B07 supertype, HLA-B*39:07 and -B*48:02 alleles from the B27 supertype, and five alleles from the HLA-B*52 family from B62 supertype retained more fully conserved epitopes in the spike protein of Khosta-2 than in SARS-CoV-1. The mean of conserved epitopes for the B07 supertype was also slightly higher for Khosta-2 (2.3) than for SARS-CoV-1 (2.1), which is not the case for any other HLA-B supertype.

Supertypes have not been defined for the class I HLA-C locus. However, the occurrence aggregate of only 16 alleles from this locus covers >95% of the world’s population regardless of ethnicity according to the Allele Frequency Net Database. As HLA-A and -B alleles, the changes in the spike protein from Khosta-2 generated a significant but not total loss of HLA-C-restricted epitopes derived from SARS-CoV-2 vaccines (Figure 1B, Table 1 and Table S3). For this locus, most HLA-C alleles analyzed retained more than three conserved epitopes among sarbecovirus spike proteins (Table S3). In addition, 6 of these 16 HLA class I molecules (C*02:02, C*03:03, C*03:04, C*12:03, C*15:02, and C*17:01) retained conserved epitopes, ranging between 8 and 11 (Table S2). As for some of the HLA-A and -B alleles aforementioned, HLA-C*12:03 retained more conserved epitopes in the spike protein of Khosta-2 than in SARS-CoV-1. In contrast, HLA-C*01:02, and -C*07:01 showed only two and three epitopes, respectively. According to the Allele Frequency Net Database, the former allele is very frequent in Far East countries, Oceania aborigine populations, Colombia, and Costa Rica, while the latter is widely distributed over European, African, and Near East countries. In summary, 203 (36%) HLA-A, -B, and -C class I alleles analyzed could bind ≥ 4 conserved epitopes between Khosta-2 and SARS-CoV-2 spike proteins (Table 3).

**Figure 2:**
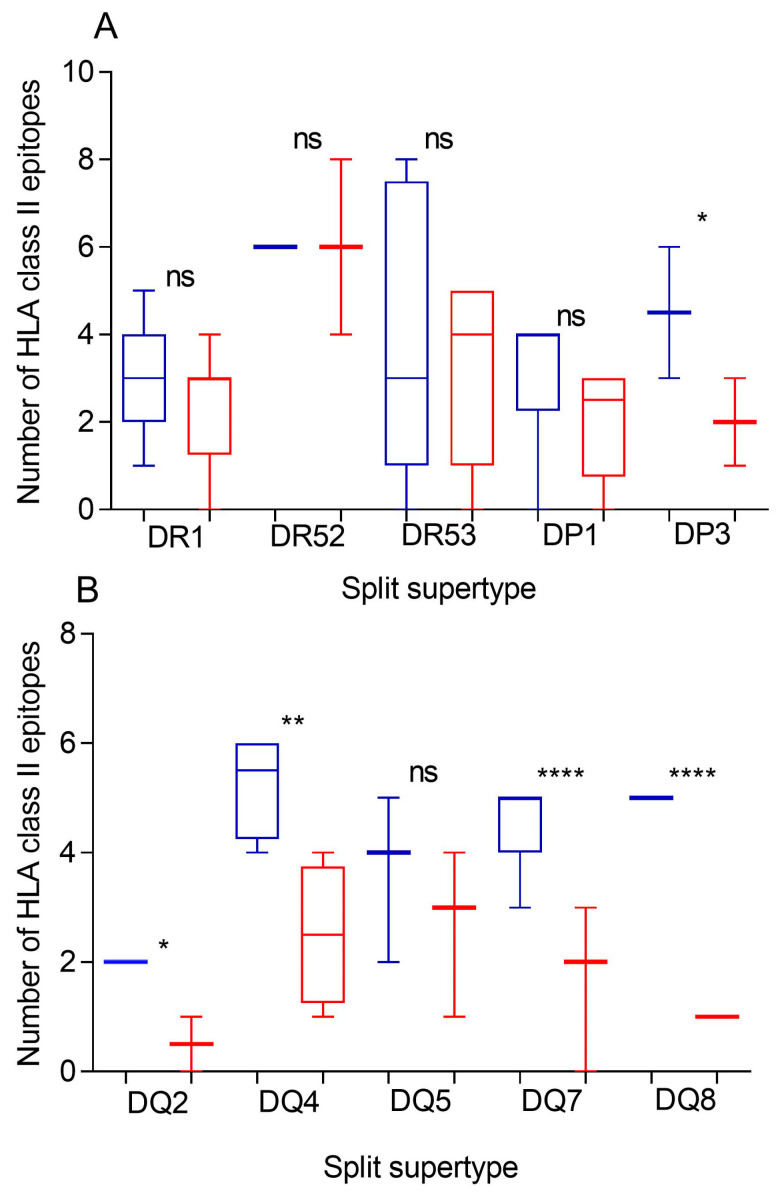
Conservation of Khosta-2 or SARS-CoV-1 epitopes with the SARS-CoV-2 spike protein sequence predicted for the most frequent HLA class II alleles. The number of fully conserved epitopes in Khosta-2 (red), and SARS-CoV-1 (blue) predicted for HLA class II alleles including in the 10 HLA-DR, -DP, or -DQ supertypes are shown in panels (**A**) or (**B**), respectively. The median value is indicated. Box limits indicate the interquartile range. Whiskers are adjusted to maximal and minimal values. Significant *p* values: *, *p* < 0.05; **, *p* < 0.01; ****, *p* < 0.001; ns, non significant.

Additionally, a predictive analysis of the impact of changes described in the Khosta-2 spike protein sequence on class II molecules was carried out. The helper response associated with the HLA class II system plays a crucial, and allele-dependent, pivotal role in the adaptive response against coronaviruses [24]. The 41 HLA-DR, -DP, and -DQ alleles included in the 10 canonical supertypes were considered for the comparison. Similarly to HLA class I-restricted epitopes, changes in Khosta-2 versus SARS-CoV-2 vaccine spike proteins generated a significant but not total loss of HLA class II fully conserved epitopes for the three DR, two DP, and five DQ supertypes analyzed, similarly to HLA class I-restricted epitopes (Figure 2, Table 1 and Table S4). Up to eight HLA class II alleles from DR1, DR52, DR53, DQ4, and DQ5 kept more than three predicted epitopes conserved among the spike proteins from both sarbecoviruses. These alleles constituted 20% of the total HLA class II molecules analyzed (Table 2). Only the DP3 supertype, but not DP1 or the three DR supertypes, was significantly affected by the 47 additional changes between Khosta-2 and SARS-CoV-1 (Figure 2A). In contrast, all supertypes of the DQ locus, except DQ5, showed statistically significant differences between Khosta-2 and SARS-CoV-1 conserved epitopes versus SARS-CoV-2 vaccines (Figure 2B). Strikingly, four HLA-DR alleles (B1*01:01, B1*11:01, B1*15:01, and B3*03:01) retained more conserved epitopes in the spike protein of Khosta-2 than in SARS-CoV-1.

The HLA class I frequencies of the 608 HLA class I molecules analyzed in this study range from low prevalence to 39% (for HLA-A*02:01) of the human population. Thus, the percentage of the human population that might have a sufficient cytolytic immune response against Khosta-2 with the currently licensed SARS-CoV-2 vaccines may be estimated on the basis of the number of retained epitopes. Although a single epitope may be sufficient to generate an HLA-restricted protective immune response [25,26], we focused on the HLA class I alleles that could bind ≥4 predicted epitopes conserved in Khosta-2 and with a world population coverage >1%. Just eight HLA-A class I alleles, HLA-A*02:01, -A*02:06, -A*03:01, -A*11:01, -A*11:02, -A*29:02, -A*68:01, and -A*68:02, cover 71.4% of the human population regardless of ethnicity. Similarly, 10 HLA-B and 14 HLA-C spread alleles with more than 3 epitopes conserved among Khosta-2 and SARS-CoV-2 sarbecoviruses cover 44.6% and 89.3% of the world’s population, respectively. In total, 32 HLA class I alleles covering >95% of the human population regardless of ethnicity could bind more than 3 epitopes conserved between Khosta-2 and SARS-CoV-2 spike proteins. In addition, the 70 HLA class I alleles (32 from A03 supertype, 30 from B07, 2 from B27, 5 from B62, and 1 from HLA-C) that particularly retained more conserved epitopes in the spike protein of Khosta-2 than in SARS-CoV-1 covered 40.1% of the world’s population.

Similarly, the HLA class II alleles with a world population coverage >1% that could bind more than three epitopes conserved in SARS-CoV-2 are indicated in Table 4. Four HLA-DR class II alleles, DRB1*13:01, DRB1*15:01, DRB3*03:01, and DRB4*01:01, cover 71.4% of the human population regardless of ethnicity (Table 4). The other two HLA-DQ alleles frequently cover 23.4% of the world’s population (Table 4). In total, these six HLA class II alleles covering 84.7% of the human population regardless of ethnicity could bind more than three epitopes conserved between Khosta-2 and SARS-CoV-2 spike proteins (Table 4). In addition, the four HLA class II alleles that particularly retained more conserved epitopes in the spike protein of Khosta-2 than in SARS-CoV-1 covered 29.9% of the world’s population.

In summary, currently licensed vaccines against SARS-CoV-2 apparently involve enough conserved epitopes to trigger complete cytotoxic and helper cellular immune responses against the Khosta-2 virus restricted by the most frequent HLA class I and class II alleles expressed by the human population. Strikingly, in a very significant percentage of the population, some HLA-restricted responses could be even superior against Khosta-2 than to SARS-CoV-1, despite the latter being much more closely related to SARS-CoV-2.

**Figure 3:**
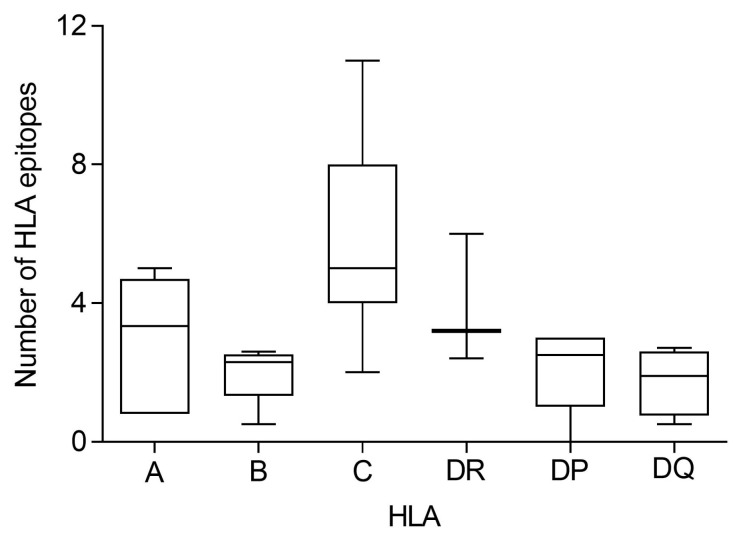
Number of fully conserved epitopes among Khosta-2 and SARS-CoV-2 spike protein sequences predicted for each individual HLA class I and II locus. The median value is indicated. Box limits indicate the interquartile range. Whiskers are adjusted to maximal and minimal values.

For a complete analysis, it should be considered that HLA loci are tightly linked in the human genome. Thus, the set of HLA-A, -B, -C, -DR, -DP, and -DQ genes, called the HLA haplotype, is commonly co-inherited in a Mendelian fashion from the paternal gametes. Therefore, the number of conserved epitopes among the Khosta-2 and SARS-CoV-2 spike protein sequences predicted for all HLA class I and II alleles were analyzed by HLA loci. An average of three, two, and six conserved epitopes for HLA-A, -B, and -C loci, respectively, and four, two, and two conserved epitopes for each HLA-DR, -DP, and -DQ loci, respectively (Figure 3). Thus, on average, 11 conserved epitopes of HLA class I and 8 of HLA class II could be associated with each individual HLA haplotype (Figure 3), and the different HLA molecules in a homozygous individual would present these 19 conserved epitopes. However, as less than 15% of humans are homozygotes for HLA [27], the currently licensed vaccines against SARS-CoV-2 could generate an average of 22 HLA class I and 16 HLA class II epitopes, respectively, conserved in Khosta-2 for more than 85% of the human population.

**Figure 4:**
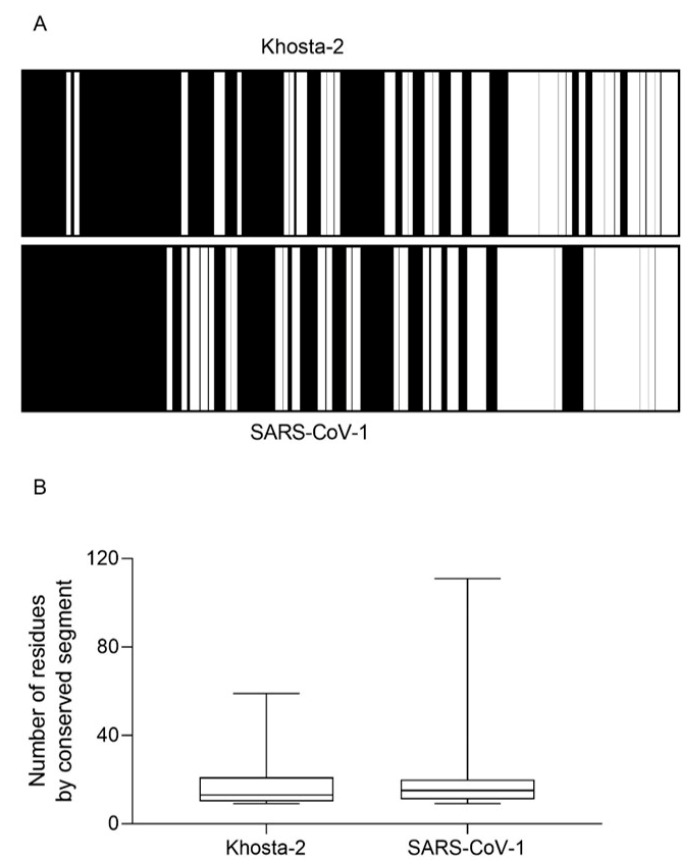
Conservation of Khosta-2 or SARS-CoV-1 spike proteins with their SARS-CoV-2 homolog. Panel (**A**): Conserved (white) or non-conserved (black) segments with ≥9 consecutive residues between SARS-CoV-2 spike protein and their homologs in Khosta-2 or SARS-CoV-1. The number of conserved segments with ≥9 consecutive residues between SARS-CoV-2 spike protein versus Khosta-2 or SARS-CoV-1 is depicted in panel (**B**). The median value is indicated. Box limits indicate the interquartile range. Whiskers are adjusted to maximal and minimal values.

This somehow surprising relative abundance of perfectly conserved epitopes between the Khosta-2 and SARS-CoV-2 spike proteins is likely because viral proteins cannot evenly mutate but there are regions that are subjected to strong structural and functional selective pressures. Thus, despite accumulating 351 changes, there are 31 sequence segments involving between 9 (i.e., the minimal length for most cellular epitopes) and 59 consecutive residues that are fully conserved among Khosta-2 and SARS-CoV-2 spike proteins (Figure 4). Thus, the immune system can take advantage of up to 522 identical amino acids of the Khosta-2 spike sequence to generate HLA-restricted epitopes conserved with SARS-CoV-2. Likewise, the spike protein from SARS-CoV-1 presents 579 conserved residues included in segments between 9 and 111 consecutive residues with the current pandemic coronavirus (Figure 4), which suffices for T cellular cross-protection [12]. Notably, a study similar to the present one reported that, on average, SARS-CoV-1 retained 40% more conserved HLA epitopes with SARS-CoV-2 vaccines than those identified for Khosta-2 [11].

An important point of this study is that the viral epitopes were computationally predicted. Nevertheless, since no single T-cell epitope has been experimentally defined for the Khosta-2 virus, current computational tools allow for fast and human allelically universal accurate predictions [28]. This may further guide the experimental confirmation design of at least the most relevant results. In the current pandemic context, the number of experimentally identified HLA-restricted epitopes of SARS-CoV-2 included in the IEDB database is steadily increasing [29]. Obviously, the most frequent HLA alleles in the population are also the most studied. Therefore, a search of the IEDB database for conserved epitopes between Khosta-2 and SARS-CoV-2 was performed for those HLA class I alleles with global population coverage >5% and with ≥4 predicted conserved epitopes between these two sarbecoviruses. The overwhelming majority of the HLA-A-restricted epitopes predicted in the present study have been identified by different laboratories around the world as epitopes recognized by cytolytic CD8^+^ T cells (Table 5). The match percentage between predicted and experimentally detected epitopes ranged from 75% (for HLA-A*11:01) to 100% (for HLA-A*02:01, -A*03:01, and -A*68:01) (Table 5). In these four HLA-A alleles, which cover 66.1% of the world’s population, 92% of the predicted epitopes were functionally detected (Table 5). Furthermore, in the five HLA-B alleles analyzed (B*15:01, B*35:01, B*40:01, B*44:02, and B*44:03) covering 35.5% of the world’s population, 90% of the predicted epitopes were included in the IEDB database as confirmed cytotoxic epitopes (Table 5). In addition, the three most frequent alleles for the HLA-C locus (C*04:01, C*06:02, and C*07:02), which cover 51.1% of the world’s population, 85% of the predicted epitopes were also functionally detected as targets of CD8^+^ T lymphocytes (Table 5). In total, 54 of the 60 predicted epitopes (90%) associated with the 12 HLA class I molecules analyzed, which combined cover 89.1% of the world’s population, are included in the IEDB database as HLA-restricted functional epitopes. Similarly, peptides linked to frequent HLA class II alleles with ≥4 predicted epitopes conserved between both sarbecoviruses and global population coverage >4% were searched in the IEDB resource. Only one of the 21 predicted HLA-DR-restricted epitopes, which was associated with HLA-DRB4*01:01, was not included in the IEDB as a verified CD4^+^ T-lymphocyte target (Table 6). In addition, seven of the eight predicted epitopes associated with the two frequent HLA-DQ alleles were experimentally identified as targets of CD4+ T lymphocytes (Table 6). In total, 93% of the predicted epitopes associated with these six HLA class II alleles, which cover 66.7% of the world’s population, were experimentally included in the IEDB database as HLA-restricted functional epitopes (Table 6). These results for both class I and class II HLA, show a stunningly high correlation between immunoinformatic predictions and functional assays concerning conserved epitopes among sarbecovirus spike proteins. This gives credence to the methodological approach developed in this study. In addition, it should be noted that epitopes predicted in the present analysis, but not currently included in the IEDB database, may not have been tested experimentally yet. Therefore, they could even be identified as novel epitopes recognized by T lymphocytes in future studies further enhancing the utility of our immunoinformatic analysis.

Using the same immunoinformatic approach as the one employed here, we previously concluded that both cytotoxic and helper cellular immune protection elicited by currently licensed COVID-19 vaccines should be effective against SARS-CoV-1 infection [11]. In support of our argument, vaccination against SARS-CoV-1 in mice protects against SARS-CoV-2 challenge [12]. Thus, although the Khosta-2 spike protein carries less conserved residues than the SARS-CoV-1 versus SARS-CoV-2 homolog, the striking relative average abundance of 38 conserved epitopes in heterozygous individuals supports global cellular protection against Khosta-2 if it massively infects the human population. In addition, there is another fact to consider. Due to the lack of adequate bioinformatics tools, possible non-conserved cross-reactive epitopes (differing by one or more residues between SARS-CoV-2 and Khosta-2 sequences), peptides that could also be relevant for immune recognition and increase the cross-reactivity between sarbecoviruses, were not analyzed in our study. Thus, the cross-reactivity that we identified in our study (based only on the fully conserved epitopes) would actually be a minimum value that would likely be increased by some non-conserved epitopes with cross-reactivity between SARS-CoV-2 and Khosta-2 viruses. Thus, new shots of current COVID-19 vaccines would be sufficient to effectively fight the spread of this novel virus. This conclusion of our study would be formally confirmed with in vivo studies using animal models immunized with SARS-CoV-2 vaccines and later infected with the Khosta-2 virus. These experiments should include analysis of viral load, disease progression, immune responses, and survival of Khosta-2 infected animals.

Although all licensed formulations against SARS-CoV-2 distributed in most developed countries used only the original sequence of the spike protein of the wild-type Wuhan-1 strain as the immunogen, inactivated whole virus vaccines have also been distributed in various populated regions such as Africa, Asia, or South America. High efficacy and effectiveness have been demonstrated with these vaccines, which contain the complete proteome of the virus [30]. It is then obvious that with immunization with the whole virus, the number of epitopes fully conserved in the Khosta-2 virus would be much higher than what we have identified in the present study with only those corresponding to the spike protein of SARS-CoV-2, resulting in increased T cell-mediated cross-protection with these inactivated vaccines. Thus, new injections of inactivated whole SARS-CoV-2 vaccines could be much more effective against the spread of Khosta-2. In this context, T cells recognizing epitopes of non-structural proteins from the phylogenetically more distant alpha and betacoronaviruses, pathogens that cause the common cold, have also been shown to be cross-reactive epitopes during COVID-19 [31].

Intramuscular administration is used for all COVID-19 formulations. However, it is well known that the immune response in the upper respiratory tract induced by this intramuscular administration is limited. Animal models indicate that intranasal immunization against infections such as SARS-CoV-2 produces a better response in the upper respiratory tract, potentiating the immune response and reducing morbidity and mortality (reviewed in [32]). Therefore, the current trend in the field of respiratory vaccines is to develop protocols and clinical trials for intranasal delivery of vaccines and to obtain regulatory approval. Among the topics covered in the current study, the use of repositioning vaccines designed against one virus and reused against another, intranasal administration would be a very good strategy to increase the efficacy and degree of protection of the vaccines against respiratory pathogens such as coronaviruses.

Finally, the present study has important public health implications for future pandemics, since the use of these immunoinformatic analyses and a similar exploration of cross-reactive humoral responses could be a useful rapid response strategy to confront future pandemics (produced by any new coronavirus or by viruses of a different family) with the vaccine tools available at any given time.

## 3. Materials and Methods

### 3.1. Selection of Antigenic Proteins

The spike protein of the SARS-CoV-2 reference proteome (Wuhan-1, RefSeq: NC_045512.2) was initially selected. In addition, the modifications of the spike protein, which were added to Moderna mRNA-1273, Pfizer BNT162b2, and Janssen Ad26.COV2.S vaccines, were also included. The spike protein of the Khosta-2 was deduced from the reported complete viral genome (GenBank: MZ190138.1).

### 3.2. Selection of HLA Class I and II Alleles

HLA class I alleles that share anchor residues of the 551 alleles including in the 12 HLA class I supertypes (A01, A0103, A0124, A02, A03, A24, B07, B08, B27, B44, B58, and B62) [18], and the 16 most frequent HLA-C alleles were selected. In addition, the 41 alleles including in the 10 HLA class II supertypes (DR1, DR52, DR53, DP1, DP3, DQ2, DQ4, DQ5, DQ7, and DQ8) [19,20] were also included in the study. The HLA class I and II alleles included in these supertypes cover >90% and >95% of the world’s population, respectively, regardless of ethnicity [18,19,20].

### 3.3. HLA Class I Epitope Prediction

Non-redundant HLA class I epitopes between 8 and 12 residues from the spike protein of the SARS-CoV-2 (including the modifications of the spike protein added to Moderna mRNA-1273, Pfizer BNT162b2, and Janssen Ad26.COV2.S vaccines) were predicted using the latest versions of the universal and artificial neural network-based netMHCpan EL and BA algorithms. These bioinformatics tools outperform any other method so far [28] and are recommended by the central Immune Epitope Database and Analysis Resource [29]. First, the peptides considered “Strong Binders” (rank ≤0.5 and score ≥0.5) by NetMHCIpan EL 4.1 [28] for HLA class I ligands were selected. For redundant epitopes, those sharing the same binding core for the same allele, only the one with the highest score was considered per allele. As the NetMHCIpan EL 4.1 algorithm was trained only with mass spectrometry elution (EL) data, non-redundant epitopes were further verified through the NetMHCIpan BA 4.1 [28] algorithm, which includes binding affinity (BA) data and thus, the combination of both bioinformatics tools yield the most accurate results. These verified non-redundant epitopes did not match any of those predicted for a random sequence with the same length and residue composition as the reference SARS-CoV-2 spike protein generated with the EXPASY RandSeq tool (https://web.expasy.org/randseq/, accessed on 1 April 2024). Predictions were carried out with the 567 HLA-A, -B, and -C alleles previously selected. To further test the specificity and sensitivity of the NetMHCIpan EL 4.1 algorithm, substitution of all Pro and Arg/Gln by Ala yielded no epitopes for HLA-B*07:02 and HLA-B*27:05 alleles, respectively, as these amino acids are their respective anchor motif residues.

### 3.4. HLA Class II Epitope Prediction

Similarly to HLA class I, non-redundant HLA class II epitopes between 12 and 18 residues were predicted using the NetMHCIIpan EL 4.0 [28]. Only the “Strong Binders” (rank ≤ 0.5) were considered with this algorithm and later were further verified through the NetMHCIIpan BA 4.0 [28] algorithm. As for HLA class I, these verified non-redundant HLA class II epitopes did not match any of those predicted for a random sequence with the same length and residue composition as the reference SARS-CoV-2 spike protein generated with the EXPASY RandSeq tool (https://web.expasy.org/randseq/, accessed on 1 April 2024). Predictions were carried out with the 41 HLA-DR, -DP, and -DQ alleles previously selected.

### 3.5. Other Analyses

Data on experimentally detected epitopes was obtained from the Immune Epitope Database and Analysis Resource (IEDB; http://www.iedb.org, accessed on 2 April 2024) [29]. A search in IEDB of the predicted epitopes for the most abundant HLA class I and II alleles in the population was carried out. Positive response for activation and/or cytokine secretion T cell assay was manually confirmed in the original article describing each epitope. Data on population coverage by HLA class I and II molecules were obtained from IEDB; http://tools.iedb.org/population/; accessed on 10 December 2022) [29].

## Figures and Tables

**Table 1:** Percentage of predicted HLA class I and class II epitopes fully conserved among KHOSTA-2 and SARS-CoV-2 spike proteins.

Supertype	% of Fully Conserved Epitopes with SARS-CoV-2
A01	5
A0103	12
A0124	21
A02	25
A03	18
A24	7
B07	20
B08	8
B27	23
B44	23
B58	9
B64	7
HLA-C	20
DR1	19
DR52	29
DR53	32
DP1	13
DP3	15
DQ2	6
DQ4	18
DQ5	23
DQ7	16
DQ8	7

**Table 2:** Number of HLA alleles with more than three predicted epitopes fully conserved among KHOSTA-2 and SARS-CoV-2 spike proteins.

HLA Superfamily	Number of HLA Alleles
A01	2
A0103	0
A0124	7
A02	49
A03	44
A24	5
HLA-A	107
B07	14
B08	0
B27	17
B44	31
B58	3
B62	17
HLA-B	82
HLA-C	14
Nº of HLA class I alleles (%)	203 (36%)
DR1	1
DR52	2
DR53	3
DP1	0
DP3	0
DQ2	0
DQ4	1
DQ5	1
DQ7	0
DQ8	0
Nº of HLA class II alleles (%)	8 (20%)

**Table 3:** Predicted epitopes fully conserved in KHOSTA-2 and % population coverage in the most frequent HLA class I alleles.

HLA Class I Allele	Supertype	Epitopes Fully Conserved in KHOSTA-2	% Population Coverage ^a^
A*29:02	A0124	6	3.9
A*02:01	A02	7	39.1
A*02:06	A02	5	2.0
A*68:02	A02	5	2.5
A*03:01	A03	7	16.8
A*11:01	A03	8	15.5
A*11:02	A03	8	1.1
A*68:01	A03	4	5.8
8 HLA-A alleles			71.4
B*35:01	B07	5	8.4
B*15:03	B27	7	1.3
B*27:05	B27	5	4.8
B*39:02	B27	4	1.0
B*40:01	B44	4	7.8
B*40:02	B44	4	3.5
B*44:02	B44	4	7.6
B*44:03	B44	4	6.7
B*15:01	B62	4	8.4
B*15:25	B62	4	1.6
10 HLA-B alleles			44.6
18 HLA-A, and -B alleles			84.2
C*02:02		11	9.5
C*03:03		8	8.1
C*03:04		8	12.8
C*04:01		5	20.0
C*05:01		4	7.9
C*06:02		4	15.5
C*07:02		4	21.5
C*08:01		5	4.6
C*08:02		4	4.2
C*12:03		8	10.3
C*14:02		5	3.0
C*15:02		8	4.4
C*16:01		5	4.7
C*17:01		8	3.3
14 HLA-C alleles			89.3
32 HLA class I alleles			>95

^a^ Only the HLA class I molecules with a world population coverage >1% were included.

**Table 4:** Predicted epitopes fully conserved in KHOSTA-2 and % population coverage in the most frequent HLA class II alleles.

HLA Class II Allele	Supertype	Epitopes Fully Conserved in KHOSTA-2	% Population Coverage ^a^
DRB1*15:01	DR1	4	18.4
DRB3*03:01	DR52	8	4.0
DRB1*13:02	DR52	4	6.7
DRB4*01:01	DR53	5	41.8
4 HLA-DR alleles			71.4
HLA-DQA1*03:03-DQB1*04:02	DQ4	4	16.0
HLA-DQA1*01:04-DQB1*05:03	DQ5	4	13.8
2 HLA-DQ alleles			23.4
6 HLA class II alleles			84.7

^a^ Only the HLA class II molecules with a world population coverage >1% were included.

**Table 5:** HLA class I predicted and experimentally detected epitopes fully conserved among sarbecoviruses.

HLA Class I Allele	HLA Class I Epitopes Conserved among Sarbecoviruses			% Population Coverage ^c^
	Predicted ^a^	Experimentally Confirmed ^b^	% Experimental/Predicted	
A*02:01	7	7	100	39.1
A*03:01	7	7	100	16.8
A*11:01	8	6	75	15.5
A*68:01	4	4	100	5.8
4 HLA-A alleles	26	24	92	66.1
B*35:01	5	4	80	8.4
B*40:01	4	4	100	7.8
B*44:02	4	4	75	7.6
B*44:03	4	3	75	6.7
B*15:01	4	4	100	8.4
5 HLA-B alleles	21	19	90	35.5
C*04:01	5	4	80	20.0
C*06:02	4	4	100	15.5
C*07:02	4	3	75	21.5
3 HLA-C alleles	13	11	85	51.1
12 HLA class I alleles	60	54	90	89.3

^a^ From this study (Table 3). ^b^ Positive for activation and/or cytokine secretion T cell assays obtained from the IEDB database. ^c^ Only the HLA-A, and -B class I molecules with a world population coverage >5% and HLA-C class I molecules with a world population coverage >15% were included.

**Table 6:** HLA class II predicted and experimentally detected epitopes fully conserved among sarbecoviruses.

HLA Class II Allele	HLA Class II Epitopes Conserved among Sarbecoviruses			% Population Coverage ^c^
	Predicted ^a^	Experimentally Confirmed ^b^	% Experimental/Predicted	
DRB1*15:01	4	4	100	18.4
DRB3*03:01	8	8	100	4.0
DRB1*13:02	4	4	100	6.7
DRB4*01:01	5	4	80	41.8
4 HLA-DR alleles	21	20	95	61.4
HLA-DQA1*03:03-DQB1*04:02	4	3	75	16.0
HLA-DQA1*01:04-DQB1*05:03	4	4	100	13.8
2 HLA-DQ alleles	8	7	88	23.4
6 HLA class II alleles	29	27	93	66.7

^a^ From this study (Table 4). ^b^ Positive for activation and/or cytokine secretion T cell assays obtained from the IEDB database. ^c^ Only HLA class II alleles with a world population coverage ≥4% were included.

## Data Availability

All data of this study are included in the article and supplemental tables.

## Supplementary Materials

The following supporting information can be downloaded at: https://www.mdpi.com/article/10.3390/ijms25116087/s1.
