# Supplementary material for: Conservation of HLA Spike Protein Epitopes Supports T Cell Cross-Protection in SARS-CoV-2 Vaccinated Individuals against the Potentially Zoonotic Coronavirus Khosta-2
Source: Int J Mol Sci. 2024 May 31;25(11):6087. doi: 10.3390/ijms25116087 (PMC11172828; doi:10.3390/ijms25116087)
Supplement: Supplementary file 1 [file ijms-25-06087-s001.zip › Supplemental Table 1.pdf]

Supplemental Table 1

Supertype	Allele	Position	Peptide	Score (EL)
A01	HLA-A*01:01	28	YTNSFTRGVY	0,8696
A01	HLA-A*01:01	28	YTNSFTRGVYY	0,9035
A01	HLA-A*01:01	135	FCNDPFLGVY	0,6253
A01	HLA-A*01:01	136	CNDPFLGVY	0,6408
A01	HLA-A*01:01	136	CNDPFLGVYY	0,6059
A01	HLA-A*01:01	160	YSSANNCTFEY	0,7273
A01	HLA-A*01:01	161	SSANNCTFEY	0,7703
A01	HLA-A*01:01	162	SANNCTFEY	0,6674
A01	HLA-A*01:01	256	SGWTAGAAAYY	0,7466
A01	HLA-A*01:01	257	GWTAGAAAYY	0,7407
A01	HLA-A*01:01	258	WTAGAAAYY	0,9622
A01	HLA-A*01:01	358	ISNCVADYSVLY	0,7628
A01	HLA-A*01:01	361	CVADYSVLY	0,8233
A01	HLA-A*01:01	362	VADYSVLY	0,8361
A01	HLA-A*01:01	370	NSASFSTFKCY	0,5177
A01	HLA-A*01:01	440	NLDSKVGGNY	0,893
A01	HLA-A*01:01	603	NTSNQVAVLY	0,8721
A01	HLA-A*01:01	604	TSNQVAVLY	0,9585
A01	HLA-A*01:01	746	STECSNLLLQY	0,8875
A01	HLA-A*01:01	828	LADAGFIKQY	0,928
A01	HLA-A*01:01	863	PLLTDEMIAQY	0,8726
A01	HLA-A*01:01	865	LTDEMIAQY	0,9987
A01	HLA-A*01:01	866	TDEMIAQY	0,8997
A01	HLA-A*01:01	1197	LIDLQELGKY	0,9031
A01	HLA-A*01:03	28	YTNSFTRGVY	0,8142
A01	HLA-A*01:03	28	YTNSFTRGVYY	0,8662
A01	HLA-A*01:03	160	YSSANNCTFEY	0,6327
A01	HLA-A*01:03	161	SSANNCTFEY	0,6876
A01	HLA-A*01:03	258	WTAGAAAYY	0,9493
A01	HLA-A*01:03	358	ISNCVADYSVLY	0,6647
A01	HLA-A*01:03	361	CVADYSVLY	0,7732
A01	HLA-A*01:03	603	NTSNQVAVLY	0,8133
A01	HLA-A*01:03	604	TSNQVAVLY	0,9385
A01	HLA-A*01:03	746	STECSNLLLQY	0,8277
A01	HLA-A*01:03	828	LADAGFIKQY	0,8857
A01	HLA-A*01:03	863	PLLTDEMIAQY	0,7915
A01	HLA-A*01:03	865	LTDEMIAQY	0,9982
A01	HLA-A*01:03	1197	LIDLQELGKY	0,8476
A01	HLA-A*01:06	28	YTNSFTRGVY	0,7602
A01	HLA-A*01:06	28	YTNSFTRGVYY	0,7892
A01	HLA-A*01:06	29	TNSFTRGVYY	0,5721
A01	HLA-A*01:06	30	NSFTRGVYY	0,8159
A01	HLA-A*01:06	160	YSSANNCTFEY	0,5052
A01	HLA-A*01:06	161	SSANNCTFEY	0,7022
A01	HLA-A*01:06	162	SANNCTFEY	0,6641
A01	HLA-A*01:06	256	SGWTAGAAAYY	0,6894
A01	HLA-A*01:06	257	GWTAGAAAYY	0,7487
A01	HLA-A*01:06	258	WTAGAAAYY	0,9282

A01	HLA-A*01:06	358	ISNCVADYSVLY	0,607
A01	HLA-A*01:06	359	SNCVADYSVLY	0,5742
A01	HLA-A*01:06	360	NCVADYSVLY	0,6328
A01	HLA-A*01:06	361	CVADYSVLY	0,8242
A01	HLA-A*01:06	601	GTNTSNQVAVLY	0,5577
A01	HLA-A*01:06	603	NTSNQVAVLY	0,7856
A01	HLA-A*01:06	604	TSNQVAVLY	0,9241
A01	HLA-A*01:06	733	KTSVDCTMY	0,6247
A01	HLA-A*01:06	746	STECSNLLLQY	0,5958
A01	HLA-A*01:06	828	LADAGFIKQY	0,7349
A01	HLA-A*01:06	865	LTDEMIAQY	0,9873
A01	HLA-A*01:06	1197	LIDLQELGKY	0,6364
A01	HLA-A*01:07	28	YTNSFTRGVY	0,7994
A01	HLA-A*01:07	28	YTNSFTRGVYY	0,7935
A01	HLA-A*01:07	135	FCNDPFLGVY	0,5269
A01	HLA-A*01:07	136	CNDPFLGVY	0,5497
A01	HLA-A*01:07	136	CNDPFLGVYY	0,5021
A01	HLA-A*01:07	160	YSSANNCTFEY	0,6031
A01	HLA-A*01:07	161	SSANNCTFEY	0,7204
A01	HLA-A*01:07	162	SANNCTFEY	0,6395
A01	HLA-A*01:07	256	SGWTAGAAAYY	0,6935
A01	HLA-A*01:07	257	GWTAGAAAYY	0,7199
A01	HLA-A*01:07	258	WTAGAAAYY	0,9322
A01	HLA-A*01:07	358	ISNCVADYSVLY	0,6325
A01	HLA-A*01:07	361	CVADYSVLY	0,7815
A01	HLA-A*01:07	440	NLDSKVGGNY	0,8818
A01	HLA-A*01:07	603	NTSNQVAVLY	0,8153
A01	HLA-A*01:07	604	TSNQVAVLY	0,9147
A01	HLA-A*01:07	746	STECSNLLLQY	0,728
A01	HLA-A*01:07	828	LADAGFIKQY	0,8525
A01	HLA-A*01:07	863	PLLTDEMIAQY	0,7608
A01	HLA-A*01:07	865	LTDEMIAQY	0,9944
A01	HLA-A*01:07	1197	LIDLQELGKY	0,8505
A01	HLA-A*01:08	28	YTNSFTRGVY	0,8357
A01	HLA-A*01:08	28	YTNSFTRGVYY	0,8794
A01	HLA-A*01:08	135	FCNDPFLGVY	0,5792
A01	HLA-A*01:08	136	CNDPFLGVY	0,5738
A01	HLA-A*01:08	136	CNDPFLGVYY	0,5287
A01	HLA-A*01:08	160	YSSANNCTFEY	0,71
A01	HLA-A*01:08	161	SSANNCTFEY	0,7846
A01	HLA-A*01:08	162	SANNCTFEY	0,6944
A01	HLA-A*01:08	254	SSSGWTAGAAAY	0,5093
A01	HLA-A*01:08	256	SGWTAGAAAYY	0,748
A01	HLA-A*01:08	257	GWTAGAAAYY	0,7602
A01	HLA-A*01:08	258	WTAGAAAYY	0,9628
A01	HLA-A*01:08	358	ISNCVADYSVLY	0,7216
A01	HLA-A*01:08	361	CVADYSVLY	0,8161
A01	HLA-A*01:08	362	VADYSVLY	0,7907
A01	HLA-A*01:08	603	NTSNQVAVLY	0,8442
A01	HLA-A*01:08	604	TSNQVAVLY	0,9539

A01	HLA-A*01:08	697	MSLGAENSVAY	0,5924
A01	HLA-A*01:08	733	KTSVDCTMY	0,6742
A01	HLA-A*01:08	746	STECSNLLLQY	0,8198
A01	HLA-A*01:08	828	LADAGFIKQY	0,9
A01	HLA-A*01:08	863	PLLTDEMIAQY	0,7815
A01	HLA-A*01:08	865	LTDEMIAQY	0,9976
A01	HLA-A*01:08	866	TDEMIAQY	0,8381
A01	HLA-A*01:08	1197	LIDLQELGKY	0,8632
A01	HLA-A*01:09	28	YTNSFTRGVY	0,8696
A01	HLA-A*01:09	28	YTNSFTRGVYY	0,9035
A01	HLA-A*01:09	135	FCNDPFLGVY	0,6253
A01	HLA-A*01:09	136	CNDPFLGVY	0,6408
A01	HLA-A*01:09	136	CNDPFLGVYY	0,6059
A01	HLA-A*01:09	160	YSSANNCTFEY	0,7273
A01	HLA-A*01:09	161	SSANNCTFEY	0,7703
A01	HLA-A*01:09	162	SANNCTFEY	0,6674
A01	HLA-A*01:09	256	SGWTAGAAAYY	0,7466
A01	HLA-A*01:09	257	GWTAGAAAYY	0,7407
A01	HLA-A*01:09	258	WTAGAAAYY	0,9622
A01	HLA-A*01:09	358	ISNCVADYSVLY	0,7628
A01	HLA-A*01:09	361	CVADYSVLY	0,8233
A01	HLA-A*01:09	362	VADYSVLY	0,8361
A01	HLA-A*01:09	370	NSASFSTFKCY	0,5177
A01	HLA-A*01:09	440	NLDSKVGGNY	0,893
A01	HLA-A*01:09	603	NTSNQVAVLY	0,8721
A01	HLA-A*01:09	604	TSNQVAVLY	0,9585
A01	HLA-A*01:09	746	STECSNLLLQY	0,8875
A01	HLA-A*01:09	828	LADAGFIKQY	0,928
A01	HLA-A*01:09	863	PLLTDEMIAQY	0,8726
A01	HLA-A*01:09	865	LTDEMIAQY	0,9987
A01	HLA-A*01:09	866	TDEMIAQY	0,8997
A01	HLA-A*01:09	1197	LIDLQELGKY	0,9031
A01	HLA-A*01:10	28	YTNSFTRGVY	0,8782
A01	HLA-A*01:10	28	YTNSFTRGVYY	0,8568
A01	HLA-A*01:10	135	FCNDPFLGVY	0,5464
A01	HLA-A*01:10	136	CNDPFLGVY	0,55
A01	HLA-A*01:10	160	YSSANNCTFEY	0,617
A01	HLA-A*01:10	161	SSANNCTFEY	0,7739
A01	HLA-A*01:10	162	SANNCTFEY	0,7209
A01	HLA-A*01:10	254	SSSGWTAGAAAY	0,5306
A01	HLA-A*01:10	256	SGWTAGAAAYY	0,7805
A01	HLA-A*01:10	257	GWTAGAAAYY	0,801
A01	HLA-A*01:10	258	WTAGAAAYY	0,9558
A01	HLA-A*01:10	269	YLQPRTFLLKY	0,5653
A01	HLA-A*01:10	358	ISNCVADYSVLY	0,7038
A01	HLA-A*01:10	361	CVADYSVLY	0,8467
A01	HLA-A*01:10	362	VADYSVLY	0,7818
A01	HLA-A*01:10	414	QTGKIADYNY	0,7033
A01	HLA-A*01:10	440	NLDSKVGGNY	0,8551
A01	HLA-A*01:10	601	GTNTSNQVAVLY	0,6737

A01	HLA-A*01:10	603	NTSNQVAVLY	0,8545
A01	HLA-A*01:10	604	TSNQVAVLY	0,9462
A01	HLA-A*01:10	733	KTSVDCTMY	0,7195
A01	HLA-A*01:10	746	STECSNLLLQY	0,8093
A01	HLA-A*01:10	828	LADAGFIKQY	0,8825
A01	HLA-A*01:10	863	PLLTDEMIAQY	0,6841
A01	HLA-A*01:10	865	LTDEMIAQY	0,996
A01	HLA-A*01:10	1039	RVDFCGKGY	0,8273
A01	HLA-A*01:10	1197	LIDLQELGKY	0,875
A01	HLA-A*01:12	28	YTNSFTRGVY	0,5329
A01	HLA-A*01:12	28	YTNSFTRGVYY	0,528
A01	HLA-A*01:12	161	SSANNCTFEY	0,5055
A01	HLA-A*01:12	162	SANNCTFEY	0,5654
A01	HLA-A*01:12	258	WTAGAAAYY	0,8102
A01	HLA-A*01:12	361	CVADYSVLY	0,7278
A01	HLA-A*01:12	444	KVGGNYNYLY	0,594
A01	HLA-A*01:12	603	NTSNQVAVLY	0,5927
A01	HLA-A*01:12	604	TSNQVAVLY	0,7812
A01	HLA-A*01:12	865	LTDEMIAQY	0,9207
A01	HLA-A*01:14	28	YTNSFTRGVY	0,8208
A01	HLA-A*01:14	28	YTNSFTRGVYY	0,8729
A01	HLA-A*01:14	135	FCNDPFLGVY	0,6119
A01	HLA-A*01:14	136	CNDPFLGVY	0,6715
A01	HLA-A*01:14	136	CNDPFLGVYY	0,6088
A01	HLA-A*01:14	160	YSSANNCTFEY	0,663
A01	HLA-A*01:14	161	SSANNCTFEY	0,7519
A01	HLA-A*01:14	162	SANNCTFEY	0,6665
A01	HLA-A*01:14	256	SGWTAGAAAYY	0,6944
A01	HLA-A*01:14	257	GWTAGAAAYY	0,7187
A01	HLA-A*01:14	258	WTAGAAAYY	0,9506
A01	HLA-A*01:14	269	YLQPRTFLLKY	0,5565
A01	HLA-A*01:14	358	ISNCVADYSVLY	0,7087
A01	HLA-A*01:14	361	CVADYSVLY	0,8057
A01	HLA-A*01:14	362	VADYSVLY	0,7998
A01	HLA-A*01:14	601	GTNTSNQVAVLY	0,6797
A01	HLA-A*01:14	603	NTSNQVAVLY	0,8237
A01	HLA-A*01:14	604	TSNQVAVLY	0,9452
A01	HLA-A*01:14	733	KTSVDCTMY	0,7034
A01	HLA-A*01:14	746	STECSNLLLQY	0,8081
A01	HLA-A*01:14	828	LADAGFIKQY	0,8737
A01	HLA-A*01:14	863	PLLTDEMIAQY	0,7423
A01	HLA-A*01:14	865	LTDEMIAQY	0,9971
A01	HLA-A*01:14	1039	RVDFCGKGY	0,8252
A01	HLA-A*01:14	1197	LIDLQELGKY	0,8561
A01	HLA-A*26:01	28	YTNSFTRGVY	0,5472
A01	HLA-A*26:01	191	EFVFKNIDGY	0,8157
A01	HLA-A*26:01	192	FVFKNIDGY	0,9119
A01	HLA-A*26:01	258	WTAGAAAYY	0,9338
A01	HLA-A*26:01	340	EVFNATRFASVY	0,6393
A01	HLA-A*26:01	360	NCVADYSVLY	0,7886

A01	HLA-A*26:01	361	CVADYSVLY	0,8578
A01	HLA-A*26:01	603	NTSNQVAVLY	0,5793
A01	HLA-A*26:01	686	SVASQSIIAY	0,6831
A01	HLA-A*26:01	780	EVFAQVKQIY	0,7706
A01	HLA-A*26:01	1095	FVSNGTHWF	0,5675
A01	HLA-A*26:02	28	YTNSFTRGVY	0,7649
A01	HLA-A*26:02	30	NSFTRGVYY	0,7196
A01	HLA-A*26:02	50	STQDLFLPF	0,7114
A01	HLA-A*26:02	125	NVVIKVCEF	0,6379
A01	HLA-A*26:02	191	EFVFKNIDGY	0,9206
A01	HLA-A*26:02	192	FVFKNIDGY	0,9721
A01	HLA-A*26:02	192	FVFKNIDGYF	0,7022
A01	HLA-A*26:02	215	DLPQGFSAL	0,5481
A01	HLA-A*26:02	256	SGWTAGAAAYY	0,5903
A01	HLA-A*26:02	257	GWTAGAAAYY	0,6462
A01	HLA-A*26:02	258	WTAGAAAY	0,5731
A01	HLA-A*26:02	258	WTAGAAAYY	0,9678
A01	HLA-A*26:02	261	GAAAYYVGY	0,6857
A01	HLA-A*26:02	298	ETKCTLKSF	0,7757
A01	HLA-A*26:02	340	EVFNATRF	0,5818
A01	HLA-A*26:02	340	EVFNATRFASVY	0,834
A01	HLA-A*26:02	343	NATRFASVY	0,526
A01	HLA-A*26:02	359	SNCVADYSVLY	0,6372
A01	HLA-A*26:02	360	NCVADYSVLY	0,9187
A01	HLA-A*26:02	361	CVADYSVLY	0,9515
A01	HLA-A*26:02	366	SVLYNSASF	0,6928
A01	HLA-A*26:02	392	FTNVYADSF	0,6269
A01	HLA-A*26:02	442	DSKVGGNYNY	0,6561
A01	HLA-A*26:02	554	ESNKKFLPF	0,6456
A01	HLA-A*26:02	568	DIADTTDAV	0,5453
A01	HLA-A*26:02	583	EILDITPCSF	0,8274
A01	HLA-A*26:02	603	NTSNQVAVL	0,5594
A01	HLA-A*26:02	603	NTSNQVAVLY	0,7784
A01	HLA-A*26:02	686	SVASQSIIAY	0,8793
A01	HLA-A*26:02	691	SIIAYTMSL	0,7185
A01	HLA-A*26:02	710	NSIAIPTNF	0,7355
A01	HLA-A*26:02	718	FTISVTTEI	0,7944
A01	HLA-A*26:02	780	EVFAQVKQI	0,672
A01	HLA-A*26:02	780	EVFAQVKQIY	0,8897
A01	HLA-A*26:02	865	LTDEMIAQY	0,7697
A01	HLA-A*26:02	869	MIAQYTSAL	0,7428
A01	HLA-A*26:02	880	GTITSGWTF	0,5972
A01	HLA-A*26:02	886	WTFGAGAAL	0,7617
A01	HLA-A*26:02	898	FAMQMAYRF	0,5082
A01	HLA-A*26:02	940	STASALGKL	0,6677
A01	HLA-A*26:02	962	LVKQLSSNF	0,536
A01	HLA-A*26:02	1054	QSAPHGVVF	0,6027
A01	HLA-A*26:02	1095	FVSNGTHWF	0,8644
A01	HLA-A*26:02	1113	QIITTDNTF	0,6231
A01	HLA-A*26:02	1168	DISGINASV	0,5184

A01	HLA-A*26:02	1188	EVAKNLNESL	0,5001
A01	HLA-A*26:03	192	FVFKNIDGY	0,7319
A01	HLA-A*26:03	258	WTAGAAAYY	0,746
A01	HLA-A*26:04	28	YTNSFTRGVY	0,5132
A01	HLA-A*26:04	191	EFVFKNIDGY	0,6376
A01	HLA-A*26:04	192	FVFKNIDGY	0,853
A01	HLA-A*26:04	258	WTAGAAAYY	0,8788
A01	HLA-A*26:04	360	NCVADYSVLY	0,6391
A01	HLA-A*26:04	361	CVADYSVLY	0,75
A01	HLA-A*26:04	686	SVASQSIIAY	0,5849
A01	HLA-A*26:04	780	EVFAQVKQIY	0,6193
A01	HLA-A*26:05	28	YTNSFTRGVY	0,5354
A01	HLA-A*26:05	191	EFVFKNIDGY	0,7735
A01	HLA-A*26:05	192	FVFKNIDGY	0,8748
A01	HLA-A*26:05	258	WTAGAAAYY	0,9178
A01	HLA-A*26:05	340	EVFNATRFASVY	0,5592
A01	HLA-A*26:05	360	NCVADYSVLY	0,7308
A01	HLA-A*26:05	361	CVADYSVLY	0,7925
A01	HLA-A*26:05	603	NTSNQVAVLY	0,5637
A01	HLA-A*26:05	686	SVASQSIIAY	0,6565
A01	HLA-A*26:05	780	EVFAQVKQIY	0,7142
A01	HLA-A*26:06	192	FVFKNIDGY	0,7319
A01	HLA-A*26:06	258	WTAGAAAYY	0,746
A01	HLA-A*26:07	28	YTNSFTRGVY	0,5921
A01	HLA-A*26:07	50	STQDLFLPF	0,531
A01	HLA-A*26:07	192	FVFKNIDGY	0,929
A01	HLA-A*26:07	257	GWTAGAAAYY	0,6545
A01	HLA-A*26:07	258	WTAGAAAYY	0,8842
A01	HLA-A*26:07	261	GAAAYYVGY	0,7223
A01	HLA-A*26:07	360	NCVADYSVLY	0,557
A01	HLA-A*26:07	361	CVADYSVLY	0,7863
A01	HLA-A*26:07	366	SVLYNSASF	0,546
A01	HLA-A*26:07	686	SVASQSIIAY	0,7678
A01	HLA-A*26:07	691	SIIAYTMSL	0,5743
A01	HLA-A*26:07	698	SLGAENSVAY	0,5027
A01	HLA-A*26:07	718	FTISVTTEI	0,5245
A01	HLA-A*26:07	864	LLTDEMIAQY	0,554
A01	HLA-A*26:07	865	LTDEMIAQY	0,7389
A01	HLA-A*26:07	880	GTITSGWTF	0,6723
A01	HLA-A*26:07	976	VLNDILSRL	0,5945
A01	HLA-A*26:07	1095	FVSNGTHWF	0,7015
A01	HLA-A*26:07	1196	SLIDLQELGKY	0,516
A01	HLA-A*26:08	28	YTNSFTRGVY	0,5682
A01	HLA-A*26:08	50	STQDLFLPF	0,5601
A01	HLA-A*26:08	191	EFVFKNIDGY	0,6318
A01	HLA-A*26:08	192	FVFKNIDGY	0,8385
A01	HLA-A*26:08	258	WTAGAAAYY	0,9025
A01	HLA-A*26:08	340	EVFNATRFASVY	0,5328
A01	HLA-A*26:08	360	NCVADYSVLY	0,766
A01	HLA-A*26:08	361	CVADYSVLY	0,819

A01	HLA-A*26:08	603	NTSNQVAVLY	0,5863
A01	HLA-A*26:08	686	SVASQSIIAY	0,7121
A01	HLA-A*26:08	780	EVFAQVKQIY	0,596
A01	HLA-A*26:08	865	LTDEMIAQY	0,7329
A01	HLA-A*26:08	1095	FVSNGTHWF	0,5998
A01	HLA-A*26:09	28	YTNSFTRGVY	0,6479
A01	HLA-A*26:09	191	EFVFKNIDGY	0,751
A01	HLA-A*26:09	192	FVFKNIDGY	0,9239
A01	HLA-A*26:09	257	GWTAGAAAYY	0,5288
A01	HLA-A*26:09	258	WTAGAAAYY	0,9326
A01	HLA-A*26:09	298	ETKCTLKSF	0,5067
A01	HLA-A*26:09	340	EVFNATRFASVY	0,6007
A01	HLA-A*26:09	360	NCVADYSVLY	0,7668
A01	HLA-A*26:09	361	CVADYSVLY	0,8649
A01	HLA-A*26:09	603	NTSNQVAVLY	0,611
A01	HLA-A*26:09	686	SVASQSIIAY	0,7378
A01	HLA-A*26:09	780	EVFAQVKQIY	0,7512
A01	HLA-A*26:09	1095	FVSNGTHWF	0,6488
A01	HLA-A*26:10	28	YTNSFTRGVY	0,5472
A01	HLA-A*26:10	191	EFVFKNIDGY	0,8157
A01	HLA-A*26:10	192	FVFKNIDGY	0,9119
A01	HLA-A*26:10	258	WTAGAAAYY	0,9338
A01	HLA-A*26:10	340	EVFNATRFASVY	0,6393
A01	HLA-A*26:10	360	NCVADYSVLY	0,7886
A01	HLA-A*26:10	361	CVADYSVLY	0,8578
A01	HLA-A*26:10	603	NTSNQVAVLY	0,5793
A01	HLA-A*26:10	686	SVASQSIIAY	0,6831
A01	HLA-A*26:10	780	EVFAQVKQIY	0,7706
A01	HLA-A*26:10	1095	FVSNGTHWF	0,5675
A01	HLA-A*26:12	191	EFVFKNIDGY	0,6509
A01	HLA-A*26:12	192	FVFKNIDGY	0,8495
A01	HLA-A*26:12	258	WTAGAAAYY	0,8859
A01	HLA-A*26:12	360	NCVADYSVLY	0,7498
A01	HLA-A*26:12	361	CVADYSVLY	0,7826
A01	HLA-A*26:12	603	NTSNQVAVLY	0,5023
A01	HLA-A*26:12	686	SVASQSIIAY	0,5107
A01	HLA-A*26:12	780	EVFAQVKQIY	0,5545
A01	HLA-A*26:12	1095	FVSNGTHWF	0,5181
A01	HLA-A*26:13	28	YTNSFTRGVY	0,5142
A01	HLA-A*26:13	191	EFVFKNIDGY	0,7343
A01	HLA-A*26:13	192	FVFKNIDGY	0,8696
A01	HLA-A*26:13	258	WTAGAAAYY	0,9172
A01	HLA-A*26:13	340	EVFNATRFASVY	0,5729
A01	HLA-A*26:13	360	NCVADYSVLY	0,7292
A01	HLA-A*26:13	361	CVADYSVLY	0,794
A01	HLA-A*26:13	603	NTSNQVAVLY	0,561
A01	HLA-A*26:13	686	SVASQSIIAY	0,6258
A01	HLA-A*26:13	780	EVFAQVKQIY	0,7104
A01	HLA-A*26:13	886	WTFGAGAAL	0,5071
A01	HLA-A*26:13	1095	FVSNGTHWF	0,5038

A01	HLA-A*26:14	28	YTNSFTRGVY	0,5472
A01	HLA-A*26:14	191	EFVFKNIDGY	0,8157
A01	HLA-A*26:14	192	FVFKNIDGY	0,9119
A01	HLA-A*26:14	258	WTAGAAAYY	0,9338
A01	HLA-A*26:14	340	EVFNATRFASVY	0,6393
A01	HLA-A*26:14	360	NCVADYSVLY	0,7886
A01	HLA-A*26:14	361	CVADYSVLY	0,8578
A01	HLA-A*26:14	603	NTSNQVAVLY	0,5793
A01	HLA-A*26:14	686	SVASQSIIAY	0,6831
A01	HLA-A*26:14	780	EVFAQVKQIY	0,7706
A01	HLA-A*26:14	1095	FVSNGTHWF	0,5675
A01	HLA-A*26:15	28	YTNSFTRGVY	0,5472
A01	HLA-A*26:15	191	EFVFKNIDGY	0,8157
A01	HLA-A*26:15	192	FVFKNIDGY	0,9119
A01	HLA-A*26:15	258	WTAGAAAYY	0,9338
A01	HLA-A*26:15	340	EVFNATRFASVY	0,6393
A01	HLA-A*26:15	360	NCVADYSVLY	0,7886
A01	HLA-A*26:15	361	CVADYSVLY	0,8578
A01	HLA-A*26:15	603	NTSNQVAVLY	0,5793
A01	HLA-A*26:15	686	SVASQSIIAY	0,6831
A01	HLA-A*26:15	780	EVFAQVKQIY	0,7706
A01	HLA-A*26:15	1095	FVSNGTHWF	0,5675
A01	HLA-A*26:17	28	YTNSFTRGVY	0,5472
A01	HLA-A*26:17	191	EFVFKNIDGY	0,8157
A01	HLA-A*26:17	192	FVFKNIDGY	0,9119
A01	HLA-A*26:17	258	WTAGAAAYY	0,9338
A01	HLA-A*26:17	340	EVFNATRFASVY	0,6393
A01	HLA-A*26:17	360	NCVADYSVLY	0,7886
A01	HLA-A*26:17	361	CVADYSVLY	0,8578
A01	HLA-A*26:17	603	NTSNQVAVLY	0,5793
A01	HLA-A*26:17	686	SVASQSIIAY	0,6831
A01	HLA-A*26:17	780	EVFAQVKQIY	0,7706
A01	HLA-A*26:17	1095	FVSNGTHWF	0,5675
A01	HLA-A*26:18	191	EFVFKNIDGY	0,6509
A01	HLA-A*26:18	192	FVFKNIDGY	0,8495
A01	HLA-A*26:18	258	WTAGAAAYY	0,8859
A01	HLA-A*26:18	360	NCVADYSVLY	0,7498
A01	HLA-A*26:18	361	CVADYSVLY	0,7826
A01	HLA-A*26:18	603	NTSNQVAVLY	0,5023
A01	HLA-A*26:18	686	SVASQSIIAY	0,5107
A01	HLA-A*26:18	780	EVFAQVKQIY	0,5545
A01	HLA-A*26:18	1095	FVSNGTHWF	0,5181
A01	HLA-A*26:19	28	YTNSFTRGVY	0,7228
A01	HLA-A*26:19	28	YTNSFTRGVYY	0,5399
A01	HLA-A*26:19	30	NSFTRGVYY	0,7407
A01	HLA-A*26:19	50	STQDLFLPF	0,6401
A01	HLA-A*26:19	161	SSANNCTFEY	0,5341
A01	HLA-A*26:19	162	SANNCTFEY	0,5571
A01	HLA-A*26:19	192	FVFKNIDGY	0,8978
A01	HLA-A*26:19	256	SGWTAGAAAYY	0,6687

A01	HLA-A*26:19	257	GWTAGAAAYY	0,8485
A01	HLA-A*26:19	258	WTAGAAAYY	0,9258
A01	HLA-A*26:19	261	GAAAYYVGY	0,8482
A01	HLA-A*26:19	359	SNCVADYSVLY	0,5535
A01	HLA-A*26:19	360	NCVADYSVLY	0,613
A01	HLA-A*26:19	361	CVADYSVLY	0,8311
A01	HLA-A*26:19	366	SVLYNSASF	0,5818
A01	HLA-A*26:19	372	ASFSTFKCY	0,5748
A01	HLA-A*26:19	603	NTSNQVAVLY	0,5876
A01	HLA-A*26:19	604	TSNQVAVLY	0,8069
A01	HLA-A*26:19	634	RVYSTGSNVF	0,5845
A01	HLA-A*26:19	686	SVASQSIIAY	0,8445
A01	HLA-A*26:19	687	VASQSIIAY	0,7283
A01	HLA-A*26:19	691	SIIAYTMSL	0,5489
A01	HLA-A*26:19	733	KTSVDCTMY	0,5057
A01	HLA-A*26:19	865	LTDEMIAQY	0,7578
A01	HLA-A*26:19	880	GTITSGWTF	0,7351
A01	HLA-A*26:19	940	STASALGKL	0,5468
A01	HLA-A*26:19	1054	QSAPHGVVF	0,5969
A01	HLA-A*26:19	1059	GVVFLHVTY	0,6507
A01	HLA-A*26:19	1095	FVSNGTHWF	0,5261
A01	HLA-A*26:19	1128	VVIGIVNNTVY	0,5268
A01	HLA-A*26:21	192	FVFKNIDGY	0,7319
A01	HLA-A*26:21	258	WTAGAAAYY	0,746
A01	HLA-A*26:23	28	YTNSFTRGVY	0,5472
A01	HLA-A*26:23	191	EFVFKNIDGY	0,8157
A01	HLA-A*26:23	192	FVFKNIDGY	0,9119
A01	HLA-A*26:23	258	WTAGAAAYY	0,9338
A01	HLA-A*26:23	340	EVFNATRFASVY	0,6393
A01	HLA-A*26:23	360	NCVADYSVLY	0,7886
A01	HLA-A*26:23	361	CVADYSVLY	0,8578
A01	HLA-A*26:23	603	NTSNQVAVLY	0,5793
A01	HLA-A*26:23	686	SVASQSIIAY	0,6831
A01	HLA-A*26:23	780	EVFAQVKQIY	0,7706
A01	HLA-A*26:23	1095	FVSNGTHWF	0,5675
A01	HLA-A*26:24	28	YTNSFTRGVY	0,5472
A01	HLA-A*26:24	191	EFVFKNIDGY	0,8157
A01	HLA-A*26:24	192	FVFKNIDGY	0,9119
A01	HLA-A*26:24	258	WTAGAAAYY	0,9338
A01	HLA-A*26:24	340	EVFNATRFASVY	0,6393
A01	HLA-A*26:24	360	NCVADYSVLY	0,7886
A01	HLA-A*26:24	361	CVADYSVLY	0,8578
A01	HLA-A*26:24	603	NTSNQVAVLY	0,5793
A01	HLA-A*26:24	686	SVASQSIIAY	0,6831
A01	HLA-A*26:24	780	EVFAQVKQIY	0,7706
A01	HLA-A*26:24	1095	FVSNGTHWF	0,5675
A01	HLA-A*26:26	28	YTNSFTRGVY	0,5472
A01	HLA-A*26:26	191	EFVFKNIDGY	0,8157
A01	HLA-A*26:26	192	FVFKNIDGY	0,9119
A01	HLA-A*26:26	258	WTAGAAAYY	0,9338

A01	HLA-A*26:26	340	EVFNATRFASVY	0,6393
A01	HLA-A*26:26	360	NCVADYSVLY	0,7886
A01	HLA-A*26:26	361	CVADYSVLY	0,8578
A01	HLA-A*26:26	603	NTSNQVAVLY	0,5793
A01	HLA-A*26:26	686	SVASQSIIAY	0,6831
A01	HLA-A*26:26	780	EVFAQVKQIY	0,7706
A01	HLA-A*26:26	1095	FVSNGTHWF	0,5675
A01	HLA-A*30:02	28	YTNSFTRGVY	0,5478
A01	HLA-A*30:02	30	NSFTRGVYY	0,6841
A01	HLA-A*30:02	161	SSANNCTFEY	0,5282
A01	HLA-A*30:02	162	SANNCTFEY	0,5717
A01	HLA-A*30:02	192	FVFKNIDGY	0,5825
A01	HLA-A*30:02	195	KNIDGYFKIY	0,5462
A01	HLA-A*30:02	240	TLLALHRSY	0,5237
A01	HLA-A*30:02	257	GWTAGAAAYY	0,6876
A01	HLA-A*30:02	258	WTAGAAAYY	0,7568
A01	HLA-A*30:02	261	GAAAYYVGY	0,6441
A01	HLA-A*30:02	304	KSFTVEKGIY	0,5482
A01	HLA-A*30:02	357	RISNCVADY	0,5878
A01	HLA-A*30:02	361	CVADYSVLY	0,5824
A01	HLA-A*30:02	372	ASFSTFKCY	0,5884
A01	HLA-A*30:02	413	GQTGKIADY	0,581
A01	HLA-A*30:02	444	KVGGNYNYLY	0,7271
A01	HLA-A*30:02	445	VGGNYNYLY	0,5772
A01	HLA-A*30:02	496	GFQPTNGVGY	0,5657
A01	HLA-A*30:02	604	TSNQVAVLY	0,8094
A01	HLA-A*30:02	628	QLTPTWRVY	0,5588
A01	HLA-A*30:02	666	IGAGICASY	0,6063
A01	HLA-A*30:02	686	SVASQSIIAY	0,5074
A01	HLA-A*30:02	687	VASQSIIAY	0,6482
A01	HLA-A*30:02	733	KTSVDCTMY	0,6807
A01	HLA-A*30:02	781	VFAQVKQIY	0,6982
A01	HLA-A*30:02	865	LTDEMIAQY	0,69
A01	HLA-A*30:02	1039	RVDFCGKGY	0,6059
A01	HLA-A*30:02	1264	VLKGVKLHY	0,8117
A01	HLA-A*30:03	28	YTNSFTRGVY	0,5478
A01	HLA-A*30:03	30	NSFTRGVYY	0,6841
A01	HLA-A*30:03	161	SSANNCTFEY	0,5282
A01	HLA-A*30:03	162	SANNCTFEY	0,5717
A01	HLA-A*30:03	192	FVFKNIDGY	0,5825
A01	HLA-A*30:03	195	KNIDGYFKIY	0,5462
A01	HLA-A*30:03	240	TLLALHRSY	0,5237
A01	HLA-A*30:03	257	GWTAGAAAYY	0,6876
A01	HLA-A*30:03	258	WTAGAAAYY	0,7568
A01	HLA-A*30:03	261	GAAAYYVGY	0,6441
A01	HLA-A*30:03	304	KSFTVEKGIY	0,5482
A01	HLA-A*30:03	357	RISNCVADY	0,5878
A01	HLA-A*30:03	361	CVADYSVLY	0,5824
A01	HLA-A*30:03	372	ASFSTFKCY	0,5884
A01	HLA-A*30:03	413	GQTGKIADY	0,581

A01	HLA-A*30:03	444	KVGGNYNYLY	0,7271
A01	HLA-A*30:03	445	VGGNYNYLY	0,5772
A01	HLA-A*30:03	496	GFQPTNGVGY	0,5657
A01	HLA-A*30:03	604	TSNQVAVLY	0,8094
A01	HLA-A*30:03	628	QLTPTWRVY	0,5588
A01	HLA-A*30:03	666	IGAGICASY	0,6063
A01	HLA-A*30:03	686	SVASQSIIAY	0,5074
A01	HLA-A*30:03	687	VASQSIIAY	0,6482
A01	HLA-A*30:03	733	KTSVDCTMY	0,6807
A01	HLA-A*30:03	781	VFAQVKQIY	0,6982
A01	HLA-A*30:03	865	LTDEMIAQY	0,69
A01	HLA-A*30:03	1039	RVDFCGKGY	0,6059
A01	HLA-A*30:03	1264	VLKGVKLHY	0,8117
A01	HLA-A*30:04	257	GWTAGAAAYY	0,5598
A01	HLA-A*30:04	258	WTAGAAAYY	0,6721
A01	HLA-A*30:04	261	GAAAYYVGY	0,5257
A01	HLA-A*30:04	444	KVGGNYNYLY	0,5264
A01	HLA-A*30:04	445	VGGNYNYLY	0,512
A01	HLA-A*30:04	604	TSNQVAVLY	0,6629
A01	HLA-A*30:06	257	GWTAGAAAYY	0,5598
A01	HLA-A*30:06	258	WTAGAAAYY	0,6721
A01	HLA-A*30:06	261	GAAAYYVGY	0,5257
A01	HLA-A*30:06	444	KVGGNYNYLY	0,5264
A01	HLA-A*30:06	445	VGGNYNYLY	0,512
A01	HLA-A*30:06	604	TSNQVAVLY	0,6629
A01	HLA-A*30:09	30	NSFTRGVYY	0,5993
A01	HLA-A*30:09	162	SANNCTFEY	0,5586
A01	HLA-A*30:09	195	KNIDGYFKIY	0,5437
A01	HLA-A*30:09	257	GWTAGAAAYY	0,5637
A01	HLA-A*30:09	258	WTAGAAAYY	0,667
A01	HLA-A*30:09	261	GAAAYYVGY	0,6001
A01	HLA-A*30:09	361	CVADYSVLY	0,5285
A01	HLA-A*30:09	444	KVGGNYNYLY	0,6407
A01	HLA-A*30:09	445	VGGNYNYLY	0,6713
A01	HLA-A*30:09	604	TSNQVAVLY	0,7365
A01	HLA-A*30:09	666	IGAGICASY	0,5459
A01	HLA-A*30:09	733	KTSVDCTMY	0,5356
A01	HLA-A*30:09	781	VFAQVKQIY	0,6655
A01	HLA-A*30:09	865	LTDEMIAQY	0,6628
A01	HLA-A*30:12	28	YTNSFTRGVY	0,5478
A01	HLA-A*30:12	30	NSFTRGVYY	0,6841
A01	HLA-A*30:12	161	SSANNCTFEY	0,5282
A01	HLA-A*30:12	162	SANNCTFEY	0,5717
A01	HLA-A*30:12	192	FVFKNIDGY	0,5825
A01	HLA-A*30:12	195	KNIDGYFKIY	0,5462
A01	HLA-A*30:12	240	TLLALHRSY	0,5237
A01	HLA-A*30:12	257	GWTAGAAAYY	0,6876
A01	HLA-A*30:12	258	WTAGAAAYY	0,7568
A01	HLA-A*30:12	261	GAAAYYVGY	0,6441
A01	HLA-A*30:12	304	KSFTVEKGIY	0,5482

A01	HLA-A*30:12	357	RISNCVADY	0,5878
A01	HLA-A*30:12	361	CVADYSVLY	0,5824
A01	HLA-A*30:12	372	ASFSTFKCY	0,5884
A01	HLA-A*30:12	413	GQTGKIADY	0,581
A01	HLA-A*30:12	444	KVGGNYNYLY	0,7271
A01	HLA-A*30:12	445	VGGNYNYLY	0,5772
A01	HLA-A*30:12	496	GFQPTNGVGY	0,5657
A01	HLA-A*30:12	604	TSNQVAVLY	0,8094
A01	HLA-A*30:12	628	QLTPTWRVY	0,5588
A01	HLA-A*30:12	666	IGAGICASY	0,6063
A01	HLA-A*30:12	686	SVASQSIIAY	0,5074
A01	HLA-A*30:12	687	VASQSIIAY	0,6482
A01	HLA-A*30:12	733	KTSVDCTMY	0,6807
A01	HLA-A*30:12	781	VFAQVKQIY	0,6982
A01	HLA-A*30:12	865	LTDEMIAQY	0,69
A01	HLA-A*30:12	1039	RVDFCGKGY	0,6059
A01	HLA-A*30:12	1264	VLKGVKLHY	0,8117
A01	HLA-A*32:01	366	SVLYNSASF	0,6299
A01	HLA-A*32:01	417	KIADYNYKL	0,8445
A01	HLA-A*32:01	634	RVYSTGSNVF	0,6705
A01	HLA-A*32:01	691	SIIAYTMSL	0,7021
A01	HLA-A*32:01	815	RSFIEDLLF	0,5863
A01	HLA-A*32:01	880	GTITSGWTF	0,7522
A01	HLA-A*32:01	1185	RLNEVAKNL	0,7116
A01	HLA-A*32:02	366	SVLYNSASF	0,5561
A01	HLA-A*32:02	417	KIADYNYKL	0,7698
A01	HLA-A*32:02	634	RVYSTGSNVF	0,5403
A01	HLA-A*32:02	691	SIIAYTMSL	0,62
A01	HLA-A*32:02	880	GTITSGWTF	0,6422
A01	HLA-A*32:02	1185	RLNEVAKNL	0,6529
A01	HLA-A*32:05	366	SVLYNSASF	0,6066
A01	HLA-A*32:05	417	KIADYNYKL	0,7528
A01	HLA-A*32:05	634	RVYSTGSNVF	0,5466
A01	HLA-A*32:05	691	SIIAYTMSL	0,6692
A01	HLA-A*32:05	815	RSFIEDLLF	0,5277
A01	HLA-A*32:05	880	GTITSGWTF	0,7114
A01	HLA-A*32:05	1185	RLNEVAKNL	0,5942
A01	HLA-A*32:06	366	SVLYNSASF	0,6299
A01	HLA-A*32:06	417	KIADYNYKL	0,8445
A01	HLA-A*32:06	634	RVYSTGSNVF	0,6705
A01	HLA-A*32:06	691	SIIAYTMSL	0,7021
A01	HLA-A*32:06	815	RSFIEDLLF	0,5863
A01	HLA-A*32:06	880	GTITSGWTF	0,7522
A01	HLA-A*32:06	1185	RLNEVAKNL	0,7116
A01	HLA-A*32:07	50	STQDLFLPF	0,5832
A01	HLA-A*32:07	77	KRFDNPVLPF	0,6519
A01	HLA-A*32:07	78	RFDNPVLPF	0,7048
A01	HLA-A*32:07	144	YYHKNNKSW	0,6197
A01	HLA-A*32:07	193	VFKNIDGYF	0,6354
A01	HLA-A*32:07	195	KNIDGYFKI	0,501

A01	HLA-A*32:07	261	GAAAYYVGY	0,5142
A01	HLA-A*32:07	267	VGYLQPRTF	0,5924
A01	HLA-A*32:07	268	GYLQPRTFL	0,5759
A01	HLA-A*32:07	310	KGIYQTSNF	0,5492
A01	HLA-A*32:07	345	TRFASVYAW	0,7472
A01	HLA-A*32:07	366	SVLYNSASF	0,7088
A01	HLA-A*32:07	417	KIADYNYKL	0,6173
A01	HLA-A*32:07	557	KKFLPFQQF	0,6339
A01	HLA-A*32:07	634	RVYSTGSNVF	0,6399
A01	HLA-A*32:07	635	VYSTGSNVF	0,566
A01	HLA-A*32:07	689	SQSIIAYTM	0,5018
A01	HLA-A*32:07	710	NSIAIPTNF	0,6477
A01	HLA-A*32:07	781	VFAQVKQIY	0,715
A01	HLA-A*32:07	815	RSFIEDLLF	0,684
A01	HLA-A*32:07	880	GTITSGWTF	0,8141
A01	HLA-A*32:07	1054	QSAPHGVVF	0,6406
A01	HLA-A*32:07	1101	HWFVTQRNF	0,7546
A01	HLA-A*32:07	1208	QYIKWPWYI	0,6932
A01	HLA-A*32:09	50	STQDLFLPF	0,5147
A01	HLA-A*32:09	366	SVLYNSASF	0,6078
A01	HLA-A*32:09	417	KIADYNYKL	0,6951
A01	HLA-A*32:09	691	SIIAYTMSL	0,552
A01	HLA-A*32:09	815	RSFIEDLLF	0,5187
A01	HLA-A*32:09	880	GTITSGWTF	0,7219
A01	HLA-A*32:10	634	RVYSTGSNVF	0,5972
A01	HLA-A*36:01	28	YTNSFTRGVY	0,7119
A01	HLA-A*36:01	28	YTNSFTRGVYY	0,7867
A01	HLA-A*36:01	160	YSSANNCTFEY	0,5391
A01	HLA-A*36:01	161	SSANNCTFEY	0,7073
A01	HLA-A*36:01	162	SANNCTFEY	0,6682
A01	HLA-A*36:01	256	SGWTAGAAAYY	0,667
A01	HLA-A*36:01	257	GWTAGAAAYY	0,7231
A01	HLA-A*36:01	258	WTAGAAAYY	0,8964
A01	HLA-A*36:01	358	ISNCVADYSVLY	0,6026
A01	HLA-A*36:01	361	CVADYSVLY	0,7473
A01	HLA-A*36:01	362	VADYSVLY	0,65
A01	HLA-A*36:01	414	QTGKIADYNY	0,5523
A01	HLA-A*36:01	601	GTNTSNQVAVLY	0,629
A01	HLA-A*36:01	603	NTSNQVAVLY	0,7051
A01	HLA-A*36:01	604	TSNQVAVLY	0,8841
A01	HLA-A*36:01	733	KTSVDCTMY	0,7042
A01	HLA-A*36:01	746	STECSNLLLQY	0,6614
A01	HLA-A*36:01	828	LADAGFIKQY	0,7384
A01	HLA-A*36:01	863	PLLTDEMIAQY	0,5018
A01	HLA-A*36:01	865	LTDEMIAQY	0,9845
A01	HLA-A*36:01	1039	RVDFCGKGY	0,8092
A01	HLA-A*36:01	1197	LIDLQELGKY	0,7101
A01	HLA-A*36:02	28	YTNSFTRGVY	0,5893
A01	HLA-A*36:02	28	YTNSFTRGVYY	0,6541
A01	HLA-A*36:02	161	SSANNCTFEY	0,6078

A01	HLA-A*36:02	162	SANNCTFEY	0,605
A01	HLA-A*36:02	256	SGWTAGAAAYY	0,583
A01	HLA-A*36:02	257	GWTAGAAAYY	0,6743
A01	HLA-A*36:02	258	WTAGAAAYY	0,8378
A01	HLA-A*36:02	361	CVADYSVLY	0,6943
A01	HLA-A*36:02	444	KVGGNYNYLY	0,5293
A01	HLA-A*36:02	601	GTNTSNQVAVLY	0,5171
A01	HLA-A*36:02	603	NTSNQVAVLY	0,5926
A01	HLA-A*36:02	604	TSNQVAVLY	0,8154
A01	HLA-A*36:02	733	KTSVDCTMY	0,6327
A01	HLA-A*36:02	865	LTDEMIAQY	0,95
A01	HLA-A*36:02	1039	RVDFCGKGY	0,7404
A01	HLA-A*36:02	1197	LIDLQELGKY	0,5242
A01	HLA-A*36:04	28	YTNSFTRGVY	0,7271
A01	HLA-A*36:04	28	YTNSFTRGVYY	0,8143
A01	HLA-A*36:04	30	NSFTRGVYY	0,6993
A01	HLA-A*36:04	160	YSSANNCTFEY	0,6118
A01	HLA-A*36:04	161	SSANNCTFEY	0,727
A01	HLA-A*36:04	162	SANNCTFEY	0,6738
A01	HLA-A*36:04	256	SGWTAGAAAYY	0,709
A01	HLA-A*36:04	257	GWTAGAAAYY	0,7396
A01	HLA-A*36:04	258	WTAGAAAYY	0,9128
A01	HLA-A*36:04	358	ISNCVADYSVLY	0,652
A01	HLA-A*36:04	361	CVADYSVLY	0,7656
A01	HLA-A*36:04	362	VADYSVLY	0,6977
A01	HLA-A*36:04	414	QTGKIADYNY	0,5768
A01	HLA-A*36:04	601	GTNTSNQVAVLY	0,6316
A01	HLA-A*36:04	603	NTSNQVAVLY	0,7383
A01	HLA-A*36:04	604	TSNQVAVLY	0,9007
A01	HLA-A*36:04	733	KTSVDCTMY	0,64
A01	HLA-A*36:04	746	STECSNLLLQY	0,7554
A01	HLA-A*36:04	828	LADAGFIKQY	0,7998
A01	HLA-A*36:04	863	PLLTDEMIAQY	0,6402
A01	HLA-A*36:04	865	LTDEMIAQY	0,9936
A01	HLA-A*36:04	1039	RVDFCGKGY	0,7674
A01	HLA-A*36:04	1197	LIDLQELGKY	0,7682
A01 A03	HLA-A*30:01	89	GVYFASTEK	0,524
A01 A03	HLA-A*30:01	142	GVYYHKNNK	0,5185
A01 A03	HLA-A*30:01	302	TLKSFTVEK	0,5401
A01 A03	HLA-A*30:01	344	ATRFASVYA	0,8043
A01 A03	HLA-A*30:01	378	KCYGVSPTK	0,5092
A01 A03	HLA-A*30:01	454	RLFRKSNLK	0,5644
A01 A03	HLA-A*30:01	634	RVYSTGSNV	0,632
A01 A03	HLA-A*30:01	1065	VTYVPAQEK	0,5221
A01 A03	HLA-A*30:08	344	ATRFASVYA	0,6186
A01 A03	HLA-A*30:11	89	GVYFASTEK	0,524
A01 A03	HLA-A*30:11	142	GVYYHKNNK	0,5185
A01 A03	HLA-A*30:11	302	TLKSFTVEK	0,5401
A01 A03	HLA-A*30:11	344	ATRFASVYA	0,8043
A01 A03	HLA-A*30:11	378	KCYGVSPTK	0,5092

A01 A03	HLA-A*30:11	454	RLFRKSNLK	0,5644
A01 A03	HLA-A*30:11	634	RVYSTGSNV	0,632
A01 A03	HLA-A*30:11	1065	VTYVPAQEK	0,5221
A01 A03	HLA-A*30:15	89	GVYFASTEK	0,524
A01 A03	HLA-A*30:15	142	GVYYHKNNK	0,5185
A01 A03	HLA-A*30:15	302	TLKSFTVEK	0,5401
A01 A03	HLA-A*30:15	344	ATRFASVYA	0,8043
A01 A03	HLA-A*30:15	378	KCYGVSPTK	0,5092
A01 A03	HLA-A*30:15	454	RLFRKSNLK	0,5644
A01 A03	HLA-A*30:15	634	RVYSTGSNV	0,632
A01 A03	HLA-A*30:15	1065	VTYVPAQEK	0,5221
A01 A24	HLA-A*29:01	30	NSFTRGVYY	0,825
A01 A24	HLA-A*29:01	151	SWMESEFRVY	0,7114
A01 A24	HLA-A*29:01	162	SANNCTFEY	0,7814
A01 A24	HLA-A*29:01	192	FVFKNIDGY	0,9181
A01 A24	HLA-A*29:01	240	TLLALHRSY	0,6806
A01 A24	HLA-A*29:01	257	GWTAGAAAY	0,5826
A01 A24	HLA-A*29:01	257	GWTAGAAAYY	0,6803
A01 A24	HLA-A*29:01	258	WTAGAAAYY	0,9403
A01 A24	HLA-A*29:01	261	GAAAYYVGY	0,7714
A01 A24	HLA-A*29:01	360	NCVADYSVLY	0,6347
A01 A24	HLA-A*29:01	361	CVADYSVLY	0,9086
A01 A24	HLA-A*29:01	444	KVGGNYNYLY	0,5931
A01 A24	HLA-A*29:01	445	VGGNYNYLY	0,6436
A01 A24	HLA-A*29:01	496	GFQPTNGVGY	0,6858
A01 A24	HLA-A*29:01	604	TSNQVAVLY	0,8854
A01 A24	HLA-A*29:01	628	QLTPTWRVY	0,51
A01 A24	HLA-A*29:01	686	SVASQSIIAY	0,6064
A01 A24	HLA-A*29:01	687	VASQSIIAY	0,6791
A01 A24	HLA-A*29:01	781	VFAQVKQIY	0,9197
A01 A24	HLA-A*29:01	827	TLADAGFIKQY	0,5983
A01 A24	HLA-A*29:01	865	LTDEMIAQY	0,819
A01 A24	HLA-A*29:01	896	IPFAMQMAY	0,5343
A01 A24	HLA-A*29:01	909	IGVTQNVLY	0,5165
A01 A24	HLA-A*29:01	1059	GVVFLHVTY	0,7218
A01 A24	HLA-A*29:01	1102	WFVTQRNFY	0,7439
A01 A24	HLA-A*29:01	1147	SFKEELDKY	0,6923
A01 A24	HLA-A*29:01	1264	VLKGVKLHY	0,8598
A01 A24	HLA-A*29:02	30	NSFTRGVYY	0,825
A01 A24	HLA-A*29:02	151	SWMESEFRVY	0,7114
A01 A24	HLA-A*29:02	162	SANNCTFEY	0,7814
A01 A24	HLA-A*29:02	192	FVFKNIDGY	0,9181
A01 A24	HLA-A*29:02	240	TLLALHRSY	0,6806
A01 A24	HLA-A*29:02	257	GWTAGAAAY	0,5826
A01 A24	HLA-A*29:02	257	GWTAGAAAYY	0,6803
A01 A24	HLA-A*29:02	258	WTAGAAAYY	0,9403
A01 A24	HLA-A*29:02	261	GAAAYYVGY	0,7714
A01 A24	HLA-A*29:02	360	NCVADYSVLY	0,6347
A01 A24	HLA-A*29:02	361	CVADYSVLY	0,9086
A01 A24	HLA-A*29:02	444	KVGGNYNYLY	0,5931

A01 A24	HLA-A*29:02	445	VGGNYNYLY	0,6436
A01 A24	HLA-A*29:02	496	GFQPTNGVGY	0,6858
A01 A24	HLA-A*29:02	604	TSNQVAVLY	0,8854
A01 A24	HLA-A*29:02	628	QLTPTWRVY	0,51
A01 A24	HLA-A*29:02	686	SVASQSIIAY	0,6064
A01 A24	HLA-A*29:02	687	VASQSIIAY	0,6791
A01 A24	HLA-A*29:02	781	VFAQVKQIY	0,9197
A01 A24	HLA-A*29:02	827	TLADAGFIKQY	0,5983
A01 A24	HLA-A*29:02	865	LTDEMIAQY	0,819
A01 A24	HLA-A*29:02	896	IPFAMQMAY	0,5343
A01 A24	HLA-A*29:02	909	IGVTQNVLY	0,5165
A01 A24	HLA-A*29:02	1059	GVVFLHVTY	0,7218
A01 A24	HLA-A*29:02	1102	WFVTQRNFY	0,7439
A01 A24	HLA-A*29:02	1147	SFKEELDKY	0,6923
A01 A24	HLA-A*29:02	1264	VLKGVKLHY	0,8598
A01 A24	HLA-A*29:03	30	NSFTRGVYY	0,7608
A01 A24	HLA-A*29:03	151	SWMESEFRVY	0,5544
A01 A24	HLA-A*29:03	152	WMESEFRVY	0,5108
A01 A24	HLA-A*29:03	162	SANNCTFEY	0,6647
A01 A24	HLA-A*29:03	192	FVFKNIDGY	0,814
A01 A24	HLA-A*29:03	240	TLLALHRSY	0,5915
A01 A24	HLA-A*29:03	258	WTAGAAAYY	0,863
A01 A24	HLA-A*29:03	261	GAAAYYVGY	0,648
A01 A24	HLA-A*29:03	360	NCVADYSVLY	0,6374
A01 A24	HLA-A*29:03	361	CVADYSVLY	0,8638
A01 A24	HLA-A*29:03	445	VGGNYNYLY	0,5077
A01 A24	HLA-A*29:03	603	NTSNQVAVLY	0,5268
A01 A24	HLA-A*29:03	604	TSNQVAVLY	0,8418
A01 A24	HLA-A*29:03	781	VFAQVKQIY	0,8384
A01 A24	HLA-A*29:03	865	LTDEMIAQY	0,7807
A01 A24	HLA-A*29:03	1102	WFVTQRNFY	0,612
A01 A24	HLA-A*29:05	30	NSFTRGVYY	0,6683
A01 A24	HLA-A*29:05	192	FVFKNIDGY	0,6624
A01 A24	HLA-A*29:05	240	TLLALHRSY	0,5807
A01 A24	HLA-A*29:05	257	GWTAGAAAYY	0,595
A01 A24	HLA-A*29:05	258	WTAGAAAYY	0,7752
A01 A24	HLA-A*29:05	361	CVADYSVLY	0,6495
A01 A24	HLA-A*29:05	496	GFQPTNGVGY	0,6148
A01 A24	HLA-A*29:05	604	TSNQVAVLY	0,6551
A01 A24	HLA-A*29:05	686	SVASQSIIAY	0,547
A01 A24	HLA-A*29:05	781	VFAQVKQIY	0,7974
A01 A24	HLA-A*29:05	1102	WFVTQRNFY	0,5904
A01 A24	HLA-A*29:05	1264	VLKGVKLHY	0,8571
A01 A24	HLA-A*29:06	30	NSFTRGVYY	0,825
A01 A24	HLA-A*29:06	151	SWMESEFRVY	0,7114
A01 A24	HLA-A*29:06	162	SANNCTFEY	0,7814
A01 A24	HLA-A*29:06	192	FVFKNIDGY	0,9181
A01 A24	HLA-A*29:06	240	TLLALHRSY	0,6806
A01 A24	HLA-A*29:06	257	GWTAGAAAY	0,5826
A01 A24	HLA-A*29:06	257	GWTAGAAAYY	0,6803

A01 A24	HLA-A*29:06	258	WTAGAAAYY	0,9403
A01 A24	HLA-A*29:06	261	GAAAYYVGY	0,7714
A01 A24	HLA-A*29:06	360	NCVADYSVLY	0,6347
A01 A24	HLA-A*29:06	361	CVADYSVLY	0,9086
A01 A24	HLA-A*29:06	444	KVGGNYNYLY	0,5931
A01 A24	HLA-A*29:06	445	VGGNYNYLY	0,6436
A01 A24	HLA-A*29:06	496	GFQPTNGVGY	0,6858
A01 A24	HLA-A*29:06	604	TSNQVAVLY	0,8854
A01 A24	HLA-A*29:06	628	QLTPTWRVY	0,51
A01 A24	HLA-A*29:06	686	SVASQSIIAY	0,6064
A01 A24	HLA-A*29:06	687	VASQSIIAY	0,6791
A01 A24	HLA-A*29:06	781	VFAQVKQIY	0,9197
A01 A24	HLA-A*29:06	827	TLADAGFIKQY	0,5983
A01 A24	HLA-A*29:06	865	LTDEMIAQY	0,819
A01 A24	HLA-A*29:06	896	IPFAMQMAY	0,5343
A01 A24	HLA-A*29:06	909	IGVTQNVLY	0,5165
A01 A24	HLA-A*29:06	1059	GVVFLHVTY	0,7218
A01 A24	HLA-A*29:06	1102	WFVTQRNFY	0,7439
A01 A24	HLA-A*29:06	1147	SFKEELDKY	0,6923
A01 A24	HLA-A*29:06	1264	VLKGVKLHY	0,8598
A01 A24	HLA-A*29:09	30	NSFTRGVYY	0,825
A01 A24	HLA-A*29:09	151	SWMESEFRVY	0,7114
A01 A24	HLA-A*29:09	162	SANNCTFEY	0,7814
A01 A24	HLA-A*29:09	192	FVFKNIDGY	0,9181
A01 A24	HLA-A*29:09	240	TLLALHRSY	0,6806
A01 A24	HLA-A*29:09	257	GWTAGAAAY	0,5826
A01 A24	HLA-A*29:09	257	GWTAGAAAYY	0,6803
A01 A24	HLA-A*29:09	258	WTAGAAAYY	0,9403
A01 A24	HLA-A*29:09	261	GAAAYYVGY	0,7714
A01 A24	HLA-A*29:09	360	NCVADYSVLY	0,6347
A01 A24	HLA-A*29:09	361	CVADYSVLY	0,9086
A01 A24	HLA-A*29:09	444	KVGGNYNYLY	0,5931
A01 A24	HLA-A*29:09	445	VGGNYNYLY	0,6436
A01 A24	HLA-A*29:09	496	GFQPTNGVGY	0,6858
A01 A24	HLA-A*29:09	604	TSNQVAVLY	0,8854
A01 A24	HLA-A*29:09	628	QLTPTWRVY	0,51
A01 A24	HLA-A*29:09	686	SVASQSIIAY	0,6064
A01 A24	HLA-A*29:09	687	VASQSIIAY	0,6791
A01 A24	HLA-A*29:09	781	VFAQVKQIY	0,9197
A01 A24	HLA-A*29:09	827	TLADAGFIKQY	0,5983
A01 A24	HLA-A*29:09	865	LTDEMIAQY	0,819
A01 A24	HLA-A*29:09	896	IPFAMQMAY	0,5343
A01 A24	HLA-A*29:09	909	IGVTQNVLY	0,5165
A01 A24	HLA-A*29:09	1059	GVVFLHVTY	0,7218
A01 A24	HLA-A*29:09	1102	WFVTQRNFY	0,7439
A01 A24	HLA-A*29:09	1147	SFKEELDKY	0,6923
A01 A24	HLA-A*29:09	1264	VLKGVKLHY	0,8598
A01 A24	HLA-A*29:10	30	NSFTRGVYY	0,825
A01 A24	HLA-A*29:10	151	SWMESEFRVY	0,7114
A01 A24	HLA-A*29:10	162	SANNCTFEY	0,7814

A01 A24	HLA-A*29:10	192	FVFKNIDGY	0,9181
A01 A24	HLA-A*29:10	240	TLLALHRSY	0,6806
A01 A24	HLA-A*29:10	257	GWTAGAAAY	0,5826
A01 A24	HLA-A*29:10	257	GWTAGAAAYY	0,6803
A01 A24	HLA-A*29:10	258	WTAGAAAYY	0,9403
A01 A24	HLA-A*29:10	261	GAAAYYVGY	0,7714
A01 A24	HLA-A*29:10	360	NCVADYSVLY	0,6347
A01 A24	HLA-A*29:10	361	CVADYSVLY	0,9086
A01 A24	HLA-A*29:10	444	KVGGNYNYLY	0,5931
A01 A24	HLA-A*29:10	445	VGGNYNYLY	0,6436
A01 A24	HLA-A*29:10	496	GFQPTNGVGY	0,6858
A01 A24	HLA-A*29:10	604	TSNQVAVLY	0,8854
A01 A24	HLA-A*29:10	628	QLTPTWRVY	0,51
A01 A24	HLA-A*29:10	686	SVASQSIIAY	0,6064
A01 A24	HLA-A*29:10	687	VASQSIIAY	0,6791
A01 A24	HLA-A*29:10	781	VFAQVKQIY	0,9197
A01 A24	HLA-A*29:10	827	TLADAGFIKQY	0,5983
A01 A24	HLA-A*29:10	865	LTDEMIAQY	0,819
A01 A24	HLA-A*29:10	896	IPFAMQMAY	0,5343
A01 A24	HLA-A*29:10	909	IGVTQNVLY	0,5165
A01 A24	HLA-A*29:10	1059	GVVFLHVTY	0,7218
A01 A24	HLA-A*29:10	1102	WFVTQRNFY	0,7439
A01 A24	HLA-A*29:10	1147	SFKEELDKY	0,6923
A01 A24	HLA-A*29:10	1264	VLKGVKLHY	0,8598
A01 A24	HLA-A*29:11	30	NSFTRGVYY	0,825
A01 A24	HLA-A*29:11	151	SWMESEFRVY	0,7114
A01 A24	HLA-A*29:11	162	SANNCTFEY	0,7814
A01 A24	HLA-A*29:11	192	FVFKNIDGY	0,9181
A01 A24	HLA-A*29:11	240	TLLALHRSY	0,6806
A01 A24	HLA-A*29:11	257	GWTAGAAAY	0,5826
A01 A24	HLA-A*29:11	257	GWTAGAAAYY	0,6803
A01 A24	HLA-A*29:11	258	WTAGAAAYY	0,9403
A01 A24	HLA-A*29:11	261	GAAAYYVGY	0,7714
A01 A24	HLA-A*29:11	360	NCVADYSVLY	0,6347
A01 A24	HLA-A*29:11	361	CVADYSVLY	0,9086
A01 A24	HLA-A*29:11	444	KVGGNYNYLY	0,5931
A01 A24	HLA-A*29:11	445	VGGNYNYLY	0,6436
A01 A24	HLA-A*29:11	496	GFQPTNGVGY	0,6858
A01 A24	HLA-A*29:11	604	TSNQVAVLY	0,8854
A01 A24	HLA-A*29:11	628	QLTPTWRVY	0,51
A01 A24	HLA-A*29:11	686	SVASQSIIAY	0,6064
A01 A24	HLA-A*29:11	687	VASQSIIAY	0,6791
A01 A24	HLA-A*29:11	781	VFAQVKQIY	0,9197
A01 A24	HLA-A*29:11	827	TLADAGFIKQY	0,5983
A01 A24	HLA-A*29:11	865	LTDEMIAQY	0,819
A01 A24	HLA-A*29:11	896	IPFAMQMAY	0,5343
A01 A24	HLA-A*29:11	909	IGVTQNVLY	0,5165
A01 A24	HLA-A*29:11	1059	GVVFLHVTY	0,7218
A01 A24	HLA-A*29:11	1102	WFVTQRNFY	0,7439
A01 A24	HLA-A*29:11	1147	SFKEELDKY	0,6923

A01 A24	HLA-A*29:11	1264	VLKGVKLHY	0,8598
A01 A24	HLA-A*29:12	30	NSFTRGVYY	0,825
A01 A24	HLA-A*29:12	151	SWMESEFRVY	0,7114
A01 A24	HLA-A*29:12	162	SANNCTFEY	0,7814
A01 A24	HLA-A*29:12	192	FVFKNIDGY	0,9181
A01 A24	HLA-A*29:12	240	TLLALHRSY	0,6806
A01 A24	HLA-A*29:12	257	GWTAGAAAY	0,5826
A01 A24	HLA-A*29:12	257	GWTAGAAAYY	0,6803
A01 A24	HLA-A*29:12	258	WTAGAAAYY	0,9403
A01 A24	HLA-A*29:12	261	GAAAYYVGY	0,7714
A01 A24	HLA-A*29:12	360	NCVADYSVLY	0,6347
A01 A24	HLA-A*29:12	361	CVADYSVLY	0,9086
A01 A24	HLA-A*29:12	444	KVGGNYNYLY	0,5931
A01 A24	HLA-A*29:12	445	VGGNYNYLY	0,6436
A01 A24	HLA-A*29:12	496	GFQPTNGVGY	0,6858
A01 A24	HLA-A*29:12	604	TSNQVAVLY	0,8854
A01 A24	HLA-A*29:12	628	QLTPTWRVY	0,51
A01 A24	HLA-A*29:12	686	SVASQSIIAY	0,6064
A01 A24	HLA-A*29:12	687	VASQSIIAY	0,6791
A01 A24	HLA-A*29:12	781	VFAQVKQIY	0,9197
A01 A24	HLA-A*29:12	827	TLADAGFIKQY	0,5983
A01 A24	HLA-A*29:12	865	LTDEMIAQY	0,819
A01 A24	HLA-A*29:12	896	IPFAMQMAY	0,5343
A01 A24	HLA-A*29:12	909	IGVTQNVLY	0,5165
A01 A24	HLA-A*29:12	1059	GVVFLHVTY	0,7218
A01 A24	HLA-A*29:12	1102	WFVTQRNFY	0,7439
A01 A24	HLA-A*29:12	1147	SFKEELDKY	0,6923
A01 A24	HLA-A*29:12	1264	VLKGVKLHY	0,8598
A02	HLA-A*02:01	109	TLDSKTQSL	0,6782
A02	HLA-A*02:01	133	FQFCNDPFL	0,518
A02	HLA-A*02:01	269	YLQPRTFLL	0,973
A02	HLA-A*02:01	386	KLNDLCFTNV	0,7108
A02	HLA-A*02:01	417	KIADYNYKL	0,909
A02	HLA-A*02:01	424	KLPDDFTGCV	0,5916
A02	HLA-A*02:01	515	FELLHAPATV	0,6033
A02	HLA-A*02:01	610	VLYQDVNCTEV	0,5092
A02	HLA-A*02:01	691	SIIAYTMSL	0,7995
A02	HLA-A*02:01	718	FTISVTTEI	0,5257
A02	HLA-A*02:01	821	LLFNKVTLA	0,7857
A02	HLA-A*02:01	857	GLTVLPPLL	0,6786
A02	HLA-A*02:01	915	VLYENQKLI	0,5378
A02	HLA-A*02:01	975	SVLNDILSRL	0,5124
A02	HLA-A*02:01	976	VLNDILSRL	0,9507
A02	HLA-A*02:01	983	RLDKVEAEV	0,8609
A02	HLA-A*02:01	1000	RLQSLQTYV	0,7431
A02	HLA-A*02:01	1047	YHLMSFPQSA	0,5452
A02	HLA-A*02:01	1048	HLMSFPQSA	0,7385
A02	HLA-A*02:01	1060	VVFLHVTYV	0,6948
A02	HLA-A*02:01	1185	RLNEVAKNL	0,6189
A02	HLA-A*02:01	1192	NLNESLIDL	0,6972

A02	HLA-A*02:01	1220	FIAGLIAIV	0,8207
A02	HLA-A*02:02	109	TLDSKTQSL	0,825
A02	HLA-A*02:02	133	FQFCNDPFL	0,5968
A02	HLA-A*02:02	269	YLQPRTFLL	0,9773
A02	HLA-A*02:02	386	KLNDLCFTNV	0,8261
A02	HLA-A*02:02	416	GKIADYNYKL	0,6474
A02	HLA-A*02:02	417	KIADYNYKL	0,9705
A02	HLA-A*02:02	424	KLPDDFTGCV	0,8161
A02	HLA-A*02:02	512	VLSFELLHA	0,5802
A02	HLA-A*02:02	515	FELLHAPATV	0,5863
A02	HLA-A*02:02	691	SIIAYTMSL	0,8402
A02	HLA-A*02:02	718	FTISVTTEI	0,6992
A02	HLA-A*02:02	821	LLFNKVTLA	0,8324
A02	HLA-A*02:02	857	GLTVLPPLL	0,7823
A02	HLA-A*02:02	869	MIAQYTSAL	0,7746
A02	HLA-A*02:02	915	VLYENQKLI	0,6825
A02	HLA-A*02:02	937	SLSSTASAL	0,6944
A02	HLA-A*02:02	947	KLQDVVNQNA	0,5801
A02	HLA-A*02:02	958	ALNTLVKQL	0,8332
A02	HLA-A*02:02	975	SVLNDILSRL	0,878
A02	HLA-A*02:02	976	VLNDILSRL	0,989
A02	HLA-A*02:02	983	RLDKVEAEV	0,8636
A02	HLA-A*02:02	1000	RLQSLQTYV	0,8614
A02	HLA-A*02:02	1047	YHLMSFPQSA	0,6493
A02	HLA-A*02:02	1048	HLMSFPQSA	0,8395
A02	HLA-A*02:02	1060	VVFLHVTYV	0,6976
A02	HLA-A*02:02	1095	FVSNGTHWFV	0,621
A02	HLA-A*02:02	1185	RLNEVAKNL	0,9052
A02	HLA-A*02:02	1192	NLNESLIDL	0,8555
A02	HLA-A*02:02	1196	SLIDLQEL	0,7114
A02	HLA-A*02:02	1220	FIAGLIAIV	0,8951
A02	HLA-A*02:03	28	YTNSFTRGV	0,6379
A02	HLA-A*02:03	109	TLDSKTQSL	0,7157
A02	HLA-A*02:03	117	LLIVNNATNV	0,6411
A02	HLA-A*02:03	118	LIVNNATNV	0,6321
A02	HLA-A*02:03	119	IVNNATNVV	0,611
A02	HLA-A*02:03	202	KIYSKHTPI	0,6197
A02	HLA-A*02:03	241	LLALHRSYL	0,6696
A02	HLA-A*02:03	269	YLQPRTFLL	0,9474
A02	HLA-A*02:03	386	KLNDLCFTNV	0,8966
A02	HLA-A*02:03	417	KIADYNYKL	0,8266
A02	HLA-A*02:03	424	KLPDDFTGCV	0,8342
A02	HLA-A*02:03	512	VLSFELLHA	0,6927
A02	HLA-A*02:03	515	FELLHAPATV	0,75
A02	HLA-A*02:03	516	ELLHAPATV	0,6201
A02	HLA-A*02:03	610	VLYQDVNCTEV	0,6785
A02	HLA-A*02:03	634	RVYSTGSNV	0,7597
A02	HLA-A*02:03	691	SIIAYTMSL	0,8766
A02	HLA-A*02:03	718	FTISVTTEI	0,7603
A02	HLA-A*02:03	762	QLNRALTGI	0,7873

A02	HLA-A*02:03	821	LLFNKVTLA	0,9477
A02	HLA-A*02:03	869	MIAQYTSAL	0,7884
A02	HLA-A*02:03	915	VLYENQKLI	0,8141
A02	HLA-A*02:03	937	SLSSTASAL	0,7506
A02	HLA-A*02:03	947	KLQDVVNQNA	0,6818
A02	HLA-A*02:03	958	ALNTLVKQL	0,8303
A02	HLA-A*02:03	975	SVLNDILSRL	0,8775
A02	HLA-A*02:03	976	VLNDILSRL	0,986
A02	HLA-A*02:03	983	RLDKVEAEV	0,7407
A02	HLA-A*02:03	995	RLITGRLQSL	0,6909
A02	HLA-A*02:03	1000	RLQSLQTYV	0,9011
A02	HLA-A*02:03	1047	YHLMSFPQSA	0,7925
A02	HLA-A*02:03	1048	HLMSFPQSA	0,9264
A02	HLA-A*02:03	1060	VVFLHVTYV	0,8543
A02	HLA-A*02:03	1171	GINASVVNI	0,7487
A02	HLA-A*02:03	1185	RLNEVAKNL	0,8898
A02	HLA-A*02:03	1192	NLNESLIDL	0,7558
A02	HLA-A*02:03	1220	FIAGLIAIV	0,9522
A02	HLA-A*02:04	269	YLQPRTFLL	0,8826
A02	HLA-A*02:04	417	KIADYNYKL	0,7529
A02	HLA-A*02:04	691	SIIAYTMSL	0,6521
A02	HLA-A*02:04	821	LLFNKVTLA	0,5441
A02	HLA-A*02:04	976	VLNDILSRL	0,7971
A02	HLA-A*02:04	1060	VVFLHVTYV	0,5925
A02	HLA-A*02:05	28	YTNSFTRGV	0,5152
A02	HLA-A*02:05	133	FQFCNDPFL	0,6267
A02	HLA-A*02:05	269	YLQPRTFLL	0,8636
A02	HLA-A*02:05	417	KIADYNYKL	0,9165
A02	HLA-A*02:05	424	KLPDDFTGCV	0,6183
A02	HLA-A*02:05	691	SIIAYTMSL	0,7523
A02	HLA-A*02:05	718	FTISVTTEI	0,7976
A02	HLA-A*02:05	777	NTQEVFAQV	0,5189
A02	HLA-A*02:05	869	MIAQYTSAL	0,6311
A02	HLA-A*02:05	975	SVLNDILSRL	0,5129
A02	HLA-A*02:05	976	VLNDILSRL	0,9314
A02	HLA-A*02:05	1000	RLQSLQTYV	0,5133
A02	HLA-A*02:05	1060	VVFLHVTYV	0,6498
A02	HLA-A*02:05	1185	RLNEVAKNL	0,5946
A02	HLA-A*02:05	1220	FIAGLIAIV	0,7813
A02	HLA-A*02:06	133	FQFCNDPFL	0,7104
A02	HLA-A*02:06	133	FQFCNDPFLGV	0,5205
A02	HLA-A*02:06	269	YLQPRTFLL	0,8955
A02	HLA-A*02:06	417	KIADYNYKL	0,9064
A02	HLA-A*02:06	424	KLPDDFTGCV	0,5686
A02	HLA-A*02:06	612	YQDVNCTEV	0,5256
A02	HLA-A*02:06	691	SIIAYTMSL	0,847
A02	HLA-A*02:06	712	IAIPTNFTI	0,5224
A02	HLA-A*02:06	718	FTISVTTEI	0,8184
A02	HLA-A*02:06	721	SVTTEILPV	0,5642
A02	HLA-A*02:06	821	LLFNKVTLA	0,5419

A02	HLA-A*02:06	894	LQIPFAMQM	0,6147
A02	HLA-A*02:06	976	VLNDILSRL	0,8346
A02	HLA-A*02:06	983	RLDKVEAEV	0,6135
A02	HLA-A*02:06	1048	HLMSFPQSA	0,5289
A02	HLA-A*02:06	1060	VVFLHVTYV	0,7813
A02	HLA-A*02:06	1136	TVYDPLQPEL	0,5968
A02	HLA-A*02:06	1220	FIAGLIAIV	0,81
A02	HLA-A*02:07	269	YLQPRTFLL	0,8775
A02	HLA-A*02:09	109	TLDSKTQSL	0,6782
A02	HLA-A*02:09	133	FQFCNDPFL	0,518
A02	HLA-A*02:09	269	YLQPRTFLL	0,973
A02	HLA-A*02:09	386	KLNDLCFTNV	0,7108
A02	HLA-A*02:09	417	KIADYNYKL	0,909
A02	HLA-A*02:09	424	KLPDDFTGCV	0,5916
A02	HLA-A*02:09	515	FELLHAPATV	0,6033
A02	HLA-A*02:09	610	VLYQDVNCTEV	0,5092
A02	HLA-A*02:09	691	SIIAYTMSL	0,7995
A02	HLA-A*02:09	718	FTISVTTEI	0,5257
A02	HLA-A*02:09	821	LLFNKVTLA	0,7857
A02	HLA-A*02:09	857	GLTVLPPLL	0,6786
A02	HLA-A*02:09	915	VLYENQKLI	0,5378
A02	HLA-A*02:09	975	SVLNDILSRL	0,5124
A02	HLA-A*02:09	976	VLNDILSRL	0,9507
A02	HLA-A*02:09	983	RLDKVEAEV	0,8609
A02	HLA-A*02:09	1000	RLQSLQTYV	0,7431
A02	HLA-A*02:09	1047	YHLMSFPQSA	0,5452
A02	HLA-A*02:09	1048	HLMSFPQSA	0,7385
A02	HLA-A*02:09	1060	VVFLHVTYV	0,6948
A02	HLA-A*02:09	1185	RLNEVAKNL	0,6189
A02	HLA-A*02:09	1192	NLNESLIDL	0,6972
A02	HLA-A*02:09	1220	FIAGLIAIV	0,8207
A02	HLA-A*02:11	109	TLDSKTQSL	0,8988
A02	HLA-A*02:11	269	YLQPRTFLL	0,9932
A02	HLA-A*02:11	386	KLNDLCFTNV	0,8871
A02	HLA-A*02:11	417	KIADYNYKL	0,97
A02	HLA-A*02:11	424	KLPDDFTGCV	0,7417
A02	HLA-A*02:11	515	FELLHAPATV	0,7889
A02	HLA-A*02:11	610	VLYQDVNCTEV	0,6974
A02	HLA-A*02:11	691	SIIAYTMSL	0,9432
A02	HLA-A*02:11	718	FTISVTTEI	0,8216
A02	HLA-A*02:11	821	LLFNKVTLA	0,9443
A02	HLA-A*02:11	857	GLTVLPPLL	0,7856
A02	HLA-A*02:11	915	VLYENQKLI	0,8632
A02	HLA-A*02:11	958	ALNTLVKQL	0,8141
A02	HLA-A*02:11	975	SVLNDILSRL	0,7945
A02	HLA-A*02:11	976	VLNDILSRL	0,9917
A02	HLA-A*02:11	983	RLDKVEAEV	0,9677
A02	HLA-A*02:11	1000	RLQSLQTYV	0,9419
A02	HLA-A*02:11	1047	YHLMSFPQSA	0,7518
A02	HLA-A*02:11	1048	HLMSFPQSA	0,9136

A02	HLA-A*02:11	1060	VVFLHVTYV	0,8865
A02	HLA-A*02:11	1171	GINASVVNI	0,7552
A02	HLA-A*02:11	1185	RLNEVAKNL	0,9099
A02	HLA-A*02:11	1192	NLNESLIDL	0,8952
A02	HLA-A*02:11	1220	FIAGLIAIV	0,9533
A02	HLA-A*02:12	109	TLDSKTQSL	0,8428
A02	HLA-A*02:12	269	YLQPRTFLL	0,9818
A02	HLA-A*02:12	386	KLNDLCFTNV	0,7871
A02	HLA-A*02:12	417	KIADYNYKL	0,9054
A02	HLA-A*02:12	424	KLPDDFTGCV	0,6822
A02	HLA-A*02:12	515	FELLHAPATV	0,681
A02	HLA-A*02:12	610	VLYQDVNCTEV	0,5467
A02	HLA-A*02:12	691	SIIAYTMSL	0,7857
A02	HLA-A*02:12	718	FTISVTTEI	0,5137
A02	HLA-A*02:12	821	LLFNKVTLA	0,8068
A02	HLA-A*02:12	857	GLTVLPPLL	0,6662
A02	HLA-A*02:12	915	VLYENQKLI	0,6143
A02	HLA-A*02:12	958	ALNTLVKQL	0,608
A02	HLA-A*02:12	975	SVLNDILSRL	0,6353
A02	HLA-A*02:12	976	VLNDILSRL	0,9646
A02	HLA-A*02:12	983	RLDKVEAEV	0,9238
A02	HLA-A*02:12	1000	RLQSLQTYV	0,8752
A02	HLA-A*02:12	1047	YHLMSFPQSA	0,6061
A02	HLA-A*02:12	1048	HLMSFPQSA	0,7977
A02	HLA-A*02:12	1060	VVFLHVTYV	0,6387
A02	HLA-A*02:12	1185	RLNEVAKNL	0,7519
A02	HLA-A*02:12	1192	NLNESLIDL	0,8032
A02	HLA-A*02:12	1196	SLIDLQEL	0,5049
A02	HLA-A*02:12	1220	FIAGLIAIV	0,8684
A02	HLA-A*02:13	109	TLDSKTQSL	0,6983
A02	HLA-A*02:13	269	YLQPRTFLL	0,941
A02	HLA-A*02:13	386	KLNDLCFTNV	0,8077
A02	HLA-A*02:13	417	KIADYNYKL	0,6473
A02	HLA-A*02:13	424	KLPDDFTGCV	0,6563
A02	HLA-A*02:13	515	FELLHAPATV	0,6753
A02	HLA-A*02:13	610	VLYQDVNCTEV	0,5488
A02	HLA-A*02:13	634	RVYSTGSNV	0,5202
A02	HLA-A*02:13	691	SIIAYTMSL	0,7124
A02	HLA-A*02:13	762	QLNRALTGI	0,5324
A02	HLA-A*02:13	821	LLFNKVTLA	0,8697
A02	HLA-A*02:13	915	VLYENQKLI	0,7047
A02	HLA-A*02:13	937	SLSSTASAL	0,5679
A02	HLA-A*02:13	958	ALNTLVKQL	0,6032
A02	HLA-A*02:13	975	SVLNDILSRL	0,6032
A02	HLA-A*02:13	976	VLNDILSRL	0,945
A02	HLA-A*02:13	983	RLDKVEAEV	0,7965
A02	HLA-A*02:13	1000	RLQSLQTYV	0,8865
A02	HLA-A*02:13	1047	YHLMSFPQSA	0,5921
A02	HLA-A*02:13	1048	HLMSFPQSA	0,8168
A02	HLA-A*02:13	1060	VVFLHVTYV	0,6866

A02	HLA-A*02:13	1171	GINASVVNI	0,5495
A02	HLA-A*02:13	1185	RLNEVAKNL	0,7327
A02	HLA-A*02:13	1192	NLNESLIDL	0,6257
A02	HLA-A*02:13	1220	FIAGLIAIV	0,8509
A02	HLA-A*02:14	133	FQFCNDPFL	0,6515
A02	HLA-A*02:14	269	YLQPRTFLL	0,8753
A02	HLA-A*02:14	417	KIADYNYKL	0,8777
A02	HLA-A*02:14	424	KLPDDFTGCV	0,5064
A02	HLA-A*02:14	691	SIIAYTMSL	0,7378
A02	HLA-A*02:14	718	FTISVTTEI	0,6932
A02	HLA-A*02:14	894	LQIPFAMQM	0,5806
A02	HLA-A*02:14	976	VLNDILSRL	0,8034
A02	HLA-A*02:14	983	RLDKVEAEV	0,5388
A02	HLA-A*02:14	1060	VVFLHVTYV	0,6298
A02	HLA-A*02:14	1220	FIAGLIAIV	0,6444
A02	HLA-A*02:16	109	TLDSKTQSL	0,7274
A02	HLA-A*02:16	133	FQFCNDPFL	0,6062
A02	HLA-A*02:16	222	ALEPLVDLPIGI	0,5004
A02	HLA-A*02:16	269	YLQPRTFLL	0,9796
A02	HLA-A*02:16	386	KLNDLCFTNV	0,7336
A02	HLA-A*02:16	417	KIADYNYKL	0,9083
A02	HLA-A*02:16	424	KLPDDFTGCV	0,6179
A02	HLA-A*02:16	515	FELLHAPATV	0,7196
A02	HLA-A*02:16	610	VLYQDVNCTEV	0,6397
A02	HLA-A*02:16	691	SIIAYTMSL	0,8424
A02	HLA-A*02:16	718	FTISVTTEI	0,6337
A02	HLA-A*02:16	821	LLFNKVTLA	0,8492
A02	HLA-A*02:16	857	GLTVLPPLL	0,7288
A02	HLA-A*02:16	915	VLYENQKLI	0,6401
A02	HLA-A*02:16	958	ALNTLVKQL	0,5125
A02	HLA-A*02:16	975	SVLNDILSRL	0,55
A02	HLA-A*02:16	976	VLNDILSRL	0,953
A02	HLA-A*02:16	983	RLDKVEAEV	0,9079
A02	HLA-A*02:16	1000	RLQSLQTYV	0,8152
A02	HLA-A*02:16	1047	YHLMSFPQSA	0,6323
A02	HLA-A*02:16	1048	HLMSFPQSA	0,8072
A02	HLA-A*02:16	1060	VVFLHVTYV	0,7386
A02	HLA-A*02:16	1185	RLNEVAKNL	0,6572
A02	HLA-A*02:16	1192	NLNESLIDL	0,7695
A02	HLA-A*02:16	1220	FIAGLIAIV	0,8906
A02	HLA-A*02:17	269	YLQPRTFLL	0,8989
A02	HLA-A*02:17	417	KIADYNYKL	0,7084
A02	HLA-A*02:17	691	SIIAYTMSL	0,5715
A02	HLA-A*02:17	976	VLNDILSRL	0,7759
A02	HLA-A*02:18	269	YLQPRTFLL	0,8775
A02	HLA-A*02:19	109	TLDSKTQSL	0,8021
A02	HLA-A*02:19	269	YLQPRTFLL	0,9638
A02	HLA-A*02:19	386	KLNDLCFTNV	0,6484
A02	HLA-A*02:19	417	KIADYNYKL	0,7644
A02	HLA-A*02:19	424	KLPDDFTGCV	0,5568

A02	HLA-A*02:19	515	FELLHAPATV	0,5795
A02	HLA-A*02:19	516	ELLHAPATV	0,6189
A02	HLA-A*02:19	691	SIIAYTMSL	0,6332
A02	HLA-A*02:19	821	LLFNKVTLA	0,6129
A02	HLA-A*02:19	975	SVLNDILSRL	0,5189
A02	HLA-A*02:19	976	VLNDILSRL	0,9385
A02	HLA-A*02:19	983	RLDKVEAEV	0,8427
A02	HLA-A*02:19	1000	RLQSLQTYV	0,7225
A02	HLA-A*02:19	1048	HLMSFPQSA	0,6609
A02	HLA-A*02:19	1185	RLNEVAKNL	0,5986
A02	HLA-A*02:19	1192	NLNESLIDL	0,8007
A02	HLA-A*02:19	1220	FIAGLIAIV	0,8159
A02	HLA-A*02:20	269	YLQPRTFLL	0,9372
A02	HLA-A*02:20	386	KLNDLCFTNV	0,609
A02	HLA-A*02:20	417	KIADYNYKL	0,8535
A02	HLA-A*02:20	691	SIIAYTMSL	0,7417
A02	HLA-A*02:20	718	FTISVTTEI	0,5048
A02	HLA-A*02:20	821	LLFNKVTLA	0,6533
A02	HLA-A*02:20	857	GLTVLPPLL	0,5648
A02	HLA-A*02:20	976	VLNDILSRL	0,883
A02	HLA-A*02:20	983	RLDKVEAEV	0,7865
A02	HLA-A*02:20	1000	RLQSLQTYV	0,6742
A02	HLA-A*02:20	1048	HLMSFPQSA	0,6259
A02	HLA-A*02:20	1060	VVFLHVTYV	0,6374
A02	HLA-A*02:20	1192	NLNESLIDL	0,5432
A02	HLA-A*02:20	1220	FIAGLIAIV	0,6882
A02	HLA-A*02:21	133	FQFCNDPFL	0,7104
A02	HLA-A*02:21	133	FQFCNDPFLGV	0,5205
A02	HLA-A*02:21	269	YLQPRTFLL	0,8955
A02	HLA-A*02:21	417	KIADYNYKL	0,9064
A02	HLA-A*02:21	424	KLPDDFTGCV	0,5686
A02	HLA-A*02:21	612	YQDVNCTEV	0,5256
A02	HLA-A*02:21	691	SIIAYTMSL	0,847
A02	HLA-A*02:21	712	IAIPTNFTI	0,5224
A02	HLA-A*02:21	718	FTISVTTEI	0,8184
A02	HLA-A*02:21	721	SVTTEILPV	0,5642
A02	HLA-A*02:21	821	LLFNKVTLA	0,5419
A02	HLA-A*02:21	894	LQIPFAMQM	0,6147
A02	HLA-A*02:21	976	VLNDILSRL	0,8346
A02	HLA-A*02:21	983	RLDKVEAEV	0,6135
A02	HLA-A*02:21	1048	HLMSFPQSA	0,5289
A02	HLA-A*02:21	1060	VVFLHVTYV	0,7813
A02	HLA-A*02:21	1136	TVYDPLQPEL	0,5968
A02	HLA-A*02:21	1220	FIAGLIAIV	0,81
A02	HLA-A*02:22	109	TLDSKTQSL	0,8377
A02	HLA-A*02:22	133	FQFCNDPFL	0,6508
A02	HLA-A*02:22	269	YLQPRTFLL	0,9799
A02	HLA-A*02:22	386	KLNDLCFTNV	0,8656
A02	HLA-A*02:22	417	KIADYNYKL	0,9698
A02	HLA-A*02:22	424	KLPDDFTGCV	0,831

A02	HLA-A*02:22	512	VLSFELLHA	0,665
A02	HLA-A*02:22	515	FELLHAPATV	0,7224
A02	HLA-A*02:22	610	VLYQDVNCTEV	0,651
A02	HLA-A*02:22	691	SIIAYTMSL	0,882
A02	HLA-A*02:22	718	FTISVTTEI	0,8007
A02	HLA-A*02:22	762	QLNRALTGI	0,6498
A02	HLA-A*02:22	821	LLFNKVTLA	0,9056
A02	HLA-A*02:22	857	GLTVLPPLL	0,7806
A02	HLA-A*02:22	869	MIAQYTSAL	0,8034
A02	HLA-A*02:22	915	VLYENQKLI	0,746
A02	HLA-A*02:22	937	SLSSTASAL	0,7146
A02	HLA-A*02:22	947	KLQDVVNQNA	0,6761
A02	HLA-A*02:22	958	ALNTLVKQL	0,8347
A02	HLA-A*02:22	975	SVLNDILSRL	0,8914
A02	HLA-A*02:22	976	VLNDILSRL	0,9899
A02	HLA-A*02:22	983	RLDKVEAEV	0,8989
A02	HLA-A*02:22	1000	RLQSLQTYV	0,9071
A02	HLA-A*02:22	1047	YHLMSFPQSA	0,7592
A02	HLA-A*02:22	1048	HLMSFPQSA	0,902
A02	HLA-A*02:22	1060	VVFLHVTYV	0,7982
A02	HLA-A*02:22	1095	FVSNGTHWFV	0,6858
A02	HLA-A*02:22	1171	GINASVVNI	0,6497
A02	HLA-A*02:22	1185	RLNEVAKNL	0,8958
A02	HLA-A*02:22	1192	NLNESLIDL	0,8894
A02	HLA-A*02:22	1196	SLIDLQEL	0,7342
A02	HLA-A*02:22	1220	FIAGLIAIV	0,9461
A02	HLA-A*02:24	109	TLDSKTQSL	0,6782
A02	HLA-A*02:24	133	FQFCNDPFL	0,518
A02	HLA-A*02:24	269	YLQPRTFLL	0,973
A02	HLA-A*02:24	386	KLNDLCFTNV	0,7108
A02	HLA-A*02:24	417	KIADYNYKL	0,909
A02	HLA-A*02:24	424	KLPDDFTGCV	0,5916
A02	HLA-A*02:24	515	FELLHAPATV	0,6033
A02	HLA-A*02:24	610	VLYQDVNCTEV	0,5092
A02	HLA-A*02:24	691	SIIAYTMSL	0,7995
A02	HLA-A*02:24	718	FTISVTTEI	0,5257
A02	HLA-A*02:24	821	LLFNKVTLA	0,7857
A02	HLA-A*02:24	857	GLTVLPPLL	0,6786
A02	HLA-A*02:24	915	VLYENQKLI	0,5378
A02	HLA-A*02:24	975	SVLNDILSRL	0,5124
A02	HLA-A*02:24	976	VLNDILSRL	0,9507
A02	HLA-A*02:24	983	RLDKVEAEV	0,8609
A02	HLA-A*02:24	1000	RLQSLQTYV	0,7431
A02	HLA-A*02:24	1047	YHLMSFPQSA	0,5452
A02	HLA-A*02:24	1048	HLMSFPQSA	0,7385
A02	HLA-A*02:24	1060	VVFLHVTYV	0,6948
A02	HLA-A*02:24	1185	RLNEVAKNL	0,6189
A02	HLA-A*02:24	1192	NLNESLIDL	0,6972
A02	HLA-A*02:24	1220	FIAGLIAIV	0,8207
A02	HLA-A*02:25	109	TLDSKTQSL	0,6782

A02	HLA-A*02:25	133	FQFCNDPFL	0,518
A02	HLA-A*02:25	269	YLQPRTFLL	0,973
A02	HLA-A*02:25	386	KLNDLCFTNV	0,7108
A02	HLA-A*02:25	417	KIADYNYKL	0,909
A02	HLA-A*02:25	424	KLPDDFTGCV	0,5916
A02	HLA-A*02:25	515	FELLHAPATV	0,6033
A02	HLA-A*02:25	610	VLYQDVNCTEV	0,5092
A02	HLA-A*02:25	691	SIIAYTMSL	0,7995
A02	HLA-A*02:25	718	FTISVTTEI	0,5257
A02	HLA-A*02:25	821	LLFNKVTLA	0,7857
A02	HLA-A*02:25	857	GLTVLPPLL	0,6786
A02	HLA-A*02:25	915	VLYENQKLI	0,5378
A02	HLA-A*02:25	975	SVLNDILSRL	0,5124
A02	HLA-A*02:25	976	VLNDILSRL	0,9507
A02	HLA-A*02:25	983	RLDKVEAEV	0,8609
A02	HLA-A*02:25	1000	RLQSLQTYV	0,7431
A02	HLA-A*02:25	1047	YHLMSFPQSA	0,5452
A02	HLA-A*02:25	1048	HLMSFPQSA	0,7385
A02	HLA-A*02:25	1060	VVFLHVTYV	0,6948
A02	HLA-A*02:25	1185	RLNEVAKNL	0,6189
A02	HLA-A*02:25	1192	NLNESLIDL	0,6972
A02	HLA-A*02:25	1220	FIAGLIAIV	0,8207
A02	HLA-A*02:26	269	YLQPRTFLL	0,9227
A02	HLA-A*02:26	386	KLNDLCFTNV	0,779
A02	HLA-A*02:26	417	KIADYNYKL	0,6418
A02	HLA-A*02:26	424	KLPDDFTGCV	0,6197
A02	HLA-A*02:26	515	FELLHAPATV	0,6456
A02	HLA-A*02:26	610	VLYQDVNCTEV	0,564
A02	HLA-A*02:26	634	RVYSTGSNV	0,5791
A02	HLA-A*02:26	691	SIIAYTMSL	0,75
A02	HLA-A*02:26	718	FTISVTTEI	0,5223
A02	HLA-A*02:26	821	LLFNKVTLA	0,8849
A02	HLA-A*02:26	915	VLYENQKLI	0,6862
A02	HLA-A*02:26	975	SVLNDILSRL	0,5439
A02	HLA-A*02:26	976	VLNDILSRL	0,9394
A02	HLA-A*02:26	983	RLDKVEAEV	0,7333
A02	HLA-A*02:26	1000	RLQSLQTYV	0,7922
A02	HLA-A*02:26	1047	YHLMSFPQSA	0,5858
A02	HLA-A*02:26	1048	HLMSFPQSA	0,8161
A02	HLA-A*02:26	1060	VVFLHVTYV	0,7683
A02	HLA-A*02:26	1171	GINASVVNI	0,5171
A02	HLA-A*02:26	1185	RLNEVAKNL	0,664
A02	HLA-A*02:26	1192	NLNESLIDL	0,524
A02	HLA-A*02:26	1220	FIAGLIAIV	0,8354
A02	HLA-A*02:27	109	TLDSKTQSL	0,7308
A02	HLA-A*02:27	269	YLQPRTFLL	0,9647
A02	HLA-A*02:27	386	KLNDLCFTNV	0,7459
A02	HLA-A*02:27	417	KIADYNYKL	0,7613
A02	HLA-A*02:27	424	KLPDDFTGCV	0,5772
A02	HLA-A*02:27	515	FELLHAPATV	0,627

A02	HLA-A*02:27	610	VLYQDVNCTEV	0,514
A02	HLA-A*02:27	691	SIIAYTMSL	0,6826
A02	HLA-A*02:27	821	LLFNKVTLA	0,8062
A02	HLA-A*02:27	857	GLTVLPPLL	0,5289
A02	HLA-A*02:27	915	VLYENQKLI	0,6196
A02	HLA-A*02:27	958	ALNTLVKQL	0,5442
A02	HLA-A*02:27	975	SVLNDILSRL	0,5493
A02	HLA-A*02:27	976	VLNDILSRL	0,9341
A02	HLA-A*02:27	983	RLDKVEAEV	0,8729
A02	HLA-A*02:27	1000	RLQSLQTYV	0,8703
A02	HLA-A*02:27	1047	YHLMSFPQSA	0,5242
A02	HLA-A*02:27	1048	HLMSFPQSA	0,7531
A02	HLA-A*02:27	1060	VVFLHVTYV	0,5815
A02	HLA-A*02:27	1185	RLNEVAKNL	0,685
A02	HLA-A*02:27	1192	NLNESLIDL	0,6819
A02	HLA-A*02:27	1220	FIAGLIAIV	0,798
A02	HLA-A*02:28	133	FQFCNDPFL	0,7104
A02	HLA-A*02:28	133	FQFCNDPFLGV	0,5205
A02	HLA-A*02:28	269	YLQPRTFLL	0,8955
A02	HLA-A*02:28	417	KIADYNYKL	0,9064
A02	HLA-A*02:28	424	KLPDDFTGCV	0,5686
A02	HLA-A*02:28	612	YQDVNCTEV	0,5256
A02	HLA-A*02:28	691	SIIAYTMSL	0,847
A02	HLA-A*02:28	712	IAIPTNFTI	0,5224
A02	HLA-A*02:28	718	FTISVTTEI	0,8184
A02	HLA-A*02:28	721	SVTTEILPV	0,5642
A02	HLA-A*02:28	821	LLFNKVTLA	0,5419
A02	HLA-A*02:28	894	LQIPFAMQM	0,6147
A02	HLA-A*02:28	976	VLNDILSRL	0,8346
A02	HLA-A*02:28	983	RLDKVEAEV	0,6135
A02	HLA-A*02:28	1048	HLMSFPQSA	0,5289
A02	HLA-A*02:28	1060	VVFLHVTYV	0,7813
A02	HLA-A*02:28	1136	TVYDPLQPEL	0,5968
A02	HLA-A*02:28	1220	FIAGLIAIV	0,81
A02	HLA-A*02:30	109	TLDSKTQSL	0,6782
A02	HLA-A*02:30	133	FQFCNDPFL	0,518
A02	HLA-A*02:30	269	YLQPRTFLL	0,973
A02	HLA-A*02:30	386	KLNDLCFTNV	0,7108
A02	HLA-A*02:30	417	KIADYNYKL	0,909
A02	HLA-A*02:30	424	KLPDDFTGCV	0,5916
A02	HLA-A*02:30	515	FELLHAPATV	0,6033
A02	HLA-A*02:30	610	VLYQDVNCTEV	0,5092
A02	HLA-A*02:30	691	SIIAYTMSL	0,7995
A02	HLA-A*02:30	718	FTISVTTEI	0,5257
A02	HLA-A*02:30	821	LLFNKVTLA	0,7857
A02	HLA-A*02:30	857	GLTVLPPLL	0,6786
A02	HLA-A*02:30	915	VLYENQKLI	0,5378
A02	HLA-A*02:30	975	SVLNDILSRL	0,5124
A02	HLA-A*02:30	976	VLNDILSRL	0,9507
A02	HLA-A*02:30	983	RLDKVEAEV	0,8609

A02	HLA-A*02:30	1000	RLQSLQTYV	0,7431
A02	HLA-A*02:30	1047	YHLMSFPQSA	0,5452
A02	HLA-A*02:30	1048	HLMSFPQSA	0,7385
A02	HLA-A*02:30	1060	VVFLHVTYV	0,6948
A02	HLA-A*02:30	1185	RLNEVAKNL	0,6189
A02	HLA-A*02:30	1192	NLNESLIDL	0,6972
A02	HLA-A*02:30	1220	FIAGLIAIV	0,8207
A02	HLA-A*02:31	109	TLDSKTQSL	0,6782
A02	HLA-A*02:31	133	FQFCNDPFL	0,518
A02	HLA-A*02:31	269	YLQPRTFLL	0,973
A02	HLA-A*02:31	386	KLNDLCFTNV	0,7108
A02	HLA-A*02:31	417	KIADYNYKL	0,909
A02	HLA-A*02:31	424	KLPDDFTGCV	0,5916
A02	HLA-A*02:31	515	FELLHAPATV	0,6033
A02	HLA-A*02:31	610	VLYQDVNCTEV	0,5092
A02	HLA-A*02:31	691	SIIAYTMSL	0,7995
A02	HLA-A*02:31	718	FTISVTTEI	0,5257
A02	HLA-A*02:31	821	LLFNKVTLA	0,7857
A02	HLA-A*02:31	857	GLTVLPPLL	0,6786
A02	HLA-A*02:31	915	VLYENQKLI	0,5378
A02	HLA-A*02:31	975	SVLNDILSRL	0,5124
A02	HLA-A*02:31	976	VLNDILSRL	0,9507
A02	HLA-A*02:31	983	RLDKVEAEV	0,8609
A02	HLA-A*02:31	1000	RLQSLQTYV	0,7431
A02	HLA-A*02:31	1047	YHLMSFPQSA	0,5452
A02	HLA-A*02:31	1048	HLMSFPQSA	0,7385
A02	HLA-A*02:31	1060	VVFLHVTYV	0,6948
A02	HLA-A*02:31	1185	RLNEVAKNL	0,6189
A02	HLA-A*02:31	1192	NLNESLIDL	0,6972
A02	HLA-A*02:31	1220	FIAGLIAIV	0,8207
A02	HLA-A*02:36	109	TLDSKTQSL	0,6446
A02	HLA-A*02:36	269	YLQPRTFLL	0,9384
A02	HLA-A*02:36	386	KLNDLCFTNV	0,5465
A02	HLA-A*02:36	417	KIADYNYKL	0,763
A02	HLA-A*02:36	515	FELLHAPATV	0,5014
A02	HLA-A*02:36	516	ELLHAPATV	0,5347
A02	HLA-A*02:36	691	SIIAYTMSL	0,6521
A02	HLA-A*02:36	821	LLFNKVTLA	0,595
A02	HLA-A*02:36	976	VLNDILSRL	0,9103
A02	HLA-A*02:36	983	RLDKVEAEV	0,7362
A02	HLA-A*02:36	1000	RLQSLQTYV	0,5633
A02	HLA-A*02:36	1048	HLMSFPQSA	0,5791
A02	HLA-A*02:36	1060	VVFLHVTYV	0,5499
A02	HLA-A*02:36	1192	NLNESLIDL	0,6714
A02	HLA-A*02:36	1220	FIAGLIAIV	0,7437
A02	HLA-A*02:38	109	TLDSKTQSL	0,6763
A02	HLA-A*02:38	269	YLQPRTFLL	0,8978
A02	HLA-A*02:38	386	KLNDLCFTNV	0,6697
A02	HLA-A*02:38	417	KIADYNYKL	0,6147
A02	HLA-A*02:38	424	KLPDDFTGCV	0,5937

A02	HLA-A*02:38	515	FELLHAPATV	0,6135
A02	HLA-A*02:38	610	VLYQDVNCTEV	0,5165
A02	HLA-A*02:38	691	SIIAYTMSL	0,7292
A02	HLA-A*02:38	718	FTISVTTEI	0,5114
A02	HLA-A*02:38	821	LLFNKVTLA	0,7991
A02	HLA-A*02:38	915	VLYENQKLI	0,6879
A02	HLA-A*02:38	937	SLSSTASAL	0,5212
A02	HLA-A*02:38	958	ALNTLVKQL	0,5209
A02	HLA-A*02:38	975	SVLNDILSRL	0,5586
A02	HLA-A*02:38	976	VLNDILSRL	0,9012
A02	HLA-A*02:38	983	RLDKVEAEV	0,7049
A02	HLA-A*02:38	1000	RLQSLQTYV	0,7606
A02	HLA-A*02:38	1047	YHLMSFPQSA	0,5778
A02	HLA-A*02:38	1048	HLMSFPQSA	0,7518
A02	HLA-A*02:38	1060	VVFLHVTYV	0,6814
A02	HLA-A*02:38	1171	GINASVVNI	0,5375
A02	HLA-A*02:38	1185	RLNEVAKNL	0,6207
A02	HLA-A*02:38	1192	NLNESLIDL	0,6694
A02	HLA-A*02:38	1220	FIAGLIAIV	0,8041
A02	HLA-A*02:39	269	YLQPRTFLL	0,9532
A02	HLA-A*02:39	417	KIADYNYKL	0,7841
A02	HLA-A*02:39	691	SIIAYTMSL	0,5907
A02	HLA-A*02:39	821	LLFNKVTLA	0,5231
A02	HLA-A*02:39	857	GLTVLPPLL	0,5068
A02	HLA-A*02:39	976	VLNDILSRL	0,8817
A02	HLA-A*02:39	983	RLDKVEAEV	0,6488
A02	HLA-A*02:39	1000	RLQSLQTYV	0,5067
A02	HLA-A*02:39	1192	NLNESLIDL	0,5275
A02	HLA-A*02:39	1220	FIAGLIAIV	0,6464
A02	HLA-A*02:40	109	TLDSKTQSL	0,6782
A02	HLA-A*02:40	133	FQFCNDPFL	0,518
A02	HLA-A*02:40	269	YLQPRTFLL	0,973
A02	HLA-A*02:40	386	KLNDLCFTNV	0,7108
A02	HLA-A*02:40	417	KIADYNYKL	0,909
A02	HLA-A*02:40	424	KLPDDFTGCV	0,5916
A02	HLA-A*02:40	515	FELLHAPATV	0,6033
A02	HLA-A*02:40	610	VLYQDVNCTEV	0,5092
A02	HLA-A*02:40	691	SIIAYTMSL	0,7995
A02	HLA-A*02:40	718	FTISVTTEI	0,5257
A02	HLA-A*02:40	821	LLFNKVTLA	0,7857
A02	HLA-A*02:40	857	GLTVLPPLL	0,6786
A02	HLA-A*02:40	915	VLYENQKLI	0,5378
A02	HLA-A*02:40	975	SVLNDILSRL	0,5124
A02	HLA-A*02:40	976	VLNDILSRL	0,9507
A02	HLA-A*02:40	983	RLDKVEAEV	0,8609
A02	HLA-A*02:40	1000	RLQSLQTYV	0,7431
A02	HLA-A*02:40	1047	YHLMSFPQSA	0,5452
A02	HLA-A*02:40	1048	HLMSFPQSA	0,7385
A02	HLA-A*02:40	1060	VVFLHVTYV	0,6948
A02	HLA-A*02:40	1185	RLNEVAKNL	0,6189

A02	HLA-A*02:40	1192	NLNESLIDL	0,6972
A02	HLA-A*02:40	1220	FIAGLIAIV	0,8207
A02	HLA-A*02:44	109	TLDSKTQSL	0,722
A02	HLA-A*02:44	133	FQFCNDPFL	0,6901
A02	HLA-A*02:44	269	YLQPRTFLL	0,9483
A02	HLA-A*02:44	386	KLNDLCFTNV	0,6273
A02	HLA-A*02:44	417	KIADYNYKL	0,9374
A02	HLA-A*02:44	424	KLPDDFTGCV	0,713
A02	HLA-A*02:44	612	YQDVNCTEV	0,6481
A02	HLA-A*02:44	691	SIIAYTMSL	0,873
A02	HLA-A*02:44	718	FTISVTTEI	0,8345
A02	HLA-A*02:44	721	SVTTEILPV	0,6283
A02	HLA-A*02:44	777	NTQEVFAQV	0,5675
A02	HLA-A*02:44	786	KQIYKTPPI	0,5169
A02	HLA-A*02:44	821	LLFNKVTLA	0,6213
A02	HLA-A*02:44	869	MIAQYTSAL	0,5812
A02	HLA-A*02:44	976	VLNDILSRL	0,9188
A02	HLA-A*02:44	983	RLDKVEAEV	0,7861
A02	HLA-A*02:44	1000	RLQSLQTYV	0,7229
A02	HLA-A*02:44	1048	HLMSFPQSA	0,6725
A02	HLA-A*02:44	1060	VVFLHVTYV	0,7638
A02	HLA-A*02:44	1095	FVSNGTHWFV	0,5522
A02	HLA-A*02:44	1136	TVYDPLQPEL	0,5349
A02	HLA-A*02:44	1171	GINASVVNI	0,5357
A02	HLA-A*02:44	1185	RLNEVAKNL	0,5876
A02	HLA-A*02:44	1192	NLNESLIDL	0,6815
A02	HLA-A*02:44	1196	SLIDLQEL	0,5121
A02	HLA-A*02:44	1220	FIAGLIAIV	0,868
A02	HLA-A*02:45	269	YLQPRTFLL	0,87
A02	HLA-A*02:45	417	KIADYNYKL	0,7495
A02	HLA-A*02:45	691	SIIAYTMSL	0,5724
A02	HLA-A*02:45	976	VLNDILSRL	0,7909
A02	HLA-A*02:45	983	RLDKVEAEV	0,6527
A02	HLA-A*02:46	269	YLQPRTFLL	0,897
A02	HLA-A*02:46	417	KIADYNYKL	0,7857
A02	HLA-A*02:46	691	SIIAYTMSL	0,5832
A02	HLA-A*02:46	976	VLNDILSRL	0,8016
A02	HLA-A*02:46	983	RLDKVEAEV	0,6558
A02	HLA-A*02:47	109	TLDSKTQSL	0,8176
A02	HLA-A*02:47	133	FQFCNDPFL	0,5963
A02	HLA-A*02:47	225	PLVDLPIGI	0,5831
A02	HLA-A*02:47	269	YLQPRTFLL	0,9772
A02	HLA-A*02:47	386	KLNDLCFTNV	0,8117
A02	HLA-A*02:47	416	GKIADYNYKL	0,6744
A02	HLA-A*02:47	417	KIADYNYKL	0,9749
A02	HLA-A*02:47	424	KLPDDFTGCV	0,8227
A02	HLA-A*02:47	691	SIIAYTMSL	0,8546
A02	HLA-A*02:47	718	FTISVTTEI	0,7036
A02	HLA-A*02:47	821	LLFNKVTLA	0,8262
A02	HLA-A*02:47	857	GLTVLPPLL	0,7895

A02	HLA-A*02:47	869	MIAQYTSAL	0,7897
A02	HLA-A*02:47	915	VLYENQKLI	0,6812
A02	HLA-A*02:47	937	SLSSTASAL	0,6691
A02	HLA-A*02:47	958	ALNTLVKQL	0,8279
A02	HLA-A*02:47	975	SVLNDILSRL	0,8782
A02	HLA-A*02:47	976	VLNDILSRL	0,9886
A02	HLA-A*02:47	983	RLDKVEAEV	0,849
A02	HLA-A*02:47	1000	RLQSLQTYV	0,8402
A02	HLA-A*02:47	1047	YHLMSFPQSA	0,6304
A02	HLA-A*02:47	1048	HLMSFPQSA	0,8224
A02	HLA-A*02:47	1060	VVFLHVTYV	0,7118
A02	HLA-A*02:47	1095	FVSNGTHWFV	0,6164
A02	HLA-A*02:47	1185	RLNEVAKNL	0,9004
A02	HLA-A*02:47	1192	NLNESLIDL	0,8471
A02	HLA-A*02:47	1196	SLIDLQEL	0,7353
A02	HLA-A*02:47	1220	FIAGLIAIV	0,8847
A02	HLA-A*02:48	109	TLDSKTQSL	0,52
A02	HLA-A*02:48	269	YLQPRTFLL	0,9298
A02	HLA-A*02:48	417	KIADYNYKL	0,8626
A02	HLA-A*02:48	691	SIIAYTMSL	0,69
A02	HLA-A*02:48	976	VLNDILSRL	0,8779
A02	HLA-A*02:48	983	RLDKVEAEV	0,7153
A02	HLA-A*02:48	1000	RLQSLQTYV	0,6002
A02	HLA-A*02:48	1060	VVFLHVTYV	0,5094
A02	HLA-A*02:48	1185	RLNEVAKNL	0,5841
A02	HLA-A*02:49	109	TLDSKTQSL	0,8693
A02	HLA-A*02:49	269	YLQPRTFLL	0,9729
A02	HLA-A*02:49	386	KLNDLCFTNV	0,6741
A02	HLA-A*02:49	417	KIADYNYKL	0,8873
A02	HLA-A*02:49	424	KLPDDFTGCV	0,6103
A02	HLA-A*02:49	515	FELLHAPATV	0,5508
A02	HLA-A*02:49	612	YQDVNCTEV	0,5076
A02	HLA-A*02:49	691	SIIAYTMSL	0,6942
A02	HLA-A*02:49	821	LLFNKVTLA	0,6712
A02	HLA-A*02:49	857	GLTVLPPLL	0,5991
A02	HLA-A*02:49	975	SVLNDILSRL	0,5907
A02	HLA-A*02:49	976	VLNDILSRL	0,9452
A02	HLA-A*02:49	982	SRLDKVEAEV	0,5478
A02	HLA-A*02:49	983	RLDKVEAEV	0,9308
A02	HLA-A*02:49	1000	RLQSLQTYV	0,8043
A02	HLA-A*02:49	1048	HLMSFPQSA	0,6591
A02	HLA-A*02:49	1060	VVFLHVTYV	0,571
A02	HLA-A*02:49	1185	RLNEVAKNL	0,6724
A02	HLA-A*02:49	1192	NLNESLIDL	0,7823
A02	HLA-A*02:49	1220	FIAGLIAIV	0,7966
A02	HLA-A*02:51	133	FQFCNDPFL	0,7104
A02	HLA-A*02:51	133	FQFCNDPFLGV	0,5205
A02	HLA-A*02:51	269	YLQPRTFLL	0,8955
A02	HLA-A*02:51	417	KIADYNYKL	0,9064
A02	HLA-A*02:51	424	KLPDDFTGCV	0,5686

A02	HLA-A*02:51	612	YQDVNCTEV	0,5256
A02	HLA-A*02:51	691	SIIAYTMSL	0,847
A02	HLA-A*02:51	712	IAIPTNFTI	0,5224
A02	HLA-A*02:51	718	FTISVTTEI	0,8184
A02	HLA-A*02:51	721	SVTTEILPV	0,5642
A02	HLA-A*02:51	821	LLFNKVTLA	0,5419
A02	HLA-A*02:51	894	LQIPFAMQM	0,6147
A02	HLA-A*02:51	976	VLNDILSRL	0,8346
A02	HLA-A*02:51	983	RLDKVEAEV	0,6135
A02	HLA-A*02:51	1048	HLMSFPQSA	0,5289
A02	HLA-A*02:51	1060	VVFLHVTYV	0,7813
A02	HLA-A*02:51	1136	TVYDPLQPEL	0,5968
A02	HLA-A*02:51	1220	FIAGLIAIV	0,81
A02	HLA-A*02:54	109	TLDSKTQSL	0,6942
A02	HLA-A*02:54	269	YLQPRTFLL	0,9082
A02	HLA-A*02:54	417	KIADYNYKL	0,8163
A02	HLA-A*02:54	424	KLPDDFTGCV	0,5607
A02	HLA-A*02:54	516	ELLHAPATV	0,5493
A02	HLA-A*02:54	612	YQDVNCTEV	0,5542
A02	HLA-A*02:54	691	SIIAYTMSL	0,7472
A02	HLA-A*02:54	718	FTISVTTEI	0,731
A02	HLA-A*02:54	721	SVTTEILPV	0,5157
A02	HLA-A*02:54	777	NTQEVFAQV	0,6031
A02	HLA-A*02:54	976	VLNDILSRL	0,8799
A02	HLA-A*02:54	983	RLDKVEAEV	0,6743
A02	HLA-A*02:54	1000	RLQSLQTYV	0,5446
A02	HLA-A*02:54	1048	HLMSFPQSA	0,532
A02	HLA-A*02:54	1060	VVFLHVTYV	0,6044
A02	HLA-A*02:54	1192	NLNESLIDL	0,6437
A02	HLA-A*02:54	1220	FIAGLIAIV	0,7718
A02	HLA-A*02:56	269	YLQPRTFLL	0,6126
A02	HLA-A*02:56	417	KIADYNYKL	0,6147
A02	HLA-A*02:56	691	SIIAYTMSL	0,5351
A02	HLA-A*02:56	1060	VVFLHVTYV	0,5227
A02	HLA-A*02:57	269	YLQPRTFLL	0,7289
A02	HLA-A*02:57	417	KIADYNYKL	0,7431
A02	HLA-A*02:57	691	SIIAYTMSL	0,6331
A02	HLA-A*02:57	976	VLNDILSRL	0,5643
A02	HLA-A*02:57	1060	VVFLHVTYV	0,5368
A02	HLA-A*02:58	109	TLDSKTQSL	0,6907
A02	HLA-A*02:58	269	YLQPRTFLL	0,9723
A02	HLA-A*02:58	386	KLNDLCFTNV	0,6236
A02	HLA-A*02:58	417	KIADYNYKL	0,9119
A02	HLA-A*02:58	424	KLPDDFTGCV	0,5701
A02	HLA-A*02:58	691	SIIAYTMSL	0,7337
A02	HLA-A*02:58	821	LLFNKVTLA	0,6551
A02	HLA-A*02:58	857	GLTVLPPLL	0,6884
A02	HLA-A*02:58	975	SVLNDILSRL	0,5159
A02	HLA-A*02:58	976	VLNDILSRL	0,95
A02	HLA-A*02:58	983	RLDKVEAEV	0,8408

A02	HLA-A*02:58	1000	RLQSLQTYV	0,656
A02	HLA-A*02:58	1048	HLMSFPQSA	0,623
A02	HLA-A*02:58	1060	VVFLHVTYV	0,5564
A02	HLA-A*02:58	1185	RLNEVAKNL	0,6577
A02	HLA-A*02:58	1192	NLNESLIDL	0,6601
A02	HLA-A*02:58	1220	FIAGLIAIV	0,707
A02	HLA-A*02:59	109	TLDSKTQSL	0,6782
A02	HLA-A*02:59	133	FQFCNDPFL	0,518
A02	HLA-A*02:59	269	YLQPRTFLL	0,973
A02	HLA-A*02:59	386	KLNDLCFTNV	0,7108
A02	HLA-A*02:59	417	KIADYNYKL	0,909
A02	HLA-A*02:59	424	KLPDDFTGCV	0,5916
A02	HLA-A*02:59	515	FELLHAPATV	0,6033
A02	HLA-A*02:59	610	VLYQDVNCTEV	0,5092
A02	HLA-A*02:59	691	SIIAYTMSL	0,7995
A02	HLA-A*02:59	718	FTISVTTEI	0,5257
A02	HLA-A*02:59	821	LLFNKVTLA	0,7857
A02	HLA-A*02:59	857	GLTVLPPLL	0,6786
A02	HLA-A*02:59	915	VLYENQKLI	0,5378
A02	HLA-A*02:59	975	SVLNDILSRL	0,5124
A02	HLA-A*02:59	976	VLNDILSRL	0,9507
A02	HLA-A*02:59	983	RLDKVEAEV	0,8609
A02	HLA-A*02:59	1000	RLQSLQTYV	0,7431
A02	HLA-A*02:59	1047	YHLMSFPQSA	0,5452
A02	HLA-A*02:59	1048	HLMSFPQSA	0,7385
A02	HLA-A*02:59	1060	VVFLHVTYV	0,6948
A02	HLA-A*02:59	1185	RLNEVAKNL	0,6189
A02	HLA-A*02:59	1192	NLNESLIDL	0,6972
A02	HLA-A*02:59	1220	FIAGLIAIV	0,8207
A02	HLA-A*02:61	133	FQFCNDPFL	0,7104
A02	HLA-A*02:61	133	FQFCNDPFLGV	0,5205
A02	HLA-A*02:61	269	YLQPRTFLL	0,8955
A02	HLA-A*02:61	417	KIADYNYKL	0,9064
A02	HLA-A*02:61	424	KLPDDFTGCV	0,5686
A02	HLA-A*02:61	612	YQDVNCTEV	0,5256
A02	HLA-A*02:61	691	SIIAYTMSL	0,847
A02	HLA-A*02:61	712	IAIPTNFTI	0,5224
A02	HLA-A*02:61	718	FTISVTTEI	0,8184
A02	HLA-A*02:61	721	SVTTEILPV	0,5642
A02	HLA-A*02:61	821	LLFNKVTLA	0,5419
A02	HLA-A*02:61	894	LQIPFAMQM	0,6147
A02	HLA-A*02:61	976	VLNDILSRL	0,8346
A02	HLA-A*02:61	983	RLDKVEAEV	0,6135
A02	HLA-A*02:61	1048	HLMSFPQSA	0,5289
A02	HLA-A*02:61	1060	VVFLHVTYV	0,7813
A02	HLA-A*02:61	1136	TVYDPLQPEL	0,5968
A02	HLA-A*02:61	1220	FIAGLIAIV	0,81
A02	HLA-A*02:62	269	YLQPRTFLL	0,778
A02	HLA-A*02:62	417	KIADYNYKL	0,6875
A02	HLA-A*02:62	691	SIIAYTMSL	0,6033

A02	HLA-A*02:62	821	LLFNKVTLA	0,5193
A02	HLA-A*02:62	976	VLNDILSRL	0,681
A02	HLA-A*02:62	983	RLDKVEAEV	0,636
A02	HLA-A*02:62	1000	RLQSLQTYV	0,5378
A02	HLA-A*02:62	1060	VVFLHVTYV	0,6207
A02	HLA-A*02:62	1220	FIAGLIAIV	0,5269
A02	HLA-A*02:63	109	TLDSKTQSL	0,825
A02	HLA-A*02:63	133	FQFCNDPFL	0,5968
A02	HLA-A*02:63	269	YLQPRTFLL	0,9773
A02	HLA-A*02:63	386	KLNDLCFTNV	0,8261
A02	HLA-A*02:63	416	GKIADYNYKL	0,6474
A02	HLA-A*02:63	417	KIADYNYKL	0,9705
A02	HLA-A*02:63	424	KLPDDFTGCV	0,8161
A02	HLA-A*02:63	512	VLSFELLHA	0,5802
A02	HLA-A*02:63	515	FELLHAPATV	0,5863
A02	HLA-A*02:63	691	SIIAYTMSL	0,8402
A02	HLA-A*02:63	718	FTISVTTEI	0,6992
A02	HLA-A*02:63	821	LLFNKVTLA	0,8324
A02	HLA-A*02:63	857	GLTVLPPLL	0,7823
A02	HLA-A*02:63	869	MIAQYTSAL	0,7746
A02	HLA-A*02:63	915	VLYENQKLI	0,6825
A02	HLA-A*02:63	937	SLSSTASAL	0,6944
A02	HLA-A*02:63	947	KLQDVVNQNA	0,5801
A02	HLA-A*02:63	958	ALNTLVKQL	0,8332
A02	HLA-A*02:63	975	SVLNDILSRL	0,878
A02	HLA-A*02:63	976	VLNDILSRL	0,989
A02	HLA-A*02:63	983	RLDKVEAEV	0,8636
A02	HLA-A*02:63	1000	RLQSLQTYV	0,8614
A02	HLA-A*02:63	1047	YHLMSFPQSA	0,6493
A02	HLA-A*02:63	1048	HLMSFPQSA	0,8395
A02	HLA-A*02:63	1060	VVFLHVTYV	0,6976
A02	HLA-A*02:63	1095	FVSNGTHWFV	0,621
A02	HLA-A*02:63	1185	RLNEVAKNL	0,9052
A02	HLA-A*02:63	1192	NLNESLIDL	0,8555
A02	HLA-A*02:63	1196	SLIDLQEL	0,7114
A02	HLA-A*02:63	1220	FIAGLIAIV	0,8951
A02	HLA-A*02:66	109	TLDSKTQSL	0,6782
A02	HLA-A*02:66	133	FQFCNDPFL	0,518
A02	HLA-A*02:66	269	YLQPRTFLL	0,973
A02	HLA-A*02:66	386	KLNDLCFTNV	0,7108
A02	HLA-A*02:66	417	KIADYNYKL	0,909
A02	HLA-A*02:66	424	KLPDDFTGCV	0,5916
A02	HLA-A*02:66	515	FELLHAPATV	0,6033
A02	HLA-A*02:66	610	VLYQDVNCTEV	0,5092
A02	HLA-A*02:66	691	SIIAYTMSL	0,7995
A02	HLA-A*02:66	718	FTISVTTEI	0,5257
A02	HLA-A*02:66	821	LLFNKVTLA	0,7857
A02	HLA-A*02:66	857	GLTVLPPLL	0,6786
A02	HLA-A*02:66	915	VLYENQKLI	0,5378
A02	HLA-A*02:66	975	SVLNDILSRL	0,5124

A02	HLA-A*02:66	976	VLNDILSRL	0,9507
A02	HLA-A*02:66	983	RLDKVEAEV	0,8609
A02	HLA-A*02:66	1000	RLQSLQTYV	0,7431
A02	HLA-A*02:66	1047	YHLMSFPQSA	0,5452
A02	HLA-A*02:66	1048	HLMSFPQSA	0,7385
A02	HLA-A*02:66	1060	VVFLHVTYV	0,6948
A02	HLA-A*02:66	1185	RLNEVAKNL	0,6189
A02	HLA-A*02:66	1192	NLNESLIDL	0,6972
A02	HLA-A*02:66	1220	FIAGLIAIV	0,8207
A02	HLA-A*02:67	109	TLDSKTQSL	0,6782
A02	HLA-A*02:67	133	FQFCNDPFL	0,518
A02	HLA-A*02:67	269	YLQPRTFLL	0,973
A02	HLA-A*02:67	386	KLNDLCFTNV	0,7108
A02	HLA-A*02:67	417	KIADYNYKL	0,909
A02	HLA-A*02:67	424	KLPDDFTGCV	0,5916
A02	HLA-A*02:67	515	FELLHAPATV	0,6033
A02	HLA-A*02:67	610	VLYQDVNCTEV	0,5092
A02	HLA-A*02:67	691	SIIAYTMSL	0,7995
A02	HLA-A*02:67	718	FTISVTTEI	0,5257
A02	HLA-A*02:67	821	LLFNKVTLA	0,7857
A02	HLA-A*02:67	857	GLTVLPPLL	0,6786
A02	HLA-A*02:67	915	VLYENQKLI	0,5378
A02	HLA-A*02:67	975	SVLNDILSRL	0,5124
A02	HLA-A*02:67	976	VLNDILSRL	0,9507
A02	HLA-A*02:67	983	RLDKVEAEV	0,8609
A02	HLA-A*02:67	1000	RLQSLQTYV	0,7431
A02	HLA-A*02:67	1047	YHLMSFPQSA	0,5452
A02	HLA-A*02:67	1048	HLMSFPQSA	0,7385
A02	HLA-A*02:67	1060	VVFLHVTYV	0,6948
A02	HLA-A*02:67	1185	RLNEVAKNL	0,6189
A02	HLA-A*02:67	1192	NLNESLIDL	0,6972
A02	HLA-A*02:67	1220	FIAGLIAIV	0,8207
A02	HLA-A*02:68	109	TLDSKTQSL	0,6782
A02	HLA-A*02:68	133	FQFCNDPFL	0,518
A02	HLA-A*02:68	269	YLQPRTFLL	0,973
A02	HLA-A*02:68	386	KLNDLCFTNV	0,7108
A02	HLA-A*02:68	417	KIADYNYKL	0,909
A02	HLA-A*02:68	424	KLPDDFTGCV	0,5916
A02	HLA-A*02:68	515	FELLHAPATV	0,6033
A02	HLA-A*02:68	610	VLYQDVNCTEV	0,5092
A02	HLA-A*02:68	691	SIIAYTMSL	0,7995
A02	HLA-A*02:68	718	FTISVTTEI	0,5257
A02	HLA-A*02:68	821	LLFNKVTLA	0,7857
A02	HLA-A*02:68	857	GLTVLPPLL	0,6786
A02	HLA-A*02:68	915	VLYENQKLI	0,5378
A02	HLA-A*02:68	975	SVLNDILSRL	0,5124
A02	HLA-A*02:68	976	VLNDILSRL	0,9507
A02	HLA-A*02:68	983	RLDKVEAEV	0,8609
A02	HLA-A*02:68	1000	RLQSLQTYV	0,7431
A02	HLA-A*02:68	1047	YHLMSFPQSA	0,5452

A02	HLA-A*02:68	1048	HLMSFPQSA	0,7385
A02	HLA-A*02:68	1060	VVFLHVTYV	0,6948
A02	HLA-A*02:68	1185	RLNEVAKNL	0,6189
A02	HLA-A*02:68	1192	NLNESLIDL	0,6972
A02	HLA-A*02:68	1220	FIAGLIAIV	0,8207
A02	HLA-A*02:69	109	TLDSKTQSL	0,8988
A02	HLA-A*02:69	269	YLQPRTFLL	0,9932
A02	HLA-A*02:69	386	KLNDLCFTNV	0,8871
A02	HLA-A*02:69	417	KIADYNYKL	0,97
A02	HLA-A*02:69	424	KLPDDFTGCV	0,7417
A02	HLA-A*02:69	515	FELLHAPATV	0,7889
A02	HLA-A*02:69	610	VLYQDVNCTEV	0,6974
A02	HLA-A*02:69	691	SIIAYTMSL	0,9432
A02	HLA-A*02:69	718	FTISVTTEI	0,8216
A02	HLA-A*02:69	821	LLFNKVTLA	0,9443
A02	HLA-A*02:69	857	GLTVLPPLL	0,7856
A02	HLA-A*02:69	915	VLYENQKLI	0,8632
A02	HLA-A*02:69	958	ALNTLVKQL	0,8141
A02	HLA-A*02:69	975	SVLNDILSRL	0,7945
A02	HLA-A*02:69	976	VLNDILSRL	0,9917
A02	HLA-A*02:69	983	RLDKVEAEV	0,9677
A02	HLA-A*02:69	1000	RLQSLQTYV	0,9419
A02	HLA-A*02:69	1047	YHLMSFPQSA	0,7518
A02	HLA-A*02:69	1048	HLMSFPQSA	0,9136
A02	HLA-A*02:69	1060	VVFLHVTYV	0,8865
A02	HLA-A*02:69	1171	GINASVVNI	0,7552
A02	HLA-A*02:69	1185	RLNEVAKNL	0,9099
A02	HLA-A*02:69	1192	NLNESLIDL	0,8952
A02	HLA-A*02:69	1220	FIAGLIAIV	0,9533
A02	HLA-A*02:70	109	TLDSKTQSL	0,6782
A02	HLA-A*02:70	133	FQFCNDPFL	0,518
A02	HLA-A*02:70	269	YLQPRTFLL	0,973
A02	HLA-A*02:70	386	KLNDLCFTNV	0,7108
A02	HLA-A*02:70	417	KIADYNYKL	0,909
A02	HLA-A*02:70	424	KLPDDFTGCV	0,5916
A02	HLA-A*02:70	515	FELLHAPATV	0,6033
A02	HLA-A*02:70	610	VLYQDVNCTEV	0,5092
A02	HLA-A*02:70	691	SIIAYTMSL	0,7995
A02	HLA-A*02:70	718	FTISVTTEI	0,5257
A02	HLA-A*02:70	821	LLFNKVTLA	0,7857
A02	HLA-A*02:70	857	GLTVLPPLL	0,6786
A02	HLA-A*02:70	915	VLYENQKLI	0,5378
A02	HLA-A*02:70	975	SVLNDILSRL	0,5124
A02	HLA-A*02:70	976	VLNDILSRL	0,9507
A02	HLA-A*02:70	983	RLDKVEAEV	0,8609
A02	HLA-A*02:70	1000	RLQSLQTYV	0,7431
A02	HLA-A*02:70	1047	YHLMSFPQSA	0,5452
A02	HLA-A*02:70	1048	HLMSFPQSA	0,7385
A02	HLA-A*02:70	1060	VVFLHVTYV	0,6948
A02	HLA-A*02:70	1185	RLNEVAKNL	0,6189

A02	HLA-A*02:70	1192	NLNESLIDL	0,6972
A02	HLA-A*02:70	1220	FIAGLIAIV	0,8207
A02	HLA-A*02:71	109	TLDSKTQSL	0,6782
A02	HLA-A*02:71	133	FQFCNDPFL	0,518
A02	HLA-A*02:71	269	YLQPRTFLL	0,973
A02	HLA-A*02:71	386	KLNDLCFTNV	0,7108
A02	HLA-A*02:71	417	KIADYNYKL	0,909
A02	HLA-A*02:71	424	KLPDDFTGCV	0,5916
A02	HLA-A*02:71	515	FELLHAPATV	0,6033
A02	HLA-A*02:71	610	VLYQDVNCTEV	0,5092
A02	HLA-A*02:71	691	SIIAYTMSL	0,7995
A02	HLA-A*02:71	718	FTISVTTEI	0,5257
A02	HLA-A*02:71	821	LLFNKVTLA	0,7857
A02	HLA-A*02:71	857	GLTVLPPLL	0,6786
A02	HLA-A*02:71	915	VLYENQKLI	0,5378
A02	HLA-A*02:71	975	SVLNDILSRL	0,5124
A02	HLA-A*02:71	976	VLNDILSRL	0,9507
A02	HLA-A*02:71	983	RLDKVEAEV	0,8609
A02	HLA-A*02:71	1000	RLQSLQTYV	0,7431
A02	HLA-A*02:71	1047	YHLMSFPQSA	0,5452
A02	HLA-A*02:71	1048	HLMSFPQSA	0,7385
A02	HLA-A*02:71	1060	VVFLHVTYV	0,6948
A02	HLA-A*02:71	1185	RLNEVAKNL	0,6189
A02	HLA-A*02:71	1192	NLNESLIDL	0,6972
A02	HLA-A*02:71	1220	FIAGLIAIV	0,8207
A02	HLA-A*02:72	133	FQFCNDPFL	0,7104
A02	HLA-A*02:72	133	FQFCNDPFLGV	0,5205
A02	HLA-A*02:72	269	YLQPRTFLL	0,8955
A02	HLA-A*02:72	417	KIADYNYKL	0,9064
A02	HLA-A*02:72	424	KLPDDFTGCV	0,5686
A02	HLA-A*02:72	612	YQDVNCTEV	0,5256
A02	HLA-A*02:72	691	SIIAYTMSL	0,847
A02	HLA-A*02:72	712	IAIPTNFTI	0,5224
A02	HLA-A*02:72	718	FTISVTTEI	0,8184
A02	HLA-A*02:72	721	SVTTEILPV	0,5642
A02	HLA-A*02:72	821	LLFNKVTLA	0,5419
A02	HLA-A*02:72	894	LQIPFAMQM	0,6147
A02	HLA-A*02:72	976	VLNDILSRL	0,8346
A02	HLA-A*02:72	983	RLDKVEAEV	0,6135
A02	HLA-A*02:72	1048	HLMSFPQSA	0,5289
A02	HLA-A*02:72	1060	VVFLHVTYV	0,7813
A02	HLA-A*02:72	1136	TVYDPLQPEL	0,5968
A02	HLA-A*02:72	1220	FIAGLIAIV	0,81
A02	HLA-A*02:74	109	TLDSKTQSL	0,6782
A02	HLA-A*02:74	133	FQFCNDPFL	0,518
A02	HLA-A*02:74	269	YLQPRTFLL	0,973
A02	HLA-A*02:74	386	KLNDLCFTNV	0,7108
A02	HLA-A*02:74	417	KIADYNYKL	0,909
A02	HLA-A*02:74	424	KLPDDFTGCV	0,5916
A02	HLA-A*02:74	515	FELLHAPATV	0,6033

A02	HLA-A*02:74	610	VLYQDVNCTEV	0,5092
A02	HLA-A*02:74	691	SIIAYTMSL	0,7995
A02	HLA-A*02:74	718	FTISVTTEI	0,5257
A02	HLA-A*02:74	821	LLFNKVTLA	0,7857
A02	HLA-A*02:74	857	GLTVLPPLL	0,6786
A02	HLA-A*02:74	915	VLYENQKLI	0,5378
A02	HLA-A*02:74	975	SVLNDILSRL	0,5124
A02	HLA-A*02:74	976	VLNDILSRL	0,9507
A02	HLA-A*02:74	983	RLDKVEAEV	0,8609
A02	HLA-A*02:74	1000	RLQSLQTYV	0,7431
A02	HLA-A*02:74	1047	YHLMSFPQSA	0,5452
A02	HLA-A*02:74	1048	HLMSFPQSA	0,7385
A02	HLA-A*02:74	1060	VVFLHVTYV	0,6948
A02	HLA-A*02:74	1185	RLNEVAKNL	0,6189
A02	HLA-A*02:74	1192	NLNESLIDL	0,6972
A02	HLA-A*02:74	1220	FIAGLIAIV	0,8207
A02	HLA-A*02:75	109	TLDSKTQSL	0,6782
A02	HLA-A*02:75	133	FQFCNDPFL	0,518
A02	HLA-A*02:75	269	YLQPRTFLL	0,973
A02	HLA-A*02:75	386	KLNDLCFTNV	0,7108
A02	HLA-A*02:75	417	KIADYNYKL	0,909
A02	HLA-A*02:75	424	KLPDDFTGCV	0,5916
A02	HLA-A*02:75	515	FELLHAPATV	0,6033
A02	HLA-A*02:75	610	VLYQDVNCTEV	0,5092
A02	HLA-A*02:75	691	SIIAYTMSL	0,7995
A02	HLA-A*02:75	718	FTISVTTEI	0,5257
A02	HLA-A*02:75	821	LLFNKVTLA	0,7857
A02	HLA-A*02:75	857	GLTVLPPLL	0,6786
A02	HLA-A*02:75	915	VLYENQKLI	0,5378
A02	HLA-A*02:75	975	SVLNDILSRL	0,5124
A02	HLA-A*02:75	976	VLNDILSRL	0,9507
A02	HLA-A*02:75	983	RLDKVEAEV	0,8609
A02	HLA-A*02:75	1000	RLQSLQTYV	0,7431
A02	HLA-A*02:75	1047	YHLMSFPQSA	0,5452
A02	HLA-A*02:75	1048	HLMSFPQSA	0,7385
A02	HLA-A*02:75	1060	VVFLHVTYV	0,6948
A02	HLA-A*02:75	1185	RLNEVAKNL	0,6189
A02	HLA-A*02:75	1192	NLNESLIDL	0,6972
A02	HLA-A*02:75	1220	FIAGLIAIV	0,8207
A02	HLA-A*02:77	109	TLDSKTQSL	0,6782
A02	HLA-A*02:77	133	FQFCNDPFL	0,518
A02	HLA-A*02:77	269	YLQPRTFLL	0,973
A02	HLA-A*02:77	386	KLNDLCFTNV	0,7108
A02	HLA-A*02:77	417	KIADYNYKL	0,909
A02	HLA-A*02:77	424	KLPDDFTGCV	0,5916
A02	HLA-A*02:77	515	FELLHAPATV	0,6033
A02	HLA-A*02:77	610	VLYQDVNCTEV	0,5092
A02	HLA-A*02:77	691	SIIAYTMSL	0,7995
A02	HLA-A*02:77	718	FTISVTTEI	0,5257
A02	HLA-A*02:77	821	LLFNKVTLA	0,7857

A02	HLA-A*02:77	857	GLTVLPPLL	0,6786
A02	HLA-A*02:77	915	VLYENQKLI	0,5378
A02	HLA-A*02:77	975	SVLNDILSRL	0,5124
A02	HLA-A*02:77	976	VLNDILSRL	0,9507
A02	HLA-A*02:77	983	RLDKVEAEV	0,8609
A02	HLA-A*02:77	1000	RLQSLQTYV	0,7431
A02	HLA-A*02:77	1047	YHLMSFPQSA	0,5452
A02	HLA-A*02:77	1048	HLMSFPQSA	0,7385
A02	HLA-A*02:77	1060	VVFLHVTYV	0,6948
A02	HLA-A*02:77	1185	RLNEVAKNL	0,6189
A02	HLA-A*02:77	1192	NLNESLIDL	0,6972
A02	HLA-A*02:77	1220	FIAGLIAIV	0,8207
A02	HLA-A*02:78	28	YTNSFTRGV	0,6301
A02	HLA-A*02:78	62	VTWFHAIHV	0,7122
A02	HLA-A*02:78	269	YLQPRTFLL	0,5176
A02	HLA-A*02:78	417	KIADYNYKL	0,8511
A02	HLA-A*02:78	634	RVYSTGSNV	0,6719
A02	HLA-A*02:78	691	SIIAYTMSL	0,7755
A02	HLA-A*02:78	712	IAIPTNFTI	0,5958
A02	HLA-A*02:78	718	FTISVTTEI	0,6858
A02	HLA-A*02:78	721	SVTTEILPV	0,5092
A02	HLA-A*02:78	777	NTQEVFAQV	0,5878
A02	HLA-A*02:78	1060	VVFLHVTYV	0,7321
A02	HLA-A*02:78	1136	TVYDPLQPEL	0,5843
A02	HLA-A*02:78	1171	GINASVVNI	0,5371
A02	HLA-A*02:79	133	FQFCNDPFL	0,7104
A02	HLA-A*02:79	133	FQFCNDPFLGV	0,5205
A02	HLA-A*02:79	269	YLQPRTFLL	0,8955
A02	HLA-A*02:79	417	KIADYNYKL	0,9064
A02	HLA-A*02:79	424	KLPDDFTGCV	0,5686
A02	HLA-A*02:79	612	YQDVNCTEV	0,5256
A02	HLA-A*02:79	691	SIIAYTMSL	0,847
A02	HLA-A*02:79	712	IAIPTNFTI	0,5224
A02	HLA-A*02:79	718	FTISVTTEI	0,8184
A02	HLA-A*02:79	721	SVTTEILPV	0,5642
A02	HLA-A*02:79	821	LLFNKVTLA	0,5419
A02	HLA-A*02:79	894	LQIPFAMQM	0,6147
A02	HLA-A*02:79	976	VLNDILSRL	0,8346
A02	HLA-A*02:79	983	RLDKVEAEV	0,6135
A02	HLA-A*02:79	1048	HLMSFPQSA	0,5289
A02	HLA-A*02:79	1060	VVFLHVTYV	0,7813
A02	HLA-A*02:79	1136	TVYDPLQPEL	0,5968
A02	HLA-A*02:79	1220	FIAGLIAIV	0,81
A02	HLA-A*02:85	109	TLDSKTQSL	0,6782
A02	HLA-A*02:85	133	FQFCNDPFL	0,518
A02	HLA-A*02:85	269	YLQPRTFLL	0,973
A02	HLA-A*02:85	386	KLNDLCFTNV	0,7108
A02	HLA-A*02:85	417	KIADYNYKL	0,909
A02	HLA-A*02:85	424	KLPDDFTGCV	0,5916
A02	HLA-A*02:85	515	FELLHAPATV	0,6033

A02	HLA-A*02:85	610	VLYQDVNCTEV	0,5092
A02	HLA-A*02:85	691	SIIAYTMSL	0,7995
A02	HLA-A*02:85	718	FTISVTTEI	0,5257
A02	HLA-A*02:85	821	LLFNKVTLA	0,7857
A02	HLA-A*02:85	857	GLTVLPPLL	0,6786
A02	HLA-A*02:85	915	VLYENQKLI	0,5378
A02	HLA-A*02:85	975	SVLNDILSRL	0,5124
A02	HLA-A*02:85	976	VLNDILSRL	0,9507
A02	HLA-A*02:85	983	RLDKVEAEV	0,8609
A02	HLA-A*02:85	1000	RLQSLQTYV	0,7431
A02	HLA-A*02:85	1047	YHLMSFPQSA	0,5452
A02	HLA-A*02:85	1048	HLMSFPQSA	0,7385
A02	HLA-A*02:85	1060	VVFLHVTYV	0,6948
A02	HLA-A*02:85	1185	RLNEVAKNL	0,6189
A02	HLA-A*02:85	1192	NLNESLIDL	0,6972
A02	HLA-A*02:85	1220	FIAGLIAIV	0,8207
A02	HLA-A*02:86	109	TLDSKTQSL	0,6782
A02	HLA-A*02:86	133	FQFCNDPFL	0,518
A02	HLA-A*02:86	269	YLQPRTFLL	0,973
A02	HLA-A*02:86	386	KLNDLCFTNV	0,7108
A02	HLA-A*02:86	417	KIADYNYKL	0,909
A02	HLA-A*02:86	424	KLPDDFTGCV	0,5916
A02	HLA-A*02:86	515	FELLHAPATV	0,6033
A02	HLA-A*02:86	610	VLYQDVNCTEV	0,5092
A02	HLA-A*02:86	691	SIIAYTMSL	0,7995
A02	HLA-A*02:86	718	FTISVTTEI	0,5257
A02	HLA-A*02:86	821	LLFNKVTLA	0,7857
A02	HLA-A*02:86	857	GLTVLPPLL	0,6786
A02	HLA-A*02:86	915	VLYENQKLI	0,5378
A02	HLA-A*02:86	975	SVLNDILSRL	0,5124
A02	HLA-A*02:86	976	VLNDILSRL	0,9507
A02	HLA-A*02:86	983	RLDKVEAEV	0,8609
A02	HLA-A*02:86	1000	RLQSLQTYV	0,7431
A02	HLA-A*02:86	1047	YHLMSFPQSA	0,5452
A02	HLA-A*02:86	1048	HLMSFPQSA	0,7385
A02	HLA-A*02:86	1060	VVFLHVTYV	0,6948
A02	HLA-A*02:86	1185	RLNEVAKNL	0,6189
A02	HLA-A*02:86	1192	NLNESLIDL	0,6972
A02	HLA-A*02:86	1220	FIAGLIAIV	0,8207
A02	HLA-A*68:02	28	YTNSFTRGV	0,845
A02	HLA-A*68:02	122	NATNVVIKV	0,7315
A02	HLA-A*68:02	258	WTAGAAAYYV	0,5761
A02	HLA-A*68:02	259	TAGAAAYYV	0,5379
A02	HLA-A*68:02	340	EVFNATRFA	0,7317
A02	HLA-A*68:02	394	NVYADSFVI	0,6993
A02	HLA-A*68:02	495	YGFQPTNGV	0,5183
A02	HLA-A*68:02	568	DIADTTDAV	0,7805
A02	HLA-A*68:02	603	NTSNQVAVL	0,5558
A02	HLA-A*68:02	691	SIIAYTMSL	0,6292
A02	HLA-A*68:02	704	SVAYSNNSI	0,7306

A02	HLA-A*68:02	717	NFTISVTTEI	0,6193
A02	HLA-A*68:02	718	FTISVTTEI	0,9208
A02	HLA-A*68:02	734	TSVDCTMYI	0,5115
A02	HLA-A*68:02	777	NTQEVFAQV	0,9221
A02	HLA-A*68:02	780	EVFAQVKQI	0,6531
A02	HLA-A*68:02	869	MIAQYTSAL	0,6202
A02	HLA-A*68:02	886	WTFGAGAAL	0,732
A02	HLA-A*68:02	907	NGIGVTQNV	0,5186
A02	HLA-A*68:02	940	STASALGKL	0,5142
A02	HLA-A*68:02	1060	VVFLHVTYV	0,6649
A02	HLA-A*68:02	1095	FVSNGTHWFV	0,5041
A02	HLA-A*68:02	1128	VVIGIVNNTV	0,5513
A02	HLA-A*68:02	1136	TVYDPLQPEL	0,6066
A02	HLA-A*68:02	1168	DISGINASV	0,8721
A02	HLA-A*68:02	1220	FIAGLIAIV	0,636
A02	HLA-A*68:27	28	YTNSFTRGV	0,845
A02	HLA-A*68:27	122	NATNVVIKV	0,7315
A02	HLA-A*68:27	258	WTAGAAAYYV	0,5761
A02	HLA-A*68:27	259	TAGAAAYYV	0,5379
A02	HLA-A*68:27	340	EVFNATRFA	0,7317
A02	HLA-A*68:27	394	NVYADSFVI	0,6993
A02	HLA-A*68:27	495	YGFQPTNGV	0,5183
A02	HLA-A*68:27	568	DIADTTDAV	0,7805
A02	HLA-A*68:27	603	NTSNQVAVL	0,5558
A02	HLA-A*68:27	691	SIIAYTMSL	0,6292
A02	HLA-A*68:27	704	SVAYSNNSI	0,7306
A02	HLA-A*68:27	717	NFTISVTTEI	0,6193
A02	HLA-A*68:27	718	FTISVTTEI	0,9208
A02	HLA-A*68:27	734	TSVDCTMYI	0,5115
A02	HLA-A*68:27	777	NTQEVFAQV	0,9221
A02	HLA-A*68:27	780	EVFAQVKQI	0,6531
A02	HLA-A*68:27	869	MIAQYTSAL	0,6202
A02	HLA-A*68:27	886	WTFGAGAAL	0,732
A02	HLA-A*68:27	907	NGIGVTQNV	0,5186
A02	HLA-A*68:27	940	STASALGKL	0,5142
A02	HLA-A*68:27	1060	VVFLHVTYV	0,6649
A02	HLA-A*68:27	1095	FVSNGTHWFV	0,5041
A02	HLA-A*68:27	1128	VVIGIVNNTV	0,5513
A02	HLA-A*68:27	1136	TVYDPLQPEL	0,6066
A02	HLA-A*68:27	1168	DISGINASV	0,8721
A02	HLA-A*68:27	1220	FIAGLIAIV	0,636
A02	HLA-A*68:28	28	YTNSFTRGV	0,5476
A02	HLA-A*68:28	568	DIADTTDAV	0,6854
A02	HLA-A*68:28	718	FTISVTTEI	0,6638
A02	HLA-A*68:28	777	NTQEVFAQV	0,7667
A02	HLA-A*68:28	1168	DISGINASV	0,5957
A02	HLA-A*69:01	28	YTNSFTRGV	0,6754
A02	HLA-A*69:01	62	VTWFHAIHV	0,5639
A02	HLA-A*69:01	122	NATNVVIKV	0,6388
A02	HLA-A*69:01	394	NVYADSFVI	0,702

A02	HLA-A*69:01	568	DIADTTDAV	0,62
A02	HLA-A*69:01	691	SIIAYTMSL	0,6455
A02	HLA-A*69:01	718	FTISVTTEI	0,8233
A02	HLA-A*69:01	777	NTQEVFAQV	0,8811
A02	HLA-A*69:01	780	EVFAQVKQI	0,5161
A02	HLA-A*69:01	886	WTFGAGAAL	0,6113
A02	HLA-A*69:01	1060	VVFLHVTYV	0,6659
A02	HLA-A*69:01	1136	TVYDPLQPEL	0,6066
A02	HLA-A*69:01	1168	DISGINASV	0,7179
A02	HLA-A*69:01	1220	FIAGLIAIV	0,5133
A03	HLA-A*03:01	35	GVYYPDKVFR	0,568
A03	HLA-A*03:01	41	KVFRSSVLH	0,6944
A03	HLA-A*03:01	89	GVYFASTEK	0,9487
A03	HLA-A*03:01	142	GVYYHKNNK	0,8871
A03	HLA-A*03:01	269	YLQPRTFLLK	0,5577
A03	HLA-A*03:01	302	TLKSFTVEK	0,8988
A03	HLA-A*03:01	311	GIYQTSNFR	0,6304
A03	HLA-A*03:01	349	SVYAWNRKR	0,6333
A03	HLA-A*03:01	367	VLYNSASFSTFK	0,5358
A03	HLA-A*03:01	378	KCYGVSPTK	0,5631
A03	HLA-A*03:01	408	RQIAPGQTGK	0,8957
A03	HLA-A*03:01	409	QIAPGQTGK	0,6982
A03	HLA-A*03:01	454	RLFRKSNLK	0,9589
A03	HLA-A*03:01	529	KSTNLVKNK	0,5122
A03	HLA-A*03:01	724	TEILPVSMTK	0,6838
A03	HLA-A*03:01	786	KQIYKTPPIK	0,8503
A03	HLA-A*03:01	787	QIYKTPPIK	0,8934
A03	HLA-A*03:01	805	ILPDPSKPSK	0,5588
A03	HLA-A*03:01	826	VTLADAGFIK	0,5395
A03	HLA-A*03:01	827	TLADAGFIK	0,7267
A03	HLA-A*03:01	924	ANQFNSAIGK	0,5091
A03	HLA-A*03:01	939	SSTASALGK	0,5175
A03	HLA-A*03:01	1019	RASANLAATK	0,5521
A03	HLA-A*03:01	1020	ASANLAATK	0,7894
A03	HLA-A*03:01	1064	HVTYVPAQEK	0,6951
A03	HLA-A*03:01	1065	VTYVPAQEK	0,9087
A03	HLA-A*03:01	1099	GTHWFVTQR	0,5011
A03	HLA-A*03:01	1196	SLIDLQELGK	0,6419
A03	HLA-A*03:01	1264	VLKGVKLHY	0,6718
A03	HLA-A*03:02	35	GVYYPDKVFR	0,6162
A03	HLA-A*03:02	89	GVYFASTEK	0,8282
A03	HLA-A*03:02	142	GVYYHKNNK	0,6008
A03	HLA-A*03:02	269	YLQPRTFLLK	0,5505
A03	HLA-A*03:02	292	ALDPLSETK	0,7332
A03	HLA-A*03:02	302	TLKSFTVEK	0,8355
A03	HLA-A*03:02	311	GIYQTSNFR	0,6485
A03	HLA-A*03:02	349	SVYAWNRKR	0,5515
A03	HLA-A*03:02	408	RQIAPGQTGK	0,607
A03	HLA-A*03:02	454	RLFRKSNLK	0,8224
A03	HLA-A*03:02	724	TEILPVSMTK	0,5896

A03	HLA-A*03:02	725	EILPVSMTK	0,5252
A03	HLA-A*03:02	787	QIYKTPPIK	0,6603
A03	HLA-A*03:02	805	ILPDPSKPSK	0,52
A03	HLA-A*03:02	826	VTLADAGFIK	0,5723
A03	HLA-A*03:02	827	TLADAGFIK	0,8003
A03	HLA-A*03:02	975	SVLNDILSR	0,6428
A03	HLA-A*03:02	1020	ASANLAATK	0,6797
A03	HLA-A*03:02	1065	VTYVPAQEK	0,744
A03	HLA-A*03:02	1099	GTHWFVTQR	0,5647
A03	HLA-A*03:02	1196	SLIDLQELGK	0,5484
A03	HLA-A*03:04	35	GVYYPDKVFR	0,568
A03	HLA-A*03:04	41	KVFRSSVLH	0,6944
A03	HLA-A*03:04	89	GVYFASTEK	0,9487
A03	HLA-A*03:04	142	GVYYHKNNK	0,8871
A03	HLA-A*03:04	269	YLQPRTFLLK	0,5577
A03	HLA-A*03:04	302	TLKSFTVEK	0,8988
A03	HLA-A*03:04	311	GIYQTSNFR	0,6304
A03	HLA-A*03:04	349	SVYAWNRKR	0,6333
A03	HLA-A*03:04	367	VLYNSASFSTFK	0,5358
A03	HLA-A*03:04	378	KCYGVSPTK	0,5631
A03	HLA-A*03:04	408	RQIAPGQTGK	0,8957
A03	HLA-A*03:04	409	QIAPGQTGK	0,6982
A03	HLA-A*03:04	454	RLFRKSNLK	0,9589
A03	HLA-A*03:04	529	KSTNLVKNK	0,5122
A03	HLA-A*03:04	724	TEILPVSMTK	0,6838
A03	HLA-A*03:04	786	KQIYKTPPIK	0,8503
A03	HLA-A*03:04	787	QIYKTPPIK	0,8934
A03	HLA-A*03:04	805	ILPDPSKPSK	0,5588
A03	HLA-A*03:04	826	VTLADAGFIK	0,5395
A03	HLA-A*03:04	827	TLADAGFIK	0,7267
A03	HLA-A*03:04	924	ANQFNSAIGK	0,5091
A03	HLA-A*03:04	939	SSTASALGK	0,5175
A03	HLA-A*03:04	1019	RASANLAATK	0,5521
A03	HLA-A*03:04	1020	ASANLAATK	0,7894
A03	HLA-A*03:04	1064	HVTYVPAQEK	0,6951
A03	HLA-A*03:04	1065	VTYVPAQEK	0,9087
A03	HLA-A*03:04	1099	GTHWFVTQR	0,5011
A03	HLA-A*03:04	1196	SLIDLQELGK	0,6419
A03	HLA-A*03:04	1264	VLKGVKLHY	0,6718
A03	HLA-A*03:05	35	GVYYPDKVFR	0,568
A03	HLA-A*03:05	41	KVFRSSVLH	0,6944
A03	HLA-A*03:05	89	GVYFASTEK	0,9487
A03	HLA-A*03:05	142	GVYYHKNNK	0,8871
A03	HLA-A*03:05	269	YLQPRTFLLK	0,5577
A03	HLA-A*03:05	302	TLKSFTVEK	0,8988
A03	HLA-A*03:05	311	GIYQTSNFR	0,6304
A03	HLA-A*03:05	349	SVYAWNRKR	0,6333
A03	HLA-A*03:05	367	VLYNSASFSTFK	0,5358
A03	HLA-A*03:05	378	KCYGVSPTK	0,5631
A03	HLA-A*03:05	408	RQIAPGQTGK	0,8957

A03	HLA-A*03:05	409	QIAPGQTGK	0,6982
A03	HLA-A*03:05	454	RLFRKSNLK	0,9589
A03	HLA-A*03:05	529	KSTNLVKNK	0,5122
A03	HLA-A*03:05	724	TEILPVSMTK	0,6838
A03	HLA-A*03:05	786	KQIYKTPPIK	0,8503
A03	HLA-A*03:05	787	QIYKTPPIK	0,8934
A03	HLA-A*03:05	805	ILPDPSKPSK	0,5588
A03	HLA-A*03:05	826	VTLADAGFIK	0,5395
A03	HLA-A*03:05	827	TLADAGFIK	0,7267
A03	HLA-A*03:05	924	ANQFNSAIGK	0,5091
A03	HLA-A*03:05	939	SSTASALGK	0,5175
A03	HLA-A*03:05	1019	RASANLAATK	0,5521
A03	HLA-A*03:05	1020	ASANLAATK	0,7894
A03	HLA-A*03:05	1064	HVTYVPAQEK	0,6951
A03	HLA-A*03:05	1065	VTYVPAQEK	0,9087
A03	HLA-A*03:05	1099	GTHWFVTQR	0,5011
A03	HLA-A*03:05	1196	SLIDLQELGK	0,6419
A03	HLA-A*03:05	1264	VLKGVKLHY	0,6718
A03	HLA-A*03:06	35	GVYYPDKVFR	0,568
A03	HLA-A*03:06	41	KVFRSSVLH	0,6944
A03	HLA-A*03:06	89	GVYFASTEK	0,9487
A03	HLA-A*03:06	142	GVYYHKNNK	0,8871
A03	HLA-A*03:06	269	YLQPRTFLLK	0,5577
A03	HLA-A*03:06	302	TLKSFTVEK	0,8988
A03	HLA-A*03:06	311	GIYQTSNFR	0,6304
A03	HLA-A*03:06	349	SVYAWNRKR	0,6333
A03	HLA-A*03:06	367	VLYNSASFSTFK	0,5358
A03	HLA-A*03:06	378	KCYGVSPTK	0,5631
A03	HLA-A*03:06	408	RQIAPGQTGK	0,8957
A03	HLA-A*03:06	409	QIAPGQTGK	0,6982
A03	HLA-A*03:06	454	RLFRKSNLK	0,9589
A03	HLA-A*03:06	529	KSTNLVKNK	0,5122
A03	HLA-A*03:06	724	TEILPVSMTK	0,6838
A03	HLA-A*03:06	786	KQIYKTPPIK	0,8503
A03	HLA-A*03:06	787	QIYKTPPIK	0,8934
A03	HLA-A*03:06	805	ILPDPSKPSK	0,5588
A03	HLA-A*03:06	826	VTLADAGFIK	0,5395
A03	HLA-A*03:06	827	TLADAGFIK	0,7267
A03	HLA-A*03:06	924	ANQFNSAIGK	0,5091
A03	HLA-A*03:06	939	SSTASALGK	0,5175
A03	HLA-A*03:06	1019	RASANLAATK	0,5521
A03	HLA-A*03:06	1020	ASANLAATK	0,7894
A03	HLA-A*03:06	1064	HVTYVPAQEK	0,6951
A03	HLA-A*03:06	1065	VTYVPAQEK	0,9087
A03	HLA-A*03:06	1099	GTHWFVTQR	0,5011
A03	HLA-A*03:06	1196	SLIDLQELGK	0,6419
A03	HLA-A*03:06	1264	VLKGVKLHY	0,6718
A03	HLA-A*03:07	35	GVYYPDKVFR	0,7348
A03	HLA-A*03:07	89	GVYFASTEK	0,8648
A03	HLA-A*03:07	142	GVYYHKNNK	0,6702

A03	HLA-A*03:07	292	ALDPLSETK	0,6955
A03	HLA-A*03:07	302	TLKSFTVEK	0,8407
A03	HLA-A*03:07	311	GIYQTSNFR	0,7033
A03	HLA-A*03:07	349	SVYAWNRKR	0,6804
A03	HLA-A*03:07	370	NSASFSTFK	0,5695
A03	HLA-A*03:07	408	RQIAPGQTGK	0,6507
A03	HLA-A*03:07	454	RLFRKSNLK	0,8435
A03	HLA-A*03:07	458	KSNLKPFER	0,5539
A03	HLA-A*03:07	724	TEILPVSMTK	0,6479
A03	HLA-A*03:07	725	EILPVSMTK	0,6008
A03	HLA-A*03:07	786	KQIYKTPPIK	0,5627
A03	HLA-A*03:07	787	QIYKTPPIK	0,757
A03	HLA-A*03:07	805	ILPDPSKPSK	0,5158
A03	HLA-A*03:07	826	VTLADAGFIK	0,6103
A03	HLA-A*03:07	827	TLADAGFIK	0,8004
A03	HLA-A*03:07	975	SVLNDILSR	0,744
A03	HLA-A*03:07	1020	ASANLAATK	0,7263
A03	HLA-A*03:07	1064	HVTYVPAQEK	0,5071
A03	HLA-A*03:07	1065	VTYVPAQEK	0,8254
A03	HLA-A*03:07	1099	GTHWFVTQR	0,6685
A03	HLA-A*03:07	1196	SLIDLQELGK	0,6149
A03	HLA-A*03:08	35	GVYYPDKVFR	0,5777
A03	HLA-A*03:08	41	KVFRSSVLH	0,5336
A03	HLA-A*03:08	89	GVYFASTEK	0,8234
A03	HLA-A*03:08	142	GVYYHKNNK	0,7338
A03	HLA-A*03:08	302	TLKSFTVEK	0,7823
A03	HLA-A*03:08	311	GIYQTSNFR	0,623
A03	HLA-A*03:08	349	SVYAWNRKR	0,6585
A03	HLA-A*03:08	408	RQIAPGQTGK	0,7314
A03	HLA-A*03:08	454	RLFRKSNLK	0,9008
A03	HLA-A*03:08	786	KQIYKTPPIK	0,6142
A03	HLA-A*03:08	787	QIYKTPPIK	0,679
A03	HLA-A*03:08	1020	ASANLAATK	0,5793
A03	HLA-A*03:08	1065	VTYVPAQEK	0,7028
A03	HLA-A*03:08	1099	GTHWFVTQR	0,5336
A03	HLA-A*03:10	35	GVYYPDKVFR	0,6162
A03	HLA-A*03:10	89	GVYFASTEK	0,8282
A03	HLA-A*03:10	142	GVYYHKNNK	0,6008
A03	HLA-A*03:10	269	YLQPRTFLLK	0,5505
A03	HLA-A*03:10	292	ALDPLSETK	0,7332
A03	HLA-A*03:10	302	TLKSFTVEK	0,8355
A03	HLA-A*03:10	311	GIYQTSNFR	0,6485
A03	HLA-A*03:10	349	SVYAWNRKR	0,5515
A03	HLA-A*03:10	408	RQIAPGQTGK	0,607
A03	HLA-A*03:10	454	RLFRKSNLK	0,8224
A03	HLA-A*03:10	724	TEILPVSMTK	0,5896
A03	HLA-A*03:10	725	EILPVSMTK	0,5252
A03	HLA-A*03:10	787	QIYKTPPIK	0,6603
A03	HLA-A*03:10	805	ILPDPSKPSK	0,52
A03	HLA-A*03:10	826	VTLADAGFIK	0,5723

A03	HLA-A*03:10	827	TLADAGFIK	0,8003
A03	HLA-A*03:10	975	SVLNDILSR	0,6428
A03	HLA-A*03:10	1020	ASANLAATK	0,6797
A03	HLA-A*03:10	1065	VTYVPAQEK	0,744
A03	HLA-A*03:10	1099	GTHWFVTQR	0,5647
A03	HLA-A*03:10	1196	SLIDLQELGK	0,5484
A03	HLA-A*03:12	35	GVYYPDKVFR	0,6548
A03	HLA-A*03:12	41	KVFRSSVLH	0,7788
A03	HLA-A*03:12	69	HVSGTNGTK	0,5995
A03	HLA-A*03:12	89	GVYFASTEK	0,9495
A03	HLA-A*03:12	142	GVYYHKNNK	0,8976
A03	HLA-A*03:12	302	TLKSFTVEK	0,8641
A03	HLA-A*03:12	311	GIYQTSNFR	0,6653
A03	HLA-A*03:12	348	ASVYAWNRK	0,6546
A03	HLA-A*03:12	349	SVYAWNRKR	0,7468
A03	HLA-A*03:12	370	NSASFSTFK	0,6426
A03	HLA-A*03:12	375	STFKCYGVSPTK	0,5581
A03	HLA-A*03:12	378	KCYGVSPTK	0,6783
A03	HLA-A*03:12	408	RQIAPGQTGK	0,8931
A03	HLA-A*03:12	409	QIAPGQTGK	0,7544
A03	HLA-A*03:12	454	RLFRKSNLK	0,9223
A03	HLA-A*03:12	458	KSNLKPFER	0,5408
A03	HLA-A*03:12	529	KSTNLVKNK	0,7666
A03	HLA-A*03:12	550	GVLTESNKK	0,6481
A03	HLA-A*03:12	724	TEILPVSMTK	0,6899
A03	HLA-A*03:12	725	EILPVSMTK	0,7052
A03	HLA-A*03:12	786	KQIYKTPPIK	0,7729
A03	HLA-A*03:12	787	QIYKTPPIK	0,8972
A03	HLA-A*03:12	803	SQILPDPSK	0,5075
A03	HLA-A*03:12	826	VTLADAGFIK	0,5973
A03	HLA-A*03:12	827	TLADAGFIK	0,6931
A03	HLA-A*03:12	845	AARDLICAQK	0,5375
A03	HLA-A*03:12	924	ANQFNSAIGK	0,679
A03	HLA-A*03:12	925	NQFNSAIGK	0,6061
A03	HLA-A*03:12	939	SSTASALGK	0,7158
A03	HLA-A*03:12	956	AQALNTLVK	0,5799
A03	HLA-A*03:12	975	SVLNDILSR	0,7042
A03	HLA-A*03:12	1019	RASANLAATK	0,6895
A03	HLA-A*03:12	1020	ASANLAATK	0,8887
A03	HLA-A*03:12	1064	HVTYVPAQEK	0,7577
A03	HLA-A*03:12	1065	VTYVPAQEK	0,9408
A03	HLA-A*03:12	1099	GTHWFVTQR	0,7035
A03	HLA-A*03:12	1196	SLIDLQELGK	0,5515
A03	HLA-A*03:12	1264	VLKGVKLHY	0,6239
A03	HLA-A*03:13	35	GVYYPDKVFR	0,568
A03	HLA-A*03:13	41	KVFRSSVLH	0,6944
A03	HLA-A*03:13	89	GVYFASTEK	0,9487
A03	HLA-A*03:13	142	GVYYHKNNK	0,8871
A03	HLA-A*03:13	269	YLQPRTFLLK	0,5577
A03	HLA-A*03:13	302	TLKSFTVEK	0,8988

A03	HLA-A*03:13	311	GIYQTSNFR	0,6304
A03	HLA-A*03:13	349	SVYAWNRKR	0,6333
A03	HLA-A*03:13	367	VLYNSASFSTFK	0,5358
A03	HLA-A*03:13	378	KCYGVSPTK	0,5631
A03	HLA-A*03:13	408	RQIAPGQTGK	0,8957
A03	HLA-A*03:13	409	QIAPGQTGK	0,6982
A03	HLA-A*03:13	454	RLFRKSNLK	0,9589
A03	HLA-A*03:13	529	KSTNLVKNK	0,5122
A03	HLA-A*03:13	724	TEILPVSMTK	0,6838
A03	HLA-A*03:13	786	KQIYKTPPIK	0,8503
A03	HLA-A*03:13	787	QIYKTPPIK	0,8934
A03	HLA-A*03:13	805	ILPDPSKPSK	0,5588
A03	HLA-A*03:13	826	VTLADAGFIK	0,5395
A03	HLA-A*03:13	827	TLADAGFIK	0,7267
A03	HLA-A*03:13	924	ANQFNSAIGK	0,5091
A03	HLA-A*03:13	939	SSTASALGK	0,5175
A03	HLA-A*03:13	1019	RASANLAATK	0,5521
A03	HLA-A*03:13	1020	ASANLAATK	0,7894
A03	HLA-A*03:13	1064	HVTYVPAQEK	0,6951
A03	HLA-A*03:13	1065	VTYVPAQEK	0,9087
A03	HLA-A*03:13	1099	GTHWFVTQR	0,5011
A03	HLA-A*03:13	1196	SLIDLQELGK	0,6419
A03	HLA-A*03:13	1264	VLKGVKLHY	0,6718
A03	HLA-A*03:14	35	GVYYPDKVFR	0,568
A03	HLA-A*03:14	41	KVFRSSVLH	0,6944
A03	HLA-A*03:14	89	GVYFASTEK	0,9487
A03	HLA-A*03:14	142	GVYYHKNNK	0,8871
A03	HLA-A*03:14	269	YLQPRTFLLK	0,5577
A03	HLA-A*03:14	302	TLKSFTVEK	0,8988
A03	HLA-A*03:14	311	GIYQTSNFR	0,6304
A03	HLA-A*03:14	349	SVYAWNRKR	0,6333
A03	HLA-A*03:14	367	VLYNSASFSTFK	0,5358
A03	HLA-A*03:14	378	KCYGVSPTK	0,5631
A03	HLA-A*03:14	408	RQIAPGQTGK	0,8957
A03	HLA-A*03:14	409	QIAPGQTGK	0,6982
A03	HLA-A*03:14	454	RLFRKSNLK	0,9589
A03	HLA-A*03:14	529	KSTNLVKNK	0,5122
A03	HLA-A*03:14	724	TEILPVSMTK	0,6838
A03	HLA-A*03:14	786	KQIYKTPPIK	0,8503
A03	HLA-A*03:14	787	QIYKTPPIK	0,8934
A03	HLA-A*03:14	805	ILPDPSKPSK	0,5588
A03	HLA-A*03:14	826	VTLADAGFIK	0,5395
A03	HLA-A*03:14	827	TLADAGFIK	0,7267
A03	HLA-A*03:14	924	ANQFNSAIGK	0,5091
A03	HLA-A*03:14	939	SSTASALGK	0,5175
A03	HLA-A*03:14	1019	RASANLAATK	0,5521
A03	HLA-A*03:14	1020	ASANLAATK	0,7894
A03	HLA-A*03:14	1064	HVTYVPAQEK	0,6951
A03	HLA-A*03:14	1065	VTYVPAQEK	0,9087
A03	HLA-A*03:14	1099	GTHWFVTQR	0,5011

A03	HLA-A*03:14	1196	SLIDLQELGK	0,6419
A03	HLA-A*03:14	1264	VLKGVKLHY	0,6718
A03	HLA-A*03:16	35	GVYYPDKVFR	0,568
A03	HLA-A*03:16	41	KVFRSSVLH	0,6944
A03	HLA-A*03:16	89	GVYFASTEK	0,9487
A03	HLA-A*03:16	142	GVYYHKNNK	0,8871
A03	HLA-A*03:16	269	YLQPRTFLLK	0,5577
A03	HLA-A*03:16	302	TLKSFTVEK	0,8988
A03	HLA-A*03:16	311	GIYQTSNFR	0,6304
A03	HLA-A*03:16	349	SVYAWNRKR	0,6333
A03	HLA-A*03:16	367	VLYNSASFSTFK	0,5358
A03	HLA-A*03:16	378	KCYGVSPTK	0,5631
A03	HLA-A*03:16	408	RQIAPGQTGK	0,8957
A03	HLA-A*03:16	409	QIAPGQTGK	0,6982
A03	HLA-A*03:16	454	RLFRKSNLK	0,9589
A03	HLA-A*03:16	529	KSTNLVKNK	0,5122
A03	HLA-A*03:16	724	TEILPVSMTK	0,6838
A03	HLA-A*03:16	786	KQIYKTPPIK	0,8503
A03	HLA-A*03:16	787	QIYKTPPIK	0,8934
A03	HLA-A*03:16	805	ILPDPSKPSK	0,5588
A03	HLA-A*03:16	826	VTLADAGFIK	0,5395
A03	HLA-A*03:16	827	TLADAGFIK	0,7267
A03	HLA-A*03:16	924	ANQFNSAIGK	0,5091
A03	HLA-A*03:16	939	SSTASALGK	0,5175
A03	HLA-A*03:16	1019	RASANLAATK	0,5521
A03	HLA-A*03:16	1020	ASANLAATK	0,7894
A03	HLA-A*03:16	1064	HVTYVPAQEK	0,6951
A03	HLA-A*03:16	1065	VTYVPAQEK	0,9087
A03	HLA-A*03:16	1099	GTHWFVTQR	0,5011
A03	HLA-A*03:16	1196	SLIDLQELGK	0,6419
A03	HLA-A*03:16	1264	VLKGVKLHY	0,6718
A03	HLA-A*03:17	35	GVYYPDKVFR	0,568
A03	HLA-A*03:17	41	KVFRSSVLH	0,6944
A03	HLA-A*03:17	89	GVYFASTEK	0,9487
A03	HLA-A*03:17	142	GVYYHKNNK	0,8871
A03	HLA-A*03:17	269	YLQPRTFLLK	0,5577
A03	HLA-A*03:17	302	TLKSFTVEK	0,8988
A03	HLA-A*03:17	311	GIYQTSNFR	0,6304
A03	HLA-A*03:17	349	SVYAWNRKR	0,6333
A03	HLA-A*03:17	367	VLYNSASFSTFK	0,5358
A03	HLA-A*03:17	378	KCYGVSPTK	0,5631
A03	HLA-A*03:17	408	RQIAPGQTGK	0,8957
A03	HLA-A*03:17	409	QIAPGQTGK	0,6982
A03	HLA-A*03:17	454	RLFRKSNLK	0,9589
A03	HLA-A*03:17	529	KSTNLVKNK	0,5122
A03	HLA-A*03:17	724	TEILPVSMTK	0,6838
A03	HLA-A*03:17	786	KQIYKTPPIK	0,8503
A03	HLA-A*03:17	787	QIYKTPPIK	0,8934
A03	HLA-A*03:17	805	ILPDPSKPSK	0,5588
A03	HLA-A*03:17	826	VTLADAGFIK	0,5395

A03	HLA-A*03:17	827	TLADAGFIK	0,7267
A03	HLA-A*03:17	924	ANQFNSAIGK	0,5091
A03	HLA-A*03:17	939	SSTASALGK	0,5175
A03	HLA-A*03:17	1019	RASANLAATK	0,5521
A03	HLA-A*03:17	1020	ASANLAATK	0,7894
A03	HLA-A*03:17	1064	HVTYVPAQEK	0,6951
A03	HLA-A*03:17	1065	VTYVPAQEK	0,9087
A03	HLA-A*03:17	1099	GTHWFVTQR	0,5011
A03	HLA-A*03:17	1196	SLIDLQELGK	0,6419
A03	HLA-A*03:17	1264	VLKGVKLHY	0,6718
A03	HLA-A*11:01	35	GVYYPDKVFR	0,6555
A03	HLA-A*11:01	89	GVYFASTEK	0,9386
A03	HLA-A*11:01	142	GVYYHKNNK	0,7229
A03	HLA-A*11:01	292	ALDPLSETK	0,6795
A03	HLA-A*11:01	302	TLKSFTVEK	0,819
A03	HLA-A*11:01	311	GIYQTSNFR	0,5901
A03	HLA-A*11:01	348	ASVYAWNRK	0,6551
A03	HLA-A*11:01	349	SVYAWNRKR	0,576
A03	HLA-A*11:01	369	YNSASFSTFK	0,5561
A03	HLA-A*11:01	370	NSASFSTFK	0,8025
A03	HLA-A*11:01	375	STFKCYGVSPTK	0,6638
A03	HLA-A*11:01	408	RQIAPGQTGK	0,6249
A03	HLA-A*11:01	409	QIAPGQTGK	0,6748
A03	HLA-A*11:01	454	RLFRKSNLK	0,6772
A03	HLA-A*11:01	550	GVLTESNKK	0,7312
A03	HLA-A*11:01	686	SVASQSIIAY	0,5292
A03	HLA-A*11:01	723	TTEILPVSMTK	0,5964
A03	HLA-A*11:01	724	TEILPVSMTK	0,7926
A03	HLA-A*11:01	725	EILPVSMTK	0,7733
A03	HLA-A*11:01	787	QIYKTPPIK	0,7378
A03	HLA-A*11:01	825	KVTLADAGFIK	0,5709
A03	HLA-A*11:01	826	VTLADAGFIK	0,7264
A03	HLA-A*11:01	827	TLADAGFIK	0,8305
A03	HLA-A*11:01	925	NQFNSAIGK	0,5165
A03	HLA-A*11:01	939	SSTASALGK	0,7797
A03	HLA-A*11:01	956	AQALNTLVK	0,5151
A03	HLA-A*11:01	975	SVLNDILSR	0,8499
A03	HLA-A*11:01	975	SVLNDILSRLDK	0,5938
A03	HLA-A*11:01	1019	RASANLAATK	0,5185
A03	HLA-A*11:01	1020	ASANLAATK	0,9119
A03	HLA-A*11:01	1064	HVTYVPAQEK	0,6484
A03	HLA-A*11:01	1065	VTYVPAQEK	0,9153
A03	HLA-A*11:01	1099	GTHWFVTQR	0,7768
A03	HLA-A*11:01	1196	SLIDLQELGK	0,5993
A03	HLA-A*11:02	35	GVYYPDKVFR	0,6555
A03	HLA-A*11:02	89	GVYFASTEK	0,9386
A03	HLA-A*11:02	142	GVYYHKNNK	0,7229
A03	HLA-A*11:02	292	ALDPLSETK	0,6795
A03	HLA-A*11:02	302	TLKSFTVEK	0,819
A03	HLA-A*11:02	311	GIYQTSNFR	0,5901

A03	HLA-A*11:02	348	ASVYAWNRK	0,6551
A03	HLA-A*11:02	349	SVYAWNRKR	0,576
A03	HLA-A*11:02	369	YNSASFSTFK	0,5561
A03	HLA-A*11:02	370	NSASFSTFK	0,8025
A03	HLA-A*11:02	375	STFKCYGVSPTK	0,6638
A03	HLA-A*11:02	408	RQIAPGQTGK	0,6249
A03	HLA-A*11:02	409	QIAPGQTGK	0,6748
A03	HLA-A*11:02	454	RLFRKSNLK	0,6772
A03	HLA-A*11:02	550	GVLTESNKK	0,7312
A03	HLA-A*11:02	686	SVASQSIIAY	0,5292
A03	HLA-A*11:02	723	TTEILPVSMTK	0,5964
A03	HLA-A*11:02	724	TEILPVSMTK	0,7926
A03	HLA-A*11:02	725	EILPVSMTK	0,7733
A03	HLA-A*11:02	787	QIYKTPPIK	0,7378
A03	HLA-A*11:02	825	KVTLADAGFIK	0,5709
A03	HLA-A*11:02	826	VTLADAGFIK	0,7264
A03	HLA-A*11:02	827	TLADAGFIK	0,8305
A03	HLA-A*11:02	925	NQFNSAIGK	0,5165
A03	HLA-A*11:02	939	SSTASALGK	0,7797
A03	HLA-A*11:02	956	AQALNTLVK	0,5151
A03	HLA-A*11:02	975	SVLNDILSR	0,8499
A03	HLA-A*11:02	975	SVLNDILSRLDK	0,5938
A03	HLA-A*11:02	1019	RASANLAATK	0,5185
A03	HLA-A*11:02	1020	ASANLAATK	0,9119
A03	HLA-A*11:02	1064	HVTYVPAQEK	0,6484
A03	HLA-A*11:02	1065	VTYVPAQEK	0,9153
A03	HLA-A*11:02	1099	GTHWFVTQR	0,7768
A03	HLA-A*11:02	1196	SLIDLQELGK	0,5993
A03	HLA-A*11:03	35	GVYYPDKVFR	0,5729
A03	HLA-A*11:03	41	KVFRSSVLH	0,6359
A03	HLA-A*11:03	69	HVSGTNGTK	0,5574
A03	HLA-A*11:03	89	GVYFASTEK	0,9479
A03	HLA-A*11:03	142	GVYYHKNNK	0,8636
A03	HLA-A*11:03	257	GWTAGAAAYY	0,5027
A03	HLA-A*11:03	292	ALDPLSETK	0,6109
A03	HLA-A*11:03	302	TLKSFTVEK	0,8758
A03	HLA-A*11:03	311	GIYQTSNFR	0,6106
A03	HLA-A*11:03	348	ASVYAWNRK	0,6559
A03	HLA-A*11:03	349	SVYAWNRKR	0,6192
A03	HLA-A*11:03	369	YNSASFSTFK	0,5161
A03	HLA-A*11:03	370	NSASFSTFK	0,7603
A03	HLA-A*11:03	375	STFKCYGVSPTK	0,6584
A03	HLA-A*11:03	408	RQIAPGQTGK	0,8115
A03	HLA-A*11:03	409	QIAPGQTGK	0,7924
A03	HLA-A*11:03	454	RLFRKSNLK	0,839
A03	HLA-A*11:03	529	KSTNLVKNK	0,6205
A03	HLA-A*11:03	550	GVLTESNKK	0,7419
A03	HLA-A*11:03	686	SVASQSIIAY	0,5979
A03	HLA-A*11:03	723	TTEILPVSMTK	0,5555
A03	HLA-A*11:03	724	TEILPVSMTK	0,7868

A03	HLA-A*11:03	725	EILPVSMTK	0,7674
A03	HLA-A*11:03	786	KQIYKTPPIK	0,5919
A03	HLA-A*11:03	787	QIYKTPPIK	0,8358
A03	HLA-A*11:03	825	KVTLADAGFIK	0,5148
A03	HLA-A*11:03	826	VTLADAGFIK	0,6588
A03	HLA-A*11:03	827	TLADAGFIK	0,7979
A03	HLA-A*11:03	924	ANQFNSAIGK	0,5982
A03	HLA-A*11:03	925	NQFNSAIGK	0,5795
A03	HLA-A*11:03	939	SSTASALGK	0,7993
A03	HLA-A*11:03	956	AQALNTLVK	0,5795
A03	HLA-A*11:03	975	SVLNDILSR	0,7656
A03	HLA-A*11:03	975	SVLNDILSRLDK	0,5176
A03	HLA-A*11:03	1019	RASANLAATK	0,6171
A03	HLA-A*11:03	1020	ASANLAATK	0,9033
A03	HLA-A*11:03	1064	HVTYVPAQEK	0,7064
A03	HLA-A*11:03	1065	VTYVPAQEK	0,9204
A03	HLA-A*11:03	1099	GTHWFVTQR	0,7038
A03	HLA-A*11:03	1196	SLIDLQELGK	0,6467
A03	HLA-A*11:04	35	GVYYPDKVFR	0,6723
A03	HLA-A*11:04	89	GVYFASTEK	0,9041
A03	HLA-A*11:04	142	GVYYHKNNK	0,7342
A03	HLA-A*11:04	292	ALDPLSETK	0,5968
A03	HLA-A*11:04	302	TLKSFTVEK	0,8186
A03	HLA-A*11:04	311	GIYQTSNFR	0,6771
A03	HLA-A*11:04	319	RVQPTESIVR	0,5363
A03	HLA-A*11:04	348	ASVYAWNRK	0,5541
A03	HLA-A*11:04	349	SVYAWNRKR	0,646
A03	HLA-A*11:04	370	NSASFSTFK	0,6355
A03	HLA-A*11:04	375	STFKCYGVSPTK	0,5031
A03	HLA-A*11:04	408	RQIAPGQTGK	0,7357
A03	HLA-A*11:04	409	QIAPGQTGK	0,6296
A03	HLA-A*11:04	454	RLFRKSNLK	0,8162
A03	HLA-A*11:04	458	KSNLKPFER	0,6282
A03	HLA-A*11:04	529	KSTNLVKNK	0,5664
A03	HLA-A*11:04	550	GVLTESNKK	0,6125
A03	HLA-A*11:04	724	TEILPVSMTK	0,653
A03	HLA-A*11:04	725	EILPVSMTK	0,6293
A03	HLA-A*11:04	786	KQIYKTPPIK	0,5121
A03	HLA-A*11:04	787	QIYKTPPIK	0,7537
A03	HLA-A*11:04	825	KVTLADAGFIK	0,5571
A03	HLA-A*11:04	826	VTLADAGFIK	0,5926
A03	HLA-A*11:04	827	TLADAGFIK	0,7565
A03	HLA-A*11:04	939	SSTASALGK	0,6578
A03	HLA-A*11:04	956	AQALNTLVK	0,5269
A03	HLA-A*11:04	975	SVLNDILSR	0,7843
A03	HLA-A*11:04	1019	RASANLAATK	0,5722
A03	HLA-A*11:04	1020	ASANLAATK	0,8712
A03	HLA-A*11:04	1064	HVTYVPAQEK	0,5792
A03	HLA-A*11:04	1065	VTYVPAQEK	0,8811
A03	HLA-A*11:04	1099	GTHWFVTQR	0,7363

A03	HLA-A*11:05	35	GVYYPDKVFR	0,6555
A03	HLA-A*11:05	89	GVYFASTEK	0,9386
A03	HLA-A*11:05	142	GVYYHKNNK	0,7229
A03	HLA-A*11:05	292	ALDPLSETK	0,6795
A03	HLA-A*11:05	302	TLKSFTVEK	0,819
A03	HLA-A*11:05	311	GIYQTSNFR	0,5901
A03	HLA-A*11:05	348	ASVYAWNRK	0,6551
A03	HLA-A*11:05	349	SVYAWNRKR	0,576
A03	HLA-A*11:05	369	YNSASFSTFK	0,5561
A03	HLA-A*11:05	370	NSASFSTFK	0,8025
A03	HLA-A*11:05	375	STFKCYGVSPTK	0,6638
A03	HLA-A*11:05	408	RQIAPGQTGK	0,6249
A03	HLA-A*11:05	409	QIAPGQTGK	0,6748
A03	HLA-A*11:05	454	RLFRKSNLK	0,6772
A03	HLA-A*11:05	550	GVLTESNKK	0,7312
A03	HLA-A*11:05	686	SVASQSIIAY	0,5292
A03	HLA-A*11:05	723	TTEILPVSMTK	0,5964
A03	HLA-A*11:05	724	TEILPVSMTK	0,7926
A03	HLA-A*11:05	725	EILPVSMTK	0,7733
A03	HLA-A*11:05	787	QIYKTPPIK	0,7378
A03	HLA-A*11:05	825	KVTLADAGFIK	0,5709
A03	HLA-A*11:05	826	VTLADAGFIK	0,7264
A03	HLA-A*11:05	827	TLADAGFIK	0,8305
A03	HLA-A*11:05	925	NQFNSAIGK	0,5165
A03	HLA-A*11:05	939	SSTASALGK	0,7797
A03	HLA-A*11:05	956	AQALNTLVK	0,5151
A03	HLA-A*11:05	975	SVLNDILSR	0,8499
A03	HLA-A*11:05	975	SVLNDILSRLDK	0,5938
A03	HLA-A*11:05	1019	RASANLAATK	0,5185
A03	HLA-A*11:05	1020	ASANLAATK	0,9119
A03	HLA-A*11:05	1064	HVTYVPAQEK	0,6484
A03	HLA-A*11:05	1065	VTYVPAQEK	0,9153
A03	HLA-A*11:05	1099	GTHWFVTQR	0,7768
A03	HLA-A*11:05	1196	SLIDLQELGK	0,5993
A03	HLA-A*11:07	35	GVYYPDKVFR	0,6555
A03	HLA-A*11:07	89	GVYFASTEK	0,9386
A03	HLA-A*11:07	142	GVYYHKNNK	0,7229
A03	HLA-A*11:07	292	ALDPLSETK	0,6795
A03	HLA-A*11:07	302	TLKSFTVEK	0,819
A03	HLA-A*11:07	311	GIYQTSNFR	0,5901
A03	HLA-A*11:07	348	ASVYAWNRK	0,6551
A03	HLA-A*11:07	349	SVYAWNRKR	0,576
A03	HLA-A*11:07	369	YNSASFSTFK	0,5561
A03	HLA-A*11:07	370	NSASFSTFK	0,8025
A03	HLA-A*11:07	375	STFKCYGVSPTK	0,6638
A03	HLA-A*11:07	408	RQIAPGQTGK	0,6249
A03	HLA-A*11:07	409	QIAPGQTGK	0,6748
A03	HLA-A*11:07	454	RLFRKSNLK	0,6772
A03	HLA-A*11:07	550	GVLTESNKK	0,7312
A03	HLA-A*11:07	686	SVASQSIIAY	0,5292

A03	HLA-A*11:07	723	TTEILPVSMTK	0,5964
A03	HLA-A*11:07	724	TEILPVSMTK	0,7926
A03	HLA-A*11:07	725	EILPVSMTK	0,7733
A03	HLA-A*11:07	787	QIYKTPPIK	0,7378
A03	HLA-A*11:07	825	KVTLADAGFIK	0,5709
A03	HLA-A*11:07	826	VTLADAGFIK	0,7264
A03	HLA-A*11:07	827	TLADAGFIK	0,8305
A03	HLA-A*11:07	925	NQFNSAIGK	0,5165
A03	HLA-A*11:07	939	SSTASALGK	0,7797
A03	HLA-A*11:07	956	AQALNTLVK	0,5151
A03	HLA-A*11:07	975	SVLNDILSR	0,8499
A03	HLA-A*11:07	975	SVLNDILSRLDK	0,5938
A03	HLA-A*11:07	1019	RASANLAATK	0,5185
A03	HLA-A*11:07	1020	ASANLAATK	0,9119
A03	HLA-A*11:07	1064	HVTYVPAQEK	0,6484
A03	HLA-A*11:07	1065	VTYVPAQEK	0,9153
A03	HLA-A*11:07	1099	GTHWFVTQR	0,7768
A03	HLA-A*11:07	1196	SLIDLQELGK	0,5993
A03	HLA-A*11:08	30	NSFTRGVYY	0,6147
A03	HLA-A*11:08	41	KVFRSSVLH	0,5716
A03	HLA-A*11:08	69	HVSGTNGTK	0,5285
A03	HLA-A*11:08	89	GVYFASTEK	0,9181
A03	HLA-A*11:08	142	GVYYHKNNK	0,7168
A03	HLA-A*11:08	257	GWTAGAAAYY	0,6585
A03	HLA-A*11:08	258	WTAGAAAYY	0,5874
A03	HLA-A*11:08	261	GAAAYYVGY	0,5405
A03	HLA-A*11:08	292	ALDPLSETK	0,7101
A03	HLA-A*11:08	302	TLKSFTVEK	0,8239
A03	HLA-A*11:08	311	GIYQTSNFR	0,5198
A03	HLA-A*11:08	348	ASVYAWNRK	0,6142
A03	HLA-A*11:08	349	SVYAWNRKR	0,5031
A03	HLA-A*11:08	361	CVADYSVLY	0,5983
A03	HLA-A*11:08	369	YNSASFSTFK	0,5604
A03	HLA-A*11:08	370	NSASFSTFK	0,8068
A03	HLA-A*11:08	375	STFKCYGVSPTK	0,5765
A03	HLA-A*11:08	408	RQIAPGQTGK	0,7216
A03	HLA-A*11:08	409	QIAPGQTGK	0,7553
A03	HLA-A*11:08	454	RLFRKSNLK	0,707
A03	HLA-A*11:08	550	GVLTESNKK	0,7019
A03	HLA-A*11:08	604	TSNQVAVLY	0,6574
A03	HLA-A*11:08	686	SVASQSIIAY	0,7162
A03	HLA-A*11:08	723	TTEILPVSMTK	0,519
A03	HLA-A*11:08	724	TEILPVSMTK	0,7064
A03	HLA-A*11:08	725	EILPVSMTK	0,7265
A03	HLA-A*11:08	787	QIYKTPPIK	0,7324
A03	HLA-A*11:08	805	ILPDPSKPSK	0,536
A03	HLA-A*11:08	825	KVTLADAGFIK	0,5029
A03	HLA-A*11:08	826	VTLADAGFIK	0,6667
A03	HLA-A*11:08	827	TLADAGFIK	0,8043
A03	HLA-A*11:08	925	NQFNSAIGK	0,5146

A03	HLA-A*11:08	939	SSTASALGK	0,7826
A03	HLA-A*11:08	956	AQALNTLVK	0,5229
A03	HLA-A*11:08	975	SVLNDILSR	0,7316
A03	HLA-A*11:08	1019	RASANLAATK	0,5652
A03	HLA-A*11:08	1020	ASANLAATK	0,8837
A03	HLA-A*11:08	1064	HVTYVPAQEK	0,6371
A03	HLA-A*11:08	1065	VTYVPAQEK	0,8948
A03	HLA-A*11:08	1099	GTHWFVTQR	0,6403
A03	HLA-A*11:08	1196	SLIDLQELGK	0,5719
A03	HLA-A*11:09	35	GVYYPDKVFR	0,6555
A03	HLA-A*11:09	89	GVYFASTEK	0,9386
A03	HLA-A*11:09	142	GVYYHKNNK	0,7229
A03	HLA-A*11:09	292	ALDPLSETK	0,6795
A03	HLA-A*11:09	302	TLKSFTVEK	0,819
A03	HLA-A*11:09	311	GIYQTSNFR	0,5901
A03	HLA-A*11:09	348	ASVYAWNRK	0,6551
A03	HLA-A*11:09	349	SVYAWNRKR	0,576
A03	HLA-A*11:09	369	YNSASFSTFK	0,5561
A03	HLA-A*11:09	370	NSASFSTFK	0,8025
A03	HLA-A*11:09	375	STFKCYGVSPTK	0,6638
A03	HLA-A*11:09	408	RQIAPGQTGK	0,6249
A03	HLA-A*11:09	409	QIAPGQTGK	0,6748
A03	HLA-A*11:09	454	RLFRKSNLK	0,6772
A03	HLA-A*11:09	550	GVLTESNKK	0,7312
A03	HLA-A*11:09	686	SVASQSIIAY	0,5292
A03	HLA-A*11:09	723	TTEILPVSMTK	0,5964
A03	HLA-A*11:09	724	TEILPVSMTK	0,7926
A03	HLA-A*11:09	725	EILPVSMTK	0,7733
A03	HLA-A*11:09	787	QIYKTPPIK	0,7378
A03	HLA-A*11:09	825	KVTLADAGFIK	0,5709
A03	HLA-A*11:09	826	VTLADAGFIK	0,7264
A03	HLA-A*11:09	827	TLADAGFIK	0,8305
A03	HLA-A*11:09	925	NQFNSAIGK	0,5165
A03	HLA-A*11:09	939	SSTASALGK	0,7797
A03	HLA-A*11:09	956	AQALNTLVK	0,5151
A03	HLA-A*11:09	975	SVLNDILSR	0,8499
A03	HLA-A*11:09	975	SVLNDILSRLDK	0,5938
A03	HLA-A*11:09	1019	RASANLAATK	0,5185
A03	HLA-A*11:09	1020	ASANLAATK	0,9119
A03	HLA-A*11:09	1064	HVTYVPAQEK	0,6484
A03	HLA-A*11:09	1065	VTYVPAQEK	0,9153
A03	HLA-A*11:09	1099	GTHWFVTQR	0,7768
A03	HLA-A*11:09	1196	SLIDLQELGK	0,5993
A03	HLA-A*11:10	30	NSFTRGVYY	0,5648
A03	HLA-A*11:10	88	DGVYFASTEK	0,5092
A03	HLA-A*11:10	89	GVYFASTEK	0,6152
A03	HLA-A*11:10	258	WTAGAAAYY	0,5276
A03	HLA-A*11:10	361	CVADYSVLY	0,5044
A03	HLA-A*11:10	370	NSASFSTFK	0,7786
A03	HLA-A*11:10	724	TEILPVSMTK	0,5103

A03	HLA-A*11:10	725	EILPVSMTK	0,8498
A03	HLA-A*11:10	817	FIEDLLFNK	0,5044
A03	HLA-A*11:10	827	TLADAGFIK	0,6366
A03	HLA-A*11:10	975	SVLNDILSR	0,6653
A03	HLA-A*11:10	1065	VTYVPAQEK	0,5627
A03	HLA-A*11:10	1173	NASVVNIQK	0,5907
A03	HLA-A*11:12	35	GVYYPDKVFR	0,6555
A03	HLA-A*11:12	89	GVYFASTEK	0,9386
A03	HLA-A*11:12	142	GVYYHKNNK	0,7229
A03	HLA-A*11:12	292	ALDPLSETK	0,6795
A03	HLA-A*11:12	302	TLKSFTVEK	0,819
A03	HLA-A*11:12	311	GIYQTSNFR	0,5901
A03	HLA-A*11:12	348	ASVYAWNRK	0,6551
A03	HLA-A*11:12	349	SVYAWNRKR	0,576
A03	HLA-A*11:12	369	YNSASFSTFK	0,5561
A03	HLA-A*11:12	370	NSASFSTFK	0,8025
A03	HLA-A*11:12	375	STFKCYGVSPTK	0,6638
A03	HLA-A*11:12	408	RQIAPGQTGK	0,6249
A03	HLA-A*11:12	409	QIAPGQTGK	0,6748
A03	HLA-A*11:12	454	RLFRKSNLK	0,6772
A03	HLA-A*11:12	550	GVLTESNKK	0,7312
A03	HLA-A*11:12	686	SVASQSIIAY	0,5292
A03	HLA-A*11:12	723	TTEILPVSMTK	0,5964
A03	HLA-A*11:12	724	TEILPVSMTK	0,7926
A03	HLA-A*11:12	725	EILPVSMTK	0,7733
A03	HLA-A*11:12	787	QIYKTPPIK	0,7378
A03	HLA-A*11:12	825	KVTLADAGFIK	0,5709
A03	HLA-A*11:12	826	VTLADAGFIK	0,7264
A03	HLA-A*11:12	827	TLADAGFIK	0,8305
A03	HLA-A*11:12	925	NQFNSAIGK	0,5165
A03	HLA-A*11:12	939	SSTASALGK	0,7797
A03	HLA-A*11:12	956	AQALNTLVK	0,5151
A03	HLA-A*11:12	975	SVLNDILSR	0,8499
A03	HLA-A*11:12	975	SVLNDILSRLDK	0,5938
A03	HLA-A*11:12	1019	RASANLAATK	0,5185
A03	HLA-A*11:12	1020	ASANLAATK	0,9119
A03	HLA-A*11:12	1064	HVTYVPAQEK	0,6484
A03	HLA-A*11:12	1065	VTYVPAQEK	0,9153
A03	HLA-A*11:12	1099	GTHWFVTQR	0,7768
A03	HLA-A*11:12	1196	SLIDLQELGK	0,5993
A03	HLA-A*11:13	35	GVYYPDKVFR	0,6555
A03	HLA-A*11:13	89	GVYFASTEK	0,9386
A03	HLA-A*11:13	142	GVYYHKNNK	0,7229
A03	HLA-A*11:13	292	ALDPLSETK	0,6795
A03	HLA-A*11:13	302	TLKSFTVEK	0,819
A03	HLA-A*11:13	311	GIYQTSNFR	0,5901
A03	HLA-A*11:13	348	ASVYAWNRK	0,6551
A03	HLA-A*11:13	349	SVYAWNRKR	0,576
A03	HLA-A*11:13	369	YNSASFSTFK	0,5561
A03	HLA-A*11:13	370	NSASFSTFK	0,8025

A03	HLA-A*11:13	375	STFKCYGVSPTK	0,6638
A03	HLA-A*11:13	408	RQIAPGQTGK	0,6249
A03	HLA-A*11:13	409	QIAPGQTGK	0,6748
A03	HLA-A*11:13	454	RLFRKSNLK	0,6772
A03	HLA-A*11:13	550	GVLTESNKK	0,7312
A03	HLA-A*11:13	686	SVASQSIIAY	0,5292
A03	HLA-A*11:13	723	TTEILPVSMTK	0,5964
A03	HLA-A*11:13	724	TEILPVSMTK	0,7926
A03	HLA-A*11:13	725	EILPVSMTK	0,7733
A03	HLA-A*11:13	787	QIYKTPPIK	0,7378
A03	HLA-A*11:13	825	KVTLADAGFIK	0,5709
A03	HLA-A*11:13	826	VTLADAGFIK	0,7264
A03	HLA-A*11:13	827	TLADAGFIK	0,8305
A03	HLA-A*11:13	925	NQFNSAIGK	0,5165
A03	HLA-A*11:13	939	SSTASALGK	0,7797
A03	HLA-A*11:13	956	AQALNTLVK	0,5151
A03	HLA-A*11:13	975	SVLNDILSR	0,8499
A03	HLA-A*11:13	975	SVLNDILSRLDK	0,5938
A03	HLA-A*11:13	1019	RASANLAATK	0,5185
A03	HLA-A*11:13	1020	ASANLAATK	0,9119
A03	HLA-A*11:13	1064	HVTYVPAQEK	0,6484
A03	HLA-A*11:13	1065	VTYVPAQEK	0,9153
A03	HLA-A*11:13	1099	GTHWFVTQR	0,7768
A03	HLA-A*11:13	1196	SLIDLQELGK	0,5993
A03	HLA-A*11:14	41	KVFRSSVLH	0,5801
A03	HLA-A*11:14	89	GVYFASTEK	0,9113
A03	HLA-A*11:14	142	GVYYHKNNK	0,7242
A03	HLA-A*11:14	292	ALDPLSETK	0,552
A03	HLA-A*11:14	302	TLKSFTVEK	0,7933
A03	HLA-A*11:14	311	GIYQTSNFR	0,5287
A03	HLA-A*11:14	348	ASVYAWNRK	0,5921
A03	HLA-A*11:14	349	SVYAWNRKR	0,524
A03	HLA-A*11:14	370	NSASFSTFK	0,7046
A03	HLA-A*11:14	375	STFKCYGVSPTK	0,5354
A03	HLA-A*11:14	408	RQIAPGQTGK	0,6772
A03	HLA-A*11:14	409	QIAPGQTGK	0,6664
A03	HLA-A*11:14	454	RLFRKSNLK	0,7323
A03	HLA-A*11:14	550	GVLTESNKK	0,6399
A03	HLA-A*11:14	686	SVASQSIIAY	0,5437
A03	HLA-A*11:14	724	TEILPVSMTK	0,6944
A03	HLA-A*11:14	725	EILPVSMTK	0,6759
A03	HLA-A*11:14	787	QIYKTPPIK	0,728
A03	HLA-A*11:14	826	VTLADAGFIK	0,595
A03	HLA-A*11:14	827	TLADAGFIK	0,7304
A03	HLA-A*11:14	924	ANQFNSAIGK	0,5078
A03	HLA-A*11:14	925	NQFNSAIGK	0,519
A03	HLA-A*11:14	939	SSTASALGK	0,7137
A03	HLA-A*11:14	956	AQALNTLVK	0,5035
A03	HLA-A*11:14	975	SVLNDILSR	0,743
A03	HLA-A*11:14	1019	RASANLAATK	0,5006

A03	HLA-A*11:14	1020	ASANLAATK	0,857
A03	HLA-A*11:14	1064	HVTYVPAQEK	0,5851
A03	HLA-A*11:14	1065	VTYVPAQEK	0,8766
A03	HLA-A*11:14	1099	GTHWFVTQR	0,6316
A03	HLA-A*11:14	1196	SLIDLQELGK	0,536
A03	HLA-A*11:15	35	GVYYPDKVFR	0,6555
A03	HLA-A*11:15	89	GVYFASTEK	0,9386
A03	HLA-A*11:15	142	GVYYHKNNK	0,7229
A03	HLA-A*11:15	292	ALDPLSETK	0,6795
A03	HLA-A*11:15	302	TLKSFTVEK	0,819
A03	HLA-A*11:15	311	GIYQTSNFR	0,5901
A03	HLA-A*11:15	348	ASVYAWNRK	0,6551
A03	HLA-A*11:15	349	SVYAWNRKR	0,576
A03	HLA-A*11:15	369	YNSASFSTFK	0,5561
A03	HLA-A*11:15	370	NSASFSTFK	0,8025
A03	HLA-A*11:15	375	STFKCYGVSPTK	0,6638
A03	HLA-A*11:15	408	RQIAPGQTGK	0,6249
A03	HLA-A*11:15	409	QIAPGQTGK	0,6748
A03	HLA-A*11:15	454	RLFRKSNLK	0,6772
A03	HLA-A*11:15	550	GVLTESNKK	0,7312
A03	HLA-A*11:15	686	SVASQSIIAY	0,5292
A03	HLA-A*11:15	723	TTEILPVSMTK	0,5964
A03	HLA-A*11:15	724	TEILPVSMTK	0,7926
A03	HLA-A*11:15	725	EILPVSMTK	0,7733
A03	HLA-A*11:15	787	QIYKTPPIK	0,7378
A03	HLA-A*11:15	825	KVTLADAGFIK	0,5709
A03	HLA-A*11:15	826	VTLADAGFIK	0,7264
A03	HLA-A*11:15	827	TLADAGFIK	0,8305
A03	HLA-A*11:15	925	NQFNSAIGK	0,5165
A03	HLA-A*11:15	939	SSTASALGK	0,7797
A03	HLA-A*11:15	956	AQALNTLVK	0,5151
A03	HLA-A*11:15	975	SVLNDILSR	0,8499
A03	HLA-A*11:15	975	SVLNDILSRLDK	0,5938
A03	HLA-A*11:15	1019	RASANLAATK	0,5185
A03	HLA-A*11:15	1020	ASANLAATK	0,9119
A03	HLA-A*11:15	1064	HVTYVPAQEK	0,6484
A03	HLA-A*11:15	1065	VTYVPAQEK	0,9153
A03	HLA-A*11:15	1099	GTHWFVTQR	0,7768
A03	HLA-A*11:15	1196	SLIDLQELGK	0,5993
A03	HLA-A*11:16	35	GVYYPDKVFR	0,6555
A03	HLA-A*11:16	89	GVYFASTEK	0,9386
A03	HLA-A*11:16	142	GVYYHKNNK	0,7229
A03	HLA-A*11:16	292	ALDPLSETK	0,6795
A03	HLA-A*11:16	302	TLKSFTVEK	0,819
A03	HLA-A*11:16	311	GIYQTSNFR	0,5901
A03	HLA-A*11:16	348	ASVYAWNRK	0,6551
A03	HLA-A*11:16	349	SVYAWNRKR	0,576
A03	HLA-A*11:16	369	YNSASFSTFK	0,5561
A03	HLA-A*11:16	370	NSASFSTFK	0,8025
A03	HLA-A*11:16	375	STFKCYGVSPTK	0,6638

A03	HLA-A*11:16	408	RQIAPGQTGK	0,6249
A03	HLA-A*11:16	409	QIAPGQTGK	0,6748
A03	HLA-A*11:16	454	RLFRKSNLK	0,6772
A03	HLA-A*11:16	550	GVLTESNKK	0,7312
A03	HLA-A*11:16	686	SVASQSIIAY	0,5292
A03	HLA-A*11:16	723	TTEILPVSMTK	0,5964
A03	HLA-A*11:16	724	TEILPVSMTK	0,7926
A03	HLA-A*11:16	725	EILPVSMTK	0,7733
A03	HLA-A*11:16	787	QIYKTPPIK	0,7378
A03	HLA-A*11:16	825	KVTLADAGFIK	0,5709
A03	HLA-A*11:16	826	VTLADAGFIK	0,7264
A03	HLA-A*11:16	827	TLADAGFIK	0,8305
A03	HLA-A*11:16	925	NQFNSAIGK	0,5165
A03	HLA-A*11:16	939	SSTASALGK	0,7797
A03	HLA-A*11:16	956	AQALNTLVK	0,5151
A03	HLA-A*11:16	975	SVLNDILSR	0,8499
A03	HLA-A*11:16	975	SVLNDILSRLDK	0,5938
A03	HLA-A*11:16	1019	RASANLAATK	0,5185
A03	HLA-A*11:16	1020	ASANLAATK	0,9119
A03	HLA-A*11:16	1064	HVTYVPAQEK	0,6484
A03	HLA-A*11:16	1065	VTYVPAQEK	0,9153
A03	HLA-A*11:16	1099	GTHWFVTQR	0,7768
A03	HLA-A*11:16	1196	SLIDLQELGK	0,5993
A03	HLA-A*11:20	35	GVYYPDKVFR	0,5729
A03	HLA-A*11:20	41	KVFRSSVLH	0,6359
A03	HLA-A*11:20	69	HVSGTNGTK	0,5574
A03	HLA-A*11:20	89	GVYFASTEK	0,9479
A03	HLA-A*11:20	142	GVYYHKNNK	0,8636
A03	HLA-A*11:20	257	GWTAGAAAYY	0,5027
A03	HLA-A*11:20	292	ALDPLSETK	0,6109
A03	HLA-A*11:20	302	TLKSFTVEK	0,8758
A03	HLA-A*11:20	311	GIYQTSNFR	0,6106
A03	HLA-A*11:20	348	ASVYAWNRK	0,6559
A03	HLA-A*11:20	349	SVYAWNRKR	0,6192
A03	HLA-A*11:20	369	YNSASFSTFK	0,5161
A03	HLA-A*11:20	370	NSASFSTFK	0,7603
A03	HLA-A*11:20	375	STFKCYGVSPTK	0,6584
A03	HLA-A*11:20	408	RQIAPGQTGK	0,8115
A03	HLA-A*11:20	409	QIAPGQTGK	0,7924
A03	HLA-A*11:20	454	RLFRKSNLK	0,839
A03	HLA-A*11:20	529	KSTNLVKNK	0,6205
A03	HLA-A*11:20	550	GVLTESNKK	0,7419
A03	HLA-A*11:20	686	SVASQSIIAY	0,5979
A03	HLA-A*11:20	723	TTEILPVSMTK	0,5555
A03	HLA-A*11:20	724	TEILPVSMTK	0,7868
A03	HLA-A*11:20	725	EILPVSMTK	0,7674
A03	HLA-A*11:20	786	KQIYKTPPIK	0,5919
A03	HLA-A*11:20	787	QIYKTPPIK	0,8358
A03	HLA-A*11:20	825	KVTLADAGFIK	0,5148
A03	HLA-A*11:20	826	VTLADAGFIK	0,6588

A03	HLA-A*11:20	827	TLADAGFIK	0,7979
A03	HLA-A*11:20	924	ANQFNSAIGK	0,5982
A03	HLA-A*11:20	925	NQFNSAIGK	0,5795
A03	HLA-A*11:20	939	SSTASALGK	0,7993
A03	HLA-A*11:20	956	AQALNTLVK	0,5795
A03	HLA-A*11:20	975	SVLNDILSR	0,7656
A03	HLA-A*11:20	975	SVLNDILSRLDK	0,5176
A03	HLA-A*11:20	1019	RASANLAATK	0,6171
A03	HLA-A*11:20	1020	ASANLAATK	0,9033
A03	HLA-A*11:20	1064	HVTYVPAQEK	0,7064
A03	HLA-A*11:20	1065	VTYVPAQEK	0,9204
A03	HLA-A*11:20	1099	GTHWFVTQR	0,7038
A03	HLA-A*11:20	1196	SLIDLQELGK	0,6467
A03	HLA-A*11:23	35	GVYYPDKVFR	0,6555
A03	HLA-A*11:23	89	GVYFASTEK	0,9386
A03	HLA-A*11:23	142	GVYYHKNNK	0,7229
A03	HLA-A*11:23	292	ALDPLSETK	0,6795
A03	HLA-A*11:23	302	TLKSFTVEK	0,819
A03	HLA-A*11:23	311	GIYQTSNFR	0,5901
A03	HLA-A*11:23	348	ASVYAWNRK	0,6551
A03	HLA-A*11:23	349	SVYAWNRKR	0,576
A03	HLA-A*11:23	369	YNSASFSTFK	0,5561
A03	HLA-A*11:23	370	NSASFSTFK	0,8025
A03	HLA-A*11:23	375	STFKCYGVSPTK	0,6638
A03	HLA-A*11:23	408	RQIAPGQTGK	0,6249
A03	HLA-A*11:23	409	QIAPGQTGK	0,6748
A03	HLA-A*11:23	454	RLFRKSNLK	0,6772
A03	HLA-A*11:23	550	GVLTESNKK	0,7312
A03	HLA-A*11:23	686	SVASQSIIAY	0,5292
A03	HLA-A*11:23	723	TTEILPVSMTK	0,5964
A03	HLA-A*11:23	724	TEILPVSMTK	0,7926
A03	HLA-A*11:23	725	EILPVSMTK	0,7733
A03	HLA-A*11:23	787	QIYKTPPIK	0,7378
A03	HLA-A*11:23	825	KVTLADAGFIK	0,5709
A03	HLA-A*11:23	826	VTLADAGFIK	0,7264
A03	HLA-A*11:23	827	TLADAGFIK	0,8305
A03	HLA-A*11:23	925	NQFNSAIGK	0,5165
A03	HLA-A*11:23	939	SSTASALGK	0,7797
A03	HLA-A*11:23	956	AQALNTLVK	0,5151
A03	HLA-A*11:23	975	SVLNDILSR	0,8499
A03	HLA-A*11:23	975	SVLNDILSRLDK	0,5938
A03	HLA-A*11:23	1019	RASANLAATK	0,5185
A03	HLA-A*11:23	1020	ASANLAATK	0,9119
A03	HLA-A*11:23	1064	HVTYVPAQEK	0,6484
A03	HLA-A*11:23	1065	VTYVPAQEK	0,9153
A03	HLA-A*11:23	1099	GTHWFVTQR	0,7768
A03	HLA-A*11:23	1196	SLIDLQELGK	0,5993
A03	HLA-A*31:01	34	RGVYYPDKVFR	0,5574
A03	HLA-A*31:01	35	GVYYPDKVFR	0,7294
A03	HLA-A*31:01	36	VYYPDKVFR	0,8085

A03	HLA-A*31:01	150	KSWMESEFR	0,5583
A03	HLA-A*31:01	182	KQGNFKNLR	0,6717
A03	HLA-A*31:01	310	KGIYQTSNFR	0,5075
A03	HLA-A*31:01	311	GIYQTSNFR	0,6612
A03	HLA-A*31:01	319	RVQPTESIVR	0,5738
A03	HLA-A*31:01	346	RFASVYAWNR	0,7025
A03	HLA-A*31:01	349	SVYAWNRKR	0,8646
A03	HLA-A*31:01	395	VYADSFVIR	0,575
A03	HLA-A*31:01	446	GGNYNYLYR	0,5086
A03	HLA-A*31:01	454	RLFRKSNLK	0,5237
A03	HLA-A*31:01	458	KSNLKPFER	0,8687
A03	HLA-A*31:01	558	KFLPFQQFGR	0,6235
A03	HLA-A*31:01	677	QTNSPRRAR	0,6557
A03	HLA-A*31:01	975	SVLNDILSR	0,6352
A03	HLA-A*31:01	1099	GTHWFVTQR	0,837
A03	HLA-A*31:03	34	RGVYYPDKVFR	0,6638
A03	HLA-A*31:03	35	GVYYPDKVFR	0,8323
A03	HLA-A*31:03	36	VYYPDKVFR	0,882
A03	HLA-A*31:03	142	GVYYHKNNK	0,5143
A03	HLA-A*31:03	150	KSWMESEFR	0,7064
A03	HLA-A*31:03	182	KQGNFKNLR	0,768
A03	HLA-A*31:03	193	VFKNIDGYFK	0,6092
A03	HLA-A*31:03	237	RFQTLLALHR	0,5604
A03	HLA-A*31:03	264	AYYVGYLQPR	0,5113
A03	HLA-A*31:03	302	TLKSFTVEK	0,6023
A03	HLA-A*31:03	310	KGIYQTSNFR	0,6
A03	HLA-A*31:03	311	GIYQTSNFR	0,7783
A03	HLA-A*31:03	319	RVQPTESIVR	0,6913
A03	HLA-A*31:03	346	RFASVYAWNR	0,73
A03	HLA-A*31:03	348	ASVYAWNRKR	0,5374
A03	HLA-A*31:03	349	SVYAWNRKR	0,8673
A03	HLA-A*31:03	394	NVYADSFVIR	0,5006
A03	HLA-A*31:03	395	VYADSFVIR	0,708
A03	HLA-A*31:03	446	GGNYNYLYR	0,7092
A03	HLA-A*31:03	449	YNYLYRLFR	0,5488
A03	HLA-A*31:03	454	RLFRKSNLK	0,6239
A03	HLA-A*31:03	457	RKSNLKPFER	0,5092
A03	HLA-A*31:03	458	KSNLKPFER	0,9139
A03	HLA-A*31:03	558	KFLPFQQFGR	0,7268
A03	HLA-A*31:03	637	STGSNVFQTR	0,6297
A03	HLA-A*31:03	638	TGSNVFQTR	0,5408
A03	HLA-A*31:03	677	QTNSPRRAR	0,6697
A03	HLA-A*31:03	757	GSFCTQLNR	0,5163
A03	HLA-A*31:03	975	SVLNDILSR	0,8099
A03	HLA-A*31:03	991	VQIDRLITGR	0,5083
A03	HLA-A*31:03	1099	GTHWFVTQR	0,8669
A03	HLA-A*31:04	34	RGVYYPDKVFR	0,6638
A03	HLA-A*31:04	35	GVYYPDKVFR	0,8323
A03	HLA-A*31:04	36	VYYPDKVFR	0,882
A03	HLA-A*31:04	142	GVYYHKNNK	0,5143

A03	HLA-A*31:04	150	KSWMESEFR	0,7064
A03	HLA-A*31:04	182	KQGNFKNLR	0,768
A03	HLA-A*31:04	193	VFKNIDGYFK	0,6092
A03	HLA-A*31:04	237	RFQTLLALHR	0,5604
A03	HLA-A*31:04	264	AYYVGYLQPR	0,5113
A03	HLA-A*31:04	302	TLKSFTVEK	0,6023
A03	HLA-A*31:04	310	KGIYQTSNFR	0,6
A03	HLA-A*31:04	311	GIYQTSNFR	0,7783
A03	HLA-A*31:04	319	RVQPTESIVR	0,6913
A03	HLA-A*31:04	346	RFASVYAWNR	0,73
A03	HLA-A*31:04	348	ASVYAWNRKR	0,5374
A03	HLA-A*31:04	349	SVYAWNRKR	0,8673
A03	HLA-A*31:04	394	NVYADSFVIR	0,5006
A03	HLA-A*31:04	395	VYADSFVIR	0,708
A03	HLA-A*31:04	446	GGNYNYLYR	0,7092
A03	HLA-A*31:04	449	YNYLYRLFR	0,5488
A03	HLA-A*31:04	454	RLFRKSNLK	0,6239
A03	HLA-A*31:04	457	RKSNLKPFER	0,5092
A03	HLA-A*31:04	458	KSNLKPFER	0,9139
A03	HLA-A*31:04	558	KFLPFQQFGR	0,7268
A03	HLA-A*31:04	637	STGSNVFQTR	0,6297
A03	HLA-A*31:04	638	TGSNVFQTR	0,5408
A03	HLA-A*31:04	677	QTNSPRRAR	0,6697
A03	HLA-A*31:04	757	GSFCTQLNR	0,5163
A03	HLA-A*31:04	975	SVLNDILSR	0,8099
A03	HLA-A*31:04	991	VQIDRLITGR	0,5083
A03	HLA-A*31:04	1099	GTHWFVTQR	0,8669
A03	HLA-A*31:05	35	GVYYPDKVFR	0,5445
A03	HLA-A*31:05	36	VYYPDKVFR	0,7165
A03	HLA-A*31:05	346	RFASVYAWNR	0,6108
A03	HLA-A*31:05	349	SVYAWNRKR	0,6829
A03	HLA-A*31:05	395	VYADSFVIR	0,5499
A03	HLA-A*31:05	458	KSNLKPFER	0,6747
A03	HLA-A*31:05	1099	GTHWFVTQR	0,6122
A03	HLA-A*31:06	34	RGVYYPDKVFR	0,5993
A03	HLA-A*31:06	35	GVYYPDKVFR	0,8058
A03	HLA-A*31:06	36	VYYPDKVFR	0,8626
A03	HLA-A*31:06	150	KSWMESEFR	0,6719
A03	HLA-A*31:06	182	KQGNFKNLR	0,7159
A03	HLA-A*31:06	193	VFKNIDGYFK	0,5689
A03	HLA-A*31:06	237	RFQTLLALHR	0,5435
A03	HLA-A*31:06	302	TLKSFTVEK	0,5191
A03	HLA-A*31:06	310	KGIYQTSNFR	0,5598
A03	HLA-A*31:06	311	GIYQTSNFR	0,7562
A03	HLA-A*31:06	319	RVQPTESIVR	0,6572
A03	HLA-A*31:06	346	RFASVYAWNR	0,7268
A03	HLA-A*31:06	349	SVYAWNRKR	0,8534
A03	HLA-A*31:06	395	VYADSFVIR	0,6684
A03	HLA-A*31:06	446	GGNYNYLYR	0,647
A03	HLA-A*31:06	449	YNYLYRLFR	0,5126

A03	HLA-A*31:06	454	RLFRKSNLK	0,5816
A03	HLA-A*31:06	458	KSNLKPFER	0,8983
A03	HLA-A*31:06	558	KFLPFQQFGR	0,6977
A03	HLA-A*31:06	637	STGSNVFQTR	0,5673
A03	HLA-A*31:06	677	QTNSPRRAR	0,5945
A03	HLA-A*31:06	975	SVLNDILSR	0,7759
A03	HLA-A*31:06	1099	GTHWFVTQR	0,856
A03	HLA-A*31:09	34	RGVYYPDKVFR	0,5141
A03	HLA-A*31:09	35	GVYYPDKVFR	0,663
A03	HLA-A*31:09	36	VYYPDKVFR	0,7682
A03	HLA-A*31:09	150	KSWMESEFR	0,517
A03	HLA-A*31:09	182	KQGNFKNLR	0,6571
A03	HLA-A*31:09	311	GIYQTSNFR	0,5434
A03	HLA-A*31:09	346	RFASVYAWNR	0,6162
A03	HLA-A*31:09	349	SVYAWNRKR	0,8004
A03	HLA-A*31:09	395	VYADSFVIR	0,5259
A03	HLA-A*31:09	458	KSNLKPFER	0,8223
A03	HLA-A*31:09	558	KFLPFQQFGR	0,5629
A03	HLA-A*31:09	677	QTNSPRRAR	0,5307
A03	HLA-A*31:09	975	SVLNDILSR	0,5479
A03	HLA-A*31:09	1099	GTHWFVTQR	0,7562
A03	HLA-A*31:11	34	RGVYYPDKVFR	0,5574
A03	HLA-A*31:11	35	GVYYPDKVFR	0,7294
A03	HLA-A*31:11	36	VYYPDKVFR	0,8085
A03	HLA-A*31:11	150	KSWMESEFR	0,5583
A03	HLA-A*31:11	182	KQGNFKNLR	0,6717
A03	HLA-A*31:11	310	KGIYQTSNFR	0,5075
A03	HLA-A*31:11	311	GIYQTSNFR	0,6612
A03	HLA-A*31:11	319	RVQPTESIVR	0,5738
A03	HLA-A*31:11	346	RFASVYAWNR	0,7025
A03	HLA-A*31:11	349	SVYAWNRKR	0,8646
A03	HLA-A*31:11	395	VYADSFVIR	0,575
A03	HLA-A*31:11	446	GGNYNYLYR	0,5086
A03	HLA-A*31:11	454	RLFRKSNLK	0,5237
A03	HLA-A*31:11	458	KSNLKPFER	0,8687
A03	HLA-A*31:11	558	KFLPFQQFGR	0,6235
A03	HLA-A*31:11	677	QTNSPRRAR	0,6557
A03	HLA-A*31:11	975	SVLNDILSR	0,6352
A03	HLA-A*31:11	1099	GTHWFVTQR	0,837
A03	HLA-A*33:01	36	VYYPDKVFR	0,6674
A03	HLA-A*33:01	265	YYVGYLQPR	0,6111
A03	HLA-A*33:01	349	SVYAWNRKR	0,5816
A03	HLA-A*33:01	394	NVYADSFVIR	0,575
A03	HLA-A*33:01	395	VYADSFVIR	0,5728
A03	HLA-A*33:01	448	NYNYLYRLFR	0,5281
A03	HLA-A*33:04	36	VYYPDKVFR	0,6674
A03	HLA-A*33:04	265	YYVGYLQPR	0,6111
A03	HLA-A*33:04	349	SVYAWNRKR	0,5816
A03	HLA-A*33:04	394	NVYADSFVIR	0,575
A03	HLA-A*33:04	395	VYADSFVIR	0,5728

A03	HLA-A*33:04	448	NYNYLYRLFR	0,5281
A03	HLA-A*33:05	36	VYYPDKVFR	0,6674
A03	HLA-A*33:05	265	YYVGYLQPR	0,6111
A03	HLA-A*33:05	349	SVYAWNRKR	0,5816
A03	HLA-A*33:05	394	NVYADSFVIR	0,575
A03	HLA-A*33:05	395	VYADSFVIR	0,5728
A03	HLA-A*33:05	448	NYNYLYRLFR	0,5281
A03	HLA-A*33:06	36	VYYPDKVFR	0,6561
A03	HLA-A*33:06	265	YYVGYLQPR	0,5103
A03	HLA-A*33:06	349	SVYAWNRKR	0,7608
A03	HLA-A*33:06	394	NVYADSFVIR	0,5705
A03	HLA-A*33:06	395	VYADSFVIR	0,5307
A03	HLA-A*33:06	677	QTNSPRRAR	0,5806
A03	HLA-A*33:06	975	SVLNDILSR	0,5342
A03	HLA-A*33:06	1099	GTHWFVTQR	0,5592
A03	HLA-A*33:07	36	VYYPDKVFR	0,6674
A03	HLA-A*33:07	265	YYVGYLQPR	0,6111
A03	HLA-A*33:07	349	SVYAWNRKR	0,5816
A03	HLA-A*33:07	394	NVYADSFVIR	0,575
A03	HLA-A*33:07	395	VYADSFVIR	0,5728
A03	HLA-A*33:07	448	NYNYLYRLFR	0,5281
A03	HLA-A*34:02	30	NSFTRGVYY	0,7033
A03	HLA-A*34:02	35	GVYYPDKVFR	0,5826
A03	HLA-A*34:02	69	HVSGTNGTK	0,6254
A03	HLA-A*34:02	88	DGVYFASTEK	0,5696
A03	HLA-A*34:02	89	GVYFASTEK	0,81
A03	HLA-A*34:02	142	GVYYHKNNK	0,7107
A03	HLA-A*34:02	192	FVFKNIDGY	0,5463
A03	HLA-A*34:02	198	DGYFKIYSK	0,5064
A03	HLA-A*34:02	258	WTAGAAAYY	0,585
A03	HLA-A*34:02	302	TLKSFTVEK	0,7513
A03	HLA-A*34:02	311	GIYQTSNFR	0,5989
A03	HLA-A*34:02	349	SVYAWNRKR	0,7569
A03	HLA-A*34:02	370	NSASFSTFK	0,7929
A03	HLA-A*34:02	394	NVYADSFVIR	0,553
A03	HLA-A*34:02	409	QIAPGQTGK	0,7709
A03	HLA-A*34:02	724	TEILPVSMTK	0,6467
A03	HLA-A*34:02	725	EILPVSMTK	0,8798
A03	HLA-A*34:02	782	FAQVKQIYK	0,569
A03	HLA-A*34:02	787	QIYKTPPIK	0,744
A03	HLA-A*34:02	827	TLADAGFIK	0,6884
A03	HLA-A*34:02	975	SVLNDILSR	0,7688
A03	HLA-A*34:02	1020	ASANLAATK	0,5816
A03	HLA-A*34:02	1064	HVTYVPAQEK	0,7046
A03	HLA-A*34:02	1065	VTYVPAQEK	0,8154
A03	HLA-A*34:02	1099	GTHWFVTQR	0,5926
A03	HLA-A*34:02	1173	NASVVNIQK	0,5921
A03	HLA-A*34:03	30	NSFTRGVYY	0,7033
A03	HLA-A*34:03	35	GVYYPDKVFR	0,5826
A03	HLA-A*34:03	69	HVSGTNGTK	0,6254

A03	HLA-A*34:03	88	DGVYFASTEK	0,5696
A03	HLA-A*34:03	89	GVYFASTEK	0,81
A03	HLA-A*34:03	142	GVYYHKNNK	0,7107
A03	HLA-A*34:03	192	FVFKNIDGY	0,5463
A03	HLA-A*34:03	198	DGYFKIYSK	0,5064
A03	HLA-A*34:03	258	WTAGAAAYY	0,585
A03	HLA-A*34:03	302	TLKSFTVEK	0,7513
A03	HLA-A*34:03	311	GIYQTSNFR	0,5989
A03	HLA-A*34:03	349	SVYAWNRKR	0,7569
A03	HLA-A*34:03	370	NSASFSTFK	0,7929
A03	HLA-A*34:03	394	NVYADSFVIR	0,553
A03	HLA-A*34:03	409	QIAPGQTGK	0,7709
A03	HLA-A*34:03	724	TEILPVSMTK	0,6467
A03	HLA-A*34:03	725	EILPVSMTK	0,8798
A03	HLA-A*34:03	782	FAQVKQIYK	0,569
A03	HLA-A*34:03	787	QIYKTPPIK	0,744
A03	HLA-A*34:03	827	TLADAGFIK	0,6884
A03	HLA-A*34:03	975	SVLNDILSR	0,7688
A03	HLA-A*34:03	1020	ASANLAATK	0,5816
A03	HLA-A*34:03	1064	HVTYVPAQEK	0,7046
A03	HLA-A*34:03	1065	VTYVPAQEK	0,8154
A03	HLA-A*34:03	1099	GTHWFVTQR	0,5926
A03	HLA-A*34:03	1173	NASVVNIQK	0,5921
A03	HLA-A*34:04	30	NSFTRGVYY	0,7033
A03	HLA-A*34:04	35	GVYYPDKVFR	0,5826
A03	HLA-A*34:04	69	HVSGTNGTK	0,6254
A03	HLA-A*34:04	88	DGVYFASTEK	0,5696
A03	HLA-A*34:04	89	GVYFASTEK	0,81
A03	HLA-A*34:04	142	GVYYHKNNK	0,7107
A03	HLA-A*34:04	192	FVFKNIDGY	0,5463
A03	HLA-A*34:04	198	DGYFKIYSK	0,5064
A03	HLA-A*34:04	258	WTAGAAAYY	0,585
A03	HLA-A*34:04	302	TLKSFTVEK	0,7513
A03	HLA-A*34:04	311	GIYQTSNFR	0,5989
A03	HLA-A*34:04	349	SVYAWNRKR	0,7569
A03	HLA-A*34:04	370	NSASFSTFK	0,7929
A03	HLA-A*34:04	394	NVYADSFVIR	0,553
A03	HLA-A*34:04	409	QIAPGQTGK	0,7709
A03	HLA-A*34:04	724	TEILPVSMTK	0,6467
A03	HLA-A*34:04	725	EILPVSMTK	0,8798
A03	HLA-A*34:04	782	FAQVKQIYK	0,569
A03	HLA-A*34:04	787	QIYKTPPIK	0,744
A03	HLA-A*34:04	827	TLADAGFIK	0,6884
A03	HLA-A*34:04	975	SVLNDILSR	0,7688
A03	HLA-A*34:04	1020	ASANLAATK	0,5816
A03	HLA-A*34:04	1064	HVTYVPAQEK	0,7046
A03	HLA-A*34:04	1065	VTYVPAQEK	0,8154
A03	HLA-A*34:04	1099	GTHWFVTQR	0,5926
A03	HLA-A*34:04	1173	NASVVNIQK	0,5921
A03	HLA-A*34:06	30	NSFTRGVYY	0,6353

A03	HLA-A*34:06	192	FVFKNIDGY	0,6689
A03	HLA-A*34:06	258	WTAGAAAYY	0,787
A03	HLA-A*34:06	349	SVYAWNRKR	0,6008
A03	HLA-A*34:06	360	NCVADYSVLY	0,5204
A03	HLA-A*34:06	361	CVADYSVLY	0,6106
A03	HLA-A*34:06	604	TSNQVAVLY	0,5152
A03	HLA-A*34:06	725	EILPVSMTK	0,7361
A03	HLA-A*34:06	865	LTDEMIAQY	0,516
A03	HLA-A*34:06	975	SVLNDILSR	0,591
A03	HLA-A*66:01	192	FVFKNIDGY	0,6341
A03	HLA-A*66:01	258	WTAGAAAYY	0,7115
A03	HLA-A*66:01	725	EILPVSMTK	0,6045
A03	HLA-A*66:02	69	HVSGTNGTK	0,5701
A03	HLA-A*66:02	192	FVFKNIDGY	0,6529
A03	HLA-A*66:02	258	WTAGAAAYY	0,7098
A03	HLA-A*66:02	349	SVYAWNRKR	0,538
A03	HLA-A*66:02	725	EILPVSMTK	0,694
A03	HLA-A*66:03	192	FVFKNIDGY	0,6992
A03	HLA-A*66:03	258	WTAGAAAYY	0,7192
A03	HLA-A*66:03	349	SVYAWNRKR	0,5229
A03	HLA-A*66:04	192	FVFKNIDGY	0,5137
A03	HLA-A*66:04	258	WTAGAAAYY	0,5766
A03	HLA-A*66:04	725	EILPVSMTK	0,603
A03	HLA-A*68:01	30	NSFTRGVYY	0,668
A03	HLA-A*68:01	35	GVYYPDKVFR	0,7011
A03	HLA-A*68:01	69	HVSGTNGTK	0,6905
A03	HLA-A*68:01	69	HVSGTNGTKR	0,6265
A03	HLA-A*68:01	88	DGVYFASTEK	0,6542
A03	HLA-A*68:01	89	GVYFASTEK	0,6612
A03	HLA-A*68:01	94	STEKSNIIR	0,5226
A03	HLA-A*68:01	192	FVFKNIDGY	0,6109
A03	HLA-A*68:01	198	DGYFKIYSK	0,5463
A03	HLA-A*68:01	228	DLPIGINITR	0,6101
A03	HLA-A*68:01	258	WTAGAAAYY	0,739
A03	HLA-A*68:01	302	TLKSFTVEK	0,5381
A03	HLA-A*68:01	311	GIYQTSNFR	0,6323
A03	HLA-A*68:01	347	FASVYAWNR	0,6502
A03	HLA-A*68:01	349	SVYAWNRKR	0,7833
A03	HLA-A*68:01	361	CVADYSVLY	0,538
A03	HLA-A*68:01	369	YNSASFSTFK	0,5211
A03	HLA-A*68:01	370	NSASFSTFK	0,8693
A03	HLA-A*68:01	394	NVYADSFVIR	0,8218
A03	HLA-A*68:01	400	FVIRGDEVR	0,7176
A03	HLA-A*68:01	409	QIAPGQTGK	0,6056
A03	HLA-A*68:01	501	NGVGYQPYR	0,5133
A03	HLA-A*68:01	568	DIADTTDAVR	0,5526
A03	HLA-A*68:01	604	TSNQVAVLY	0,5237
A03	HLA-A*68:01	637	STGSNVFQTR	0,6834
A03	HLA-A*68:01	677	QTNSPRRAR	0,5628
A03	HLA-A*68:01	725	EILPVSMTK	0,8883

A03	HLA-A*68:01	777	NTQEVFAQVK	0,5821
A03	HLA-A*68:01	782	FAQVKQIYK	0,557
A03	HLA-A*68:01	817	FIEDLLFNK	0,5167
A03	HLA-A*68:01	827	TLADAGFIK	0,6674
A03	HLA-A*68:01	975	SVLNDILSR	0,7755
A03	HLA-A*68:01	1020	ASANLAATK	0,5422
A03	HLA-A*68:01	1064	HVTYVPAQEK	0,6592
A03	HLA-A*68:01	1065	VTYVPAQEK	0,6604
A03	HLA-A*68:01	1099	GTHWFVTQR	0,7781
A03	HLA-A*68:01	1173	NASVVNIQK	0,8236
A03	HLA-A*68:03	30	NSFTRGVYY	0,52
A03	HLA-A*68:03	35	GVYYPDKVFR	0,583
A03	HLA-A*68:03	69	HVSGTNGTKR	0,5195
A03	HLA-A*68:03	192	FVFKNIDGY	0,5247
A03	HLA-A*68:03	228	DLPIGINITR	0,5626
A03	HLA-A*68:03	258	WTAGAAAYY	0,6587
A03	HLA-A*68:03	311	GIYQTSNFR	0,5487
A03	HLA-A*68:03	347	FASVYAWNR	0,5769
A03	HLA-A*68:03	349	SVYAWNRKR	0,6915
A03	HLA-A*68:03	370	NSASFSTFK	0,6811
A03	HLA-A*68:03	394	NVYADSFVIR	0,7431
A03	HLA-A*68:03	400	FVIRGDEVR	0,6382
A03	HLA-A*68:03	637	STGSNVFQTR	0,5981
A03	HLA-A*68:03	725	EILPVSMTK	0,7062
A03	HLA-A*68:03	975	SVLNDILSR	0,7019
A03	HLA-A*68:03	1099	GTHWFVTQR	0,6828
A03	HLA-A*68:03	1173	NASVVNIQK	0,5167
A03	HLA-A*68:04	35	GVYYPDKVFR	0,5995
A03	HLA-A*68:04	69	HVSGTNGTKR	0,5678
A03	HLA-A*68:04	228	DLPIGINITR	0,5843
A03	HLA-A*68:04	258	WTAGAAAYY	0,5018
A03	HLA-A*68:04	311	GIYQTSNFR	0,5866
A03	HLA-A*68:04	347	FASVYAWNR	0,6029
A03	HLA-A*68:04	349	SVYAWNRKR	0,7626
A03	HLA-A*68:04	370	NSASFSTFK	0,6296
A03	HLA-A*68:04	394	NVYADSFVIR	0,7377
A03	HLA-A*68:04	400	FVIRGDEVR	0,6688
A03	HLA-A*68:04	568	DIADTTDAVR	0,5213
A03	HLA-A*68:04	637	STGSNVFQTR	0,6194
A03	HLA-A*68:04	677	QTNSPRRAR	0,5976
A03	HLA-A*68:04	725	EILPVSMTK	0,6512
A03	HLA-A*68:04	975	SVLNDILSR	0,7053
A03	HLA-A*68:04	1099	GTHWFVTQR	0,6927
A03	HLA-A*68:08	30	NSFTRGVYY	0,6319
A03	HLA-A*68:08	35	GVYYPDKVFR	0,6146
A03	HLA-A*68:08	89	GVYFASTEK	0,5888
A03	HLA-A*68:08	192	FVFKNIDGY	0,5341
A03	HLA-A*68:08	258	WTAGAAAYY	0,6296
A03	HLA-A*68:08	311	GIYQTSNFR	0,5259
A03	HLA-A*68:08	349	SVYAWNRKR	0,7003

A03	HLA-A*68:08	361	CVADYSVLY	0,5107
A03	HLA-A*68:08	370	NSASFSTFK	0,7548
A03	HLA-A*68:08	394	NVYADSFVIR	0,7003
A03	HLA-A*68:08	725	EILPVSMTK	0,8497
A03	HLA-A*68:08	817	FIEDLLFNK	0,54
A03	HLA-A*68:08	827	TLADAGFIK	0,6538
A03	HLA-A*68:08	975	SVLNDILSR	0,7898
A03	HLA-A*68:08	1065	VTYVPAQEK	0,5951
A03	HLA-A*68:08	1099	GTHWFVTQR	0,627
A03	HLA-A*68:08	1173	NASVVNIQK	0,7036
A03	HLA-A*68:09	30	NSFTRGVYY	0,5551
A03	HLA-A*68:09	35	GVYYPDKVFR	0,5657
A03	HLA-A*68:09	89	GVYFASTEK	0,5852
A03	HLA-A*68:09	94	STEKSNIIR	0,5242
A03	HLA-A*68:09	258	WTAGAAAYY	0,6062
A03	HLA-A*68:09	302	TLKSFTVEK	0,5606
A03	HLA-A*68:09	311	GIYQTSNFR	0,5449
A03	HLA-A*68:09	349	SVYAWNRKR	0,6482
A03	HLA-A*68:09	370	NSASFSTFK	0,7717
A03	HLA-A*68:09	394	NVYADSFVIR	0,6549
A03	HLA-A*68:09	409	QIAPGQTGK	0,5013
A03	HLA-A*68:09	725	EILPVSMTK	0,8372
A03	HLA-A*68:09	782	FAQVKQIYK	0,5222
A03	HLA-A*68:09	817	FIEDLLFNK	0,5307
A03	HLA-A*68:09	827	TLADAGFIK	0,7002
A03	HLA-A*68:09	975	SVLNDILSR	0,773
A03	HLA-A*68:09	1065	VTYVPAQEK	0,5868
A03	HLA-A*68:09	1099	GTHWFVTQR	0,6191
A03	HLA-A*68:09	1173	NASVVNIQK	0,6784
A03	HLA-A*68:10	35	GVYYPDKVFR	0,8083
A03	HLA-A*68:10	69	HVSGTNGTK	0,53
A03	HLA-A*68:10	89	GVYFASTEK	0,8449
A03	HLA-A*68:10	142	GVYYHKNNK	0,6813
A03	HLA-A*68:10	150	KSWMESEFR	0,5571
A03	HLA-A*68:10	302	TLKSFTVEK	0,7187
A03	HLA-A*68:10	310	KGIYQTSNFR	0,5025
A03	HLA-A*68:10	311	GIYQTSNFR	0,7383
A03	HLA-A*68:10	319	RVQPTESIVR	0,5288
A03	HLA-A*68:10	348	ASVYAWNRK	0,6444
A03	HLA-A*68:10	349	SVYAWNRKR	0,7606
A03	HLA-A*68:10	369	YNSASFSTFK	0,5505
A03	HLA-A*68:10	370	NSASFSTFK	0,782
A03	HLA-A*68:10	394	NVYADSFVIR	0,5174
A03	HLA-A*68:10	408	RQIAPGQTGK	0,6091
A03	HLA-A*68:10	409	QIAPGQTGK	0,6387
A03	HLA-A*68:10	454	RLFRKSNLK	0,6427
A03	HLA-A*68:10	458	KSNLKPFER	0,7028
A03	HLA-A*68:10	529	KSTNLVKNK	0,5693
A03	HLA-A*68:10	550	GVLTESNKK	0,5463
A03	HLA-A*68:10	637	STGSNVFQTR	0,6581

A03	HLA-A*68:10	724	TEILPVSMTK	0,5558
A03	HLA-A*68:10	725	EILPVSMTK	0,6932
A03	HLA-A*68:10	757	GSFCTQLNR	0,5531
A03	HLA-A*68:10	782	FAQVKQIYK	0,5621
A03	HLA-A*68:10	787	QIYKTPPIK	0,5872
A03	HLA-A*68:10	826	VTLADAGFIK	0,6153
A03	HLA-A*68:10	827	TLADAGFIK	0,7116
A03	HLA-A*68:10	939	SSTASALGK	0,5627
A03	HLA-A*68:10	975	SVLNDILSR	0,813
A03	HLA-A*68:10	1019	RASANLAATK	0,5906
A03	HLA-A*68:10	1020	ASANLAATK	0,8515
A03	HLA-A*68:10	1064	HVTYVPAQEK	0,6145
A03	HLA-A*68:10	1065	VTYVPAQEK	0,8181
A03	HLA-A*68:10	1099	GTHWFVTQR	0,8652
A03	HLA-A*68:10	1173	NASVVNIQK	0,5721
A03	HLA-A*68:12	30	NSFTRGVYY	0,668
A03	HLA-A*68:12	35	GVYYPDKVFR	0,7011
A03	HLA-A*68:12	69	HVSGTNGTK	0,6905
A03	HLA-A*68:12	69	HVSGTNGTKR	0,6265
A03	HLA-A*68:12	88	DGVYFASTEK	0,6542
A03	HLA-A*68:12	89	GVYFASTEK	0,6612
A03	HLA-A*68:12	94	STEKSNIIR	0,5226
A03	HLA-A*68:12	192	FVFKNIDGY	0,6109
A03	HLA-A*68:12	198	DGYFKIYSK	0,5463
A03	HLA-A*68:12	228	DLPIGINITR	0,6101
A03	HLA-A*68:12	258	WTAGAAAYY	0,739
A03	HLA-A*68:12	302	TLKSFTVEK	0,5381
A03	HLA-A*68:12	311	GIYQTSNFR	0,6323
A03	HLA-A*68:12	347	FASVYAWNR	0,6502
A03	HLA-A*68:12	349	SVYAWNRKR	0,7833
A03	HLA-A*68:12	361	CVADYSVLY	0,538
A03	HLA-A*68:12	369	YNSASFSTFK	0,5211
A03	HLA-A*68:12	370	NSASFSTFK	0,8693
A03	HLA-A*68:12	394	NVYADSFVIR	0,8218
A03	HLA-A*68:12	400	FVIRGDEVR	0,7176
A03	HLA-A*68:12	409	QIAPGQTGK	0,6056
A03	HLA-A*68:12	501	NGVGYQPYR	0,5133
A03	HLA-A*68:12	568	DIADTTDAVR	0,5526
A03	HLA-A*68:12	604	TSNQVAVLY	0,5237
A03	HLA-A*68:12	637	STGSNVFQTR	0,6834
A03	HLA-A*68:12	677	QTNSPRRAR	0,5628
A03	HLA-A*68:12	725	EILPVSMTK	0,8883
A03	HLA-A*68:12	777	NTQEVFAQVK	0,5821
A03	HLA-A*68:12	782	FAQVKQIYK	0,557
A03	HLA-A*68:12	817	FIEDLLFNK	0,5167
A03	HLA-A*68:12	827	TLADAGFIK	0,6674
A03	HLA-A*68:12	975	SVLNDILSR	0,7755
A03	HLA-A*68:12	1020	ASANLAATK	0,5422
A03	HLA-A*68:12	1064	HVTYVPAQEK	0,6592
A03	HLA-A*68:12	1065	VTYVPAQEK	0,6604

A03	HLA-A*68:12	1099	GTHWFVTQR	0,7781
A03	HLA-A*68:12	1173	NASVVNIQK	0,8236
A03	HLA-A*68:13	35	GVYYPDKVFR	0,8316
A03	HLA-A*68:13	69	HVSGTNGTK	0,5191
A03	HLA-A*68:13	89	GVYFASTEK	0,8528
A03	HLA-A*68:13	142	GVYYHKNNK	0,6657
A03	HLA-A*68:13	258	WTAGAAAYY	0,5568
A03	HLA-A*68:13	302	TLKSFTVEK	0,7097
A03	HLA-A*68:13	311	GIYQTSNFR	0,7417
A03	HLA-A*68:13	348	ASVYAWNRK	0,588
A03	HLA-A*68:13	349	SVYAWNRKR	0,7769
A03	HLA-A*68:13	369	YNSASFSTFK	0,5945
A03	HLA-A*68:13	370	NSASFSTFK	0,7984
A03	HLA-A*68:13	394	NVYADSFVIR	0,6004
A03	HLA-A*68:13	400	FVIRGDEVR	0,5034
A03	HLA-A*68:13	409	QIAPGQTGK	0,6035
A03	HLA-A*68:13	454	RLFRKSNLK	0,5075
A03	HLA-A*68:13	458	KSNLKPFER	0,5406
A03	HLA-A*68:13	637	STGSNVFQTR	0,6827
A03	HLA-A*68:13	724	TEILPVSMTK	0,541
A03	HLA-A*68:13	725	EILPVSMTK	0,7319
A03	HLA-A*68:13	757	GSFCTQLNR	0,5489
A03	HLA-A*68:13	782	FAQVKQIYK	0,5799
A03	HLA-A*68:13	787	QIYKTPPIK	0,5619
A03	HLA-A*68:13	826	VTLADAGFIK	0,6185
A03	HLA-A*68:13	827	TLADAGFIK	0,6995
A03	HLA-A*68:13	939	SSTASALGK	0,5057
A03	HLA-A*68:13	975	SVLNDILSR	0,8131
A03	HLA-A*68:13	1020	ASANLAATK	0,8167
A03	HLA-A*68:13	1064	HVTYVPAQEK	0,6451
A03	HLA-A*68:13	1065	VTYVPAQEK	0,823
A03	HLA-A*68:13	1099	GTHWFVTQR	0,8791
A03	HLA-A*68:13	1173	NASVVNIQK	0,58
A03	HLA-A*68:14	35	GVYYPDKVFR	0,8083
A03	HLA-A*68:14	69	HVSGTNGTK	0,53
A03	HLA-A*68:14	89	GVYFASTEK	0,8449
A03	HLA-A*68:14	142	GVYYHKNNK	0,6813
A03	HLA-A*68:14	150	KSWMESEFR	0,5571
A03	HLA-A*68:14	302	TLKSFTVEK	0,7187
A03	HLA-A*68:14	310	KGIYQTSNFR	0,5025
A03	HLA-A*68:14	311	GIYQTSNFR	0,7383
A03	HLA-A*68:14	319	RVQPTESIVR	0,5288
A03	HLA-A*68:14	348	ASVYAWNRK	0,6444
A03	HLA-A*68:14	349	SVYAWNRKR	0,7606
A03	HLA-A*68:14	369	YNSASFSTFK	0,5505
A03	HLA-A*68:14	370	NSASFSTFK	0,782
A03	HLA-A*68:14	394	NVYADSFVIR	0,5174
A03	HLA-A*68:14	408	RQIAPGQTGK	0,6091
A03	HLA-A*68:14	409	QIAPGQTGK	0,6387
A03	HLA-A*68:14	454	RLFRKSNLK	0,6427

A03	HLA-A*68:14	458	KSNLKPFER	0,7028
A03	HLA-A*68:14	529	KSTNLVKNK	0,5693
A03	HLA-A*68:14	550	GVLTESNKK	0,5463
A03	HLA-A*68:14	637	STGSNVFQTR	0,6581
A03	HLA-A*68:14	724	TEILPVSMTK	0,5558
A03	HLA-A*68:14	725	EILPVSMTK	0,6932
A03	HLA-A*68:14	757	GSFCTQLNR	0,5531
A03	HLA-A*68:14	782	FAQVKQIYK	0,5621
A03	HLA-A*68:14	787	QIYKTPPIK	0,5872
A03	HLA-A*68:14	826	VTLADAGFIK	0,6153
A03	HLA-A*68:14	827	TLADAGFIK	0,7116
A03	HLA-A*68:14	939	SSTASALGK	0,5627
A03	HLA-A*68:14	975	SVLNDILSR	0,813
A03	HLA-A*68:14	1019	RASANLAATK	0,5906
A03	HLA-A*68:14	1020	ASANLAATK	0,8515
A03	HLA-A*68:14	1064	HVTYVPAQEK	0,6145
A03	HLA-A*68:14	1065	VTYVPAQEK	0,8181
A03	HLA-A*68:14	1099	GTHWFVTQR	0,8652
A03	HLA-A*68:14	1173	NASVVNIQK	0,5721
A03	HLA-A*68:16	30	NSFTRGVYY	0,668
A03	HLA-A*68:16	35	GVYYPDKVFR	0,7011
A03	HLA-A*68:16	69	HVSGTNGTK	0,6905
A03	HLA-A*68:16	69	HVSGTNGTKR	0,6265
A03	HLA-A*68:16	88	DGVYFASTEK	0,6542
A03	HLA-A*68:16	89	GVYFASTEK	0,6612
A03	HLA-A*68:16	94	STEKSNIIR	0,5226
A03	HLA-A*68:16	192	FVFKNIDGY	0,6109
A03	HLA-A*68:16	198	DGYFKIYSK	0,5463
A03	HLA-A*68:16	228	DLPIGINITR	0,6101
A03	HLA-A*68:16	258	WTAGAAAYY	0,739
A03	HLA-A*68:16	302	TLKSFTVEK	0,5381
A03	HLA-A*68:16	311	GIYQTSNFR	0,6323
A03	HLA-A*68:16	347	FASVYAWNR	0,6502
A03	HLA-A*68:16	349	SVYAWNRKR	0,7833
A03	HLA-A*68:16	361	CVADYSVLY	0,538
A03	HLA-A*68:16	369	YNSASFSTFK	0,5211
A03	HLA-A*68:16	370	NSASFSTFK	0,8693
A03	HLA-A*68:16	394	NVYADSFVIR	0,8218
A03	HLA-A*68:16	400	FVIRGDEVR	0,7176
A03	HLA-A*68:16	409	QIAPGQTGK	0,6056
A03	HLA-A*68:16	501	NGVGYQPYR	0,5133
A03	HLA-A*68:16	568	DIADTTDAVR	0,5526
A03	HLA-A*68:16	604	TSNQVAVLY	0,5237
A03	HLA-A*68:16	637	STGSNVFQTR	0,6834
A03	HLA-A*68:16	677	QTNSPRRAR	0,5628
A03	HLA-A*68:16	725	EILPVSMTK	0,8883
A03	HLA-A*68:16	777	NTQEVFAQVK	0,5821
A03	HLA-A*68:16	782	FAQVKQIYK	0,557
A03	HLA-A*68:16	817	FIEDLLFNK	0,5167
A03	HLA-A*68:16	827	TLADAGFIK	0,6674

A03	HLA-A*68:16	975	SVLNDILSR	0,7755
A03	HLA-A*68:16	1020	ASANLAATK	0,5422
A03	HLA-A*68:16	1064	HVTYVPAQEK	0,6592
A03	HLA-A*68:16	1065	VTYVPAQEK	0,6604
A03	HLA-A*68:16	1099	GTHWFVTQR	0,7781
A03	HLA-A*68:16	1173	NASVVNIQK	0,8236
A03	HLA-A*68:19	30	NSFTRGVYY	0,668
A03	HLA-A*68:19	35	GVYYPDKVFR	0,7011
A03	HLA-A*68:19	69	HVSGTNGTK	0,6905
A03	HLA-A*68:19	69	HVSGTNGTKR	0,6265
A03	HLA-A*68:19	88	DGVYFASTEK	0,6542
A03	HLA-A*68:19	89	GVYFASTEK	0,6612
A03	HLA-A*68:19	94	STEKSNIIR	0,5226
A03	HLA-A*68:19	192	FVFKNIDGY	0,6109
A03	HLA-A*68:19	198	DGYFKIYSK	0,5463
A03	HLA-A*68:19	228	DLPIGINITR	0,6101
A03	HLA-A*68:19	258	WTAGAAAYY	0,739
A03	HLA-A*68:19	302	TLKSFTVEK	0,5381
A03	HLA-A*68:19	311	GIYQTSNFR	0,6323
A03	HLA-A*68:19	347	FASVYAWNR	0,6502
A03	HLA-A*68:19	349	SVYAWNRKR	0,7833
A03	HLA-A*68:19	361	CVADYSVLY	0,538
A03	HLA-A*68:19	369	YNSASFSTFK	0,5211
A03	HLA-A*68:19	370	NSASFSTFK	0,8693
A03	HLA-A*68:19	394	NVYADSFVIR	0,8218
A03	HLA-A*68:19	400	FVIRGDEVR	0,7176
A03	HLA-A*68:19	409	QIAPGQTGK	0,6056
A03	HLA-A*68:19	501	NGVGYQPYR	0,5133
A03	HLA-A*68:19	568	DIADTTDAVR	0,5526
A03	HLA-A*68:19	604	TSNQVAVLY	0,5237
A03	HLA-A*68:19	637	STGSNVFQTR	0,6834
A03	HLA-A*68:19	677	QTNSPRRAR	0,5628
A03	HLA-A*68:19	725	EILPVSMTK	0,8883
A03	HLA-A*68:19	777	NTQEVFAQVK	0,5821
A03	HLA-A*68:19	782	FAQVKQIYK	0,557
A03	HLA-A*68:19	817	FIEDLLFNK	0,5167
A03	HLA-A*68:19	827	TLADAGFIK	0,6674
A03	HLA-A*68:19	975	SVLNDILSR	0,7755
A03	HLA-A*68:19	1020	ASANLAATK	0,5422
A03	HLA-A*68:19	1064	HVTYVPAQEK	0,6592
A03	HLA-A*68:19	1065	VTYVPAQEK	0,6604
A03	HLA-A*68:19	1099	GTHWFVTQR	0,7781
A03	HLA-A*68:19	1173	NASVVNIQK	0,8236
A03	HLA-A*68:21	30	NSFTRGVYY	0,668
A03	HLA-A*68:21	35	GVYYPDKVFR	0,7011
A03	HLA-A*68:21	69	HVSGTNGTK	0,6905
A03	HLA-A*68:21	69	HVSGTNGTKR	0,6265
A03	HLA-A*68:21	88	DGVYFASTEK	0,6542
A03	HLA-A*68:21	89	GVYFASTEK	0,6612
A03	HLA-A*68:21	94	STEKSNIIR	0,5226

A03	HLA-A*68:21	192	FVFKNIDGY	0,6109
A03	HLA-A*68:21	198	DGYFKIYSK	0,5463
A03	HLA-A*68:21	228	DLPIGINITR	0,6101
A03	HLA-A*68:21	258	WTAGAAAYY	0,739
A03	HLA-A*68:21	302	TLKSFTVEK	0,5381
A03	HLA-A*68:21	311	GIYQTSNFR	0,6323
A03	HLA-A*68:21	347	FASVYAWNR	0,6502
A03	HLA-A*68:21	349	SVYAWNRKR	0,7833
A03	HLA-A*68:21	361	CVADYSVLY	0,538
A03	HLA-A*68:21	369	YNSASFSTFK	0,5211
A03	HLA-A*68:21	370	NSASFSTFK	0,8693
A03	HLA-A*68:21	394	NVYADSFVIR	0,8218
A03	HLA-A*68:21	400	FVIRGDEVR	0,7176
A03	HLA-A*68:21	409	QIAPGQTGK	0,6056
A03	HLA-A*68:21	501	NGVGYQPYR	0,5133
A03	HLA-A*68:21	568	DIADTTDAVR	0,5526
A03	HLA-A*68:21	604	TSNQVAVLY	0,5237
A03	HLA-A*68:21	637	STGSNVFQTR	0,6834
A03	HLA-A*68:21	677	QTNSPRRAR	0,5628
A03	HLA-A*68:21	725	EILPVSMTK	0,8883
A03	HLA-A*68:21	777	NTQEVFAQVK	0,5821
A03	HLA-A*68:21	782	FAQVKQIYK	0,557
A03	HLA-A*68:21	817	FIEDLLFNK	0,5167
A03	HLA-A*68:21	827	TLADAGFIK	0,6674
A03	HLA-A*68:21	975	SVLNDILSR	0,7755
A03	HLA-A*68:21	1020	ASANLAATK	0,5422
A03	HLA-A*68:21	1064	HVTYVPAQEK	0,6592
A03	HLA-A*68:21	1065	VTYVPAQEK	0,6604
A03	HLA-A*68:21	1099	GTHWFVTQR	0,7781
A03	HLA-A*68:21	1173	NASVVNIQK	0,8236
A03	HLA-A*68:22	30	NSFTRGVYY	0,668
A03	HLA-A*68:22	35	GVYYPDKVFR	0,7011
A03	HLA-A*68:22	69	HVSGTNGTK	0,6905
A03	HLA-A*68:22	69	HVSGTNGTKR	0,6265
A03	HLA-A*68:22	88	DGVYFASTEK	0,6542
A03	HLA-A*68:22	89	GVYFASTEK	0,6612
A03	HLA-A*68:22	94	STEKSNIIR	0,5226
A03	HLA-A*68:22	192	FVFKNIDGY	0,6109
A03	HLA-A*68:22	198	DGYFKIYSK	0,5463
A03	HLA-A*68:22	228	DLPIGINITR	0,6101
A03	HLA-A*68:22	258	WTAGAAAYY	0,739
A03	HLA-A*68:22	302	TLKSFTVEK	0,5381
A03	HLA-A*68:22	311	GIYQTSNFR	0,6323
A03	HLA-A*68:22	347	FASVYAWNR	0,6502
A03	HLA-A*68:22	349	SVYAWNRKR	0,7833
A03	HLA-A*68:22	361	CVADYSVLY	0,538
A03	HLA-A*68:22	369	YNSASFSTFK	0,5211
A03	HLA-A*68:22	370	NSASFSTFK	0,8693
A03	HLA-A*68:22	394	NVYADSFVIR	0,8218
A03	HLA-A*68:22	400	FVIRGDEVR	0,7176

A03	HLA-A*68:22	409	QIAPGQTGK	0,6056
A03	HLA-A*68:22	501	NGVGYQPYR	0,5133
A03	HLA-A*68:22	568	DIADTTDAVR	0,5526
A03	HLA-A*68:22	604	TSNQVAVLY	0,5237
A03	HLA-A*68:22	637	STGSNVFQTR	0,6834
A03	HLA-A*68:22	677	QTNSPRRAR	0,5628
A03	HLA-A*68:22	725	EILPVSMTK	0,8883
A03	HLA-A*68:22	777	NTQEVFAQVK	0,5821
A03	HLA-A*68:22	782	FAQVKQIYK	0,557
A03	HLA-A*68:22	817	FIEDLLFNK	0,5167
A03	HLA-A*68:22	827	TLADAGFIK	0,6674
A03	HLA-A*68:22	975	SVLNDILSR	0,7755
A03	HLA-A*68:22	1020	ASANLAATK	0,5422
A03	HLA-A*68:22	1064	HVTYVPAQEK	0,6592
A03	HLA-A*68:22	1065	VTYVPAQEK	0,6604
A03	HLA-A*68:22	1099	GTHWFVTQR	0,7781
A03	HLA-A*68:22	1173	NASVVNIQK	0,8236
A03	HLA-A*68:24	30	NSFTRGVYY	0,668
A03	HLA-A*68:24	35	GVYYPDKVFR	0,7011
A03	HLA-A*68:24	69	HVSGTNGTK	0,6905
A03	HLA-A*68:24	69	HVSGTNGTKR	0,6265
A03	HLA-A*68:24	88	DGVYFASTEK	0,6542
A03	HLA-A*68:24	89	GVYFASTEK	0,6612
A03	HLA-A*68:24	94	STEKSNIIR	0,5226
A03	HLA-A*68:24	192	FVFKNIDGY	0,6109
A03	HLA-A*68:24	198	DGYFKIYSK	0,5463
A03	HLA-A*68:24	228	DLPIGINITR	0,6101
A03	HLA-A*68:24	258	WTAGAAAYY	0,739
A03	HLA-A*68:24	302	TLKSFTVEK	0,5381
A03	HLA-A*68:24	311	GIYQTSNFR	0,6323
A03	HLA-A*68:24	347	FASVYAWNR	0,6502
A03	HLA-A*68:24	349	SVYAWNRKR	0,7833
A03	HLA-A*68:24	361	CVADYSVLY	0,538
A03	HLA-A*68:24	369	YNSASFSTFK	0,5211
A03	HLA-A*68:24	370	NSASFSTFK	0,8693
A03	HLA-A*68:24	394	NVYADSFVIR	0,8218
A03	HLA-A*68:24	400	FVIRGDEVR	0,7176
A03	HLA-A*68:24	409	QIAPGQTGK	0,6056
A03	HLA-A*68:24	501	NGVGYQPYR	0,5133
A03	HLA-A*68:24	568	DIADTTDAVR	0,5526
A03	HLA-A*68:24	604	TSNQVAVLY	0,5237
A03	HLA-A*68:24	637	STGSNVFQTR	0,6834
A03	HLA-A*68:24	677	QTNSPRRAR	0,5628
A03	HLA-A*68:24	725	EILPVSMTK	0,8883
A03	HLA-A*68:24	777	NTQEVFAQVK	0,5821
A03	HLA-A*68:24	782	FAQVKQIYK	0,557
A03	HLA-A*68:24	817	FIEDLLFNK	0,5167
A03	HLA-A*68:24	827	TLADAGFIK	0,6674
A03	HLA-A*68:24	975	SVLNDILSR	0,7755
A03	HLA-A*68:24	1020	ASANLAATK	0,5422

A03	HLA-A*68:24	1064	HVTYVPAQEK	0,6592
A03	HLA-A*68:24	1065	VTYVPAQEK	0,6604
A03	HLA-A*68:24	1099	GTHWFVTQR	0,7781
A03	HLA-A*68:24	1173	NASVVNIQK	0,8236
A03	HLA-A*68:25	30	NSFTRGVYY	0,668
A03	HLA-A*68:25	35	GVYYPDKVFR	0,7011
A03	HLA-A*68:25	69	HVSGTNGTK	0,6905
A03	HLA-A*68:25	69	HVSGTNGTKR	0,6265
A03	HLA-A*68:25	88	DGVYFASTEK	0,6542
A03	HLA-A*68:25	89	GVYFASTEK	0,6612
A03	HLA-A*68:25	94	STEKSNIIR	0,5226
A03	HLA-A*68:25	192	FVFKNIDGY	0,6109
A03	HLA-A*68:25	198	DGYFKIYSK	0,5463
A03	HLA-A*68:25	228	DLPIGINITR	0,6101
A03	HLA-A*68:25	258	WTAGAAAYY	0,739
A03	HLA-A*68:25	302	TLKSFTVEK	0,5381
A03	HLA-A*68:25	311	GIYQTSNFR	0,6323
A03	HLA-A*68:25	347	FASVYAWNR	0,6502
A03	HLA-A*68:25	349	SVYAWNRKR	0,7833
A03	HLA-A*68:25	361	CVADYSVLY	0,538
A03	HLA-A*68:25	369	YNSASFSTFK	0,5211
A03	HLA-A*68:25	370	NSASFSTFK	0,8693
A03	HLA-A*68:25	394	NVYADSFVIR	0,8218
A03	HLA-A*68:25	400	FVIRGDEVR	0,7176
A03	HLA-A*68:25	409	QIAPGQTGK	0,6056
A03	HLA-A*68:25	501	NGVGYQPYR	0,5133
A03	HLA-A*68:25	568	DIADTTDAVR	0,5526
A03	HLA-A*68:25	604	TSNQVAVLY	0,5237
A03	HLA-A*68:25	637	STGSNVFQTR	0,6834
A03	HLA-A*68:25	677	QTNSPRRAR	0,5628
A03	HLA-A*68:25	725	EILPVSMTK	0,8883
A03	HLA-A*68:25	777	NTQEVFAQVK	0,5821
A03	HLA-A*68:25	782	FAQVKQIYK	0,557
A03	HLA-A*68:25	817	FIEDLLFNK	0,5167
A03	HLA-A*68:25	827	TLADAGFIK	0,6674
A03	HLA-A*68:25	975	SVLNDILSR	0,7755
A03	HLA-A*68:25	1020	ASANLAATK	0,5422
A03	HLA-A*68:25	1064	HVTYVPAQEK	0,6592
A03	HLA-A*68:25	1065	VTYVPAQEK	0,6604
A03	HLA-A*68:25	1099	GTHWFVTQR	0,7781
A03	HLA-A*68:25	1173	NASVVNIQK	0,8236
A03	HLA-A*68:26	258	WTAGAAAYY	0,5231
A03	HLA-A*68:26	370	NSASFSTFK	0,5588
A03	HLA-A*68:26	725	EILPVSMTK	0,699
A03	HLA-A*68:26	827	TLADAGFIK	0,5161
A03	HLA-A*74:01	349	SVYAWNRKR	0,536
A03	HLA-A*74:02	349	SVYAWNRKR	0,536
A03	HLA-A*74:03	349	SVYAWNRKR	0,536
A03	HLA-A*74:05	35	GVYYPDKVFR	0,5051
A03	HLA-A*74:05	349	SVYAWNRKR	0,5895

A03	HLA-A*74:05	454	RLFRKSNLK	0,5208
A03	HLA-A*74:05	1099	GTHWFVTQR	0,5226
A03	HLA-A*74:07	35	GVYYPDKVFR	0,5118
A03	HLA-A*74:07	349	SVYAWNRKR	0,6319
A03	HLA-A*74:07	1099	GTHWFVTQR	0,5316
A03	HLA-A*74:08	349	SVYAWNRKR	0,536
A03	HLA-A*74:09	349	SVYAWNRKR	0,536
A03	HLA-A*74:11	349	SVYAWNRKR	0,536
A24	HLA-A*23:01	57	PFFSNVTWF	0,9141
A24	HLA-A*23:01	78	RFDNPVLPF	0,8396
A24	HLA-A*23:01	143	VYYHKNNKSW	0,7142
A24	HLA-A*23:01	144	YYHKNNKSW	0,8036
A24	HLA-A*23:01	159	VYSSANNCTF	0,917
A24	HLA-A*23:01	169	EYVSQPFLM	0,8499
A24	HLA-A*23:01	193	VFKNIDGYF	0,7818
A24	HLA-A*23:01	203	IYSKHTPINL	0,6028
A24	HLA-A*23:01	264	AYYVGYLQPRTF	0,7912
A24	HLA-A*23:01	265	YYVGYLQPRTF	0,879
A24	HLA-A*23:01	268	GYLQPRTFLL	0,7046
A24	HLA-A*23:01	269	YLQPRTFLL	0,5573
A24	HLA-A*23:01	312	IYQTSNFRV	0,6966
A24	HLA-A*23:01	328	RFPNITNLCPF	0,8158
A24	HLA-A*23:01	368	LYNSASFSTF	0,8553
A24	HLA-A*23:01	395	VYADSFVI	0,5149
A24	HLA-A*23:01	448	NYNYLYRLF	0,8881
A24	HLA-A*23:01	488	CYFPLQSYGF	0,6136
A24	HLA-A*23:01	489	YFPLQSYGF	0,9108
A24	HLA-A*23:01	504	GYQPYRVVVLSF	0,667
A24	HLA-A*23:01	507	PYRVVVLSF	0,8298
A24	HLA-A*23:01	558	KFLPFQQF	0,7881
A24	HLA-A*23:01	634	RVYSTGSNVF	0,5782
A24	HLA-A*23:01	635	VYSTGSNVF	0,934
A24	HLA-A*23:01	788	IYKTPPIKDF	0,9066
A24	HLA-A*23:01	816	SFIEDLLF	0,7909
A24	HLA-A*23:01	880	GTITSGWTF	0,5317
A24	HLA-A*23:01	898	FAMQMAYRF	0,5028
A24	HLA-A*23:01	1066	TYVPAQEKNF	0,9478
A24	HLA-A*23:01	1094	VFVSNGTHW	0,8761
A24	HLA-A*23:01	1094	VFVSNGTHWF	0,8258
A24	HLA-A*23:01	1101	HWFVTQRNF	0,7845
A24	HLA-A*23:01	1137	VYDPLQPELDSF	0,7515
A24	HLA-A*23:01	1208	QYIKWPWYI	0,9638
A24	HLA-A*23:01	1208	QYIKWPWYIW	0,7814
A24	HLA-A*23:01	1211	KWPWYIWLGF	0,6037
A24	HLA-A*23:01	1216	IWLGFIAGL	0,5925
A24	HLA-A*23:02	57	PFFSNVTWF	0,8811
A24	HLA-A*23:02	143	VYYHKNNKSW	0,7728
A24	HLA-A*23:02	144	YYHKNNKSW	0,8613
A24	HLA-A*23:02	159	VYSSANNCTF	0,9503
A24	HLA-A*23:02	169	EYVSQPFLM	0,8797

A24	HLA-A*23:02	193	VFKNIDGYF	0,8456
A24	HLA-A*23:02	203	IYSKHTPINL	0,6962
A24	HLA-A*23:02	264	AYYVGYLQPRTF	0,759
A24	HLA-A*23:02	265	YYVGYLQPRTF	0,9004
A24	HLA-A*23:02	268	GYLQPRTFLL	0,6697
A24	HLA-A*23:02	269	YLQPRTFLL	0,5368
A24	HLA-A*23:02	312	IYQTSNFRV	0,6415
A24	HLA-A*23:02	328	RFPNITNLCPF	0,8669
A24	HLA-A*23:02	368	LYNSASFSTF	0,901
A24	HLA-A*23:02	448	NYNYLYRLF	0,9202
A24	HLA-A*23:02	488	CYFPLQSYGF	0,6273
A24	HLA-A*23:02	489	YFPLQSYGF	0,9384
A24	HLA-A*23:02	504	GYQPYRVVVLSF	0,6274
A24	HLA-A*23:02	507	PYRVVVLSF	0,8272
A24	HLA-A*23:02	634	RVYSTGSNVF	0,7093
A24	HLA-A*23:02	635	VYSTGSNVF	0,9475
A24	HLA-A*23:02	755	QYGSFCTQL	0,5573
A24	HLA-A*23:02	788	IYKTPPIKDF	0,9187
A24	HLA-A*23:02	1066	TYVPAQEKNF	0,9671
A24	HLA-A*23:02	1094	VFVSNGTHW	0,8829
A24	HLA-A*23:02	1094	VFVSNGTHWF	0,8701
A24	HLA-A*23:02	1101	HWFVTQRNF	0,7966
A24	HLA-A*23:02	1208	QYIKWPWYI	0,9423
A24	HLA-A*23:02	1208	QYIKWPWYIW	0,7679
A24	HLA-A*23:02	1211	KWPWYIWLGF	0,5976
A24	HLA-A*23:03	57	PFFSNVTWF	0,9141
A24	HLA-A*23:03	78	RFDNPVLPF	0,8396
A24	HLA-A*23:03	143	VYYHKNNKSW	0,7142
A24	HLA-A*23:03	144	YYHKNNKSW	0,8036
A24	HLA-A*23:03	159	VYSSANNCTF	0,917
A24	HLA-A*23:03	169	EYVSQPFLM	0,8499
A24	HLA-A*23:03	193	VFKNIDGYF	0,7818
A24	HLA-A*23:03	203	IYSKHTPINL	0,6028
A24	HLA-A*23:03	264	AYYVGYLQPRTF	0,7912
A24	HLA-A*23:03	265	YYVGYLQPRTF	0,879
A24	HLA-A*23:03	268	GYLQPRTFLL	0,7046
A24	HLA-A*23:03	269	YLQPRTFLL	0,5573
A24	HLA-A*23:03	312	IYQTSNFRV	0,6966
A24	HLA-A*23:03	328	RFPNITNLCPF	0,8158
A24	HLA-A*23:03	368	LYNSASFSTF	0,8553
A24	HLA-A*23:03	395	VYADSFVI	0,5149
A24	HLA-A*23:03	448	NYNYLYRLF	0,8881
A24	HLA-A*23:03	488	CYFPLQSYGF	0,6136
A24	HLA-A*23:03	489	YFPLQSYGF	0,9108
A24	HLA-A*23:03	504	GYQPYRVVVLSF	0,667
A24	HLA-A*23:03	507	PYRVVVLSF	0,8298
A24	HLA-A*23:03	558	KFLPFQQF	0,7881
A24	HLA-A*23:03	634	RVYSTGSNVF	0,5782
A24	HLA-A*23:03	635	VYSTGSNVF	0,934
A24	HLA-A*23:03	788	IYKTPPIKDF	0,9066

A24	HLA-A*23:03	816	SFIEDLLF	0,7909
A24	HLA-A*23:03	880	GTITSGWTF	0,5317
A24	HLA-A*23:03	898	FAMQMAYRF	0,5028
A24	HLA-A*23:03	1066	TYVPAQEKNF	0,9478
A24	HLA-A*23:03	1094	VFVSNGTHW	0,8761
A24	HLA-A*23:03	1094	VFVSNGTHWF	0,8258
A24	HLA-A*23:03	1101	HWFVTQRNF	0,7845
A24	HLA-A*23:03	1137	VYDPLQPELDSF	0,7515
A24	HLA-A*23:03	1208	QYIKWPWYI	0,9638
A24	HLA-A*23:03	1208	QYIKWPWYIW	0,7814
A24	HLA-A*23:03	1211	KWPWYIWLGF	0,6037
A24	HLA-A*23:03	1216	IWLGFIAGL	0,5925
A24	HLA-A*23:04	36	VYYPDKVF	0,9008
A24	HLA-A*23:04	37	YYPDKVFRSSVL	0,5679
A24	HLA-A*23:04	57	PFFSNVTWF	0,9013
A24	HLA-A*23:04	77	KRFDNPVLPF	0,6675
A24	HLA-A*23:04	78	RFDNPVLPF	0,9308
A24	HLA-A*23:04	143	VYYHKNNKSW	0,7934
A24	HLA-A*23:04	144	YYHKNNKSW	0,9179
A24	HLA-A*23:04	151	SWMESEFRV	0,667
A24	HLA-A*23:04	159	VYSSANNCTF	0,9321
A24	HLA-A*23:04	167	TFEYVSQPF	0,5826
A24	HLA-A*23:04	168	FEYVSQPFLM	0,5386
A24	HLA-A*23:04	169	EYVSQPFLM	0,8708
A24	HLA-A*23:04	193	VFKNIDGYF	0,8925
A24	HLA-A*23:04	203	IYSKHTPINL	0,679
A24	HLA-A*23:04	247	SYLTPGDSSSGW	0,6431
A24	HLA-A*23:04	264	AYYVGYLQPRTF	0,8651
A24	HLA-A*23:04	265	YYVGYLQPRTF	0,926
A24	HLA-A*23:04	265	YYVGYLQPRTFL	0,5396
A24	HLA-A*23:04	267	VGYLQPRTFL	0,6172
A24	HLA-A*23:04	268	GYLQPRTF	0,7597
A24	HLA-A*23:04	268	GYLQPRTFL	0,9029
A24	HLA-A*23:04	268	GYLQPRTFLL	0,8435
A24	HLA-A*23:04	269	YLQPRTFLL	0,5793
A24	HLA-A*23:04	312	IYQTSNFRV	0,807
A24	HLA-A*23:04	328	RFPNITNLCPF	0,8874
A24	HLA-A*23:04	345	TRFASVYAW	0,5445
A24	HLA-A*23:04	346	RFASVYAW	0,6882
A24	HLA-A*23:04	368	LYNSASFSTF	0,8917
A24	HLA-A*23:04	395	VYADSFVI	0,5709
A24	HLA-A*23:04	448	NYNYLYRLF	0,8977
A24	HLA-A*23:04	488	CYFPLQSYGF	0,7405
A24	HLA-A*23:04	489	YFPLQSYGF	0,9665
A24	HLA-A*23:04	504	GYQPYRVVV	0,6984
A24	HLA-A*23:04	504	GYQPYRVVVL	0,6433
A24	HLA-A*23:04	504	GYQPYRVVVLSF	0,8025
A24	HLA-A*23:04	507	PYRVVVLSF	0,8366
A24	HLA-A*23:04	558	KFLPFQQF	0,8975
A24	HLA-A*23:04	634	RVYSTGSNVF	0,7676

A24	HLA-A*23:04	635	VYSTGSNVF	0,9621
A24	HLA-A*23:04	659	SYECDIPIGAGI	0,5668
A24	HLA-A*23:04	706	AYSNNSIAI	0,7351
A24	HLA-A*23:04	755	QYGSFCTQL	0,6733
A24	HLA-A*23:04	788	IYKTPPIKDF	0,9258
A24	HLA-A*23:04	816	SFIEDLLF	0,8315
A24	HLA-A*23:04	880	GTITSGWTF	0,6365
A24	HLA-A*23:04	898	FAMQMAYRF	0,5384
A24	HLA-A*23:04	1051	SFPQSAPHGVVF	0,7156
A24	HLA-A*23:04	1066	TYVPAQEKNF	0,9578
A24	HLA-A*23:04	1094	VFVSNGTHW	0,9161
A24	HLA-A*23:04	1094	VFVSNGTHWF	0,829
A24	HLA-A*23:04	1101	HWFVTQRNF	0,9099
A24	HLA-A*23:04	1137	VYDPLQPEL	0,9435
A24	HLA-A*23:04	1137	VYDPLQPELDSF	0,802
A24	HLA-A*23:04	1147	SFKEELDKYF	0,5695
A24	HLA-A*23:04	1205	KYEQYIKW	0,612
A24	HLA-A*23:04	1208	QYIKWPWYI	0,9656
A24	HLA-A*23:04	1208	QYIKWPWYIW	0,7453
A24	HLA-A*23:04	1211	KWPWYIWLGF	0,7165
A24	HLA-A*23:04	1216	IWLGFIAGL	0,7942
A24	HLA-A*23:04	1219	GFIAGLIAI	0,5361
A24	HLA-A*23:06	57	PFFSNVTWF	0,9141
A24	HLA-A*23:06	78	RFDNPVLPF	0,8396
A24	HLA-A*23:06	143	VYYHKNNKSW	0,7142
A24	HLA-A*23:06	144	YYHKNNKSW	0,8036
A24	HLA-A*23:06	159	VYSSANNCTF	0,917
A24	HLA-A*23:06	169	EYVSQPFLM	0,8499
A24	HLA-A*23:06	193	VFKNIDGYF	0,7818
A24	HLA-A*23:06	203	IYSKHTPINL	0,6028
A24	HLA-A*23:06	264	AYYVGYLQPRTF	0,7912
A24	HLA-A*23:06	265	YYVGYLQPRTF	0,879
A24	HLA-A*23:06	268	GYLQPRTFLL	0,7046
A24	HLA-A*23:06	269	YLQPRTFLL	0,5573
A24	HLA-A*23:06	312	IYQTSNFRV	0,6966
A24	HLA-A*23:06	328	RFPNITNLCPF	0,8158
A24	HLA-A*23:06	368	LYNSASFSTF	0,8553
A24	HLA-A*23:06	395	VYADSFVI	0,5149
A24	HLA-A*23:06	448	NYNYLYRLF	0,8881
A24	HLA-A*23:06	488	CYFPLQSYGF	0,6136
A24	HLA-A*23:06	489	YFPLQSYGF	0,9108
A24	HLA-A*23:06	504	GYQPYRVVVLSF	0,667
A24	HLA-A*23:06	507	PYRVVVLSF	0,8298
A24	HLA-A*23:06	558	KFLPFQQF	0,7881
A24	HLA-A*23:06	634	RVYSTGSNVF	0,5782
A24	HLA-A*23:06	635	VYSTGSNVF	0,934
A24	HLA-A*23:06	788	IYKTPPIKDF	0,9066
A24	HLA-A*23:06	816	SFIEDLLF	0,7909
A24	HLA-A*23:06	880	GTITSGWTF	0,5317
A24	HLA-A*23:06	898	FAMQMAYRF	0,5028

A24	HLA-A*23:06	1066	TYVPAQEKNF	0,9478
A24	HLA-A*23:06	1094	VFVSNGTHW	0,8761
A24	HLA-A*23:06	1094	VFVSNGTHWF	0,8258
A24	HLA-A*23:06	1101	HWFVTQRNF	0,7845
A24	HLA-A*23:06	1137	VYDPLQPELDSF	0,7515
A24	HLA-A*23:06	1208	QYIKWPWYI	0,9638
A24	HLA-A*23:06	1208	QYIKWPWYIW	0,7814
A24	HLA-A*23:06	1211	KWPWYIWLGF	0,6037
A24	HLA-A*23:06	1216	IWLGFIAGL	0,5925
A24	HLA-A*23:10	57	PFFSNVTWF	0,8852
A24	HLA-A*23:10	78	RFDNPVLPF	0,8057
A24	HLA-A*23:10	159	VYSSANNCTF	0,8567
A24	HLA-A*23:10	169	EYVSQPFLM	0,8502
A24	HLA-A*23:10	264	AYYVGYLQPRTF	0,6408
A24	HLA-A*23:10	265	YYVGYLQPRTF	0,7982
A24	HLA-A*23:10	268	GYLQPRTFLL	0,6349
A24	HLA-A*23:10	269	YLQPRTFLL	0,5339
A24	HLA-A*23:10	312	IYQTSNFRV	0,5674
A24	HLA-A*23:10	328	RFPNITNLCPF	0,6995
A24	HLA-A*23:10	368	LYNSASFSTF	0,7785
A24	HLA-A*23:10	448	NYNYLYRLF	0,8162
A24	HLA-A*23:10	488	CYFPLQSYGF	0,5004
A24	HLA-A*23:10	489	YFPLQSYGF	0,8984
A24	HLA-A*23:10	504	GYQPYRVVVLSF	0,5485
A24	HLA-A*23:10	507	PYRVVVLSF	0,7181
A24	HLA-A*23:10	635	VYSTGSNVF	0,8708
A24	HLA-A*23:10	816	SFIEDLLF	0,7518
A24	HLA-A*23:10	880	GTITSGWTF	0,6006
A24	HLA-A*23:10	898	FAMQMAYRF	0,5102
A24	HLA-A*23:10	1066	TYVPAQEKNF	0,9105
A24	HLA-A*23:10	1094	VFVSNGTHW	0,8044
A24	HLA-A*23:10	1094	VFVSNGTHWF	0,7522
A24	HLA-A*23:10	1101	HWFVTQRNF	0,6918
A24	HLA-A*23:10	1137	VYDPLQPELDSF	0,682
A24	HLA-A*23:10	1208	QYIKWPWYI	0,9438
A24	HLA-A*23:10	1208	QYIKWPWYIW	0,6787
A24	HLA-A*23:10	1216	IWLGFIAGL	0,5657
A24	HLA-A*24:02	37	YYPDKVFRSSVL	0,601
A24	HLA-A*24:02	57	PFFSNVTWF	0,8728
A24	HLA-A*24:02	78	RFDNPVLPF	0,8538
A24	HLA-A*24:02	143	VYYHKNNKSW	0,7378
A24	HLA-A*24:02	144	YYHKNNKSW	0,8416
A24	HLA-A*24:02	151	SWMESEFRV	0,5046
A24	HLA-A*24:02	159	VYSSANNCTF	0,9582
A24	HLA-A*24:02	167	TFEYVSQPF	0,5365
A24	HLA-A*24:02	169	EYVSQPFLM	0,8484
A24	HLA-A*24:02	203	IYSKHTPINL	0,7085
A24	HLA-A*24:02	264	AYYVGYLQPRTF	0,8037
A24	HLA-A*24:02	265	YYVGYLQPRTF	0,9005
A24	HLA-A*24:02	268	GYLQPRTFLL	0,731

A24	HLA-A*24:02	269	YLQPRTFLL	0,5359
A24	HLA-A*24:02	312	IYQTSNFRV	0,785
A24	HLA-A*24:02	328	RFPNITNLCPF	0,8789
A24	HLA-A*24:02	346	RFASVYAW	0,521
A24	HLA-A*24:02	368	LYNSASFSTF	0,914
A24	HLA-A*24:02	395	VYADSFVI	0,6394
A24	HLA-A*24:02	448	NYNYLYRLF	0,9353
A24	HLA-A*24:02	488	CYFPLQSYGF	0,5885
A24	HLA-A*24:02	489	YFPLQSYGF	0,9442
A24	HLA-A*24:02	504	GYQPYRVVVLSF	0,6707
A24	HLA-A*24:02	507	PYRVVVLSF	0,8166
A24	HLA-A*24:02	558	KFLPFQQF	0,6948
A24	HLA-A*24:02	634	RVYSTGSNVF	0,7146
A24	HLA-A*24:02	635	VYSTGSNVF	0,9696
A24	HLA-A*24:02	755	QYGSFCTQL	0,5614
A24	HLA-A*24:02	788	IYKTPPIKDF	0,9226
A24	HLA-A*24:02	816	SFIEDLLF	0,7253
A24	HLA-A*24:02	1066	TYVPAQEKNF	0,9479
A24	HLA-A*24:02	1094	VFVSNGTHW	0,8658
A24	HLA-A*24:02	1094	VFVSNGTHWF	0,8335
A24	HLA-A*24:02	1101	HWFVTQRNF	0,754
A24	HLA-A*24:02	1137	VYDPLQPELDSF	0,8174
A24	HLA-A*24:02	1208	QYIKWPWYI	0,9613
A24	HLA-A*24:02	1208	QYIKWPWYIW	0,7582
A24	HLA-A*24:02	1211	KWPWYIWLGF	0,6582
A24	HLA-A*24:03	36	VYYPDKVF	0,8969
A24	HLA-A*24:03	37	YYPDKVFRSSVL	0,7149
A24	HLA-A*24:03	57	PFFSNVTWF	0,8822
A24	HLA-A*24:03	77	KRFDNPVLPF	0,6134
A24	HLA-A*24:03	78	RFDNPVLPF	0,9523
A24	HLA-A*24:03	143	VYYHKNNKSW	0,8351
A24	HLA-A*24:03	144	YYHKNNKSW	0,9523
A24	HLA-A*24:03	151	SWMESEFRV	0,7048
A24	HLA-A*24:03	159	VYSSANNCTF	0,9732
A24	HLA-A*24:03	167	TFEYVSQPF	0,6889
A24	HLA-A*24:03	169	EYVSQPFLM	0,8798
A24	HLA-A*24:03	193	VFKNIDGYF	0,8984
A24	HLA-A*24:03	203	IYSKHTPINL	0,8029
A24	HLA-A*24:03	247	SYLTPGDSSSGW	0,7197
A24	HLA-A*24:03	264	AYYVGYLQPRTF	0,8966
A24	HLA-A*24:03	265	YYVGYLQPRTF	0,9506
A24	HLA-A*24:03	265	YYVGYLQPRTFL	0,6242
A24	HLA-A*24:03	267	VGYLQPRTFL	0,7184
A24	HLA-A*24:03	268	GYLQPRTF	0,7874
A24	HLA-A*24:03	268	GYLQPRTFL	0,9337
A24	HLA-A*24:03	268	GYLQPRTFLL	0,8923
A24	HLA-A*24:03	312	IYQTSNFRV	0,8841
A24	HLA-A*24:03	328	RFPNITNLCPF	0,9389
A24	HLA-A*24:03	346	RFASVYAW	0,7335
A24	HLA-A*24:03	350	VYAWNRKRI	0,6686

A24	HLA-A*24:03	368	LYNSASFSTF	0,9453
A24	HLA-A*24:03	379	CYGVSPTKL	0,6202
A24	HLA-A*24:03	395	VYADSFVI	0,6956
A24	HLA-A*24:03	448	NYNYLYRLF	0,9379
A24	HLA-A*24:03	488	CYFPLQSYGF	0,7404
A24	HLA-A*24:03	489	YFPLQSYGF	0,9778
A24	HLA-A*24:03	504	GYQPYRVVV	0,7982
A24	HLA-A*24:03	504	GYQPYRVVVL	0,7444
A24	HLA-A*24:03	504	GYQPYRVVVLSF	0,8581
A24	HLA-A*24:03	507	PYRVVVLSF	0,8533
A24	HLA-A*24:03	558	KFLPFQQF	0,8636
A24	HLA-A*24:03	632	TWRVYSTGSNVF	0,5957
A24	HLA-A*24:03	634	RVYSTGSNVF	0,8822
A24	HLA-A*24:03	635	VYSTGSNVF	0,9872
A24	HLA-A*24:03	659	SYECDIPIGAGI	0,6187
A24	HLA-A*24:03	706	AYSNNSIAI	0,8828
A24	HLA-A*24:03	755	QYGSFCTQL	0,7929
A24	HLA-A*24:03	788	IYKTPPIKDF	0,9538
A24	HLA-A*24:03	816	SFIEDLLF	0,8244
A24	HLA-A*24:03	1051	SFPQSAPHGVVF	0,8397
A24	HLA-A*24:03	1066	TYVPAQEKNF	0,97
A24	HLA-A*24:03	1094	VFVSNGTHW	0,9343
A24	HLA-A*24:03	1094	VFVSNGTHWF	0,8723
A24	HLA-A*24:03	1101	HWFVTQRNF	0,9047
A24	HLA-A*24:03	1137	VYDPLQPEL	0,9496
A24	HLA-A*24:03	1137	VYDPLQPELDSF	0,8822
A24	HLA-A*24:03	1147	SFKEELDKYF	0,6438
A24	HLA-A*24:03	1205	KYEQYIKW	0,6328
A24	HLA-A*24:03	1208	QYIKWPWYI	0,9753
A24	HLA-A*24:03	1208	QYIKWPWYIW	0,7703
A24	HLA-A*24:03	1211	KWPWYIWLGF	0,7747
A24	HLA-A*24:03	1216	IWLGFIAGL	0,7773
A24	HLA-A*24:05	37	YYPDKVFRSSVL	0,601
A24	HLA-A*24:05	57	PFFSNVTWF	0,8728
A24	HLA-A*24:05	78	RFDNPVLPF	0,8538
A24	HLA-A*24:05	143	VYYHKNNKSW	0,7378
A24	HLA-A*24:05	144	YYHKNNKSW	0,8416
A24	HLA-A*24:05	151	SWMESEFRV	0,5046
A24	HLA-A*24:05	159	VYSSANNCTF	0,9582
A24	HLA-A*24:05	167	TFEYVSQPF	0,5365
A24	HLA-A*24:05	169	EYVSQPFLM	0,8484
A24	HLA-A*24:05	203	IYSKHTPINL	0,7085
A24	HLA-A*24:05	264	AYYVGYLQPRTF	0,8037
A24	HLA-A*24:05	265	YYVGYLQPRTF	0,9005
A24	HLA-A*24:05	268	GYLQPRTFLL	0,731
A24	HLA-A*24:05	269	YLQPRTFLL	0,5359
A24	HLA-A*24:05	312	IYQTSNFRV	0,785
A24	HLA-A*24:05	328	RFPNITNLCPF	0,8789
A24	HLA-A*24:05	346	RFASVYAW	0,521
A24	HLA-A*24:05	368	LYNSASFSTF	0,914

A24	HLA-A*24:05	395	VYADSFVI	0,6394
A24	HLA-A*24:05	448	NYNYLYRLF	0,9353
A24	HLA-A*24:05	488	CYFPLQSYGF	0,5885
A24	HLA-A*24:05	489	YFPLQSYGF	0,9442
A24	HLA-A*24:05	504	GYQPYRVVVLSF	0,6707
A24	HLA-A*24:05	507	PYRVVVLSF	0,8166
A24	HLA-A*24:05	558	KFLPFQQF	0,6948
A24	HLA-A*24:05	634	RVYSTGSNVF	0,7146
A24	HLA-A*24:05	635	VYSTGSNVF	0,9696
A24	HLA-A*24:05	755	QYGSFCTQL	0,5614
A24	HLA-A*24:05	788	IYKTPPIKDF	0,9226
A24	HLA-A*24:05	816	SFIEDLLF	0,7253
A24	HLA-A*24:05	1066	TYVPAQEKNF	0,9479
A24	HLA-A*24:05	1094	VFVSNGTHW	0,8658
A24	HLA-A*24:05	1094	VFVSNGTHWF	0,8335
A24	HLA-A*24:05	1101	HWFVTQRNF	0,754
A24	HLA-A*24:05	1137	VYDPLQPELDSF	0,8174
A24	HLA-A*24:05	1208	QYIKWPWYI	0,9613
A24	HLA-A*24:05	1208	QYIKWPWYIW	0,7582
A24	HLA-A*24:05	1211	KWPWYIWLGF	0,6582
A24	HLA-A*24:06	57	PFFSNVTWF	0,8811
A24	HLA-A*24:06	143	VYYHKNNKSW	0,7728
A24	HLA-A*24:06	144	YYHKNNKSW	0,8613
A24	HLA-A*24:06	159	VYSSANNCTF	0,9503
A24	HLA-A*24:06	169	EYVSQPFLM	0,8797
A24	HLA-A*24:06	193	VFKNIDGYF	0,8456
A24	HLA-A*24:06	203	IYSKHTPINL	0,6962
A24	HLA-A*24:06	264	AYYVGYLQPRTF	0,759
A24	HLA-A*24:06	265	YYVGYLQPRTF	0,9004
A24	HLA-A*24:06	268	GYLQPRTFLL	0,6697
A24	HLA-A*24:06	269	YLQPRTFLL	0,5368
A24	HLA-A*24:06	312	IYQTSNFRV	0,6415
A24	HLA-A*24:06	328	RFPNITNLCPF	0,8669
A24	HLA-A*24:06	368	LYNSASFSTF	0,901
A24	HLA-A*24:06	448	NYNYLYRLF	0,9202
A24	HLA-A*24:06	488	CYFPLQSYGF	0,6273
A24	HLA-A*24:06	489	YFPLQSYGF	0,9384
A24	HLA-A*24:06	504	GYQPYRVVVLSF	0,6274
A24	HLA-A*24:06	507	PYRVVVLSF	0,8272
A24	HLA-A*24:06	634	RVYSTGSNVF	0,7093
A24	HLA-A*24:06	635	VYSTGSNVF	0,9475
A24	HLA-A*24:06	755	QYGSFCTQL	0,5573
A24	HLA-A*24:06	788	IYKTPPIKDF	0,9187
A24	HLA-A*24:06	1066	TYVPAQEKNF	0,9671
A24	HLA-A*24:06	1094	VFVSNGTHW	0,8829
A24	HLA-A*24:06	1094	VFVSNGTHWF	0,8701
A24	HLA-A*24:06	1101	HWFVTQRNF	0,7966
A24	HLA-A*24:06	1208	QYIKWPWYI	0,9423
A24	HLA-A*24:06	1208	QYIKWPWYIW	0,7679
A24	HLA-A*24:06	1211	KWPWYIWLGF	0,5976

A24	HLA-A*24:08	144	YYHKNNKSW	0,7358
A24	HLA-A*24:08	159	VYSSANNCTF	0,8525
A24	HLA-A*24:08	265	YYVGYLQPRTF	0,757
A24	HLA-A*24:08	268	GYLQPRTFLL	0,6018
A24	HLA-A*24:08	269	YLQPRTFLL	0,6645
A24	HLA-A*24:08	312	IYQTSNFRV	0,6407
A24	HLA-A*24:08	328	RFPNITNLCPF	0,7387
A24	HLA-A*24:08	368	LYNSASFSTF	0,7867
A24	HLA-A*24:08	448	NYNYLYRLF	0,827
A24	HLA-A*24:08	489	YFPLQSYGF	0,8868
A24	HLA-A*24:08	635	VYSTGSNVF	0,8901
A24	HLA-A*24:08	1094	VFVSNGTHWF	0,7487
A24	HLA-A*24:08	1208	QYIKWPWYI	0,9046
A24	HLA-A*24:08	1208	QYIKWPWYIW	0,6181
A24	HLA-A*24:10	23	QLPPAYTNSF	0,6067
A24	HLA-A*24:10	36	VYYPDKVF	0,8127
A24	HLA-A*24:10	37	YYPDKVFRSSVL	0,7336
A24	HLA-A*24:10	57	PFFSNVTWF	0,8735
A24	HLA-A*24:10	77	KRFDNPVLPF	0,571
A24	HLA-A*24:10	78	RFDNPVLPF	0,9417
A24	HLA-A*24:10	143	VYYHKNNKSW	0,6813
A24	HLA-A*24:10	144	YYHKNNKSW	0,898
A24	HLA-A*24:10	151	SWMESEFRV	0,7017
A24	HLA-A*24:10	159	VYSSANNCTF	0,9516
A24	HLA-A*24:10	167	TFEYVSQPF	0,6744
A24	HLA-A*24:10	168	FEYVSQPFLM	0,5891
A24	HLA-A*24:10	169	EYVSQPFLM	0,8711
A24	HLA-A*24:10	193	VFKNIDGYF	0,8319
A24	HLA-A*24:10	203	IYSKHTPINL	0,7032
A24	HLA-A*24:10	247	SYLTPGDSSSGW	0,6388
A24	HLA-A*24:10	264	AYYVGYLQPRTF	0,8043
A24	HLA-A*24:10	265	YYVGYLQPRTF	0,8996
A24	HLA-A*24:10	265	YYVGYLQPRTFL	0,5933
A24	HLA-A*24:10	267	VGYLQPRTFL	0,6401
A24	HLA-A*24:10	268	GYLQPRTF	0,6374
A24	HLA-A*24:10	268	GYLQPRTFL	0,8962
A24	HLA-A*24:10	268	GYLQPRTFLL	0,8395
A24	HLA-A*24:10	269	YLQPRTFLL	0,5904
A24	HLA-A*24:10	312	IYQTSNFRV	0,8099
A24	HLA-A*24:10	328	RFPNITNLCPF	0,8972
A24	HLA-A*24:10	346	RFASVYAW	0,5964
A24	HLA-A*24:10	368	LYNSASFSTF	0,9153
A24	HLA-A*24:10	395	VYADSFVI	0,6432
A24	HLA-A*24:10	448	NYNYLYRLF	0,9031
A24	HLA-A*24:10	488	CYFPLQSYGF	0,6933
A24	HLA-A*24:10	489	YFPLQSYGF	0,9707
A24	HLA-A*24:10	504	GYQPYRVVV	0,6833
A24	HLA-A*24:10	504	GYQPYRVVVL	0,6625
A24	HLA-A*24:10	504	GYQPYRVVVLSF	0,7723
A24	HLA-A*24:10	507	PYRVVVLSF	0,7928

A24	HLA-A*24:10	558	KFLPFQQF	0,7572
A24	HLA-A*24:10	634	RVYSTGSNVF	0,7497
A24	HLA-A*24:10	635	VYSTGSNVF	0,9761
A24	HLA-A*24:10	659	SYECDIPIGAGI	0,5966
A24	HLA-A*24:10	706	AYSNNSIAI	0,8424
A24	HLA-A*24:10	755	QYGSFCTQL	0,7571
A24	HLA-A*24:10	788	IYKTPPIKDF	0,8924
A24	HLA-A*24:10	816	SFIEDLLF	0,8218
A24	HLA-A*24:10	880	GTITSGWTF	0,6067
A24	HLA-A*24:10	1051	SFPQSAPHGVVF	0,8219
A24	HLA-A*24:10	1066	TYVPAQEKNF	0,9499
A24	HLA-A*24:10	1094	VFVSNGTHW	0,8862
A24	HLA-A*24:10	1094	VFVSNGTHWF	0,8275
A24	HLA-A*24:10	1101	HWFVTQRNF	0,8486
A24	HLA-A*24:10	1137	VYDPLQPEL	0,9463
A24	HLA-A*24:10	1137	VYDPLQPELDSF	0,8552
A24	HLA-A*24:10	1147	SFKEELDKYF	0,621
A24	HLA-A*24:10	1208	QYIKWPWYI	0,9619
A24	HLA-A*24:10	1208	QYIKWPWYIW	0,6962
A24	HLA-A*24:10	1211	KWPWYIWLGF	0,6791
A24	HLA-A*24:10	1216	IWLGFIAGL	0,748
A24	HLA-A*24:13	57	PFFSNVTWF	0,9141
A24	HLA-A*24:13	78	RFDNPVLPF	0,8396
A24	HLA-A*24:13	143	VYYHKNNKSW	0,7142
A24	HLA-A*24:13	144	YYHKNNKSW	0,8036
A24	HLA-A*24:13	159	VYSSANNCTF	0,917
A24	HLA-A*24:13	169	EYVSQPFLM	0,8499
A24	HLA-A*24:13	193	VFKNIDGYF	0,7818
A24	HLA-A*24:13	203	IYSKHTPINL	0,6028
A24	HLA-A*24:13	264	AYYVGYLQPRTF	0,7912
A24	HLA-A*24:13	265	YYVGYLQPRTF	0,879
A24	HLA-A*24:13	268	GYLQPRTFLL	0,7046
A24	HLA-A*24:13	269	YLQPRTFLL	0,5573
A24	HLA-A*24:13	312	IYQTSNFRV	0,6966
A24	HLA-A*24:13	328	RFPNITNLCPF	0,8158
A24	HLA-A*24:13	368	LYNSASFSTF	0,8553
A24	HLA-A*24:13	395	VYADSFVI	0,5149
A24	HLA-A*24:13	448	NYNYLYRLF	0,8881
A24	HLA-A*24:13	488	CYFPLQSYGF	0,6136
A24	HLA-A*24:13	489	YFPLQSYGF	0,9108
A24	HLA-A*24:13	504	GYQPYRVVVLSF	0,667
A24	HLA-A*24:13	507	PYRVVVLSF	0,8298
A24	HLA-A*24:13	558	KFLPFQQF	0,7881
A24	HLA-A*24:13	634	RVYSTGSNVF	0,5782
A24	HLA-A*24:13	635	VYSTGSNVF	0,934
A24	HLA-A*24:13	788	IYKTPPIKDF	0,9066
A24	HLA-A*24:13	816	SFIEDLLF	0,7909
A24	HLA-A*24:13	880	GTITSGWTF	0,5317
A24	HLA-A*24:13	898	FAMQMAYRF	0,5028
A24	HLA-A*24:13	1066	TYVPAQEKNF	0,9478

A24	HLA-A*24:13	1094	VFVSNGTHW	0,8761
A24	HLA-A*24:13	1094	VFVSNGTHWF	0,8258
A24	HLA-A*24:13	1101	HWFVTQRNF	0,7845
A24	HLA-A*24:13	1137	VYDPLQPELDSF	0,7515
A24	HLA-A*24:13	1208	QYIKWPWYI	0,9638
A24	HLA-A*24:13	1208	QYIKWPWYIW	0,7814
A24	HLA-A*24:13	1211	KWPWYIWLGF	0,6037
A24	HLA-A*24:13	1216	IWLGFIAGL	0,5925
A24	HLA-A*24:18	36	VYYPDKVF	0,8173
A24	HLA-A*24:18	57	PFFSNVTWF	0,7076
A24	HLA-A*24:18	77	KRFDNPVLPF	0,5982
A24	HLA-A*24:18	78	RFDNPVLPF	0,8452
A24	HLA-A*24:18	143	VYYHKNNKSW	0,6438
A24	HLA-A*24:18	144	YYHKNNKSW	0,7986
A24	HLA-A*24:18	159	VYSSANNCTF	0,8212
A24	HLA-A*24:18	169	EYVSQPFLM	0,6396
A24	HLA-A*24:18	193	VFKNIDGYF	0,8479
A24	HLA-A*24:18	203	IYSKHTPINL	0,5908
A24	HLA-A*24:18	264	AYYVGYLQPRTF	0,7906
A24	HLA-A*24:18	265	YYVGYLQPRTF	0,7951
A24	HLA-A*24:18	267	VGYLQPRTFL	0,5749
A24	HLA-A*24:18	268	GYLQPRTF	0,7049
A24	HLA-A*24:18	268	GYLQPRTFL	0,8826
A24	HLA-A*24:18	268	GYLQPRTFLL	0,7029
A24	HLA-A*24:18	312	IYQTSNFRV	0,625
A24	HLA-A*24:18	328	RFPNITNLCPF	0,6955
A24	HLA-A*24:18	350	VYAWNRKRI	0,5642
A24	HLA-A*24:18	368	LYNSASFSTF	0,7395
A24	HLA-A*24:18	448	NYNYLYRLF	0,8086
A24	HLA-A*24:18	488	CYFPLQSYGF	0,5236
A24	HLA-A*24:18	489	YFPLQSYGF	0,8335
A24	HLA-A*24:18	504	GYQPYRVVV	0,659
A24	HLA-A*24:18	504	GYQPYRVVVL	0,5063
A24	HLA-A*24:18	504	GYQPYRVVVLSF	0,6043
A24	HLA-A*24:18	507	PYRVVVLSF	0,7472
A24	HLA-A*24:18	558	KFLPFQQF	0,7919
A24	HLA-A*24:18	634	RVYSTGSNVF	0,6968
A24	HLA-A*24:18	635	VYSTGSNVF	0,9155
A24	HLA-A*24:18	706	AYSNNSIAI	0,6343
A24	HLA-A*24:18	755	QYGSFCTQL	0,5402
A24	HLA-A*24:18	788	IYKTPPIKDF	0,8671
A24	HLA-A*24:18	1066	TYVPAQEKNF	0,7987
A24	HLA-A*24:18	1094	VFVSNGTHW	0,7787
A24	HLA-A*24:18	1094	VFVSNGTHWF	0,6654
A24	HLA-A*24:18	1101	HWFVTQRNF	0,8544
A24	HLA-A*24:18	1137	VYDPLQPEL	0,8216
A24	HLA-A*24:18	1208	QYIKWPWYI	0,8782
A24	HLA-A*24:18	1211	KWPWYIWLGF	0,5123
A24	HLA-A*24:18	1216	IWLGFIAGL	0,7231
A24	HLA-A*24:20	37	YYPDKVFRSSVL	0,601

A24	HLA-A*24:20	57	PFFSNVTWF	0,8728
A24	HLA-A*24:20	78	RFDNPVLPF	0,8538
A24	HLA-A*24:20	143	VYYHKNNKSW	0,7378
A24	HLA-A*24:20	144	YYHKNNKSW	0,8416
A24	HLA-A*24:20	151	SWMESEFRV	0,5046
A24	HLA-A*24:20	159	VYSSANNCTF	0,9582
A24	HLA-A*24:20	167	TFEYVSQPF	0,5365
A24	HLA-A*24:20	169	EYVSQPFLM	0,8484
A24	HLA-A*24:20	203	IYSKHTPINL	0,7085
A24	HLA-A*24:20	264	AYYVGYLQPRTF	0,8037
A24	HLA-A*24:20	265	YYVGYLQPRTF	0,9005
A24	HLA-A*24:20	268	GYLQPRTFLL	0,731
A24	HLA-A*24:20	269	YLQPRTFLL	0,5359
A24	HLA-A*24:20	312	IYQTSNFRV	0,785
A24	HLA-A*24:20	328	RFPNITNLCPF	0,8789
A24	HLA-A*24:20	346	RFASVYAW	0,521
A24	HLA-A*24:20	368	LYNSASFSTF	0,914
A24	HLA-A*24:20	395	VYADSFVI	0,6394
A24	HLA-A*24:20	448	NYNYLYRLF	0,9353
A24	HLA-A*24:20	488	CYFPLQSYGF	0,5885
A24	HLA-A*24:20	489	YFPLQSYGF	0,9442
A24	HLA-A*24:20	504	GYQPYRVVVLSF	0,6707
A24	HLA-A*24:20	507	PYRVVVLSF	0,8166
A24	HLA-A*24:20	558	KFLPFQQF	0,6948
A24	HLA-A*24:20	634	RVYSTGSNVF	0,7146
A24	HLA-A*24:20	635	VYSTGSNVF	0,9696
A24	HLA-A*24:20	755	QYGSFCTQL	0,5614
A24	HLA-A*24:20	788	IYKTPPIKDF	0,9226
A24	HLA-A*24:20	816	SFIEDLLF	0,7253
A24	HLA-A*24:20	1066	TYVPAQEKNF	0,9479
A24	HLA-A*24:20	1094	VFVSNGTHW	0,8658
A24	HLA-A*24:20	1094	VFVSNGTHWF	0,8335
A24	HLA-A*24:20	1101	HWFVTQRNF	0,754
A24	HLA-A*24:20	1137	VYDPLQPELDSF	0,8174
A24	HLA-A*24:20	1208	QYIKWPWYI	0,9613
A24	HLA-A*24:20	1208	QYIKWPWYIW	0,7582
A24	HLA-A*24:20	1211	KWPWYIWLGF	0,6582
A24	HLA-A*24:21	37	YYPDKVFRSSVL	0,601
A24	HLA-A*24:21	57	PFFSNVTWF	0,8728
A24	HLA-A*24:21	78	RFDNPVLPF	0,8538
A24	HLA-A*24:21	143	VYYHKNNKSW	0,7378
A24	HLA-A*24:21	144	YYHKNNKSW	0,8416
A24	HLA-A*24:21	151	SWMESEFRV	0,5046
A24	HLA-A*24:21	159	VYSSANNCTF	0,9582
A24	HLA-A*24:21	167	TFEYVSQPF	0,5365
A24	HLA-A*24:21	169	EYVSQPFLM	0,8484
A24	HLA-A*24:21	203	IYSKHTPINL	0,7085
A24	HLA-A*24:21	264	AYYVGYLQPRTF	0,8037
A24	HLA-A*24:21	265	YYVGYLQPRTF	0,9005
A24	HLA-A*24:21	268	GYLQPRTFLL	0,731

A24	HLA-A*24:21	269	YLQPRTFLL	0,5359
A24	HLA-A*24:21	312	IYQTSNFRV	0,785
A24	HLA-A*24:21	328	RFPNITNLCPF	0,8789
A24	HLA-A*24:21	346	RFASVYAW	0,521
A24	HLA-A*24:21	368	LYNSASFSTF	0,914
A24	HLA-A*24:21	395	VYADSFVI	0,6394
A24	HLA-A*24:21	448	NYNYLYRLF	0,9353
A24	HLA-A*24:21	488	CYFPLQSYGF	0,5885
A24	HLA-A*24:21	489	YFPLQSYGF	0,9442
A24	HLA-A*24:21	504	GYQPYRVVVLSF	0,6707
A24	HLA-A*24:21	507	PYRVVVLSF	0,8166
A24	HLA-A*24:21	558	KFLPFQQF	0,6948
A24	HLA-A*24:21	634	RVYSTGSNVF	0,7146
A24	HLA-A*24:21	635	VYSTGSNVF	0,9696
A24	HLA-A*24:21	755	QYGSFCTQL	0,5614
A24	HLA-A*24:21	788	IYKTPPIKDF	0,9226
A24	HLA-A*24:21	816	SFIEDLLF	0,7253
A24	HLA-A*24:21	1066	TYVPAQEKNF	0,9479
A24	HLA-A*24:21	1094	VFVSNGTHW	0,8658
A24	HLA-A*24:21	1094	VFVSNGTHWF	0,8335
A24	HLA-A*24:21	1101	HWFVTQRNF	0,754
A24	HLA-A*24:21	1137	VYDPLQPELDSF	0,8174
A24	HLA-A*24:21	1208	QYIKWPWYI	0,9613
A24	HLA-A*24:21	1208	QYIKWPWYIW	0,7582
A24	HLA-A*24:21	1211	KWPWYIWLGF	0,6582
A24	HLA-A*24:22	23	QLPPAYTNSF	0,6934
A24	HLA-A*24:22	36	VYYPDKVF	0,8478
A24	HLA-A*24:22	37	YYPDKVFRSSVL	0,692
A24	HLA-A*24:22	47	VLHSTQDLF	0,6614
A24	HLA-A*24:22	57	PFFSNVTWF	0,8749
A24	HLA-A*24:22	77	KRFDNPVLPF	0,6655
A24	HLA-A*24:22	78	RFDNPVLPF	0,861
A24	HLA-A*24:22	143	VYYHKNNKSW	0,8066
A24	HLA-A*24:22	144	YYHKNNKSW	0,9429
A24	HLA-A*24:22	151	SWMESEFRV	0,6473
A24	HLA-A*24:22	159	VYSSANNCTF	0,9584
A24	HLA-A*24:22	167	TFEYVSQPF	0,6382
A24	HLA-A*24:22	169	EYVSQPFLM	0,8978
A24	HLA-A*24:22	193	VFKNIDGYF	0,9224
A24	HLA-A*24:22	203	IYSKHTPINL	0,7532
A24	HLA-A*24:22	247	SYLTPGDSSSGW	0,7072
A24	HLA-A*24:22	264	AYYVGYLQPRTF	0,8149
A24	HLA-A*24:22	265	YYVGYLQPRTF	0,9218
A24	HLA-A*24:22	268	GYLQPRTFL	0,8871
A24	HLA-A*24:22	268	GYLQPRTFLL	0,7943
A24	HLA-A*24:22	269	YLQPRTFLL	0,6428
A24	HLA-A*24:22	312	IYQTSNFRV	0,7597
A24	HLA-A*24:22	328	RFPNITNLCPF	0,8917
A24	HLA-A*24:22	346	RFASVYAW	0,6744
A24	HLA-A*24:22	350	VYAWNRKRI	0,6447

A24	HLA-A*24:22	368	LYNSASFSTF	0,9108
A24	HLA-A*24:22	369	YNSASFSTF	0,6618
A24	HLA-A*24:22	379	CYGVSPTKL	0,6159
A24	HLA-A*24:22	448	NYNYLYRLF	0,9199
A24	HLA-A*24:22	488	CYFPLQSYGF	0,766
A24	HLA-A*24:22	489	YFPLQSYGF	0,9702
A24	HLA-A*24:22	504	GYQPYRVVV	0,6915
A24	HLA-A*24:22	504	GYQPYRVVVL	0,6647
A24	HLA-A*24:22	504	GYQPYRVVVLSF	0,7575
A24	HLA-A*24:22	507	PYRVVVLSF	0,8211
A24	HLA-A*24:22	558	KFLPFQQF	0,7981
A24	HLA-A*24:22	634	RVYSTGSNVF	0,8412
A24	HLA-A*24:22	635	VYSTGSNVF	0,9766
A24	HLA-A*24:22	659	SYECDIPIGAGI	0,6229
A24	HLA-A*24:22	706	AYSNNSIAI	0,7925
A24	HLA-A*24:22	755	QYGSFCTQL	0,7423
A24	HLA-A*24:22	788	IYKTPPIKDF	0,9284
A24	HLA-A*24:22	816	SFIEDLLF	0,7943
A24	HLA-A*24:22	880	GTITSGWTF	0,6649
A24	HLA-A*24:22	1051	SFPQSAPHGVVF	0,8172
A24	HLA-A*24:22	1066	TYVPAQEKNF	0,9704
A24	HLA-A*24:22	1094	VFVSNGTHW	0,9217
A24	HLA-A*24:22	1094	VFVSNGTHWF	0,8558
A24	HLA-A*24:22	1095	FVSNGTHWF	0,6853
A24	HLA-A*24:22	1101	HWFVTQRNF	0,884
A24	HLA-A*24:22	1137	VYDPLQPEL	0,9126
A24	HLA-A*24:22	1137	VYDPLQPELDSF	0,8166
A24	HLA-A*24:22	1147	SFKEELDKYF	0,7313
A24	HLA-A*24:22	1208	QYIKWPWYI	0,9408
A24	HLA-A*24:22	1208	QYIKWPWYIW	0,7516
A24	HLA-A*24:22	1211	KWPWYIWLGF	0,6645
A24	HLA-A*24:22	1216	IWLGFIAGL	0,7116
A24	HLA-A*24:23	36	VYYPDKVF	0,8969
A24	HLA-A*24:23	37	YYPDKVFRSSVL	0,7149
A24	HLA-A*24:23	57	PFFSNVTWF	0,8822
A24	HLA-A*24:23	77	KRFDNPVLPF	0,6134
A24	HLA-A*24:23	78	RFDNPVLPF	0,9523
A24	HLA-A*24:23	143	VYYHKNNKSW	0,8351
A24	HLA-A*24:23	144	YYHKNNKSW	0,9523
A24	HLA-A*24:23	151	SWMESEFRV	0,7048
A24	HLA-A*24:23	159	VYSSANNCTF	0,9732
A24	HLA-A*24:23	167	TFEYVSQPF	0,6889
A24	HLA-A*24:23	169	EYVSQPFLM	0,8798
A24	HLA-A*24:23	193	VFKNIDGYF	0,8984
A24	HLA-A*24:23	203	IYSKHTPINL	0,8029
A24	HLA-A*24:23	247	SYLTPGDSSSGW	0,7197
A24	HLA-A*24:23	264	AYYVGYLQPRTF	0,8966
A24	HLA-A*24:23	265	YYVGYLQPRTF	0,9506
A24	HLA-A*24:23	265	YYVGYLQPRTFL	0,6242
A24	HLA-A*24:23	267	VGYLQPRTFL	0,7184

A24	HLA-A*24:23	268	GYLQPRTF	0,7874
A24	HLA-A*24:23	268	GYLQPRTFL	0,9337
A24	HLA-A*24:23	268	GYLQPRTFLL	0,8923
A24	HLA-A*24:23	312	IYQTSNFRV	0,8841
A24	HLA-A*24:23	328	RFPNITNLCPF	0,9389
A24	HLA-A*24:23	346	RFASVYAW	0,7335
A24	HLA-A*24:23	350	VYAWNRKRI	0,6686
A24	HLA-A*24:23	368	LYNSASFSTF	0,9453
A24	HLA-A*24:23	379	CYGVSPTKL	0,6202
A24	HLA-A*24:23	395	VYADSFVI	0,6956
A24	HLA-A*24:23	448	NYNYLYRLF	0,9379
A24	HLA-A*24:23	488	CYFPLQSYGF	0,7404
A24	HLA-A*24:23	489	YFPLQSYGF	0,9778
A24	HLA-A*24:23	504	GYQPYRVVV	0,7982
A24	HLA-A*24:23	504	GYQPYRVVVL	0,7444
A24	HLA-A*24:23	504	GYQPYRVVVLSF	0,8581
A24	HLA-A*24:23	507	PYRVVVLSF	0,8533
A24	HLA-A*24:23	558	KFLPFQQF	0,8636
A24	HLA-A*24:23	632	TWRVYSTGSNVF	0,5957
A24	HLA-A*24:23	634	RVYSTGSNVF	0,8822
A24	HLA-A*24:23	635	VYSTGSNVF	0,9872
A24	HLA-A*24:23	659	SYECDIPIGAGI	0,6187
A24	HLA-A*24:23	706	AYSNNSIAI	0,8828
A24	HLA-A*24:23	755	QYGSFCTQL	0,7929
A24	HLA-A*24:23	788	IYKTPPIKDF	0,9538
A24	HLA-A*24:23	816	SFIEDLLF	0,8244
A24	HLA-A*24:23	1051	SFPQSAPHGVVF	0,8397
A24	HLA-A*24:23	1066	TYVPAQEKNF	0,97
A24	HLA-A*24:23	1094	VFVSNGTHW	0,9343
A24	HLA-A*24:23	1094	VFVSNGTHWF	0,8723
A24	HLA-A*24:23	1101	HWFVTQRNF	0,9047
A24	HLA-A*24:23	1137	VYDPLQPEL	0,9496
A24	HLA-A*24:23	1137	VYDPLQPELDSF	0,8822
A24	HLA-A*24:23	1147	SFKEELDKYF	0,6438
A24	HLA-A*24:23	1205	KYEQYIKW	0,6328
A24	HLA-A*24:23	1208	QYIKWPWYI	0,9753
A24	HLA-A*24:23	1208	QYIKWPWYIW	0,7703
A24	HLA-A*24:23	1211	KWPWYIWLGF	0,7747
A24	HLA-A*24:23	1216	IWLGFIAGL	0,7773
A24	HLA-A*24:26	37	YYPDKVFRSSVL	0,601
A24	HLA-A*24:26	57	PFFSNVTWF	0,8728
A24	HLA-A*24:26	78	RFDNPVLPF	0,8538
A24	HLA-A*24:26	143	VYYHKNNKSW	0,7378
A24	HLA-A*24:26	144	YYHKNNKSW	0,8416
A24	HLA-A*24:26	151	SWMESEFRV	0,5046
A24	HLA-A*24:26	159	VYSSANNCTF	0,9582
A24	HLA-A*24:26	167	TFEYVSQPF	0,5365
A24	HLA-A*24:26	169	EYVSQPFLM	0,8484
A24	HLA-A*24:26	203	IYSKHTPINL	0,7085
A24	HLA-A*24:26	264	AYYVGYLQPRTF	0,8037

A24	HLA-A*24:26	265	YYVGYLQPRTF	0,9005
A24	HLA-A*24:26	268	GYLQPRTFLL	0,731
A24	HLA-A*24:26	269	YLQPRTFLL	0,5359
A24	HLA-A*24:26	312	IYQTSNFRV	0,785
A24	HLA-A*24:26	328	RFPNITNLCPF	0,8789
A24	HLA-A*24:26	346	RFASVYAW	0,521
A24	HLA-A*24:26	368	LYNSASFSTF	0,914
A24	HLA-A*24:26	395	VYADSFVI	0,6394
A24	HLA-A*24:26	448	NYNYLYRLF	0,9353
A24	HLA-A*24:26	488	CYFPLQSYGF	0,5885
A24	HLA-A*24:26	489	YFPLQSYGF	0,9442
A24	HLA-A*24:26	504	GYQPYRVVVLSF	0,6707
A24	HLA-A*24:26	507	PYRVVVLSF	0,8166
A24	HLA-A*24:26	558	KFLPFQQF	0,6948
A24	HLA-A*24:26	634	RVYSTGSNVF	0,7146
A24	HLA-A*24:26	635	VYSTGSNVF	0,9696
A24	HLA-A*24:26	755	QYGSFCTQL	0,5614
A24	HLA-A*24:26	788	IYKTPPIKDF	0,9226
A24	HLA-A*24:26	816	SFIEDLLF	0,7253
A24	HLA-A*24:26	1066	TYVPAQEKNF	0,9479
A24	HLA-A*24:26	1094	VFVSNGTHW	0,8658
A24	HLA-A*24:26	1094	VFVSNGTHWF	0,8335
A24	HLA-A*24:26	1101	HWFVTQRNF	0,754
A24	HLA-A*24:26	1137	VYDPLQPELDSF	0,8174
A24	HLA-A*24:26	1208	QYIKWPWYI	0,9613
A24	HLA-A*24:26	1208	QYIKWPWYIW	0,7582
A24	HLA-A*24:26	1211	KWPWYIWLGF	0,6582
A24	HLA-A*24:27	37	YYPDKVFRSSVL	0,601
A24	HLA-A*24:27	57	PFFSNVTWF	0,8728
A24	HLA-A*24:27	78	RFDNPVLPF	0,8538
A24	HLA-A*24:27	143	VYYHKNNKSW	0,7378
A24	HLA-A*24:27	144	YYHKNNKSW	0,8416
A24	HLA-A*24:27	151	SWMESEFRV	0,5046
A24	HLA-A*24:27	159	VYSSANNCTF	0,9582
A24	HLA-A*24:27	167	TFEYVSQPF	0,5365
A24	HLA-A*24:27	169	EYVSQPFLM	0,8484
A24	HLA-A*24:27	203	IYSKHTPINL	0,7085
A24	HLA-A*24:27	264	AYYVGYLQPRTF	0,8037
A24	HLA-A*24:27	265	YYVGYLQPRTF	0,9005
A24	HLA-A*24:27	268	GYLQPRTFLL	0,731
A24	HLA-A*24:27	269	YLQPRTFLL	0,5359
A24	HLA-A*24:27	312	IYQTSNFRV	0,785
A24	HLA-A*24:27	328	RFPNITNLCPF	0,8789
A24	HLA-A*24:27	346	RFASVYAW	0,521
A24	HLA-A*24:27	368	LYNSASFSTF	0,914
A24	HLA-A*24:27	395	VYADSFVI	0,6394
A24	HLA-A*24:27	448	NYNYLYRLF	0,9353
A24	HLA-A*24:27	488	CYFPLQSYGF	0,5885
A24	HLA-A*24:27	489	YFPLQSYGF	0,9442
A24	HLA-A*24:27	504	GYQPYRVVVLSF	0,6707

A24	HLA-A*24:27	507	PYRVVVLSF	0,8166
A24	HLA-A*24:27	558	KFLPFQQF	0,6948
A24	HLA-A*24:27	634	RVYSTGSNVF	0,7146
A24	HLA-A*24:27	635	VYSTGSNVF	0,9696
A24	HLA-A*24:27	755	QYGSFCTQL	0,5614
A24	HLA-A*24:27	788	IYKTPPIKDF	0,9226
A24	HLA-A*24:27	816	SFIEDLLF	0,7253
A24	HLA-A*24:27	1066	TYVPAQEKNF	0,9479
A24	HLA-A*24:27	1094	VFVSNGTHW	0,8658
A24	HLA-A*24:27	1094	VFVSNGTHWF	0,8335
A24	HLA-A*24:27	1101	HWFVTQRNF	0,754
A24	HLA-A*24:27	1137	VYDPLQPELDSF	0,8174
A24	HLA-A*24:27	1208	QYIKWPWYI	0,9613
A24	HLA-A*24:27	1208	QYIKWPWYIW	0,7582
A24	HLA-A*24:27	1211	KWPWYIWLGF	0,6582
A24	HLA-A*24:28	151	SWMESEFRV	0,6568
A24	HLA-A*24:28	269	YLQPRTFLL	0,5702
A24	HLA-A*24:28	312	IYQTSNFRV	0,5721
A24	HLA-A*24:28	1208	QYIKWPWYI	0,7279
A24	HLA-A*24:28	1216	IWLGFIAGL	0,697
A24	HLA-A*24:29	37	YYPDKVFRSSVL	0,601
A24	HLA-A*24:29	57	PFFSNVTWF	0,8728
A24	HLA-A*24:29	78	RFDNPVLPF	0,8538
A24	HLA-A*24:29	143	VYYHKNNKSW	0,7378
A24	HLA-A*24:29	144	YYHKNNKSW	0,8416
A24	HLA-A*24:29	151	SWMESEFRV	0,5046
A24	HLA-A*24:29	159	VYSSANNCTF	0,9582
A24	HLA-A*24:29	167	TFEYVSQPF	0,5365
A24	HLA-A*24:29	169	EYVSQPFLM	0,8484
A24	HLA-A*24:29	203	IYSKHTPINL	0,7085
A24	HLA-A*24:29	264	AYYVGYLQPRTF	0,8037
A24	HLA-A*24:29	265	YYVGYLQPRTF	0,9005
A24	HLA-A*24:29	268	GYLQPRTFLL	0,731
A24	HLA-A*24:29	269	YLQPRTFLL	0,5359
A24	HLA-A*24:29	312	IYQTSNFRV	0,785
A24	HLA-A*24:29	328	RFPNITNLCPF	0,8789
A24	HLA-A*24:29	346	RFASVYAW	0,521
A24	HLA-A*24:29	368	LYNSASFSTF	0,914
A24	HLA-A*24:29	395	VYADSFVI	0,6394
A24	HLA-A*24:29	448	NYNYLYRLF	0,9353
A24	HLA-A*24:29	488	CYFPLQSYGF	0,5885
A24	HLA-A*24:29	489	YFPLQSYGF	0,9442
A24	HLA-A*24:29	504	GYQPYRVVVLSF	0,6707
A24	HLA-A*24:29	507	PYRVVVLSF	0,8166
A24	HLA-A*24:29	558	KFLPFQQF	0,6948
A24	HLA-A*24:29	634	RVYSTGSNVF	0,7146
A24	HLA-A*24:29	635	VYSTGSNVF	0,9696
A24	HLA-A*24:29	755	QYGSFCTQL	0,5614
A24	HLA-A*24:29	788	IYKTPPIKDF	0,9226
A24	HLA-A*24:29	816	SFIEDLLF	0,7253

A24	HLA-A*24:29	1066	TYVPAQEKNF	0,9479
A24	HLA-A*24:29	1094	VFVSNGTHW	0,8658
A24	HLA-A*24:29	1094	VFVSNGTHWF	0,8335
A24	HLA-A*24:29	1101	HWFVTQRNF	0,754
A24	HLA-A*24:29	1137	VYDPLQPELDSF	0,8174
A24	HLA-A*24:29	1208	QYIKWPWYI	0,9613
A24	HLA-A*24:29	1208	QYIKWPWYIW	0,7582
A24	HLA-A*24:29	1211	KWPWYIWLGF	0,6582
A24	HLA-A*24:33	36	VYYPDKVF	0,8969
A24	HLA-A*24:33	37	YYPDKVFRSSVL	0,7149
A24	HLA-A*24:33	57	PFFSNVTWF	0,8822
A24	HLA-A*24:33	77	KRFDNPVLPF	0,6134
A24	HLA-A*24:33	78	RFDNPVLPF	0,9523
A24	HLA-A*24:33	143	VYYHKNNKSW	0,8351
A24	HLA-A*24:33	144	YYHKNNKSW	0,9523
A24	HLA-A*24:33	151	SWMESEFRV	0,7048
A24	HLA-A*24:33	159	VYSSANNCTF	0,9732
A24	HLA-A*24:33	167	TFEYVSQPF	0,6889
A24	HLA-A*24:33	169	EYVSQPFLM	0,8798
A24	HLA-A*24:33	193	VFKNIDGYF	0,8984
A24	HLA-A*24:33	203	IYSKHTPINL	0,8029
A24	HLA-A*24:33	247	SYLTPGDSSSGW	0,7197
A24	HLA-A*24:33	264	AYYVGYLQPRTF	0,8966
A24	HLA-A*24:33	265	YYVGYLQPRTF	0,9506
A24	HLA-A*24:33	265	YYVGYLQPRTFL	0,6242
A24	HLA-A*24:33	267	VGYLQPRTFL	0,7184
A24	HLA-A*24:33	268	GYLQPRTF	0,7874
A24	HLA-A*24:33	268	GYLQPRTFL	0,9337
A24	HLA-A*24:33	268	GYLQPRTFLL	0,8923
A24	HLA-A*24:33	312	IYQTSNFRV	0,8841
A24	HLA-A*24:33	328	RFPNITNLCPF	0,9389
A24	HLA-A*24:33	346	RFASVYAW	0,7335
A24	HLA-A*24:33	350	VYAWNRKRI	0,6686
A24	HLA-A*24:33	368	LYNSASFSTF	0,9453
A24	HLA-A*24:33	379	CYGVSPTKL	0,6202
A24	HLA-A*24:33	395	VYADSFVI	0,6956
A24	HLA-A*24:33	448	NYNYLYRLF	0,9379
A24	HLA-A*24:33	488	CYFPLQSYGF	0,7404
A24	HLA-A*24:33	489	YFPLQSYGF	0,9778
A24	HLA-A*24:33	504	GYQPYRVVV	0,7982
A24	HLA-A*24:33	504	GYQPYRVVVL	0,7444
A24	HLA-A*24:33	504	GYQPYRVVVLSF	0,8581
A24	HLA-A*24:33	507	PYRVVVLSF	0,8533
A24	HLA-A*24:33	558	KFLPFQQF	0,8636
A24	HLA-A*24:33	632	TWRVYSTGSNVF	0,5957
A24	HLA-A*24:33	634	RVYSTGSNVF	0,8822
A24	HLA-A*24:33	635	VYSTGSNVF	0,9872
A24	HLA-A*24:33	659	SYECDIPIGAGI	0,6187
A24	HLA-A*24:33	706	AYSNNSIAI	0,8828
A24	HLA-A*24:33	755	QYGSFCTQL	0,7929

A24	HLA-A*24:33	788	IYKTPPIKDF	0,9538
A24	HLA-A*24:33	816	SFIEDLLF	0,8244
A24	HLA-A*24:33	1051	SFPQSAPHGVVF	0,8397
A24	HLA-A*24:33	1066	TYVPAQEKNF	0,97
A24	HLA-A*24:33	1094	VFVSNGTHW	0,9343
A24	HLA-A*24:33	1094	VFVSNGTHWF	0,8723
A24	HLA-A*24:33	1101	HWFVTQRNF	0,9047
A24	HLA-A*24:33	1137	VYDPLQPEL	0,9496
A24	HLA-A*24:33	1137	VYDPLQPELDSF	0,8822
A24	HLA-A*24:33	1147	SFKEELDKYF	0,6438
A24	HLA-A*24:33	1205	KYEQYIKW	0,6328
A24	HLA-A*24:33	1208	QYIKWPWYI	0,9753
A24	HLA-A*24:33	1208	QYIKWPWYIW	0,7703
A24	HLA-A*24:33	1211	KWPWYIWLGF	0,7747
A24	HLA-A*24:33	1216	IWLGFIAGL	0,7773
A24	HLA-A*24:34	37	YYPDKVFRSSVL	0,6196
A24	HLA-A*24:34	57	PFFSNVTWF	0,8335
A24	HLA-A*24:34	78	RFDNPVLPF	0,8388
A24	HLA-A*24:34	143	VYYHKNNKSW	0,6844
A24	HLA-A*24:34	144	YYHKNNKSW	0,819
A24	HLA-A*24:34	151	SWMESEFRV	0,5644
A24	HLA-A*24:34	159	VYSSANNCTF	0,9424
A24	HLA-A*24:34	167	TFEYVSQPF	0,5518
A24	HLA-A*24:34	169	EYVSQPFLM	0,8551
A24	HLA-A*24:34	193	VFKNIDGYF	0,7905
A24	HLA-A*24:34	203	IYSKHTPINL	0,7288
A24	HLA-A*24:34	264	AYYVGYLQPRTF	0,7698
A24	HLA-A*24:34	265	YYVGYLQPRTF	0,857
A24	HLA-A*24:34	268	GYLQPRTFLL	0,7636
A24	HLA-A*24:34	269	YLQPRTFLL	0,6367
A24	HLA-A*24:34	312	IYQTSNFRV	0,7925
A24	HLA-A*24:34	328	RFPNITNLCPF	0,8538
A24	HLA-A*24:34	350	VYAWNRKRI	0,5795
A24	HLA-A*24:34	368	LYNSASFSTF	0,9096
A24	HLA-A*24:34	395	VYADSFVI	0,6072
A24	HLA-A*24:34	448	NYNYLYRLF	0,9345
A24	HLA-A*24:34	488	CYFPLQSYGF	0,618
A24	HLA-A*24:34	489	YFPLQSYGF	0,9263
A24	HLA-A*24:34	504	GYQPYRVVVLSF	0,6666
A24	HLA-A*24:34	507	PYRVVVLSF	0,7841
A24	HLA-A*24:34	634	RVYSTGSNVF	0,6878
A24	HLA-A*24:34	635	VYSTGSNVF	0,9648
A24	HLA-A*24:34	706	AYSNNSIAI	0,7097
A24	HLA-A*24:34	755	QYGSFCTQL	0,6183
A24	HLA-A*24:34	788	IYKTPPIKDF	0,9047
A24	HLA-A*24:34	897	PFAMQMAYRF	0,5116
A24	HLA-A*24:34	1066	TYVPAQEKNF	0,9166
A24	HLA-A*24:34	1094	VFVSNGTHW	0,866
A24	HLA-A*24:34	1094	VFVSNGTHWF	0,8212
A24	HLA-A*24:34	1101	HWFVTQRNF	0,7483

A24	HLA-A*24:34	1137	VYDPLQPELDSF	0,7712
A24	HLA-A*24:34	1208	QYIKWPWYI	0,9658
A24	HLA-A*24:34	1208	QYIKWPWYIW	0,7348
A24	HLA-A*24:34	1211	KWPWYIWLGF	0,6265
A24	HLA-A*24:35	37	YYPDKVFRSSVL	0,601
A24	HLA-A*24:35	57	PFFSNVTWF	0,8728
A24	HLA-A*24:35	78	RFDNPVLPF	0,8538
A24	HLA-A*24:35	143	VYYHKNNKSW	0,7378
A24	HLA-A*24:35	144	YYHKNNKSW	0,8416
A24	HLA-A*24:35	151	SWMESEFRV	0,5046
A24	HLA-A*24:35	159	VYSSANNCTF	0,9582
A24	HLA-A*24:35	167	TFEYVSQPF	0,5365
A24	HLA-A*24:35	169	EYVSQPFLM	0,8484
A24	HLA-A*24:35	203	IYSKHTPINL	0,7085
A24	HLA-A*24:35	264	AYYVGYLQPRTF	0,8037
A24	HLA-A*24:35	265	YYVGYLQPRTF	0,9005
A24	HLA-A*24:35	268	GYLQPRTFLL	0,731
A24	HLA-A*24:35	269	YLQPRTFLL	0,5359
A24	HLA-A*24:35	312	IYQTSNFRV	0,785
A24	HLA-A*24:35	328	RFPNITNLCPF	0,8789
A24	HLA-A*24:35	346	RFASVYAW	0,521
A24	HLA-A*24:35	368	LYNSASFSTF	0,914
A24	HLA-A*24:35	395	VYADSFVI	0,6394
A24	HLA-A*24:35	448	NYNYLYRLF	0,9353
A24	HLA-A*24:35	488	CYFPLQSYGF	0,5885
A24	HLA-A*24:35	489	YFPLQSYGF	0,9442
A24	HLA-A*24:35	504	GYQPYRVVVLSF	0,6707
A24	HLA-A*24:35	507	PYRVVVLSF	0,8166
A24	HLA-A*24:35	558	KFLPFQQF	0,6948
A24	HLA-A*24:35	634	RVYSTGSNVF	0,7146
A24	HLA-A*24:35	635	VYSTGSNVF	0,9696
A24	HLA-A*24:35	755	QYGSFCTQL	0,5614
A24	HLA-A*24:35	788	IYKTPPIKDF	0,9226
A24	HLA-A*24:35	816	SFIEDLLF	0,7253
A24	HLA-A*24:35	1066	TYVPAQEKNF	0,9479
A24	HLA-A*24:35	1094	VFVSNGTHW	0,8658
A24	HLA-A*24:35	1094	VFVSNGTHWF	0,8335
A24	HLA-A*24:35	1101	HWFVTQRNF	0,754
A24	HLA-A*24:35	1137	VYDPLQPELDSF	0,8174
A24	HLA-A*24:35	1208	QYIKWPWYI	0,9613
A24	HLA-A*24:35	1208	QYIKWPWYIW	0,7582
A24	HLA-A*24:35	1211	KWPWYIWLGF	0,6582
A24	HLA-A*24:37	37	YYPDKVFRSSVL	0,601
A24	HLA-A*24:37	57	PFFSNVTWF	0,8728
A24	HLA-A*24:37	78	RFDNPVLPF	0,8538
A24	HLA-A*24:37	143	VYYHKNNKSW	0,7378
A24	HLA-A*24:37	144	YYHKNNKSW	0,8416
A24	HLA-A*24:37	151	SWMESEFRV	0,5046
A24	HLA-A*24:37	159	VYSSANNCTF	0,9582
A24	HLA-A*24:37	167	TFEYVSQPF	0,5365

A24	HLA-A*24:37	169	EYVSQPFLM	0,8484
A24	HLA-A*24:37	203	IYSKHTPINL	0,7085
A24	HLA-A*24:37	264	AYYVGYLQPRTF	0,8037
A24	HLA-A*24:37	265	YYVGYLQPRTF	0,9005
A24	HLA-A*24:37	268	GYLQPRTFLL	0,731
A24	HLA-A*24:37	269	YLQPRTFLL	0,5359
A24	HLA-A*24:37	312	IYQTSNFRV	0,785
A24	HLA-A*24:37	328	RFPNITNLCPF	0,8789
A24	HLA-A*24:37	346	RFASVYAW	0,521
A24	HLA-A*24:37	368	LYNSASFSTF	0,914
A24	HLA-A*24:37	395	VYADSFVI	0,6394
A24	HLA-A*24:37	448	NYNYLYRLF	0,9353
A24	HLA-A*24:37	488	CYFPLQSYGF	0,5885
A24	HLA-A*24:37	489	YFPLQSYGF	0,9442
A24	HLA-A*24:37	504	GYQPYRVVVLSF	0,6707
A24	HLA-A*24:37	507	PYRVVVLSF	0,8166
A24	HLA-A*24:37	558	KFLPFQQF	0,6948
A24	HLA-A*24:37	634	RVYSTGSNVF	0,7146
A24	HLA-A*24:37	635	VYSTGSNVF	0,9696
A24	HLA-A*24:37	755	QYGSFCTQL	0,5614
A24	HLA-A*24:37	788	IYKTPPIKDF	0,9226
A24	HLA-A*24:37	816	SFIEDLLF	0,7253
A24	HLA-A*24:37	1066	TYVPAQEKNF	0,9479
A24	HLA-A*24:37	1094	VFVSNGTHW	0,8658
A24	HLA-A*24:37	1094	VFVSNGTHWF	0,8335
A24	HLA-A*24:37	1101	HWFVTQRNF	0,754
A24	HLA-A*24:37	1137	VYDPLQPELDSF	0,8174
A24	HLA-A*24:37	1208	QYIKWPWYI	0,9613
A24	HLA-A*24:37	1208	QYIKWPWYIW	0,7582
A24	HLA-A*24:37	1211	KWPWYIWLGF	0,6582
A24	HLA-A*24:38	37	YYPDKVFRSSVL	0,601
A24	HLA-A*24:38	57	PFFSNVTWF	0,8728
A24	HLA-A*24:38	78	RFDNPVLPF	0,8538
A24	HLA-A*24:38	143	VYYHKNNKSW	0,7378
A24	HLA-A*24:38	144	YYHKNNKSW	0,8416
A24	HLA-A*24:38	151	SWMESEFRV	0,5046
A24	HLA-A*24:38	159	VYSSANNCTF	0,9582
A24	HLA-A*24:38	167	TFEYVSQPF	0,5365
A24	HLA-A*24:38	169	EYVSQPFLM	0,8484
A24	HLA-A*24:38	203	IYSKHTPINL	0,7085
A24	HLA-A*24:38	264	AYYVGYLQPRTF	0,8037
A24	HLA-A*24:38	265	YYVGYLQPRTF	0,9005
A24	HLA-A*24:38	268	GYLQPRTFLL	0,731
A24	HLA-A*24:38	269	YLQPRTFLL	0,5359
A24	HLA-A*24:38	312	IYQTSNFRV	0,785
A24	HLA-A*24:38	328	RFPNITNLCPF	0,8789
A24	HLA-A*24:38	346	RFASVYAW	0,521
A24	HLA-A*24:38	368	LYNSASFSTF	0,914
A24	HLA-A*24:38	395	VYADSFVI	0,6394
A24	HLA-A*24:38	448	NYNYLYRLF	0,9353

A24	HLA-A*24:38	488	CYFPLQSYGF	0,5885
A24	HLA-A*24:38	489	YFPLQSYGF	0,9442
A24	HLA-A*24:38	504	GYQPYRVVVLSF	0,6707
A24	HLA-A*24:38	507	PYRVVVLSF	0,8166
A24	HLA-A*24:38	558	KFLPFQQF	0,6948
A24	HLA-A*24:38	634	RVYSTGSNVF	0,7146
A24	HLA-A*24:38	635	VYSTGSNVF	0,9696
A24	HLA-A*24:38	755	QYGSFCTQL	0,5614
A24	HLA-A*24:38	788	IYKTPPIKDF	0,9226
A24	HLA-A*24:38	816	SFIEDLLF	0,7253
A24	HLA-A*24:38	1066	TYVPAQEKNF	0,9479
A24	HLA-A*24:38	1094	VFVSNGTHW	0,8658
A24	HLA-A*24:38	1094	VFVSNGTHWF	0,8335
A24	HLA-A*24:38	1101	HWFVTQRNF	0,754
A24	HLA-A*24:38	1137	VYDPLQPELDSF	0,8174
A24	HLA-A*24:38	1208	QYIKWPWYI	0,9613
A24	HLA-A*24:38	1208	QYIKWPWYIW	0,7582
A24	HLA-A*24:38	1211	KWPWYIWLGF	0,6582
A24	HLA-A*24:39	37	YYPDKVFRSSVL	0,601
A24	HLA-A*24:39	57	PFFSNVTWF	0,8728
A24	HLA-A*24:39	78	RFDNPVLPF	0,8538
A24	HLA-A*24:39	143	VYYHKNNKSW	0,7378
A24	HLA-A*24:39	144	YYHKNNKSW	0,8416
A24	HLA-A*24:39	151	SWMESEFRV	0,5046
A24	HLA-A*24:39	159	VYSSANNCTF	0,9582
A24	HLA-A*24:39	167	TFEYVSQPF	0,5365
A24	HLA-A*24:39	169	EYVSQPFLM	0,8484
A24	HLA-A*24:39	203	IYSKHTPINL	0,7085
A24	HLA-A*24:39	264	AYYVGYLQPRTF	0,8037
A24	HLA-A*24:39	265	YYVGYLQPRTF	0,9005
A24	HLA-A*24:39	268	GYLQPRTFLL	0,731
A24	HLA-A*24:39	269	YLQPRTFLL	0,5359
A24	HLA-A*24:39	312	IYQTSNFRV	0,785
A24	HLA-A*24:39	328	RFPNITNLCPF	0,8789
A24	HLA-A*24:39	346	RFASVYAW	0,521
A24	HLA-A*24:39	368	LYNSASFSTF	0,914
A24	HLA-A*24:39	395	VYADSFVI	0,6394
A24	HLA-A*24:39	448	NYNYLYRLF	0,9353
A24	HLA-A*24:39	488	CYFPLQSYGF	0,5885
A24	HLA-A*24:39	489	YFPLQSYGF	0,9442
A24	HLA-A*24:39	504	GYQPYRVVVLSF	0,6707
A24	HLA-A*24:39	507	PYRVVVLSF	0,8166
A24	HLA-A*24:39	558	KFLPFQQF	0,6948
A24	HLA-A*24:39	634	RVYSTGSNVF	0,7146
A24	HLA-A*24:39	635	VYSTGSNVF	0,9696
A24	HLA-A*24:39	755	QYGSFCTQL	0,5614
A24	HLA-A*24:39	788	IYKTPPIKDF	0,9226
A24	HLA-A*24:39	816	SFIEDLLF	0,7253
A24	HLA-A*24:39	1066	TYVPAQEKNF	0,9479
A24	HLA-A*24:39	1094	VFVSNGTHW	0,8658

A24	HLA-A*24:39	1094	VFVSNGTHWF	0,8335
A24	HLA-A*24:39	1101	HWFVTQRNF	0,754
A24	HLA-A*24:39	1137	VYDPLQPELDSF	0,8174
A24	HLA-A*24:39	1208	QYIKWPWYI	0,9613
A24	HLA-A*24:39	1208	QYIKWPWYIW	0,7582
A24	HLA-A*24:39	1211	KWPWYIWLGF	0,6582
A24	HLA-A*24:43	37	YYPDKVFRSSVL	0,601
A24	HLA-A*24:43	57	PFFSNVTWF	0,8728
A24	HLA-A*24:43	78	RFDNPVLPF	0,8538
A24	HLA-A*24:43	143	VYYHKNNKSW	0,7378
A24	HLA-A*24:43	144	YYHKNNKSW	0,8416
A24	HLA-A*24:43	151	SWMESEFRV	0,5046
A24	HLA-A*24:43	159	VYSSANNCTF	0,9582
A24	HLA-A*24:43	167	TFEYVSQPF	0,5365
A24	HLA-A*24:43	169	EYVSQPFLM	0,8484
A24	HLA-A*24:43	203	IYSKHTPINL	0,7085
A24	HLA-A*24:43	264	AYYVGYLQPRTF	0,8037
A24	HLA-A*24:43	265	YYVGYLQPRTF	0,9005
A24	HLA-A*24:43	268	GYLQPRTFLL	0,731
A24	HLA-A*24:43	269	YLQPRTFLL	0,5359
A24	HLA-A*24:43	312	IYQTSNFRV	0,785
A24	HLA-A*24:43	328	RFPNITNLCPF	0,8789
A24	HLA-A*24:43	346	RFASVYAW	0,521
A24	HLA-A*24:43	368	LYNSASFSTF	0,914
A24	HLA-A*24:43	395	VYADSFVI	0,6394
A24	HLA-A*24:43	448	NYNYLYRLF	0,9353
A24	HLA-A*24:43	488	CYFPLQSYGF	0,5885
A24	HLA-A*24:43	489	YFPLQSYGF	0,9442
A24	HLA-A*24:43	504	GYQPYRVVVLSF	0,6707
A24	HLA-A*24:43	507	PYRVVVLSF	0,8166
A24	HLA-A*24:43	558	KFLPFQQF	0,6948
A24	HLA-A*24:43	634	RVYSTGSNVF	0,7146
A24	HLA-A*24:43	635	VYSTGSNVF	0,9696
A24	HLA-A*24:43	755	QYGSFCTQL	0,5614
A24	HLA-A*24:43	788	IYKTPPIKDF	0,9226
A24	HLA-A*24:43	816	SFIEDLLF	0,7253
A24	HLA-A*24:43	1066	TYVPAQEKNF	0,9479
A24	HLA-A*24:43	1094	VFVSNGTHW	0,8658
A24	HLA-A*24:43	1094	VFVSNGTHWF	0,8335
A24	HLA-A*24:43	1101	HWFVTQRNF	0,754
A24	HLA-A*24:43	1137	VYDPLQPELDSF	0,8174
A24	HLA-A*24:43	1208	QYIKWPWYI	0,9613
A24	HLA-A*24:43	1208	QYIKWPWYIW	0,7582
A24	HLA-A*24:43	1211	KWPWYIWLGF	0,6582
A24	HLA-A*24:46	37	YYPDKVFRSSVL	0,5855
A24	HLA-A*24:46	57	PFFSNVTWF	0,8459
A24	HLA-A*24:46	78	RFDNPVLPF	0,8229
A24	HLA-A*24:46	159	VYSSANNCTF	0,9103
A24	HLA-A*24:46	169	EYVSQPFLM	0,851
A24	HLA-A*24:46	264	AYYVGYLQPRTF	0,6417

A24	HLA-A*24:46	265	YYVGYLQPRTF	0,8194
A24	HLA-A*24:46	268	GYLQPRTFLL	0,6533
A24	HLA-A*24:46	269	YLQPRTFLL	0,5285
A24	HLA-A*24:46	312	IYQTSNFRV	0,6584
A24	HLA-A*24:46	328	RFPNITNLCPF	0,7845
A24	HLA-A*24:46	368	LYNSASFSTF	0,8513
A24	HLA-A*24:46	395	VYADSFVI	0,5561
A24	HLA-A*24:46	448	NYNYLYRLF	0,871
A24	HLA-A*24:46	488	CYFPLQSYGF	0,5138
A24	HLA-A*24:46	489	YFPLQSYGF	0,9325
A24	HLA-A*24:46	504	GYQPYRVVVLSF	0,5468
A24	HLA-A*24:46	635	VYSTGSNVF	0,9278
A24	HLA-A*24:46	755	QYGSFCTQL	0,5152
A24	HLA-A*24:46	816	SFIEDLLF	0,7078
A24	HLA-A*24:46	1066	TYVPAQEKNF	0,9054
A24	HLA-A*24:46	1094	VFVSNGTHWF	0,756
A24	HLA-A*24:46	1137	VYDPLQPELDSF	0,7602
A24	HLA-A*24:46	1208	QYIKWPWYI	0,9343
A24	HLA-A*24:46	1208	QYIKWPWYIW	0,6481
A24	HLA-A*24:46	1211	KWPWYIWLGF	0,5239
A24	HLA-A*24:47	37	YYPDKVFRSSVL	0,601
A24	HLA-A*24:47	57	PFFSNVTWF	0,8728
A24	HLA-A*24:47	78	RFDNPVLPF	0,8538
A24	HLA-A*24:47	143	VYYHKNNKSW	0,7378
A24	HLA-A*24:47	144	YYHKNNKSW	0,8416
A24	HLA-A*24:47	151	SWMESEFRV	0,5046
A24	HLA-A*24:47	159	VYSSANNCTF	0,9582
A24	HLA-A*24:47	167	TFEYVSQPF	0,5365
A24	HLA-A*24:47	169	EYVSQPFLM	0,8484
A24	HLA-A*24:47	203	IYSKHTPINL	0,7085
A24	HLA-A*24:47	264	AYYVGYLQPRTF	0,8037
A24	HLA-A*24:47	265	YYVGYLQPRTF	0,9005
A24	HLA-A*24:47	268	GYLQPRTFLL	0,731
A24	HLA-A*24:47	269	YLQPRTFLL	0,5359
A24	HLA-A*24:47	312	IYQTSNFRV	0,785
A24	HLA-A*24:47	328	RFPNITNLCPF	0,8789
A24	HLA-A*24:47	346	RFASVYAW	0,521
A24	HLA-A*24:47	368	LYNSASFSTF	0,914
A24	HLA-A*24:47	395	VYADSFVI	0,6394
A24	HLA-A*24:47	448	NYNYLYRLF	0,9353
A24	HLA-A*24:47	488	CYFPLQSYGF	0,5885
A24	HLA-A*24:47	489	YFPLQSYGF	0,9442
A24	HLA-A*24:47	504	GYQPYRVVVLSF	0,6707
A24	HLA-A*24:47	507	PYRVVVLSF	0,8166
A24	HLA-A*24:47	558	KFLPFQQF	0,6948
A24	HLA-A*24:47	634	RVYSTGSNVF	0,7146
A24	HLA-A*24:47	635	VYSTGSNVF	0,9696
A24	HLA-A*24:47	755	QYGSFCTQL	0,5614
A24	HLA-A*24:47	788	IYKTPPIKDF	0,9226
A24	HLA-A*24:47	816	SFIEDLLF	0,7253

A24	HLA-A*24:47	1066	TYVPAQEKNF	0,9479
A24	HLA-A*24:47	1094	VFVSNGTHW	0,8658
A24	HLA-A*24:47	1094	VFVSNGTHWF	0,8335
A24	HLA-A*24:47	1101	HWFVTQRNF	0,754
A24	HLA-A*24:47	1137	VYDPLQPELDSF	0,8174
A24	HLA-A*24:47	1208	QYIKWPWYI	0,9613
A24	HLA-A*24:47	1208	QYIKWPWYIW	0,7582
A24	HLA-A*24:47	1211	KWPWYIWLGF	0,6582
A24	HLA-A*24:49	37	YYPDKVFRSSVL	0,601
A24	HLA-A*24:49	57	PFFSNVTWF	0,8728
A24	HLA-A*24:49	78	RFDNPVLPF	0,8538
A24	HLA-A*24:49	143	VYYHKNNKSW	0,7378
A24	HLA-A*24:49	144	YYHKNNKSW	0,8416
A24	HLA-A*24:49	151	SWMESEFRV	0,5046
A24	HLA-A*24:49	159	VYSSANNCTF	0,9582
A24	HLA-A*24:49	167	TFEYVSQPF	0,5365
A24	HLA-A*24:49	169	EYVSQPFLM	0,8484
A24	HLA-A*24:49	203	IYSKHTPINL	0,7085
A24	HLA-A*24:49	264	AYYVGYLQPRTF	0,8037
A24	HLA-A*24:49	265	YYVGYLQPRTF	0,9005
A24	HLA-A*24:49	268	GYLQPRTFLL	0,731
A24	HLA-A*24:49	269	YLQPRTFLL	0,5359
A24	HLA-A*24:49	312	IYQTSNFRV	0,785
A24	HLA-A*24:49	328	RFPNITNLCPF	0,8789
A24	HLA-A*24:49	346	RFASVYAW	0,521
A24	HLA-A*24:49	368	LYNSASFSTF	0,914
A24	HLA-A*24:49	395	VYADSFVI	0,6394
A24	HLA-A*24:49	448	NYNYLYRLF	0,9353
A24	HLA-A*24:49	488	CYFPLQSYGF	0,5885
A24	HLA-A*24:49	489	YFPLQSYGF	0,9442
A24	HLA-A*24:49	504	GYQPYRVVVLSF	0,6707
A24	HLA-A*24:49	507	PYRVVVLSF	0,8166
A24	HLA-A*24:49	558	KFLPFQQF	0,6948
A24	HLA-A*24:49	634	RVYSTGSNVF	0,7146
A24	HLA-A*24:49	635	VYSTGSNVF	0,9696
A24	HLA-A*24:49	755	QYGSFCTQL	0,5614
A24	HLA-A*24:49	788	IYKTPPIKDF	0,9226
A24	HLA-A*24:49	816	SFIEDLLF	0,7253
A24	HLA-A*24:49	1066	TYVPAQEKNF	0,9479
A24	HLA-A*24:49	1094	VFVSNGTHW	0,8658
A24	HLA-A*24:49	1094	VFVSNGTHWF	0,8335
A24	HLA-A*24:49	1101	HWFVTQRNF	0,754
A24	HLA-A*24:49	1137	VYDPLQPELDSF	0,8174
A24	HLA-A*24:49	1208	QYIKWPWYI	0,9613
A24	HLA-A*24:49	1208	QYIKWPWYIW	0,7582
A24	HLA-A*24:49	1211	KWPWYIWLGF	0,6582

Note: The epitopes conserved among Khosta-2 and SARS-CoV-2 are indicated in red
