# Supplementary material for: Conservation of HLA Spike Protein Epitopes Supports T Cell Cross-Protection in SARS-CoV-2 Vaccinated Individuals against the Potentially Zoonotic Coronavirus Khosta-2
Source: Int J Mol Sci. 2024 May 31;25(11):6087. doi: 10.3390/ijms25116087 (PMC11172828; doi:10.3390/ijms25116087)
Supplement: Supplementary file 1 [file ijms-25-06087-s001.zip › Supplemental Table 2.pdf]

Supplemental Table 2

Supertype	Allele	Position	Peptide	Score (EL)
B07	HLA-B*07:02	24	LPPAYTNSF	0,5263
B07	HLA-B*07:02	38	YPDKVFRSSV	0,5308
B07	HLA-B*07:02	208	TPINLVRDL	0,7332
B07	HLA-B*07:02	216	LPQGFSAL	0,6854
B07	HLA-B*07:02	462	KPFERDISTEI	0,6344
B07	HLA-B*07:02	506	QPYRVVVL	0,6389
B07	HLA-B*07:02	526	GPKKSTNLV	0,6037
B07	HLA-B*07:02	620	VPVAIHADQL	0,5101
B07	HLA-B*07:02	680	SPRRARSVA	0,7296
B07	HLA-B*07:02	714	IPTNFTISV	0,6596
B07	HLA-B*07:02	1056	APHGVVFL	0,6399
B07	HLA-B*07:02	1261	SEPVLKGVKL	0,6496
B07	HLA-B*07:03	24	LPPAYTNSF	0,6635
B07	HLA-B*07:03	38	YPDKVFRSSV	0,6468
B07	HLA-B*07:03	38	YPDKVFRSSVL	0,5441
B07	HLA-B*07:03	208	TPINLVRDL	0,7801
B07	HLA-B*07:03	216	LPQGFSAL	0,8331
B07	HLA-B*07:03	462	KPFERDISTEI	0,5705
B07	HLA-B*07:03	506	QPYRVVVL	0,8112
B07	HLA-B*07:03	526	GPKKSTNL	0,5568
B07	HLA-B*07:03	526	GPKKSTNLV	0,6514
B07	HLA-B*07:03	588	TPCSFGGVSV	0,525
B07	HLA-B*07:03	620	VPVAIHADQL	0,5699
B07	HLA-B*07:03	680	SPRRARSVA	0,7613
B07	HLA-B*07:03	714	IPTNFTISV	0,7313
B07	HLA-B*07:03	869	MIAQYTSAL	0,584
B07	HLA-B*07:03	1052	FPQSAPHGV	0,5776
B07	HLA-B*07:03	1052	FPQSAPHGVVF	0,6134
B07	HLA-B*07:03	1056	APHGVVFL	0,7644
B07	HLA-B*07:03	1261	SEPVLKGVKL	0,7262
B07	HLA-B*07:04	208	TPINLVRDL	0,6264
B07	HLA-B*07:04	462	KPFERDISTEI	0,5191
B07	HLA-B*07:04	506	QPYRVVVL	0,6271
B07	HLA-B*07:04	526	GPKKSTNLV	0,5173
B07	HLA-B*07:04	680	SPRRARSVA	0,6865
B07	HLA-B*07:04	714	IPTNFTISV	0,5207
B07	HLA-B*07:04	1261	SEPVLKGVKL	0,5427
B07	HLA-B*07:05	24	LPPAYTNSF	0,5378
B07	HLA-B*07:05	38	YPDKVFRSSV	0,6462
B07	HLA-B*07:05	208	TPINLVRDL	0,7572
B07	HLA-B*07:05	216	LPQGFSAL	0,7578
B07	HLA-B*07:05	462	KPFERDISTEI	0,6075
B07	HLA-B*07:05	506	QPYRVVVL	0,7004
B07	HLA-B*07:05	526	GPKKSTNLV	0,6522
B07	HLA-B*07:05	588	TPCSFGGVSV	0,5218
B07	HLA-B*07:05	620	VPVAIHADQL	0,5518
B07	HLA-B*07:05	680	SPRRARSVA	0,7396
B07	HLA-B*07:05	714	IPTNFTISV	0,75

B07	HLA-B*07:05	1056	APHGVVFL	0,6769
B07	HLA-B*07:05	1261	SEPVLKGVKL	0,6685
B07	HLA-B*07:06	24	LPPAYTNSF	0,5378
B07	HLA-B*07:06	38	YPDKVFRSSV	0,6462
B07	HLA-B*07:06	208	TPINLVRDL	0,7572
B07	HLA-B*07:06	216	LPQGFSAL	0,7578
B07	HLA-B*07:06	462	KPFERDISTEI	0,6075
B07	HLA-B*07:06	506	QPYRVVVL	0,7004
B07	HLA-B*07:06	526	GPKKSTNLV	0,6522
B07	HLA-B*07:06	588	TPCSFGGVSV	0,5218
B07	HLA-B*07:06	620	VPVAIHADQL	0,5518
B07	HLA-B*07:06	680	SPRRARSVA	0,7396
B07	HLA-B*07:06	714	IPTNFTISV	0,75
B07	HLA-B*07:06	1056	APHGVVFL	0,6769
B07	HLA-B*07:06	1261	SEPVLKGVKL	0,6685
B07	HLA-B*07:15	38	YPDKVFRSSV	0,6004
B07	HLA-B*07:15	208	TPINLVRDL	0,7101
B07	HLA-B*07:15	216	LPQGFSAL	0,6649
B07	HLA-B*07:15	506	QPYRVVVL	0,6118
B07	HLA-B*07:15	526	GPKKSTNLV	0,5338
B07	HLA-B*07:15	680	SPRRARSVA	0,6589
B07	HLA-B*07:15	714	IPTNFTISV	0,7194
B07	HLA-B*07:15	1052	FPQSAPHGV	0,5075
B07	HLA-B*07:15	1056	APHGVVFL	0,6254
B07	HLA-B*07:15	1261	SEPVLKGVKL	0,5393
B07	HLA-B*07:19	24	LPPAYTNSF	0,5493
B07	HLA-B*07:19	208	TPINLVRDL	0,7209
B07	HLA-B*07:19	216	LPQGFSAL	0,5972
B07	HLA-B*07:19	462	KPFERDISTEI	0,5462
B07	HLA-B*07:19	506	QPYRVVVL	0,744
B07	HLA-B*07:19	526	GPKKSTNLV	0,6123
B07	HLA-B*07:19	680	SPRRARSV	0,5347
B07	HLA-B*07:19	680	SPRRARSVA	0,7444
B07	HLA-B*07:19	714	IPTNFTISV	0,6042
B07	HLA-B*07:19	1056	APHGVVFL	0,5804
B07	HLA-B*07:19	1261	SEPVLKGVKL	0,6196
B07	HLA-B*07:20	24	LPPAYTNSF	0,5771
B07	HLA-B*07:20	208	TPINLVRDL	0,6858
B07	HLA-B*07:20	216	LPQGFSAL	0,5023
B07	HLA-B*07:21	24	LPPAYTNSF	0,5263
B07	HLA-B*07:21	38	YPDKVFRSSV	0,5308
B07	HLA-B*07:21	208	TPINLVRDL	0,7332
B07	HLA-B*07:21	216	LPQGFSAL	0,6854
B07	HLA-B*07:21	462	KPFERDISTEI	0,6344
B07	HLA-B*07:21	506	QPYRVVVL	0,6389
B07	HLA-B*07:21	526	GPKKSTNLV	0,6037
B07	HLA-B*07:21	620	VPVAIHADQL	0,5101
B07	HLA-B*07:21	680	SPRRARSVA	0,7296
B07	HLA-B*07:21	714	IPTNFTISV	0,6596
B07	HLA-B*07:21	1056	APHGVVFL	0,6399

B07	HLA-B*07:21	1261	SEPVLKGVKL	0,6496
B07	HLA-B*07:22	24	LPPAYTNSF	0,5263
B07	HLA-B*07:22	38	YPDKVFRSSV	0,5308
B07	HLA-B*07:22	208	TPINLVRDL	0,7332
B07	HLA-B*07:22	216	LPQGFSAL	0,6854
B07	HLA-B*07:22	462	KPFERDISTEI	0,6344
B07	HLA-B*07:22	506	QPYRVVVL	0,6389
B07	HLA-B*07:22	526	GPKKSTNLV	0,6037
B07	HLA-B*07:22	620	VPVAIHADQL	0,5101
B07	HLA-B*07:22	680	SPRRARSVA	0,7296
B07	HLA-B*07:22	714	IPTNFTISV	0,6596
B07	HLA-B*07:22	1056	APHGVVFL	0,6399
B07	HLA-B*07:22	1261	SEPVLKGVKL	0,6496
B07	HLA-B*07:24	24	LPPAYTNSF	0,6317
B07	HLA-B*07:24	38	YPDKVFRSSV	0,5009
B07	HLA-B*07:24	84	LPFNDGVYF	0,6784
B07	HLA-B*07:24	208	TPINLVRDL	0,787
B07	HLA-B*07:24	216	LPQGFSAL	0,6678
B07	HLA-B*07:24	506	QPYRVVVL	0,6042
B07	HLA-B*07:24	526	GPKKSTNLV	0,5315
B07	HLA-B*07:24	680	SPRRARSVA	0,6404
B07	HLA-B*07:24	714	IPTNFTISV	0,6579
B07	HLA-B*07:24	1052	FPQSAPHGVVF	0,5403
B07	HLA-B*07:24	1261	SEPVLKGVKL	0,6013
B07	HLA-B*07:25	24	LPPAYTNSF	0,6744
B07	HLA-B*07:25	84	LPFNDGVYF	0,6682
B07	HLA-B*07:25	208	TPINLVRDL	0,7696
B07	HLA-B*07:25	216	LPQGFSAL	0,5739
B07	HLA-B*07:25	506	QPYRVVVL	0,6556
B07	HLA-B*07:25	620	VPVAIHADQL	0,5421
B07	HLA-B*07:25	714	IPTNFTISV	0,6917
B07	HLA-B*07:25	1052	FPQSAPHGV	0,5272
B07	HLA-B*07:25	1052	FPQSAPHGVVF	0,524
B07	HLA-B*07:25	1056	APHGVVFL	0,6273
B07	HLA-B*07:25	1261	SEPVLKGVKL	0,6103
B07	HLA-B*07:25	1262	EPVLKGVKL	0,6809
B07	HLA-B*07:26	208	TPINLVRDL	0,6472
B07	HLA-B*07:26	506	QPYRVVVL	0,5503
B07	HLA-B*07:26	680	SPRRARSVA	0,542
B07	HLA-B*07:26	714	IPTNFTISV	0,5255
B07	HLA-B*07:30	24	LPPAYTNSF	0,5263
B07	HLA-B*07:30	38	YPDKVFRSSV	0,5308
B07	HLA-B*07:30	208	TPINLVRDL	0,7332
B07	HLA-B*07:30	216	LPQGFSAL	0,6854
B07	HLA-B*07:30	462	KPFERDISTEI	0,6344
B07	HLA-B*07:30	506	QPYRVVVL	0,6389
B07	HLA-B*07:30	526	GPKKSTNLV	0,6037
B07	HLA-B*07:30	620	VPVAIHADQL	0,5101
B07	HLA-B*07:30	680	SPRRARSVA	0,7296
B07	HLA-B*07:30	714	IPTNFTISV	0,6596

B07	HLA-B*07:30	1056	APHGVVFL	0,6399
B07	HLA-B*07:30	1261	SEPVLKGVKL	0,6496
B07	HLA-B*07:31	24	LPPAYTNSF	0,6652
B07	HLA-B*07:31	38	YPDKVFRSSV	0,6505
B07	HLA-B*07:31	84	LPFNDGVYF	0,7128
B07	HLA-B*07:31	208	TPINLVRDL	0,8331
B07	HLA-B*07:31	216	LPQGFSAL	0,8036
B07	HLA-B*07:31	229	LPIGINITRF	0,5034
B07	HLA-B*07:31	462	KPFERDISTEI	0,6827
B07	HLA-B*07:31	506	QPYRVVVL	0,7867
B07	HLA-B*07:31	526	GPKKSTNLV	0,7144
B07	HLA-B*07:31	620	VPVAIHADQL	0,6023
B07	HLA-B*07:31	680	SPRRARSVA	0,8018
B07	HLA-B*07:31	714	IPTNFTISV	0,7547
B07	HLA-B*07:31	1052	FPQSAPHGV	0,5262
B07	HLA-B*07:31	1052	FPQSAPHGVVF	0,5664
B07	HLA-B*07:31	1056	APHGVVFL	0,7394
B07	HLA-B*07:31	1261	SEPVLKGVKL	0,7452
B07	HLA-B*07:31	1262	EPVLKGVKL	0,7089
B07	HLA-B*07:33	24	LPPAYTNSF	0,5263
B07	HLA-B*07:33	38	YPDKVFRSSV	0,5308
B07	HLA-B*07:33	208	TPINLVRDL	0,7332
B07	HLA-B*07:33	216	LPQGFSAL	0,6854
B07	HLA-B*07:33	462	KPFERDISTEI	0,6344
B07	HLA-B*07:33	506	QPYRVVVL	0,6389
B07	HLA-B*07:33	526	GPKKSTNLV	0,6037
B07	HLA-B*07:33	620	VPVAIHADQL	0,5101
B07	HLA-B*07:33	680	SPRRARSVA	0,7296
B07	HLA-B*07:33	714	IPTNFTISV	0,6596
B07	HLA-B*07:33	1056	APHGVVFL	0,6399
B07	HLA-B*07:33	1261	SEPVLKGVKL	0,6496
B07	HLA-B*07:34	24	LPPAYTNSF	0,5137
B07	HLA-B*07:34	84	LPFNDGVYF	0,6562
B07	HLA-B*07:34	208	TPINLVRDL	0,7702
B07	HLA-B*07:34	216	LPQGFSAL	0,6635
B07	HLA-B*07:34	506	QPYRVVVL	0,7653
B07	HLA-B*07:34	526	GPKKSTNLV	0,5309
B07	HLA-B*07:34	680	SPRRARSVA	0,6471
B07	HLA-B*07:34	714	IPTNFTISV	0,7183
B07	HLA-B*07:34	1056	APHGVVFL	0,6103
B07	HLA-B*07:34	1261	SEPVLKGVKL	0,5539
B07	HLA-B*07:35	24	LPPAYTNSF	0,5263
B07	HLA-B*07:35	38	YPDKVFRSSV	0,5308
B07	HLA-B*07:35	208	TPINLVRDL	0,7332
B07	HLA-B*07:35	216	LPQGFSAL	0,6854
B07	HLA-B*07:35	462	KPFERDISTEI	0,6344
B07	HLA-B*07:35	506	QPYRVVVL	0,6389
B07	HLA-B*07:35	526	GPKKSTNLV	0,6037
B07	HLA-B*07:35	620	VPVAIHADQL	0,5101
B07	HLA-B*07:35	680	SPRRARSVA	0,7296

B07	HLA-B*07:35	714	IPTNFTISV	0,6596
B07	HLA-B*07:35	1056	APHGVVFL	0,6399
B07	HLA-B*07:35	1261	SEPVLKGVKL	0,6496
B07	HLA-B*07:39	24	LPPAYTNSF	0,5263
B07	HLA-B*07:39	38	YPDKVFRSSV	0,5308
B07	HLA-B*07:39	208	TPINLVRDL	0,7332
B07	HLA-B*07:39	216	LPQGFSAL	0,6854
B07	HLA-B*07:39	462	KPFERDISTEI	0,6344
B07	HLA-B*07:39	506	QPYRVVVL	0,6389
B07	HLA-B*07:39	526	GPKKSTNLV	0,6037
B07	HLA-B*07:39	620	VPVAIHADQL	0,5101
B07	HLA-B*07:39	680	SPRRARSVA	0,7296
B07	HLA-B*07:39	714	IPTNFTISV	0,6596
B07	HLA-B*07:39	1056	APHGVVFL	0,6399
B07	HLA-B*07:39	1261	SEPVLKGVKL	0,6496
B07	HLA-B*07:40	24	LPPAYTNSF	0,5378
B07	HLA-B*07:40	38	YPDKVFRSSV	0,6462
B07	HLA-B*07:40	208	TPINLVRDL	0,7572
B07	HLA-B*07:40	216	LPQGFSAL	0,7578
B07	HLA-B*07:40	462	KPFERDISTEI	0,6075
B07	HLA-B*07:40	506	QPYRVVVL	0,7004
B07	HLA-B*07:40	526	GPKKSTNLV	0,6522
B07	HLA-B*07:40	588	TPCSFGGVSV	0,5218
B07	HLA-B*07:40	620	VPVAIHADQL	0,5518
B07	HLA-B*07:40	680	SPRRARSVA	0,7396
B07	HLA-B*07:40	714	IPTNFTISV	0,75
B07	HLA-B*07:40	1056	APHGVVFL	0,6769
B07	HLA-B*07:40	1261	SEPVLKGVKL	0,6685
B07	HLA-B*07:41	24	LPPAYTNSF	0,5263
B07	HLA-B*07:41	38	YPDKVFRSSV	0,5308
B07	HLA-B*07:41	208	TPINLVRDL	0,7332
B07	HLA-B*07:41	216	LPQGFSAL	0,6854
B07	HLA-B*07:41	462	KPFERDISTEI	0,6344
B07	HLA-B*07:41	506	QPYRVVVL	0,6389
B07	HLA-B*07:41	526	GPKKSTNLV	0,6037
B07	HLA-B*07:41	620	VPVAIHADQL	0,5101
B07	HLA-B*07:41	680	SPRRARSVA	0,7296
B07	HLA-B*07:41	714	IPTNFTISV	0,6596
B07	HLA-B*07:41	1056	APHGVVFL	0,6399
B07	HLA-B*07:41	1261	SEPVLKGVKL	0,6496
B07	HLA-B*07:42	24	LPPAYTNSF	0,5263
B07	HLA-B*07:42	38	YPDKVFRSSV	0,5308
B07	HLA-B*07:42	208	TPINLVRDL	0,7332
B07	HLA-B*07:42	216	LPQGFSAL	0,6854
B07	HLA-B*07:42	462	KPFERDISTEI	0,6344
B07	HLA-B*07:42	506	QPYRVVVL	0,6389
B07	HLA-B*07:42	526	GPKKSTNLV	0,6037
B07	HLA-B*07:42	620	VPVAIHADQL	0,5101
B07	HLA-B*07:42	680	SPRRARSVA	0,7296
B07	HLA-B*07:42	714	IPTNFTISV	0,6596

B07	HLA-B*07:42	1056	APHGVVFL	0,6399
B07	HLA-B*07:42	1261	SEPVLKGVKL	0,6496
B07	HLA-B*07:43	84	LPFNDGVYF	0,6105
B07	HLA-B*07:43	208	TPINLVRDL	0,7315
B07	HLA-B*07:43	216	LPQGFSAL	0,5483
B07	HLA-B*07:43	506	QPYRVVVL	0,6776
B07	HLA-B*07:43	680	SPRRARSVA	0,6226
B07	HLA-B*07:43	714	IPTNFTISV	0,6031
B07	HLA-B*15:08	84	LPFNDGVYF	0,6442
B07	HLA-B*15:08	192	FVFKNIDGY	0,5399
B07	HLA-B*15:08	687	VASQSIIAY	0,7217
B07	HLA-B*15:08	699	LGAENSVAY	0,5058
B07	HLA-B*15:08	896	IPFAMQMAY	0,7301
B07	HLA-B*15:08	1054	QSAPHGVVF	0,5652
B07	HLA-B*35:01	24	LPPAYTNSF	0,8786
B07	HLA-B*35:01	30	NSFTRGVYY	0,5335
B07	HLA-B*35:01	56	LPFFSNVTW	0,8069
B07	HLA-B*35:01	83	VLPFNDGVYF	0,7228
B07	HLA-B*35:01	84	LPFNDGVY	0,824
B07	HLA-B*35:01	84	LPFNDGVYF	0,9877
B07	HLA-B*35:01	162	SANNCTFEY	0,6698
B07	HLA-B*35:01	192	FVFKNIDGY	0,7837
B07	HLA-B*35:01	229	LPIGINITRF	0,8394
B07	HLA-B*35:01	258	WTAGAAAYY	0,5393
B07	HLA-B*35:01	271	QPRTFLLKY	0,6565
B07	HLA-B*35:01	321	QPTESIVRF	0,9476
B07	HLA-B*35:01	343	NATRFASVY	0,7573
B07	HLA-B*35:01	361	CVADYSVLY	0,5167
B07	HLA-B*35:01	478	TPCNGVEGF	0,5961
B07	HLA-B*35:01	604	TSNQVAVLY	0,5492
B07	HLA-B*35:01	625	HADQLTPTW	0,7176
B07	HLA-B*35:01	664	IPIGAGICASY	0,8514
B07	HLA-B*35:01	687	VASQSIIAY	0,9634
B07	HLA-B*35:01	699	LGAENSVAY	0,8303
B07	HLA-B*35:01	714	IPTNFTISV	0,5007
B07	HLA-B*35:01	861	LPPLLTDEM	0,6186
B07	HLA-B*35:01	865	LTDEMIAQY	0,6118
B07	HLA-B*35:01	892	AALQIPFAM	0,5789
B07	HLA-B*35:01	895	QIPFAMQMAY	0,6098
B07	HLA-B*35:01	896	IPFAMQMAY	0,9872
B07	HLA-B*35:01	898	FAMQMAYRF	0,7448
B07	HLA-B*35:01	1052	FPQSAPHGVVF	0,8504
B07	HLA-B*35:01	1054	QSAPHGVVF	0,6307
B07	HLA-B*35:01	1095	FVSNGTHWF	0,5997
B07	HLA-B*35:03	84	LPFNDGVYF	0,8449
B07	HLA-B*35:03	714	IPTNFTISV	0,6549
B07	HLA-B*35:03	861	LPPLLTDEM	0,674
B07	HLA-B*35:03	1052	FPQSAPHGVVF	0,6557
B07	HLA-B*35:07	24	LPPAYTNSF	0,8786
B07	HLA-B*35:07	30	NSFTRGVYY	0,5335

B07	HLA-B*35:07	56	LPFFSNVTW	0,8069
B07	HLA-B*35:07	83	VLPFNDGVYF	0,7228
B07	HLA-B*35:07	84	LPFNDGVY	0,824
B07	HLA-B*35:07	84	LPFNDGVYF	0,9877
B07	HLA-B*35:07	162	SANNCTFEY	0,6698
B07	HLA-B*35:07	192	FVFKNIDGY	0,7837
B07	HLA-B*35:07	229	LPIGINITRF	0,8394
B07	HLA-B*35:07	258	WTAGAAAYY	0,5393
B07	HLA-B*35:07	271	QPRTFLLKY	0,6565
B07	HLA-B*35:07	321	QPTESIVRF	0,9476
B07	HLA-B*35:07	343	NATRFASVY	0,7573
B07	HLA-B*35:07	361	CVADYSVLY	0,5167
B07	HLA-B*35:07	478	TPCNGVEGF	0,5961
B07	HLA-B*35:07	604	TSNQVAVLY	0,5492
B07	HLA-B*35:07	625	HADQLTPTW	0,7176
B07	HLA-B*35:07	664	IPIGAGICASY	0,8514
B07	HLA-B*35:07	687	VASQSIIAY	0,9634
B07	HLA-B*35:07	699	LGAENSVAY	0,8303
B07	HLA-B*35:07	714	IPTNFTISV	0,5007
B07	HLA-B*35:07	861	LPPLLTDEM	0,6186
B07	HLA-B*35:07	865	LTDEMIAQY	0,6118
B07	HLA-B*35:07	892	AALQIPFAM	0,5789
B07	HLA-B*35:07	895	QIPFAMQMAY	0,6098
B07	HLA-B*35:07	896	IPFAMQMAY	0,9872
B07	HLA-B*35:07	898	FAMQMAYRF	0,7448
B07	HLA-B*35:07	1052	FPQSAPHGVVF	0,8504
B07	HLA-B*35:07	1054	QSAPHGVVF	0,6307
B07	HLA-B*35:07	1095	FVSNGTHWF	0,5997
B07	HLA-B*35:08	24	LPPAYTNSF	0,797
B07	HLA-B*35:08	56	LPFFSNVTW	0,7097
B07	HLA-B*35:08	84	LPFNDGVY	0,6312
B07	HLA-B*35:08	84	LPFNDGVYF	0,9511
B07	HLA-B*35:08	162	SANNCTFEY	0,5112
B07	HLA-B*35:08	229	LPIGINITRF	0,6418
B07	HLA-B*35:08	321	QPTESIVRF	0,8721
B07	HLA-B*35:08	343	NATRFASVY	0,5359
B07	HLA-B*35:08	664	IPIGAGICASY	0,6771
B07	HLA-B*35:08	687	VASQSIIAY	0,8861
B07	HLA-B*35:08	699	LGAENSVAY	0,6356
B07	HLA-B*35:08	861	LPPLLTDEM	0,5504
B07	HLA-B*35:08	896	IPFAMQMAY	0,9572
B07	HLA-B*35:08	1052	FPQSAPHGVVF	0,787
B07	HLA-B*35:11	24	LPPAYTNSF	0,8352
B07	HLA-B*35:11	30	NSFTRGVYY	0,7008
B07	HLA-B*35:11	56	LPFFSNVTW	0,6542
B07	HLA-B*35:11	83	VLPFNDGVYF	0,6879
B07	HLA-B*35:11	84	LPFNDGVY	0,8263
B07	HLA-B*35:11	84	LPFNDGVYF	0,9718
B07	HLA-B*35:11	160	YSSANNCTF	0,5303
B07	HLA-B*35:11	162	SANNCTFEY	0,5536

B07	HLA-B*35:11	192	FVFKNIDGY	0,7481
B07	HLA-B*35:11	212	LVRDLPQGF	0,575
B07	HLA-B*35:11	229	LPIGINITRF	0,775
B07	HLA-B*35:11	258	WTAGAAAYY	0,5592
B07	HLA-B*35:11	261	GAAAYYVGY	0,5071
B07	HLA-B*35:11	271	QPRTFLLKY	0,8033
B07	HLA-B*35:11	321	QPTESIVRF	0,879
B07	HLA-B*35:11	343	NATRFASVY	0,812
B07	HLA-B*35:11	366	SVLYNSASF	0,7009
B07	HLA-B*35:11	604	TSNQVAVLY	0,5291
B07	HLA-B*35:11	664	IPIGAGICASY	0,8485
B07	HLA-B*35:11	686	SVASQSIIAY	0,5827
B07	HLA-B*35:11	687	VASQSIIAY	0,9537
B07	HLA-B*35:11	699	LGAENSVAY	0,8145
B07	HLA-B*35:11	710	NSIAIPTNF	0,608
B07	HLA-B*35:11	865	LTDEMIAQY	0,5297
B07	HLA-B*35:11	869	MIAQYTSAL	0,5203
B07	HLA-B*35:11	886	WTFGAGAAL	0,5144
B07	HLA-B*35:11	892	AALQIPFAM	0,6266
B07	HLA-B*35:11	895	QIPFAMQMAY	0,6561
B07	HLA-B*35:11	896	IPFAMQMAY	0,9842
B07	HLA-B*35:11	898	FAMQMAYRF	0,6952
B07	HLA-B*35:11	1021	SANLAATKM	0,6657
B07	HLA-B*35:11	1052	FPQSAPHGVVF	0,7606
B07	HLA-B*35:11	1054	QSAPHGVVF	0,7902
B07	HLA-B*35:11	1095	FVSNGTHWF	0,6488
B07	HLA-B*35:11	1113	QIITTDNTF	0,5819
B07	HLA-B*35:14	24	LPPAYTNSF	0,7489
B07	HLA-B*35:14	84	LPFNDGVY	0,6173
B07	HLA-B*35:14	84	LPFNDGVYF	0,8767
B07	HLA-B*35:14	192	FVFKNIDGY	0,677
B07	HLA-B*35:14	229	LPIGINITRF	0,6057
B07	HLA-B*35:14	258	WTAGAAAYY	0,5528
B07	HLA-B*35:14	343	NATRFASVY	0,7039
B07	HLA-B*35:14	366	SVLYNSASF	0,5417
B07	HLA-B*35:14	664	IPIGAGICASY	0,6706
B07	HLA-B*35:14	686	SVASQSIIAY	0,5487
B07	HLA-B*35:14	687	VASQSIIAY	0,8589
B07	HLA-B*35:14	699	LGAENSVAY	0,6846
B07	HLA-B*35:14	896	IPFAMQMAY	0,9083
B07	HLA-B*35:14	898	FAMQMAYRF	0,501
B07	HLA-B*35:14	1052	FPQSAPHGVVF	0,5835
B07	HLA-B*35:14	1054	QSAPHGVVF	0,6632
B07	HLA-B*35:14	1095	FVSNGTHWF	0,5633
B07	HLA-B*35:15	24	LPPAYTNSF	0,7919
B07	HLA-B*35:15	56	LPFFSNVTW	0,7224
B07	HLA-B*35:15	83	VLPFNDGVYF	0,6829
B07	HLA-B*35:15	84	LPFNDGVY	0,7275
B07	HLA-B*35:15	84	LPFNDGVYF	0,9676
B07	HLA-B*35:15	162	SANNCTFEY	0,5711

B07	HLA-B*35:15	192	FVFKNIDGY	0,5935
B07	HLA-B*35:15	229	LPIGINITRF	0,7637
B07	HLA-B*35:15	321	QPTESIVRF	0,8872
B07	HLA-B*35:15	343	NATRFASVY	0,5685
B07	HLA-B*35:15	664	IPIGAGICASY	0,7825
B07	HLA-B*35:15	687	VASQSIIAY	0,9029
B07	HLA-B*35:15	699	LGAENSVAY	0,6765
B07	HLA-B*35:15	714	IPTNFTISV	0,5146
B07	HLA-B*35:15	861	LPPLLTDEM	0,5696
B07	HLA-B*35:15	892	AALQIPFAM	0,6734
B07	HLA-B*35:15	895	QIPFAMQMAY	0,5998
B07	HLA-B*35:15	896	IPFAMQMAY	0,9714
B07	HLA-B*35:15	898	FAMQMAYRF	0,665
B07	HLA-B*35:15	1052	FPQSAPHGVVF	0,781
B07	HLA-B*35:15	1054	QSAPHGVVF	0,5581
B07	HLA-B*35:21	24	LPPAYTNSF	0,8395
B07	HLA-B*35:21	30	NSFTRGVYY	0,7343
B07	HLA-B*35:21	56	LPFFSNVTW	0,7008
B07	HLA-B*35:21	83	VLPFNDGVYF	0,6004
B07	HLA-B*35:21	84	LPFNDGVY	0,8356
B07	HLA-B*35:21	84	LPFNDGVYF	0,9541
B07	HLA-B*35:21	138	DPFLGVYY	0,7423
B07	HLA-B*35:21	192	FVFKNIDGY	0,6843
B07	HLA-B*35:21	229	LPIGINITRF	0,7683
B07	HLA-B*35:21	271	QPRTFLLKY	0,8503
B07	HLA-B*35:21	321	QPTESIVRF	0,8652
B07	HLA-B*35:21	343	NATRFASVY	0,817
B07	HLA-B*35:21	366	SVLYNSASF	0,6264
B07	HLA-B*35:21	487	NCYFPLQSY	0,57
B07	HLA-B*35:21	490	FPLQSYGF	0,5592
B07	HLA-B*35:21	664	IPIGAGICASY	0,8085
B07	HLA-B*35:21	686	SVASQSIIAY	0,5007
B07	HLA-B*35:21	687	VASQSIIAY	0,9249
B07	HLA-B*35:21	699	LGAENSVAY	0,7697
B07	HLA-B*35:21	869	MIAQYTSAL	0,5073
B07	HLA-B*35:21	892	AALQIPFAM	0,5807
B07	HLA-B*35:21	895	QIPFAMQMAY	0,688
B07	HLA-B*35:21	896	IPFAMQMAY	0,9842
B07	HLA-B*35:21	897	PFAMQMAY	0,6134
B07	HLA-B*35:21	898	FAMQMAYRF	0,7194
B07	HLA-B*35:21	1021	SANLAATKM	0,5645
B07	HLA-B*35:21	1052	FPQSAPHGVVF	0,7764
B07	HLA-B*35:21	1054	QSAPHGVVF	0,7008
B07	HLA-B*35:21	1095	FVSNGTHWF	0,5717
B07	HLA-B*35:21	1130	IGIVNNTVY	0,5011
B07	HLA-B*35:22	24	LPPAYTNSF	0,6758
B07	HLA-B*35:22	84	LPFNDGVYF	0,8301
B07	HLA-B*35:22	216	LPQGFSAL	0,5263
B07	HLA-B*35:22	714	IPTNFTISV	0,8019
B07	HLA-B*35:22	861	LPPLLTDEM	0,6575

B07	HLA-B*35:22	896	IPFAMQMAY	0,568
B07	HLA-B*35:22	1052	FPQSAPHGV	0,7661
B07	HLA-B*35:22	1052	FPQSAPHGVVF	0,6166
B07	HLA-B*35:24	24	LPPAYTNSF	0,8659
B07	HLA-B*35:24	30	NSFTRGVYY	0,6
B07	HLA-B*35:24	56	LPFFSNVTW	0,8189
B07	HLA-B*35:24	56	LPFFSNVTWF	0,517
B07	HLA-B*35:24	83	VLPFNDGVYF	0,6239
B07	HLA-B*35:24	84	LPFNDGVY	0,8242
B07	HLA-B*35:24	84	LPFNDGVYF	0,9759
B07	HLA-B*35:24	138	DPFLGVYY	0,7649
B07	HLA-B*35:24	138	DPFLGVYYH	0,6062
B07	HLA-B*35:24	162	SANNCTFEY	0,5434
B07	HLA-B*35:24	192	FVFKNIDGY	0,6882
B07	HLA-B*35:24	229	LPIGINITRF	0,8081
B07	HLA-B*35:24	271	QPRTFLLKY	0,7209
B07	HLA-B*35:24	321	QPTESIVRF	0,9142
B07	HLA-B*35:24	343	NATRFASVY	0,7681
B07	HLA-B*35:24	490	FPLQSYGF	0,6001
B07	HLA-B*35:24	604	TSNQVAVLY	0,5175
B07	HLA-B*35:24	625	HADQLTPTW	0,6622
B07	HLA-B*35:24	664	IPIGAGICASY	0,7856
B07	HLA-B*35:24	687	VASQSIIAY	0,9318
B07	HLA-B*35:24	699	LGAENSVAY	0,7685
B07	HLA-B*35:24	714	IPTNFTISV	0,5262
B07	HLA-B*35:24	861	LPPLLTDEM	0,5723
B07	HLA-B*35:24	865	LTDEMIAQY	0,5411
B07	HLA-B*35:24	892	AALQIPFAM	0,5331
B07	HLA-B*35:24	895	QIPFAMQMAY	0,6479
B07	HLA-B*35:24	896	IPFAMQMAY	0,987
B07	HLA-B*35:24	897	PFAMQMAY	0,5448
B07	HLA-B*35:24	898	FAMQMAYRF	0,7695
B07	HLA-B*35:24	1052	FPQSAPHGVVF	0,8489
B07	HLA-B*35:24	1054	QSAPHGVVF	0,5377
B07	HLA-B*35:24	1095	FVSNGTHWF	0,5268
B07	HLA-B*35:31	38	YPDKVFRSSVL	0,5154
B07	HLA-B*35:31	84	LPFNDGVYF	0,7397
B07	HLA-B*35:31	216	LPQGFSAL	0,5549
B07	HLA-B*35:31	714	IPTNFTISV	0,825
B07	HLA-B*35:31	861	LPPLLTDEM	0,5885
B07	HLA-B*35:31	892	AALQIPFAM	0,5669
B07	HLA-B*35:31	1052	FPQSAPHGV	0,7222
B07	HLA-B*35:31	1052	FPQSAPHGVVF	0,5623
B07	HLA-B*35:32	24	LPPAYTNSF	0,9052
B07	HLA-B*35:32	30	NSFTRGVYY	0,5702
B07	HLA-B*35:32	56	LPFFSNVTW	0,8087
B07	HLA-B*35:32	56	LPFFSNVTWF	0,5164
B07	HLA-B*35:32	83	VLPFNDGVYF	0,7561
B07	HLA-B*35:32	84	LPFNDGVY	0,8314
B07	HLA-B*35:32	84	LPFNDGVYF	0,9881

B07	HLA-B*35:32	160	YSSANNCTF	0,5049
B07	HLA-B*35:32	162	SANNCTFEY	0,7098
B07	HLA-B*35:32	192	FVFKNIDGY	0,8032
B07	HLA-B*35:32	229	LPIGINITRF	0,8498
B07	HLA-B*35:32	258	WTAGAAAYY	0,5683
B07	HLA-B*35:32	271	QPRTFLLKY	0,6724
B07	HLA-B*35:32	321	QPTESIVRF	0,9486
B07	HLA-B*35:32	343	NATRFASVY	0,7659
B07	HLA-B*35:32	361	CVADYSVLY	0,53
B07	HLA-B*35:32	478	TPCNGVEGF	0,6358
B07	HLA-B*35:32	490	FPLQSYGF	0,5082
B07	HLA-B*35:32	604	TSNQVAVLY	0,5842
B07	HLA-B*35:32	625	HADQLTPTW	0,7442
B07	HLA-B*35:32	664	IPIGAGICASY	0,8564
B07	HLA-B*35:32	686	SVASQSIIAY	0,5225
B07	HLA-B*35:32	687	VASQSIIAY	0,9698
B07	HLA-B*35:32	699	LGAENSVAY	0,863
B07	HLA-B*35:32	710	NSIAIPTNF	0,5244
B07	HLA-B*35:32	714	IPTNFTISV	0,5438
B07	HLA-B*35:32	861	LPPLLTDEM	0,6652
B07	HLA-B*35:32	865	LTDEMIAQY	0,6517
B07	HLA-B*35:32	892	AALQIPFAM	0,6373
B07	HLA-B*35:32	895	QIPFAMQMAY	0,599
B07	HLA-B*35:32	896	IPFAMQMAY	0,9853
B07	HLA-B*35:32	898	FAMQMAYRF	0,7754
B07	HLA-B*35:32	1021	SANLAATKM	0,5136
B07	HLA-B*35:32	1052	FPQSAPHGV	0,5544
B07	HLA-B*35:32	1052	FPQSAPHGVVF	0,8763
B07	HLA-B*35:32	1054	QSAPHGVVF	0,697
B07	HLA-B*35:32	1095	FVSNGTHWF	0,6496
B07	HLA-B*35:32	1113	QIITTDNTF	0,5211
B07	HLA-B*35:33	714	IPTNFTISV	0,6955
B07	HLA-B*35:33	861	LPPLLTDEM	0,589
B07	HLA-B*35:35	24	LPPAYTNSF	0,8931
B07	HLA-B*35:35	30	NSFTRGVYY	0,6432
B07	HLA-B*35:35	38	YPDKVFRSS	0,5022
B07	HLA-B*35:35	56	LPFFSNVTW	0,8081
B07	HLA-B*35:35	56	LPFFSNVTWF	0,5997
B07	HLA-B*35:35	81	NPVLPFNDGVYF	0,5813
B07	HLA-B*35:35	83	VLPFNDGVYF	0,7798
B07	HLA-B*35:35	84	LPFNDGVY	0,8427
B07	HLA-B*35:35	84	LPFNDGVYF	0,987
B07	HLA-B*35:35	160	YSSANNCTF	0,5537
B07	HLA-B*35:35	162	SANNCTFEY	0,7379
B07	HLA-B*35:35	192	FVFKNIDGY	0,7847
B07	HLA-B*35:35	196	NIDGYFKIY	0,5682
B07	HLA-B*35:35	229	LPIGINITRF	0,8518
B07	HLA-B*35:35	258	WTAGAAAYY	0,6006
B07	HLA-B*35:35	261	GAAAYYVGY	0,5655
B07	HLA-B*35:35	271	QPRTFLLKY	0,7481

B07	HLA-B*35:35	321	QPTESIVRF	0,9497
B07	HLA-B*35:35	329	FPNITNLCPF	0,5517
B07	HLA-B*35:35	343	NATRFASVY	0,791
B07	HLA-B*35:35	361	CVADYSVLY	0,579
B07	HLA-B*35:35	366	SVLYNSASF	0,6081
B07	HLA-B*35:35	392	FTNVYADSF	0,5023
B07	HLA-B*35:35	462	KPFERDISTEIY	0,6963
B07	HLA-B*35:35	478	TPCNGVEGF	0,6736
B07	HLA-B*35:35	490	FPLQSYGF	0,6408
B07	HLA-B*35:35	604	TSNQVAVLY	0,653
B07	HLA-B*35:35	625	HADQLTPTW	0,77
B07	HLA-B*35:35	652	GAEHVNNSY	0,6983
B07	HLA-B*35:35	664	IPIGAGICASY	0,8472
B07	HLA-B*35:35	686	SVASQSIIAY	0,5942
B07	HLA-B*35:35	687	VASQSIIAY	0,9671
B07	HLA-B*35:35	699	LGAENSVAY	0,8503
B07	HLA-B*35:35	710	NSIAIPTNF	0,6147
B07	HLA-B*35:35	714	IPTNFTISV	0,6668
B07	HLA-B*35:35	861	LPPLLTDEM	0,6989
B07	HLA-B*35:35	865	LTDEMIAQY	0,7051
B07	HLA-B*35:35	869	MIAQYTSAL	0,5063
B07	HLA-B*35:35	886	WTFGAGAAL	0,5733
B07	HLA-B*35:35	892	AALQIPFAM	0,7769
B07	HLA-B*35:35	895	QIPFAMQMAY	0,6833
B07	HLA-B*35:35	896	IPFAMQMAY	0,9858
B07	HLA-B*35:35	897	PFAMQMAY	0,5282
B07	HLA-B*35:35	898	FAMQMAYRF	0,8293
B07	HLA-B*35:35	1021	SANLAATKM	0,6342
B07	HLA-B*35:35	1052	FPQSAPHGV	0,6179
B07	HLA-B*35:35	1052	FPQSAPHGVVF	0,8964
B07	HLA-B*35:35	1054	QSAPHGVVF	0,7525
B07	HLA-B*35:35	1056	APHGVVFLHVTY	0,6046
B07	HLA-B*35:35	1089	FPREGVFVS	0,5258
B07	HLA-B*35:35	1095	FVSNGTHWF	0,7045
B07	HLA-B*35:35	1113	QIITTDNTF	0,592
B07	HLA-B*35:36	84	LPFNDGVYF	0,8449
B07	HLA-B*35:36	714	IPTNFTISV	0,6549
B07	HLA-B*35:36	861	LPPLLTDEM	0,674
B07	HLA-B*35:36	1052	FPQSAPHGVVF	0,6557
B07	HLA-B*35:38	1052	FPQSAPHGVVF	0,6186
B07	HLA-B*35:41	24	LPPAYTNSF	0,9203
B07	HLA-B*35:41	30	NSFTRGVYY	0,69
B07	HLA-B*35:41	56	LPFFSNVTW	0,8713
B07	HLA-B*35:41	56	LPFFSNVTWF	0,6188
B07	HLA-B*35:41	81	NPVLPFNDGVYF	0,5272
B07	HLA-B*35:41	83	VLPFNDGVYF	0,7855
B07	HLA-B*35:41	84	LPFNDGVY	0,8727
B07	HLA-B*35:41	84	LPFNDGVYF	0,9927
B07	HLA-B*35:41	160	YSSANNCTF	0,5877
B07	HLA-B*35:41	162	SANNCTFEY	0,7881

B07	HLA-B*35:41	192	FVFKNIDGY	0,8905
B07	HLA-B*35:41	196	NIDGYFKIY	0,577
B07	HLA-B*35:41	229	LPIGINITRF	0,8935
B07	HLA-B*35:41	258	WTAGAAAYY	0,7113
B07	HLA-B*35:41	261	GAAAYYVGY	0,5904
B07	HLA-B*35:41	271	QPRTFLLKY	0,731
B07	HLA-B*35:41	321	QPTESIVRF	0,9644
B07	HLA-B*35:41	329	FPNITNLCPF	0,5798
B07	HLA-B*35:41	343	NATRFASVY	0,8709
B07	HLA-B*35:41	361	CVADYSVLY	0,6962
B07	HLA-B*35:41	366	SVLYNSASF	0,6032
B07	HLA-B*35:41	392	FTNVYADSF	0,5373
B07	HLA-B*35:41	462	KPFERDISTEIY	0,6461
B07	HLA-B*35:41	478	TPCNGVEGF	0,6721
B07	HLA-B*35:41	481	NGVEGFNCY	0,5089
B07	HLA-B*35:41	490	FPLQSYGF	0,5518
B07	HLA-B*35:41	604	TSNQVAVLY	0,7229
B07	HLA-B*35:41	625	HADQLTPTW	0,7962
B07	HLA-B*35:41	652	GAEHVNNSY	0,6893
B07	HLA-B*35:41	664	IPIGAGICASY	0,908
B07	HLA-B*35:41	686	SVASQSIIAY	0,6499
B07	HLA-B*35:41	687	VASQSIIAY	0,9842
B07	HLA-B*35:41	699	LGAENSVAY	0,9146
B07	HLA-B*35:41	710	NSIAIPTNF	0,6291
B07	HLA-B*35:41	714	IPTNFTISV	0,6147
B07	HLA-B*35:41	861	LPPLLTDEM	0,695
B07	HLA-B*35:41	865	LTDEMIAQY	0,7508
B07	HLA-B*35:41	892	AALQIPFAM	0,7116
B07	HLA-B*35:41	895	QIPFAMQMAY	0,7205
B07	HLA-B*35:41	896	IPFAMQMAY	0,993
B07	HLA-B*35:41	897	PFAMQMAY	0,5108
B07	HLA-B*35:41	898	FAMQMAYRF	0,8561
B07	HLA-B*35:41	1021	SANLAATKM	0,6172
B07	HLA-B*35:41	1052	FPQSAPHGV	0,5784
B07	HLA-B*35:41	1052	FPQSAPHGVVF	0,8981
B07	HLA-B*35:41	1054	QSAPHGVVF	0,7647
B07	HLA-B*35:41	1056	APHGVVFLHVTY	0,5536
B07	HLA-B*35:41	1089	FPREGVFVS	0,5633
B07	HLA-B*35:41	1095	FVSNGTHWF	0,7432
B07	HLA-B*35:41	1113	QIITTDNTF	0,6101
B07	HLA-B*35:41	1130	IGIVNNTVY	0,512
B07	HLA-B*35:42	24	LPPAYTNSF	0,8786
B07	HLA-B*35:42	30	NSFTRGVYY	0,5335
B07	HLA-B*35:42	56	LPFFSNVTW	0,8069
B07	HLA-B*35:42	83	VLPFNDGVYF	0,7228
B07	HLA-B*35:42	84	LPFNDGVY	0,824
B07	HLA-B*35:42	84	LPFNDGVYF	0,9877
B07	HLA-B*35:42	162	SANNCTFEY	0,6698
B07	HLA-B*35:42	192	FVFKNIDGY	0,7837
B07	HLA-B*35:42	229	LPIGINITRF	0,8394

B07	HLA-B*35:42	258	WTAGAAAYY	0,5393
B07	HLA-B*35:42	271	QPRTFLLKY	0,6565
B07	HLA-B*35:42	321	QPTESIVRF	0,9476
B07	HLA-B*35:42	343	NATRFASVY	0,7573
B07	HLA-B*35:42	361	CVADYSVLY	0,5167
B07	HLA-B*35:42	478	TPCNGVEGF	0,5961
B07	HLA-B*35:42	604	TSNQVAVLY	0,5492
B07	HLA-B*35:42	625	HADQLTPTW	0,7176
B07	HLA-B*35:42	664	IPIGAGICASY	0,8514
B07	HLA-B*35:42	687	VASQSIIAY	0,9634
B07	HLA-B*35:42	699	LGAENSVAY	0,8303
B07	HLA-B*35:42	714	IPTNFTISV	0,5007
B07	HLA-B*35:42	861	LPPLLTDEM	0,6186
B07	HLA-B*35:42	865	LTDEMIAQY	0,6118
B07	HLA-B*35:42	892	AALQIPFAM	0,5789
B07	HLA-B*35:42	895	QIPFAMQMAY	0,6098
B07	HLA-B*35:42	896	IPFAMQMAY	0,9872
B07	HLA-B*35:42	898	FAMQMAYRF	0,7448
B07	HLA-B*35:42	1052	FPQSAPHGVVF	0,8504
B07	HLA-B*35:42	1054	QSAPHGVVF	0,6307
B07	HLA-B*35:42	1095	FVSNGTHWF	0,5997
B07	HLA-B*35:43	24	LPPAYTNSF	0,7875
B07	HLA-B*35:43	30	NSFTRGVYY	0,5895
B07	HLA-B*35:43	84	LPFNDGVY	0,6447
B07	HLA-B*35:43	84	LPFNDGVYF	0,8888
B07	HLA-B*35:43	160	YSSANNCTF	0,5206
B07	HLA-B*35:43	192	FVFKNIDGY	0,7054
B07	HLA-B*35:43	229	LPIGINITRF	0,6427
B07	HLA-B*35:43	258	WTAGAAAYY	0,5829
B07	HLA-B*35:43	343	NATRFASVY	0,7189
B07	HLA-B*35:43	366	SVLYNSASF	0,6027
B07	HLA-B*35:43	664	IPIGAGICASY	0,6933
B07	HLA-B*35:43	686	SVASQSIIAY	0,5892
B07	HLA-B*35:43	687	VASQSIIAY	0,8891
B07	HLA-B*35:43	699	LGAENSVAY	0,7466
B07	HLA-B*35:43	896	IPFAMQMAY	0,9166
B07	HLA-B*35:43	898	FAMQMAYRF	0,543
B07	HLA-B*35:43	962	LVKQLSSNF	0,5124
B07	HLA-B*35:43	1052	FPQSAPHGVVF	0,6324
B07	HLA-B*35:43	1054	QSAPHGVVF	0,7377
B07	HLA-B*35:43	1095	FVSNGTHWF	0,6086
B07	HLA-B*35:43	1113	QIITTDNTF	0,5611
B07	HLA-B*35:45	84	LPFNDGVYF	0,7419
B07	HLA-B*35:45	687	VASQSIIAY	0,5885
B07	HLA-B*35:45	896	IPFAMQMAY	0,7957
B07	HLA-B*35:46	24	LPPAYTNSF	0,6364
B07	HLA-B*35:46	56	LPFFSNVTW	0,5165
B07	HLA-B*35:46	84	LPFNDGVYF	0,9096
B07	HLA-B*35:46	192	FVFKNIDGY	0,5961
B07	HLA-B*35:46	229	LPIGINITRF	0,5183

B07	HLA-B*35:46	321	QPTESIVRF	0,7199
B07	HLA-B*35:46	343	NATRFASVY	0,5076
B07	HLA-B*35:46	664	IPIGAGICASY	0,6105
B07	HLA-B*35:46	687	VASQSIIAY	0,8432
B07	HLA-B*35:46	699	LGAENSVAY	0,6048
B07	HLA-B*35:46	896	IPFAMQMAY	0,9239
B07	HLA-B*35:46	1052	FPQSAPHGVVF	0,5441
B07	HLA-B*35:46	1054	QSAPHGVVF	0,5022
B07	HLA-B*35:54	24	LPPAYTNSF	0,8786
B07	HLA-B*35:54	30	NSFTRGVYY	0,5335
B07	HLA-B*35:54	56	LPFFSNVTW	0,8069
B07	HLA-B*35:54	83	VLPFNDGVYF	0,7228
B07	HLA-B*35:54	84	LPFNDGVY	0,824
B07	HLA-B*35:54	84	LPFNDGVYF	0,9877
B07	HLA-B*35:54	162	SANNCTFEY	0,6698
B07	HLA-B*35:54	192	FVFKNIDGY	0,7837
B07	HLA-B*35:54	229	LPIGINITRF	0,8394
B07	HLA-B*35:54	258	WTAGAAAYY	0,5393
B07	HLA-B*35:54	271	QPRTFLLKY	0,6565
B07	HLA-B*35:54	321	QPTESIVRF	0,9476
B07	HLA-B*35:54	343	NATRFASVY	0,7573
B07	HLA-B*35:54	361	CVADYSVLY	0,5167
B07	HLA-B*35:54	478	TPCNGVEGF	0,5961
B07	HLA-B*35:54	604	TSNQVAVLY	0,5492
B07	HLA-B*35:54	625	HADQLTPTW	0,7176
B07	HLA-B*35:54	664	IPIGAGICASY	0,8514
B07	HLA-B*35:54	687	VASQSIIAY	0,9634
B07	HLA-B*35:54	699	LGAENSVAY	0,8303
B07	HLA-B*35:54	714	IPTNFTISV	0,5007
B07	HLA-B*35:54	861	LPPLLTDEM	0,6186
B07	HLA-B*35:54	865	LTDEMIAQY	0,6118
B07	HLA-B*35:54	892	AALQIPFAM	0,5789
B07	HLA-B*35:54	895	QIPFAMQMAY	0,6098
B07	HLA-B*35:54	896	IPFAMQMAY	0,9872
B07	HLA-B*35:54	898	FAMQMAYRF	0,7448
B07	HLA-B*35:54	1052	FPQSAPHGVVF	0,8504
B07	HLA-B*35:54	1054	QSAPHGVVF	0,6307
B07	HLA-B*35:54	1095	FVSNGTHWF	0,5997
B07	HLA-B*35:55	84	LPFNDGVYF	0,8449
B07	HLA-B*35:55	714	IPTNFTISV	0,6549
B07	HLA-B*35:55	861	LPPLLTDEM	0,674
B07	HLA-B*35:55	1052	FPQSAPHGVVF	0,6557
B07	HLA-B*35:57	24	LPPAYTNSF	0,8786
B07	HLA-B*35:57	30	NSFTRGVYY	0,5335
B07	HLA-B*35:57	56	LPFFSNVTW	0,8069
B07	HLA-B*35:57	83	VLPFNDGVYF	0,7228
B07	HLA-B*35:57	84	LPFNDGVY	0,824
B07	HLA-B*35:57	84	LPFNDGVYF	0,9877
B07	HLA-B*35:57	162	SANNCTFEY	0,6698
B07	HLA-B*35:57	192	FVFKNIDGY	0,7837

B07	HLA-B*35:57	229	LPIGINITRF	0,8394
B07	HLA-B*35:57	258	WTAGAAAYY	0,5393
B07	HLA-B*35:57	271	QPRTFLLKY	0,6565
B07	HLA-B*35:57	321	QPTESIVRF	0,9476
B07	HLA-B*35:57	343	NATRFASVY	0,7573
B07	HLA-B*35:57	361	CVADYSVLY	0,5167
B07	HLA-B*35:57	478	TPCNGVEGF	0,5961
B07	HLA-B*35:57	604	TSNQVAVLY	0,5492
B07	HLA-B*35:57	625	HADQLTPTW	0,7176
B07	HLA-B*35:57	664	IPIGAGICASY	0,8514
B07	HLA-B*35:57	687	VASQSIIAY	0,9634
B07	HLA-B*35:57	699	LGAENSVAY	0,8303
B07	HLA-B*35:57	714	IPTNFTISV	0,5007
B07	HLA-B*35:57	861	LPPLLTDEM	0,6186
B07	HLA-B*35:57	865	LTDEMIAQY	0,6118
B07	HLA-B*35:57	892	AALQIPFAM	0,5789
B07	HLA-B*35:57	895	QIPFAMQMAY	0,6098
B07	HLA-B*35:57	896	IPFAMQMAY	0,9872
B07	HLA-B*35:57	898	FAMQMAYRF	0,7448
B07	HLA-B*35:57	1052	FPQSAPHGVVF	0,8504
B07	HLA-B*35:57	1054	QSAPHGVVF	0,6307
B07	HLA-B*35:57	1095	FVSNGTHWF	0,5997
B07	HLA-B*35:61	24	LPPAYTNSF	0,797
B07	HLA-B*35:61	56	LPFFSNVTW	0,7097
B07	HLA-B*35:61	84	LPFNDGVY	0,6312
B07	HLA-B*35:61	84	LPFNDGVYF	0,9511
B07	HLA-B*35:61	162	SANNCTFEY	0,5112
B07	HLA-B*35:61	229	LPIGINITRF	0,6418
B07	HLA-B*35:61	321	QPTESIVRF	0,8721
B07	HLA-B*35:61	343	NATRFASVY	0,5359
B07	HLA-B*35:61	664	IPIGAGICASY	0,6771
B07	HLA-B*35:61	687	VASQSIIAY	0,8861
B07	HLA-B*35:61	699	LGAENSVAY	0,6356
B07	HLA-B*35:61	861	LPPLLTDEM	0,5504
B07	HLA-B*35:61	896	IPFAMQMAY	0,9572
B07	HLA-B*35:61	1052	FPQSAPHGVVF	0,787
B07	HLA-B*39:10	24	LPPAYTNSF	0,7059
B07	HLA-B*39:10	38	YPDKVFRSSV	0,6561
B07	HLA-B*39:10	38	YPDKVFRSSVL	0,6644
B07	HLA-B*39:10	84	LPFNDGVYF	0,7911
B07	HLA-B*39:10	208	TPINLVRDL	0,8013
B07	HLA-B*39:10	216	LPQGFSAL	0,7184
B07	HLA-B*39:10	229	LPIGINITRF	0,5061
B07	HLA-B*39:10	506	QPYRVVVL	0,6651
B07	HLA-B*39:10	714	IPTNFTISV	0,8392
B07	HLA-B*39:10	861	LPPLLTDEM	0,6235
B07	HLA-B*39:10	869	MIAQYTSAL	0,6143
B07	HLA-B*39:10	1052	FPQSAPHGV	0,8631
B07	HLA-B*39:10	1052	FPQSAPHGVV	0,603
B07	HLA-B*39:10	1052	FPQSAPHGVVF	0,7108

B07	HLA-B*39:10	1262	EPVLKGVKL	0,8325
B07	HLA-B*39:16	24	LPPAYTNSF	0,7059
B07	HLA-B*39:16	38	YPDKVFRSSV	0,6561
B07	HLA-B*39:16	38	YPDKVFRSSVL	0,6644
B07	HLA-B*39:16	84	LPFNDGVYF	0,7911
B07	HLA-B*39:16	208	TPINLVRDL	0,8013
B07	HLA-B*39:16	216	LPQGFSAL	0,7184
B07	HLA-B*39:16	229	LPIGINITRF	0,5061
B07	HLA-B*39:16	506	QPYRVVVL	0,6651
B07	HLA-B*39:16	714	IPTNFTISV	0,8392
B07	HLA-B*39:16	861	LPPLLTDEM	0,6235
B07	HLA-B*39:16	869	MIAQYTSAL	0,6143
B07	HLA-B*39:16	1052	FPQSAPHGV	0,8631
B07	HLA-B*39:16	1052	FPQSAPHGVV	0,603
B07	HLA-B*39:16	1052	FPQSAPHGVVF	0,7108
B07	HLA-B*39:16	1262	EPVLKGVKL	0,8325
B07	HLA-B*42:01	24	LPPAYTNSF	0,7878
B07	HLA-B*42:01	38	YPDKVFRSSV	0,6536
B07	HLA-B*42:01	84	LPFNDGVYF	0,7423
B07	HLA-B*42:01	208	TPINLVRDL	0,869
B07	HLA-B*42:01	216	LPQGFSAL	0,7808
B07	HLA-B*42:01	229	LPIGINITRF	0,5486
B07	HLA-B*42:01	506	QPYRVVVL	0,8243
B07	HLA-B*42:01	526	GPKKSTNLV	0,6419
B07	HLA-B*42:01	620	VPVAIHADQL	0,633
B07	HLA-B*42:01	714	IPTNFTISV	0,8435
B07	HLA-B*42:01	869	MIAQYTSAL	0,5968
B07	HLA-B*42:01	1052	FPQSAPHGV	0,7467
B07	HLA-B*42:01	1052	FPQSAPHGVVF	0,6491
B07	HLA-B*42:01	1056	APHGVVFL	0,7492
B07	HLA-B*42:01	1261	SEPVLKGVKL	0,7228
B07	HLA-B*42:01	1262	EPVLKGVKL	0,831
B07	HLA-B*42:04	38	YPDKVFRSSV	0,7244
B07	HLA-B*42:04	208	TPINLVRDL	0,7004
B07	HLA-B*42:04	216	LPQGFSAL	0,6226
B07	HLA-B*42:04	462	KPFERDIST	0,5873
B07	HLA-B*42:04	506	QPYRVVVL	0,6967
B07	HLA-B*42:04	526	GPKKSTNLV	0,662
B07	HLA-B*42:04	588	TPCSFGGVSV	0,5412
B07	HLA-B*42:04	664	IPIGAGICA	0,5648
B07	HLA-B*42:04	680	SPRRARSVA	0,6117
B07	HLA-B*42:04	714	IPTNFTISV	0,8825
B07	HLA-B*42:04	1052	FPQSAPHGV	0,8239
B07	HLA-B*42:04	1052	FPQSAPHGVV	0,6478
B07	HLA-B*42:04	1089	FPREGVFV	0,655
B07	HLA-B*42:04	1089	FPREGVFVS	0,5819
B07	HLA-B*42:05	24	LPPAYTNSF	0,7878
B07	HLA-B*42:05	38	YPDKVFRSSV	0,6536
B07	HLA-B*42:05	84	LPFNDGVYF	0,7423
B07	HLA-B*42:05	208	TPINLVRDL	0,869

B07	HLA-B*42:05	216	LPQGFSAL	0,7808
B07	HLA-B*42:05	229	LPIGINITRF	0,5486
B07	HLA-B*42:05	506	QPYRVVVL	0,8243
B07	HLA-B*42:05	526	GPKKSTNLV	0,6419
B07	HLA-B*42:05	620	VPVAIHADQL	0,633
B07	HLA-B*42:05	714	IPTNFTISV	0,8435
B07	HLA-B*42:05	869	MIAQYTSAL	0,5968
B07	HLA-B*42:05	1052	FPQSAPHGV	0,7467
B07	HLA-B*42:05	1052	FPQSAPHGVVF	0,6491
B07	HLA-B*42:05	1056	APHGVVFL	0,7492
B07	HLA-B*42:05	1261	SEPVLKGVKL	0,7228
B07	HLA-B*42:05	1262	EPVLKGVKL	0,831
B07	HLA-B*51:01	712	IAIPTNFTI	0,8936
B07	HLA-B*51:01	714	IPTNFTISV	0,9219
B07	HLA-B*51:02	712	IAIPTNFTI	0,9071
B07	HLA-B*51:02	714	IPTNFTISV	0,9166
B07	HLA-B*51:02	923	IANQFNSAI	0,7018
B07	HLA-B*51:02	1052	FPQSAPHGV	0,7998
B07	HLA-B*51:02	1052	FPQSAPHGVV	0,6058
B07	HLA-B*51:05	425	LPDDFTGCVI	0,5315
B07	HLA-B*51:09	712	IAIPTNFTI	0,7984
B07	HLA-B*51:09	714	IPTNFTISV	0,7885
B07	HLA-B*51:16	712	IAIPTNFTI	0,8496
B07	HLA-B*51:16	714	IPTNFTISV	0,882
B07	HLA-B*51:17	712	IAIPTNFTI	0,8936
B07	HLA-B*51:17	714	IPTNFTISV	0,9219
B07	HLA-B*51:18	712	IAIPTNFTI	0,8936
B07	HLA-B*51:18	714	IPTNFTISV	0,9219
B07	HLA-B*51:19	56	LPFFSNVTW	0,5994
B07	HLA-B*51:19	84	LPFNDGVYF	0,7163
B07	HLA-B*51:19	712	IAIPTNFTI	0,8629
B07	HLA-B*51:19	714	IPTNFTISV	0,8404
B07	HLA-B*51:21	712	IAIPTNFTI	0,8586
B07	HLA-B*51:21	714	IPTNFTISV	0,8761
B07	HLA-B*51:24	712	IAIPTNFTI	0,8936
B07	HLA-B*51:24	714	IPTNFTISV	0,9219
B07	HLA-B*51:26	712	IAIPTNFTI	0,8936
B07	HLA-B*51:26	714	IPTNFTISV	0,9219
B07	HLA-B*51:28	712	IAIPTNFTI	0,8936
B07	HLA-B*51:28	714	IPTNFTISV	0,9219
B07	HLA-B*51:29	425	LPDDFTGCVI	0,6074
B07	HLA-B*51:29	714	IPTNFTISV	0,8847
B07	HLA-B*51:30	712	IAIPTNFTI	0,8936
B07	HLA-B*51:30	714	IPTNFTISV	0,9219
B07	HLA-B*51:31	712	IAIPTNFTI	0,8324
B07	HLA-B*51:31	714	IPTNFTISV	0,8262
B07	HLA-B*51:32	712	IAIPTNFTI	0,8936
B07	HLA-B*51:32	714	IPTNFTISV	0,9219
B07	HLA-B*51:33	712	IAIPTNFTI	0,8936
B07	HLA-B*51:33	714	IPTNFTISV	0,9219

B07	HLA-B*51:34	712	IAIPTNFTI	0,8619
B07	HLA-B*51:34	714	IPTNFTISV	0,8627
B07	HLA-B*51:34	1052	FPQSAPHGVV	0,5175
B07	HLA-B*51:35	712	IAIPTNFTI	0,8936
B07	HLA-B*51:35	714	IPTNFTISV	0,9219
B07	HLA-B*51:36	714	IPTNFTISV	0,8244
B07	HLA-B*51:36	1052	FPQSAPHGVV	0,5349
B07	HLA-B*51:38	712	IAIPTNFTI	0,8936
B07	HLA-B*51:38	714	IPTNFTISV	0,9219
B07	HLA-B*53:01	24	LPPAYTNSF	0,7097
B07	HLA-B*53:01	55	FLPFFSNVTW	0,7688
B07	HLA-B*53:01	56	LPFFSNVTW	0,9556
B07	HLA-B*53:01	56	LPFFSNVTWF	0,502
B07	HLA-B*53:01	83	VLPFNDGVYF	0,7116
B07	HLA-B*53:01	84	LPFNDGVYF	0,967
B07	HLA-B*53:01	229	LPIGINITRF	0,8328
B07	HLA-B*53:01	250	TPGDSSSGW	0,8069
B07	HLA-B*53:01	320	VQPTESIVRF	0,5847
B07	HLA-B*53:01	321	QPTESIVRF	0,9157
B07	HLA-B*53:01	625	HADQLTPTW	0,9493
B07	HLA-B*53:01	714	IPTNFTISV	0,5509
B07	HLA-B*53:01	878	LAGTITSGW	0,6015
B07	HLA-B*53:01	896	IPFAMQMAY	0,8495
B07	HLA-B*53:01	898	FAMQMAYRF	0,7049
B07	HLA-B*53:01	1052	FPQSAPHGV	0,6048
B07	HLA-B*53:01	1052	FPQSAPHGVVF	0,6896
B07	HLA-B*53:02	24	LPPAYTNSF	0,6754
B07	HLA-B*53:02	53	DLFLPFFSNVTW	0,5156
B07	HLA-B*53:02	55	FLPFFSNVTW	0,7377
B07	HLA-B*53:02	56	LPFFSNVTW	0,9419
B07	HLA-B*53:02	83	VLPFNDGVYF	0,5685
B07	HLA-B*53:02	84	LPFNDGVYF	0,927
B07	HLA-B*53:02	229	LPIGINITRF	0,7712
B07	HLA-B*53:02	321	QPTESIVRF	0,8542
B07	HLA-B*53:02	625	HADQLTPTW	0,8778
B07	HLA-B*53:02	714	IPTNFTISV	0,5046
B07	HLA-B*53:02	896	IPFAMQMAY	0,8286
B07	HLA-B*53:02	898	FAMQMAYRF	0,7523
B07	HLA-B*53:02	1052	FPQSAPHGVVF	0,6331
B07	HLA-B*53:06	24	LPPAYTNSF	0,7435
B07	HLA-B*53:06	55	FLPFFSNVTW	0,611
B07	HLA-B*53:06	56	LPFFSNVTW	0,897
B07	HLA-B*53:06	84	LPFNDGVYF	0,9269
B07	HLA-B*53:06	229	LPIGINITRF	0,7787
B07	HLA-B*53:06	321	QPTESIVRF	0,8336
B07	HLA-B*53:06	490	FPLQSYGF	0,5333
B07	HLA-B*53:06	625	HADQLTPTW	0,7925
B07	HLA-B*53:06	896	IPFAMQMAY	0,8651
B07	HLA-B*53:06	898	FAMQMAYRF	0,7249
B07	HLA-B*53:06	1052	FPQSAPHGVVF	0,6456

B07	HLA-B*53:06	1095	FVSNGTHWF	0,5268
B07	HLA-B*53:08	24	LPPAYTNSF	0,7478
B07	HLA-B*53:08	55	FLPFFSNVTW	0,634
B07	HLA-B*53:08	56	LPFFSNVTW	0,9209
B07	HLA-B*53:08	83	VLPFNDGVYF	0,713
B07	HLA-B*53:08	84	LPFNDGVYF	0,9579
B07	HLA-B*53:08	229	LPIGINITRF	0,8211
B07	HLA-B*53:08	250	TPGDSSSGW	0,701
B07	HLA-B*53:08	321	QPTESIVRF	0,8829
B07	HLA-B*53:08	625	HADQLTPTW	0,8712
B07	HLA-B*53:08	687	VASQSIIAY	0,7141
B07	HLA-B*53:08	712	IAIPTNFTI	0,5344
B07	HLA-B*53:08	714	IPTNFTISV	0,5417
B07	HLA-B*53:08	878	LAGTITSGW	0,576
B07	HLA-B*53:08	896	IPFAMQMAY	0,8758
B07	HLA-B*53:08	898	FAMQMAYRF	0,6831
B07	HLA-B*53:08	1052	FPQSAPHGVVF	0,6472
B07	HLA-B*53:08	1095	FVSNGTHWF	0,6097
B07	HLA-B*53:10	24	LPPAYTNSF	0,7097
B07	HLA-B*53:10	55	FLPFFSNVTW	0,7688
B07	HLA-B*53:10	56	LPFFSNVTW	0,9556
B07	HLA-B*53:10	56	LPFFSNVTWF	0,502
B07	HLA-B*53:10	83	VLPFNDGVYF	0,7116
B07	HLA-B*53:10	84	LPFNDGVYF	0,967
B07	HLA-B*53:10	229	LPIGINITRF	0,8328
B07	HLA-B*53:10	250	TPGDSSSGW	0,8069
B07	HLA-B*53:10	320	VQPTESIVRF	0,5847
B07	HLA-B*53:10	321	QPTESIVRF	0,9157
B07	HLA-B*53:10	625	HADQLTPTW	0,9493
B07	HLA-B*53:10	714	IPTNFTISV	0,5509
B07	HLA-B*53:10	878	LAGTITSGW	0,6015
B07	HLA-B*53:10	896	IPFAMQMAY	0,8495
B07	HLA-B*53:10	898	FAMQMAYRF	0,7049
B07	HLA-B*53:10	1052	FPQSAPHGV	0,6048
B07	HLA-B*53:10	1052	FPQSAPHGVVF	0,6896
B07	HLA-B*54:01	38	YPDKVFRSS	0,5631
B07	HLA-B*54:01	38	YPDKVFRSSV	0,551
B07	HLA-B*54:01	84	LPFNDGVYFA	0,8147
B07	HLA-B*54:01	336	CPFGEVFNA	0,675
B07	HLA-B*54:01	506	QPYRVVVLS	0,517
B07	HLA-B*54:01	664	IPIGAGICA	0,7761
B07	HLA-B*54:01	714	IPTNFTISV	0,8556
B07	HLA-B*54:01	1052	FPQSAPHGV	0,7788
B07	HLA-B*54:01	1052	FPQSAPHGVV	0,5637
B07	HLA-B*54:01	1089	FPREGVFV	0,581
B07	HLA-B*54:01	1089	FPREGVFVS	0,826
B07	HLA-B*54:03	38	YPDKVFRSS	0,5065
B07	HLA-B*54:03	84	LPFNDGVYFA	0,7103
B07	HLA-B*54:03	336	CPFGEVFNA	0,5864
B07	HLA-B*54:03	664	IPIGAGICA	0,6982

B07	HLA-B*54:03	714	IPTNFTISV	0,759
B07	HLA-B*54:03	1052	FPQSAPHGV	0,7099
B07	HLA-B*54:03	1089	FPREGVFVS	0,7861
B07	HLA-B*54:04	38	YPDKVFRSSV	0,6067
B07	HLA-B*54:04	84	LPFNDGVYF	0,6325
B07	HLA-B*54:04	84	LPFNDGVYFA	0,7208
B07	HLA-B*54:04	336	CPFGEVFNA	0,5184
B07	HLA-B*54:04	664	IPIGAGICA	0,7162
B07	HLA-B*54:04	714	IPTNFTISV	0,8759
B07	HLA-B*54:04	1052	FPQSAPHGV	0,8116
B07	HLA-B*54:04	1052	FPQSAPHGVV	0,6221
B07	HLA-B*54:04	1089	FPREGVFV	0,626
B07	HLA-B*54:04	1089	FPREGVFVS	0,7032
B07	HLA-B*54:06	24	LPPAYTNSF	0,5438
B07	HLA-B*54:06	84	LPFNDGVYF	0,7179
B07	HLA-B*54:06	271	QPRTFLLKY	0,5428
B07	HLA-B*54:06	687	VASQSIIAY	0,5887
B07	HLA-B*54:06	896	IPFAMQMAY	0,8139
B07	HLA-B*54:07	38	YPDKVFRSS	0,5631
B07	HLA-B*54:07	38	YPDKVFRSSV	0,551
B07	HLA-B*54:07	84	LPFNDGVYFA	0,8147
B07	HLA-B*54:07	336	CPFGEVFNA	0,675
B07	HLA-B*54:07	506	QPYRVVVLS	0,517
B07	HLA-B*54:07	664	IPIGAGICA	0,7761
B07	HLA-B*54:07	714	IPTNFTISV	0,8556
B07	HLA-B*54:07	1052	FPQSAPHGV	0,7788
B07	HLA-B*54:07	1052	FPQSAPHGVV	0,5637
B07	HLA-B*54:07	1089	FPREGVFV	0,581
B07	HLA-B*54:07	1089	FPREGVFVS	0,826
B07	HLA-B*55:01	664	IPIGAGICA	0,566
B07	HLA-B*55:01	680	SPRRARSVA	0,6445
B07	HLA-B*55:01	714	IPTNFTISV	0,699
B07	HLA-B*55:01	1052	FPQSAPHGV	0,551
B07	HLA-B*55:01	1089	FPREGVFVS	0,6525
B07	HLA-B*55:02	38	YPDKVFRSS	0,6589
B07	HLA-B*55:02	38	YPDKVFRSSV	0,5249
B07	HLA-B*55:02	84	LPFNDGVYF	0,6389
B07	HLA-B*55:02	84	LPFNDGVYFA	0,6565
B07	HLA-B*55:02	336	CPFGEVFNA	0,5229
B07	HLA-B*55:02	462	KPFERDIST	0,6378
B07	HLA-B*55:02	506	QPYRVVVLS	0,527
B07	HLA-B*55:02	664	IPIGAGICA	0,7531
B07	HLA-B*55:02	714	IPTNFTISV	0,8516
B07	HLA-B*55:02	896	IPFAMQMAY	0,5572
B07	HLA-B*55:02	1052	FPQSAPHGV	0,758
B07	HLA-B*55:02	1052	FPQSAPHGVV	0,5174
B07	HLA-B*55:02	1089	FPREGVFV	0,5413
B07	HLA-B*55:02	1089	FPREGVFVS	0,8144
B07	HLA-B*55:03	714	IPTNFTISV	0,6188
B07	HLA-B*55:03	1052	FPQSAPHGV	0,5151

B07	HLA-B*55:03	1089	FPREGVFVS	0,531
B07	HLA-B*55:04	24	LPPAYTNSF	0,7178
B07	HLA-B*55:04	38	YPDKVFRSSV	0,6017
B07	HLA-B*55:04	56	LPFFSNVTW	0,5466
B07	HLA-B*55:04	83	VLPFNDGVYF	0,5131
B07	HLA-B*55:04	84	LPFNDGVYF	0,8753
B07	HLA-B*55:04	208	TPINLVRDL	0,8684
B07	HLA-B*55:04	216	LPQGFSAL	0,6991
B07	HLA-B*55:04	229	LPIGINITRF	0,7322
B07	HLA-B*55:04	321	QPTESIVRF	0,7159
B07	HLA-B*55:04	462	KPFERDIST	0,5424
B07	HLA-B*55:04	506	QPYRVVVL	0,7157
B07	HLA-B*55:04	620	VPVAIHADQL	0,6147
B07	HLA-B*55:04	714	IPTNFTISV	0,896
B07	HLA-B*55:04	869	MIAQYTSAL	0,5791
B07	HLA-B*55:04	892	AALQIPFAM	0,5124
B07	HLA-B*55:04	896	IPFAMQMAY	0,6175
B07	HLA-B*55:04	1052	FPQSAPHGV	0,8055
B07	HLA-B*55:04	1052	FPQSAPHGVVF	0,6991
B07	HLA-B*55:04	1056	APHGVVFL	0,6967
B07	HLA-B*55:04	1089	FPREGVFVS	0,5475
B07	HLA-B*55:04	1261	SEPVLKGVKL	0,5737
B07	HLA-B*55:04	1262	EPVLKGVKL	0,7445
B07	HLA-B*55:05	664	IPIGAGICA	0,566
B07	HLA-B*55:05	680	SPRRARSVA	0,6445
B07	HLA-B*55:05	714	IPTNFTISV	0,699
B07	HLA-B*55:05	1052	FPQSAPHGV	0,551
B07	HLA-B*55:05	1089	FPREGVFVS	0,6525
B07	HLA-B*55:07	38	YPDKVFRSS	0,5631
B07	HLA-B*55:07	38	YPDKVFRSSV	0,551
B07	HLA-B*55:07	84	LPFNDGVYFA	0,8147
B07	HLA-B*55:07	336	CPFGEVFNA	0,675
B07	HLA-B*55:07	506	QPYRVVVLS	0,517
B07	HLA-B*55:07	664	IPIGAGICA	0,7761
B07	HLA-B*55:07	714	IPTNFTISV	0,8556
B07	HLA-B*55:07	1052	FPQSAPHGV	0,7788
B07	HLA-B*55:07	1052	FPQSAPHGVV	0,5637
B07	HLA-B*55:07	1089	FPREGVFV	0,581
B07	HLA-B*55:07	1089	FPREGVFVS	0,826
B07	HLA-B*55:09	38	YPDKVFRSSV	0,6045
B07	HLA-B*55:09	664	IPIGAGICA	0,5924
B07	HLA-B*55:09	680	SPRRARSVA	0,7242
B07	HLA-B*55:09	714	IPTNFTISV	0,7624
B07	HLA-B*55:09	1052	FPQSAPHGV	0,5713
B07	HLA-B*55:09	1089	FPREGVFVS	0,6196
B07	HLA-B*55:10	38	YPDKVFRSS	0,6429
B07	HLA-B*55:10	84	LPFNDGVYF	0,6586
B07	HLA-B*55:10	84	LPFNDGVYFA	0,567
B07	HLA-B*55:10	462	KPFERDIST	0,64
B07	HLA-B*55:10	664	IPIGAGICA	0,7168

B07	HLA-B*55:10	714	IPTNFTISV	0,8293
B07	HLA-B*55:10	896	IPFAMQMAY	0,5528
B07	HLA-B*55:10	1052	FPQSAPHGV	0,723
B07	HLA-B*55:10	1089	FPREGVFV	0,5148
B07	HLA-B*55:10	1089	FPREGVFVS	0,776
B07	HLA-B*55:15	664	IPIGAGICA	0,566
B07	HLA-B*55:15	680	SPRRARSVA	0,6445
B07	HLA-B*55:15	714	IPTNFTISV	0,699
B07	HLA-B*55:15	1052	FPQSAPHGV	0,551
B07	HLA-B*55:15	1089	FPREGVFVS	0,6525
B07	HLA-B*55:17	462	KPFERDIST	0,5535
B07	HLA-B*55:17	664	IPIGAGICA	0,5355
B07	HLA-B*55:17	680	SPRRARSVA	0,6844
B07	HLA-B*55:17	714	IPTNFTISV	0,7936
B07	HLA-B*55:17	1052	FPQSAPHGV	0,6104
B07	HLA-B*55:17	1089	FPREGVFVS	0,5754
B07	HLA-B*55:19	38	YPDKVFRSS	0,6589
B07	HLA-B*55:19	38	YPDKVFRSSV	0,5249
B07	HLA-B*55:19	84	LPFNDGVYF	0,6389
B07	HLA-B*55:19	84	LPFNDGVYFA	0,6565
B07	HLA-B*55:19	336	CPFGEVFNA	0,5229
B07	HLA-B*55:19	462	KPFERDIST	0,6378
B07	HLA-B*55:19	506	QPYRVVVLS	0,527
B07	HLA-B*55:19	664	IPIGAGICA	0,7531
B07	HLA-B*55:19	714	IPTNFTISV	0,8516
B07	HLA-B*55:19	896	IPFAMQMAY	0,5572
B07	HLA-B*55:19	1052	FPQSAPHGV	0,758
B07	HLA-B*55:19	1052	FPQSAPHGVV	0,5174
B07	HLA-B*55:19	1089	FPREGVFV	0,5413
B07	HLA-B*55:19	1089	FPREGVFVS	0,8144
B07	HLA-B*56:01	38	YPDKVFRSS	0,5594
B07	HLA-B*56:01	84	LPFNDGVYF	0,6481
B07	HLA-B*56:01	84	LPFNDGVYFA	0,5533
B07	HLA-B*56:01	664	IPIGAGICA	0,6738
B07	HLA-B*56:01	714	IPTNFTISV	0,7754
B07	HLA-B*56:01	896	IPFAMQMAY	0,5446
B07	HLA-B*56:01	1052	FPQSAPHGV	0,6791
B07	HLA-B*56:01	1089	FPREGVFVS	0,7781
B07	HLA-B*56:03	84	LPFNDGVYF	0,65
B07	HLA-B*56:03	271	QPRTFLLKY	0,5652
B07	HLA-B*56:03	687	VASQSIIAY	0,5692
B07	HLA-B*56:03	896	IPFAMQMAY	0,7714
B07	HLA-B*56:05	664	IPIGAGICA	0,503
B07	HLA-B*56:05	680	SPRRARSVA	0,5529
B07	HLA-B*56:05	714	IPTNFTISV	0,7632
B07	HLA-B*56:05	896	IPFAMQMAY	0,5665
B07	HLA-B*56:05	1052	FPQSAPHGV	0,6122
B07	HLA-B*56:05	1089	FPREGVFV	0,5865
B07	HLA-B*56:05	1089	FPREGVFVS	0,6335
B07	HLA-B*56:13	38	YPDKVFRSS	0,5603

B07	HLA-B*56:13	38	YPDKVFRSSV	0,5158
B07	HLA-B*56:13	664	IPIGAGICA	0,6238
B07	HLA-B*56:13	680	SPRRARSVA	0,513
B07	HLA-B*56:13	714	IPTNFTISV	0,769
B07	HLA-B*56:13	1052	FPQSAPHGV	0,731
B07	HLA-B*56:13	1052	FPQSAPHGVV	0,5165
B07	HLA-B*56:13	1089	FPREGVFV	0,5099
B07	HLA-B*56:13	1089	FPREGVFVS	0,715
B07	HLA-B*56:15	84	LPFNDGVYF	0,7262
B07	HLA-B*56:15	664	IPIGAGICA	0,6282
B07	HLA-B*56:15	714	IPTNFTISV	0,8617
B07	HLA-B*56:15	1052	FPQSAPHGV	0,7753
B07	HLA-B*56:15	1052	FPQSAPHGVV	0,5072
B07	HLA-B*56:15	1089	FPREGVFVS	0,685
B07	HLA-B*56:16	38	YPDKVFRSS	0,5423
B07	HLA-B*56:16	84	LPFNDGVYF	0,6574
B07	HLA-B*56:16	664	IPIGAGICA	0,6319
B07	HLA-B*56:16	714	IPTNFTISV	0,7528
B07	HLA-B*56:16	896	IPFAMQMAY	0,5261
B07	HLA-B*56:16	1052	FPQSAPHGV	0,6463
B07	HLA-B*56:16	1089	FPREGVFVS	0,7472
B07	HLA-B*67:01	24	LPPAYTNSF	0,6526
B07	HLA-B*67:01	38	YPDKVFRSSV	0,5867
B07	HLA-B*67:01	38	YPDKVFRSSVL	0,5124
B07	HLA-B*67:01	84	LPFNDGVYF	0,8292
B07	HLA-B*67:01	208	TPINLVRDL	0,7949
B07	HLA-B*67:01	216	LPQGFSAL	0,5784
B07	HLA-B*67:01	229	LPIGINITRF	0,5824
B07	HLA-B*67:01	321	QPTESIVRF	0,6782
B07	HLA-B*67:01	506	QPYRVVVL	0,5066
B07	HLA-B*67:01	714	IPTNFTISV	0,8295
B07	HLA-B*67:01	861	LPPLLTDEM	0,5088
B07	HLA-B*67:01	896	IPFAMQMAY	0,5126
B07	HLA-B*67:01	1052	FPQSAPHGV	0,7937
B07	HLA-B*67:01	1052	FPQSAPHGVVF	0,6798
B07	HLA-B*67:01	1262	EPVLKGVKL	0,7213
B07	HLA-B*78:01	714	IPTNFTISV	0,7595
B07	HLA-B*78:01	1052	FPQSAPHGV	0,6988
B07	HLA-B*78:01	1089	FPREGVFV	0,6628
B07	HLA-B*78:01	1089	FPREGVFVS	0,5573
B07	HLA-B*78:02	714	IPTNFTISV	0,7684
B07	HLA-B*78:04	714	IPTNFTISV	0,7551
B07	HLA-B*78:04	1089	FPREGVFVS	0,5305
B08	HLA-B*08:01	17	NLTTRTQL	0,5899
B08	HLA-B*08:01	233	INITRFQTL	0,7017
B08	HLA-B*08:01	234	NITRFQTL	0,7511
B08	HLA-B*08:01	241	LLALHRSYL	0,5553
B08	HLA-B*08:01	269	YLQPRTFL	0,7406
B08	HLA-B*08:01	269	YLQPRTFLL	0,7867
B08	HLA-B*08:01	506	QPYRVVVL	0,766

B08	HLA-B*08:01	821	LLFNKVTL	0,6195
B08	HLA-B*08:01	996	LITGRLQSL	0,5644
B08	HLA-B*08:07	233	INITRFQTL	0,6349
B08	HLA-B*08:07	234	NITRFQTL	0,6414
B08	HLA-B*08:07	269	YLQPRTFL	0,6444
B08	HLA-B*08:07	269	YLQPRTFLL	0,7258
B08	HLA-B*08:07	506	QPYRVVVL	0,7156
B08	HLA-B*08:07	821	LLFNKVTL	0,5358
B08	HLA-B*08:07	996	LITGRLQSL	0,5316
B08	HLA-B*08:09	269	YLQPRTFLL	0,5745
B08	HLA-B*08:11	17	NLTTRTQL	0,5719
B08	HLA-B*08:11	233	INITRFQTL	0,6926
B08	HLA-B*08:11	234	NITRFQTL	0,7381
B08	HLA-B*08:11	241	LLALHRSYL	0,5524
B08	HLA-B*08:11	269	YLQPRTFL	0,7196
B08	HLA-B*08:11	269	YLQPRTFLL	0,7806
B08	HLA-B*08:11	506	QPYRVVVL	0,7665
B08	HLA-B*08:11	821	LLFNKVTL	0,5958
B08	HLA-B*08:11	996	LITGRLQSL	0,5708
B08	HLA-B*08:13	233	INITRFQTL	0,5821
B08	HLA-B*08:13	269	YLQPRTFL	0,5148
B08	HLA-B*08:13	269	YLQPRTFLL	0,6693
B08	HLA-B*08:13	506	QPYRVVVL	0,6559
B08	HLA-B*08:15	233	INITRFQTL	0,6284
B08	HLA-B*08:15	234	NITRFQTL	0,5714
B08	HLA-B*08:15	269	YLQPRTFL	0,6627
B08	HLA-B*08:15	269	YLQPRTFLL	0,7736
B08	HLA-B*08:15	821	LLFNKVTL	0,507
B08	HLA-B*08:18	17	NLTTRTQL	0,5899
B08	HLA-B*08:18	233	INITRFQTL	0,7017
B08	HLA-B*08:18	234	NITRFQTL	0,7511
B08	HLA-B*08:18	241	LLALHRSYL	0,5553
B08	HLA-B*08:18	269	YLQPRTFL	0,7406
B08	HLA-B*08:18	269	YLQPRTFLL	0,7867
B08	HLA-B*08:18	506	QPYRVVVL	0,766
B08	HLA-B*08:18	821	LLFNKVTL	0,6195
B08	HLA-B*08:18	996	LITGRLQSL	0,5644
B08	HLA-B*08:20	241	LLALHRSYL	0,5123
B08	HLA-B*08:20	269	YLQPRTFL	0,5485
B08	HLA-B*08:20	269	YLQPRTFLL	0,6503
B08	HLA-B*08:20	869	MIAQYTSAL	0,5115
B08	HLA-B*08:21	233	INITRFQTL	0,5468
B08	HLA-B*08:21	234	NITRFQTL	0,598
B08	HLA-B*08:21	269	YLQPRTFL	0,5663
B08	HLA-B*08:21	269	YLQPRTFLL	0,6617
B08	HLA-B*08:21	506	QPYRVVVL	0,6419
B08	HLA-B*08:22	17	NLTTRTQL	0,5899
B08	HLA-B*08:22	233	INITRFQTL	0,7017
B08	HLA-B*08:22	234	NITRFQTL	0,7511
B08	HLA-B*08:22	241	LLALHRSYL	0,5553

B08	HLA-B*08:22	269	YLQPRTFL	0,7406
B08	HLA-B*08:22	269	YLQPRTFLL	0,7867
B08	HLA-B*08:22	506	QPYRVVVL	0,766
B08	HLA-B*08:22	821	LLFNKVTL	0,6195
B08	HLA-B*08:22	996	LITGRLQSL	0,5644
B08	HLA-B*08:23	269	YLQPRTFL	0,5575
B08	HLA-B*08:23	269	YLQPRTFLL	0,6446
B08	HLA-B*08:23	821	LLFNKVTL	0,5015
B08	HLA-B*08:24	17	NLTTRTQL	0,5899
B08	HLA-B*08:24	233	INITRFQTL	0,7017
B08	HLA-B*08:24	234	NITRFQTL	0,7511
B08	HLA-B*08:24	241	LLALHRSYL	0,5553
B08	HLA-B*08:24	269	YLQPRTFL	0,7406
B08	HLA-B*08:24	269	YLQPRTFLL	0,7867
B08	HLA-B*08:24	506	QPYRVVVL	0,766
B08	HLA-B*08:24	821	LLFNKVTL	0,6195
B08	HLA-B*08:24	996	LITGRLQSL	0,5644
B08	HLA-B*08:25	269	YLQPRTFLL	0,5151
B27	HLA-B*14:01	236	TRFQTLLAL	0,8018
B27	HLA-B*14:01	453	YRLFRKSNL	0,6967
B27	HLA-B*14:02	236	TRFQTLLAL	0,8018
B27	HLA-B*14:02	453	YRLFRKSNL	0,6967
B27	HLA-B*14:03	236	TRFQTLLAL	0,6892
B27	HLA-B*14:03	453	YRLFRKSNL	0,576
B27	HLA-B*14:06	236	TRFQTLLAL	0,8675
B27	HLA-B*14:06	764	NRALTGIAV	0,7167
B27	HLA-B*15:03	20	TRTQLPPAY	0,7211
B27	HLA-B*15:03	22	TQLPPAYTNSF	0,6455
B27	HLA-B*15:03	35	GVYYPDKVF	0,6663
B27	HLA-B*15:03	47	VLHSTQDLF	0,5243
B27	HLA-B*15:03	77	KRFDNPVLPF	0,8296
B27	HLA-B*15:03	184	GNFKNLREF	0,6499
B27	HLA-B*15:03	186	FKNLREFVF	0,5195
B27	HLA-B*15:03	192	FVFKNIDGY	0,5282
B27	HLA-B*15:03	212	LVRDLPQGF	0,576
B27	HLA-B*15:03	236	TRFQTLLAL	0,608
B27	HLA-B*15:03	240	TLLALHRSY	0,6413
B27	HLA-B*15:03	261	GAAAYYVGY	0,5148
B27	HLA-B*15:03	267	VGYLQPRTF	0,643
B27	HLA-B*15:03	320	VQPTESIVRF	0,5258
B27	HLA-B*15:03	327	VRFPNITNL	0,8039
B27	HLA-B*15:03	339	GEVFNATRF	0,7209
B27	HLA-B*15:03	345	TRFASVYAW	0,6043
B27	HLA-B*15:03	366	SVLYNSASF	0,5032
B27	HLA-B*15:03	369	YNSASFSTF	0,7633
B27	HLA-B*15:03	408	RQIAPGQTG	0,5804
B27	HLA-B*15:03	413	GQTGKIADY	0,8037
B27	HLA-B*15:03	443	SKVGGNYNY	0,9112
B27	HLA-B*15:03	456	FRKSNLKPF	0,5235
B27	HLA-B*15:03	464	FERDISTEIY	0,635

B27	HLA-B*15:03	497	FQPTNGVGY	0,8242
B27	HLA-B*15:03	557	KKFLPFQQF	0,9568
B27	HLA-B*15:03	628	QLTPTWRVY	0,5065
B27	HLA-B*15:03	634	RVYSTGSNVF	0,7212
B27	HLA-B*15:03	687	VASQSIIAY	0,7907
B27	HLA-B*15:03	689	SQSIIAYTM	0,7655
B27	HLA-B*15:03	699	LGAENSVAY	0,5647
B27	HLA-B*15:03	710	NSIAIPTNF	0,5451
B27	HLA-B*15:03	789	YKTPPIKDF	0,8036
B27	HLA-B*15:03	794	IKDFGGFNF	0,7241
B27	HLA-B*15:03	815	RSFIEDLLF	0,624
B27	HLA-B*15:03	852	AQKFNGLTV	0,6121
B27	HLA-B*15:03	852	AQKFNGLTVL	0,5743
B27	HLA-B*15:03	853	QKFNGLTVL	0,7866
B27	HLA-B*15:03	880	GTITSGWTF	0,5063
B27	HLA-B*15:03	893	ALQIPFAMQM	0,5298
B27	HLA-B*15:03	894	LQIPFAMQM	0,9192
B27	HLA-B*15:03	919	NQKLIANQF	0,8596
B27	HLA-B*15:03	999	GRLQSLQTY	0,8486
B27	HLA-B*15:03	1044	GKGYHLMSF	0,7673
B27	HLA-B*15:03	1054	QSAPHGVVF	0,8355
B27	HLA-B*15:03	1087	AHFPREGVF	0,7921
B27	HLA-B*15:03	1113	QIITTDNTF	0,5046
B27	HLA-B*15:03	1130	IGIVNNTVY	0,5224
B27	HLA-B*15:03	1264	VLKGVKLHY	0,6224
B27	HLA-B*15:18	20	TRTQLPPAY	0,7269
B27	HLA-B*15:18	236	TRFQTLLAL	0,6174
B27	HLA-B*15:18	1087	AHFPREGVF	0,6545
B27	HLA-B*15:47	77	KRFDNPVLPF	0,5237
B27	HLA-B*15:47	339	GEVFNATRF	0,6636
B27	HLA-B*15:47	369	YNSASFSTF	0,5152
B27	HLA-B*15:47	413	GQTGKIADY	0,5603
B27	HLA-B*15:47	464	FERDISTEIY	0,5227
B27	HLA-B*15:47	557	KKFLPFQQF	0,7088
B27	HLA-B*15:47	687	VASQSIIAY	0,5883
B27	HLA-B*15:47	689	SQSIIAYTM	0,6903
B27	HLA-B*15:47	852	AQKFNGLTVL	0,503
B27	HLA-B*15:47	894	LQIPFAMQM	0,6781
B27	HLA-B*15:47	919	NQKLIANQF	0,6441
B27	HLA-B*15:47	1054	QSAPHGVVF	0,5973
B27	HLA-B*15:47	1087	AHFPREGVF	0,5178
B27	HLA-B*15:49	77	KRFDNPVLPF	0,5237
B27	HLA-B*15:49	339	GEVFNATRF	0,6636
B27	HLA-B*15:49	369	YNSASFSTF	0,5152
B27	HLA-B*15:49	413	GQTGKIADY	0,5603
B27	HLA-B*15:49	464	FERDISTEIY	0,5227
B27	HLA-B*15:49	557	KKFLPFQQF	0,7088
B27	HLA-B*15:49	687	VASQSIIAY	0,5883
B27	HLA-B*15:49	689	SQSIIAYTM	0,6903
B27	HLA-B*15:49	852	AQKFNGLTVL	0,503

B27	HLA-B*15:49	894	LQIPFAMQM	0,6781
B27	HLA-B*15:49	919	NQKLIANQF	0,6441
B27	HLA-B*15:49	1054	QSAPHGVVF	0,5973
B27	HLA-B*15:49	1087	AHFPREGVF	0,5178
B27	HLA-B*15:52	236	TRFQTLLAL	0,6881
B27	HLA-B*15:54	20	TRTQLPPAY	0,6536
B27	HLA-B*15:54	22	TQLPPAYTNSF	0,5407
B27	HLA-B*15:54	35	GVYYPDKVF	0,5415
B27	HLA-B*15:54	77	KRFDNPVLPF	0,707
B27	HLA-B*15:54	212	LVRDLPQGF	0,5164
B27	HLA-B*15:54	240	TLLALHRSY	0,5397
B27	HLA-B*15:54	320	VQPTESIVRF	0,5256
B27	HLA-B*15:54	339	GEVFNATRF	0,706
B27	HLA-B*15:54	369	YNSASFSTF	0,659
B27	HLA-B*15:54	408	RQIAPGQTG	0,5392
B27	HLA-B*15:54	413	GQTGKIADY	0,8083
B27	HLA-B*15:54	443	SKVGGNYNY	0,849
B27	HLA-B*15:54	456	FRKSNLKPF	0,5182
B27	HLA-B*15:54	464	FERDISTEIY	0,7035
B27	HLA-B*15:54	497	FQPTNGVGY	0,7908
B27	HLA-B*15:54	557	KKFLPFQQF	0,8533
B27	HLA-B*15:54	634	RVYSTGSNVF	0,6621
B27	HLA-B*15:54	687	VASQSIIAY	0,6625
B27	HLA-B*15:54	689	SQSIIAYTM	0,6728
B27	HLA-B*15:54	789	YKTPPIKDF	0,6416
B27	HLA-B*15:54	852	AQKFNGLTV	0,5786
B27	HLA-B*15:54	852	AQKFNGLTVL	0,5715
B27	HLA-B*15:54	853	QKFNGLTVL	0,5953
B27	HLA-B*15:54	894	LQIPFAMQM	0,7958
B27	HLA-B*15:54	919	NQKLIANQF	0,8585
B27	HLA-B*15:54	999	GRLQSLQTY	0,757
B27	HLA-B*15:54	1044	GKGYHLMSF	0,6503
B27	HLA-B*15:54	1054	QSAPHGVVF	0,7441
B27	HLA-B*15:54	1087	AHFPREGVF	0,6276
B27	HLA-B*15:54	1264	VLKGVKLHY	0,6417
B27	HLA-B*15:61	20	TRTQLPPAY	0,771
B27	HLA-B*15:61	35	GVYYPDKVF	0,5503
B27	HLA-B*15:61	77	KRFDNPVLPF	0,8652
B27	HLA-B*15:61	236	TRFQTLLAL	0,7603
B27	HLA-B*15:61	318	FRVQPTESI	0,5563
B27	HLA-B*15:61	327	VRFPNITNL	0,9057
B27	HLA-B*15:61	345	TRFASVYAW	0,6322
B27	HLA-B*15:61	369	YNSASFSTF	0,5292
B27	HLA-B*15:61	413	GQTGKIADY	0,6318
B27	HLA-B*15:61	443	SKVGGNYNY	0,8055
B27	HLA-B*15:61	456	FRKSNLKPF	0,5874
B27	HLA-B*15:61	497	FQPTNGVGY	0,6258
B27	HLA-B*15:61	557	KKFLPFQQF	0,895
B27	HLA-B*15:61	634	RVYSTGSNVF	0,5973
B27	HLA-B*15:61	687	VASQSIIAY	0,5972

B27	HLA-B*15:61	689	SQSIIAYTM	0,6401
B27	HLA-B*15:61	789	YKTPPIKDF	0,7085
B27	HLA-B*15:61	794	IKDFGGFNF	0,5487
B27	HLA-B*15:61	852	AQKFNGLTV	0,6105
B27	HLA-B*15:61	852	AQKFNGLTVL	0,5775
B27	HLA-B*15:61	853	QKFNGLTVL	0,7774
B27	HLA-B*15:61	894	LQIPFAMQM	0,7963
B27	HLA-B*15:61	919	NQKLIANQF	0,6902
B27	HLA-B*15:61	999	GRLQSLQTY	0,8572
B27	HLA-B*15:61	1038	KRVDFCGKGY	0,5634
B27	HLA-B*15:61	1044	GKGYHLMSF	0,636
B27	HLA-B*15:61	1054	QSAPHGVVF	0,6853
B27	HLA-B*15:61	1087	AHFPREGVF	0,6534
B27	HLA-B*15:61	1264	VLKGVKLHY	0,5156
B27	HLA-B*15:62	20	TRTQLPPAY	0,7053
B27	HLA-B*15:62	22	TQLPPAYTNSF	0,6112
B27	HLA-B*15:62	30	NSFTRGVYY	0,5218
B27	HLA-B*15:62	35	GVYYPDKVF	0,6492
B27	HLA-B*15:62	47	VLHSTQDLF	0,5047
B27	HLA-B*15:62	77	KRFDNPVLPF	0,7733
B27	HLA-B*15:62	184	GNFKNLREF	0,6307
B27	HLA-B*15:62	192	FVFKNIDGY	0,5448
B27	HLA-B*15:62	212	LVRDLPQGF	0,5648
B27	HLA-B*15:62	236	TRFQTLLAL	0,573
B27	HLA-B*15:62	240	TLLALHRSY	0,6832
B27	HLA-B*15:62	261	GAAAYYVGY	0,5324
B27	HLA-B*15:62	267	VGYLQPRTF	0,6274
B27	HLA-B*15:62	327	VRFPNITNL	0,7421
B27	HLA-B*15:62	339	GEVFNATRF	0,7077
B27	HLA-B*15:62	345	TRFASVYAW	0,577
B27	HLA-B*15:62	366	SVLYNSASF	0,5129
B27	HLA-B*15:62	369	YNSASFSTF	0,746
B27	HLA-B*15:62	408	RQIAPGQTG	0,5385
B27	HLA-B*15:62	413	GQTGKIADY	0,813
B27	HLA-B*15:62	443	SKVGGNYNY	0,9183
B27	HLA-B*15:62	464	FERDISTEIY	0,6286
B27	HLA-B*15:62	497	FQPTNGVGY	0,8232
B27	HLA-B*15:62	557	KKFLPFQQF	0,9461
B27	HLA-B*15:62	628	QLTPTWRVY	0,5393
B27	HLA-B*15:62	634	RVYSTGSNVF	0,7129
B27	HLA-B*15:62	687	VASQSIIAY	0,7985
B27	HLA-B*15:62	689	SQSIIAYTM	0,7527
B27	HLA-B*15:62	699	LGAENSVAY	0,5751
B27	HLA-B*15:62	710	NSIAIPTNF	0,5361
B27	HLA-B*15:62	789	YKTPPIKDF	0,7655
B27	HLA-B*15:62	794	IKDFGGFNF	0,6781
B27	HLA-B*15:62	815	RSFIEDLLF	0,5936
B27	HLA-B*15:62	852	AQKFNGLTV	0,582
B27	HLA-B*15:62	852	AQKFNGLTVL	0,5232
B27	HLA-B*15:62	853	QKFNGLTVL	0,7613

B27	HLA-B*15:62	893	ALQIPFAMQM	0,5135
B27	HLA-B*15:62	894	LQIPFAMQM	0,911
B27	HLA-B*15:62	896	IPFAMQMAY	0,5327
B27	HLA-B*15:62	919	NQKLIANQF	0,8564
B27	HLA-B*15:62	999	GRLQSLQTY	0,8305
B27	HLA-B*15:62	1044	GKGYHLMSF	0,7316
B27	HLA-B*15:62	1054	QSAPHGVVF	0,8256
B27	HLA-B*15:62	1087	AHFPREGVF	0,7587
B27	HLA-B*15:62	1113	QIITTDNTF	0,5006
B27	HLA-B*15:62	1130	IGIVNNTVY	0,5213
B27	HLA-B*15:62	1264	VLKGVKLHY	0,645
B27	HLA-B*15:69	20	TRTQLPPAY	0,7227
B27	HLA-B*15:69	22	TQLPPAYTNSF	0,5591
B27	HLA-B*15:69	35	GVYYPDKVF	0,5772
B27	HLA-B*15:69	77	KRFDNPVLPF	0,851
B27	HLA-B*15:69	184	GNFKNLREF	0,546
B27	HLA-B*15:69	236	TRFQTLLAL	0,6858
B27	HLA-B*15:69	240	TLLALHRSY	0,525
B27	HLA-B*15:69	267	VGYLQPRTF	0,5372
B27	HLA-B*15:69	327	VRFPNITNL	0,8345
B27	HLA-B*15:69	339	GEVFNATRF	0,7224
B27	HLA-B*15:69	345	TRFASVYAW	0,5818
B27	HLA-B*15:69	369	YNSASFSTF	0,6438
B27	HLA-B*15:69	408	RQIAPGQTG	0,5308
B27	HLA-B*15:69	413	GQTGKIADY	0,7354
B27	HLA-B*15:69	443	SKVGGNYNY	0,8229
B27	HLA-B*15:69	464	FERDISTEIY	0,6128
B27	HLA-B*15:69	497	FQPTNGVGY	0,674
B27	HLA-B*15:69	505	YQPYRVVVL	0,5216
B27	HLA-B*15:69	557	KKFLPFQQF	0,9341
B27	HLA-B*15:69	634	RVYSTGSNVF	0,6281
B27	HLA-B*15:69	687	VASQSIIAY	0,6873
B27	HLA-B*15:69	689	SQSIIAYTM	0,7524
B27	HLA-B*15:69	789	YKTPPIKDF	0,6877
B27	HLA-B*15:69	794	IKDFGGFNF	0,691
B27	HLA-B*15:69	815	RSFIEDLLF	0,5525
B27	HLA-B*15:69	852	AQKFNGLTV	0,584
B27	HLA-B*15:69	852	AQKFNGLTVL	0,6049
B27	HLA-B*15:69	853	QKFNGLTVL	0,7612
B27	HLA-B*15:69	893	ALQIPFAMQM	0,5311
B27	HLA-B*15:69	894	LQIPFAMQM	0,8912
B27	HLA-B*15:69	919	NQKLIANQF	0,8102
B27	HLA-B*15:69	999	GRLQSLQTY	0,8376
B27	HLA-B*15:69	1016	AEIRASANL	0,5219
B27	HLA-B*15:69	1038	KRVDFCGKGY	0,5143
B27	HLA-B*15:69	1044	GKGYHLMSF	0,7181
B27	HLA-B*15:69	1054	QSAPHGVVF	0,7141
B27	HLA-B*15:69	1087	AHFPREGVF	0,769
B27	HLA-B*15:69	1264	VLKGVKLHY	0,5553
B27	HLA-B*15:72	20	TRTQLPPAY	0,7269

B27	HLA-B*15:72	236	TRFQTLLAL	0,6174
B27	HLA-B*15:72	1087	AHFPREGVF	0,6545
B27	HLA-B*15:74	20	TRTQLPPAY	0,712
B27	HLA-B*15:74	22	TQLPPAYTNSF	0,6447
B27	HLA-B*15:74	30	NSFTRGVYY	0,5264
B27	HLA-B*15:74	35	GVYYPDKVF	0,6849
B27	HLA-B*15:74	47	VLHSTQDLF	0,519
B27	HLA-B*15:74	77	KRFDNPVLPF	0,8142
B27	HLA-B*15:74	84	LPFNDGVYF	0,5129
B27	HLA-B*15:74	184	GNFKNLREF	0,6596
B27	HLA-B*15:74	186	FKNLREFVF	0,5086
B27	HLA-B*15:74	192	FVFKNIDGY	0,5716
B27	HLA-B*15:74	212	LVRDLPQGF	0,5986
B27	HLA-B*15:74	236	TRFQTLLAL	0,6029
B27	HLA-B*15:74	240	TLLALHRSY	0,6632
B27	HLA-B*15:74	261	GAAAYYVGY	0,5496
B27	HLA-B*15:74	267	VGYLQPRTF	0,6398
B27	HLA-B*15:74	320	VQPTESIVRF	0,5264
B27	HLA-B*15:74	327	VRFPNITNL	0,7868
B27	HLA-B*15:74	339	GEVFNATRF	0,7102
B27	HLA-B*15:74	345	TRFASVYAW	0,583
B27	HLA-B*15:74	366	SVLYNSASF	0,5392
B27	HLA-B*15:74	369	YNSASFSTF	0,7528
B27	HLA-B*15:74	408	RQIAPGQTG	0,5683
B27	HLA-B*15:74	413	GQTGKIADY	0,7923
B27	HLA-B*15:74	443	SKVGGNYNY	0,9075
B27	HLA-B*15:74	456	FRKSNLKPF	0,5033
B27	HLA-B*15:74	464	FERDISTEIY	0,6276
B27	HLA-B*15:74	497	FQPTNGVGY	0,8159
B27	HLA-B*15:74	557	KKFLPFQQF	0,96
B27	HLA-B*15:74	628	QLTPTWRVY	0,5215
B27	HLA-B*15:74	634	RVYSTGSNVF	0,7445
B27	HLA-B*15:74	687	VASQSIIAY	0,7995
B27	HLA-B*15:74	689	SQSIIAYTM	0,7813
B27	HLA-B*15:74	699	LGAENSVAY	0,5653
B27	HLA-B*15:74	710	NSIAIPTNF	0,5635
B27	HLA-B*15:74	789	YKTPPIKDF	0,7759
B27	HLA-B*15:74	794	IKDFGGFNF	0,6901
B27	HLA-B*15:74	815	RSFIEDLLF	0,6421
B27	HLA-B*15:74	852	AQKFNGLTV	0,6162
B27	HLA-B*15:74	852	AQKFNGLTVL	0,5897
B27	HLA-B*15:74	853	QKFNGLTVL	0,772
B27	HLA-B*15:74	880	GTITSGWTF	0,5407
B27	HLA-B*15:74	893	ALQIPFAMQM	0,57
B27	HLA-B*15:74	894	LQIPFAMQM	0,9324
B27	HLA-B*15:74	896	IPFAMQMAY	0,5172
B27	HLA-B*15:74	919	NQKLIANQF	0,843
B27	HLA-B*15:74	999	GRLQSLQTY	0,8373
B27	HLA-B*15:74	1044	GKGYHLMSF	0,7534
B27	HLA-B*15:74	1054	QSAPHGVVF	0,8478

B27	HLA-B*15:74	1087	AHFPREGVF	0,7577
B27	HLA-B*15:74	1113	QIITTDNTF	0,5173
B27	HLA-B*15:74	1130	IGIVNNTVY	0,5267
B27	HLA-B*15:74	1264	VLKGVKLHY	0,6447
B27	HLA-B*15:80	20	TRTQLPPAY	0,7191
B27	HLA-B*15:80	236	TRFQTLLAL	0,5772
B27	HLA-B*15:80	1087	AHFPREGVF	0,5968
B27	HLA-B*15:91	153	MESEFRVY	0,6327
B27	HLA-B*15:91	297	SETKCTLKSF	0,5716
B27	HLA-B*15:91	339	GEVFNATRF	0,8899
B27	HLA-B*15:91	464	FERDISTEIY	0,7127
B27	HLA-B*15:91	653	AEHVNNSY	0,6902
B27	HLA-B*15:91	689	SQSIIAYTM	0,5235
B27	HLA-B*15:91	919	NQKLIANQF	0,6049
B27	HLA-B*15:91	1016	AEIRASANL	0,7495
B27	HLA-B*15:91	1201	QELGKYEQY	0,8926
B27	HLA-B*15:93	236	TRFQTLLAL	0,7254
B27	HLA-B*15:93	1087	AHFPREGVF	0,7077
B27	HLA-B*15:98	20	TRTQLPPAY	0,7211
B27	HLA-B*15:98	22	TQLPPAYTNSF	0,6455
B27	HLA-B*15:98	35	GVYYPDKVF	0,6663
B27	HLA-B*15:98	47	VLHSTQDLF	0,5243
B27	HLA-B*15:98	77	KRFDNPVLPF	0,8296
B27	HLA-B*15:98	184	GNFKNLREF	0,6499
B27	HLA-B*15:98	186	FKNLREFVF	0,5195
B27	HLA-B*15:98	192	FVFKNIDGY	0,5282
B27	HLA-B*15:98	212	LVRDLPQGF	0,576
B27	HLA-B*15:98	236	TRFQTLLAL	0,608
B27	HLA-B*15:98	240	TLLALHRSY	0,6413
B27	HLA-B*15:98	261	GAAAYYVGY	0,5148
B27	HLA-B*15:98	267	VGYLQPRTF	0,643
B27	HLA-B*15:98	320	VQPTESIVRF	0,5258
B27	HLA-B*15:98	327	VRFPNITNL	0,8039
B27	HLA-B*15:98	339	GEVFNATRF	0,7209
B27	HLA-B*15:98	345	TRFASVYAW	0,6043
B27	HLA-B*15:98	366	SVLYNSASF	0,5032
B27	HLA-B*15:98	369	YNSASFSTF	0,7633
B27	HLA-B*15:98	408	RQIAPGQTG	0,5804
B27	HLA-B*15:98	413	GQTGKIADY	0,8037
B27	HLA-B*15:98	443	SKVGGNYNY	0,9112
B27	HLA-B*15:98	456	FRKSNLKPF	0,5235
B27	HLA-B*15:98	464	FERDISTEIY	0,635
B27	HLA-B*15:98	497	FQPTNGVGY	0,8242
B27	HLA-B*15:98	557	KKFLPFQQF	0,9568
B27	HLA-B*15:98	628	QLTPTWRVY	0,5065
B27	HLA-B*15:98	634	RVYSTGSNVF	0,7212
B27	HLA-B*15:98	687	VASQSIIAY	0,7907
B27	HLA-B*15:98	689	SQSIIAYTM	0,7655
B27	HLA-B*15:98	699	LGAENSVAY	0,5647
B27	HLA-B*15:98	710	NSIAIPTNF	0,5451

B27	HLA-B*15:98	789	YKTPPIKDF	0,8036
B27	HLA-B*15:98	794	IKDFGGFNF	0,7241
B27	HLA-B*15:98	815	RSFIEDLLF	0,624
B27	HLA-B*15:98	852	AQKFNGLTV	0,6121
B27	HLA-B*15:98	852	AQKFNGLTVL	0,5743
B27	HLA-B*15:98	853	QKFNGLTVL	0,7866
B27	HLA-B*15:98	880	GTITSGWTF	0,5063
B27	HLA-B*15:98	893	ALQIPFAMQM	0,5298
B27	HLA-B*15:98	894	LQIPFAMQM	0,9192
B27	HLA-B*15:98	919	NQKLIANQF	0,8596
B27	HLA-B*15:98	999	GRLQSLQTY	0,8486
B27	HLA-B*15:98	1044	GKGYHLMSF	0,7673
B27	HLA-B*15:98	1054	QSAPHGVVF	0,8355
B27	HLA-B*15:98	1087	AHFPREGVF	0,7921
B27	HLA-B*15:98	1113	QIITTDNTF	0,5046
B27	HLA-B*15:98	1130	IGIVNNTVY	0,5224
B27	HLA-B*15:98	1264	VLKGVKLHY	0,6224
B27	HLA-B*27:02	77	KRFDNPVLPF	0,9936
B27	HLA-B*27:02	236	TRFQTLLAL	0,9672
B27	HLA-B*27:02	342	FNATRFASVYAW	0,5105
B27	HLA-B*27:02	343	NATRFASVYAW	0,7021
B27	HLA-B*27:02	344	ATRFASVYAW	0,9567
B27	HLA-B*27:02	345	TRFASVYAW	0,9908
B27	HLA-B*27:02	356	KRISNCVADY	0,7362
B27	HLA-B*27:02	814	KRSFIEDLLF	0,9772
B27	HLA-B*27:02	846	ARDLICAQKF	0,8438
B27	HLA-B*27:02	999	GRLQSLQTY	0,9922
B27	HLA-B*27:03	77	KRFDNPVLPF	0,9834
B27	HLA-B*27:03	814	KRSFIEDLLF	0,8897
B27	HLA-B*27:04	77	KRFDNPVLPF	0,986
B27	HLA-B*27:04	235	ITRFQTLLAL	0,5856
B27	HLA-B*27:04	236	TRFQTLLAL	0,9822
B27	HLA-B*27:04	327	VRFPNITNL	0,9799
B27	HLA-B*27:04	453	YRLFRKSNL	0,6317
B27	HLA-B*27:04	814	KRSFIEDLLF	0,8383
B27	HLA-B*27:04	904	YRFNGIGVT	0,6001
B27	HLA-B*27:04	999	GRLQSLQTY	0,967
B27	HLA-B*27:05	77	KRFDNPVLPF	0,9904
B27	HLA-B*27:05	101	IRGWIFGTTL	0,6849
B27	HLA-B*27:05	235	ITRFQTLLAL	0,5227
B27	HLA-B*27:05	236	TRFQTLLAL	0,9782
B27	HLA-B*27:05	318	FRVQPTESI	0,6722
B27	HLA-B*27:05	327	VRFPNITNL	0,9818
B27	HLA-B*27:05	344	ATRFASVYAW	0,7783
B27	HLA-B*27:05	345	TRFASVYAW	0,9414
B27	HLA-B*27:05	356	KRISNCVADY	0,6725
B27	HLA-B*27:05	453	YRLFRKSNL	0,6377
B27	HLA-B*27:05	814	KRSFIEDLL	0,8024
B27	HLA-B*27:05	814	KRSFIEDLLF	0,9257
B27	HLA-B*27:05	846	ARDLICAQKF	0,5398

B27	HLA-B*27:05	904	YRFNGIGVTQ	0,7685
B27	HLA-B*27:05	999	GRLQSLQTY	0,9825
B27	HLA-B*27:05	1038	KRVDFCGKGY	0,8924
B27	HLA-B*27:05	1038	KRVDFCGKGYHL	0,663
B27	HLA-B*27:06	77	KRFDNPVL	0,7543
B27	HLA-B*27:06	77	KRFDNPVLPF	0,9867
B27	HLA-B*27:06	101	IRGWIFGTTL	0,7577
B27	HLA-B*27:06	235	ITRFQTLLAL	0,6798
B27	HLA-B*27:06	236	TRFQTLLAL	0,9905
B27	HLA-B*27:06	318	FRVQPTESI	0,887
B27	HLA-B*27:06	325	SIVRFPNITNL	0,8466
B27	HLA-B*27:06	326	IVRFPNITNL	0,8762
B27	HLA-B*27:06	327	VRFPNITNL	0,9922
B27	HLA-B*27:06	453	YRLFRKSNL	0,7499
B27	HLA-B*27:06	684	ARSVASQSI	0,6368
B27	HLA-B*27:06	764	NRALTGIAV	0,588
B27	HLA-B*27:06	814	KRSFIEDLL	0,749
B27	HLA-B*27:06	814	KRSFIEDLLF	0,6968
B27	HLA-B*27:06	853	QKFNGLTVL	0,5745
B27	HLA-B*27:06	904	YRFNGIGV	0,617
B27	HLA-B*27:06	904	YRFNGIGVTQNV	0,5027
B27	HLA-B*27:06	1038	KRVDFCGKGYHL	0,6164
B27	HLA-B*27:06	1106	QRNFYEPQI	0,601
B27	HLA-B*27:07	77	KRFDNPVL	0,7135
B27	HLA-B*27:07	77	KRFDNPVLPF	0,9902
B27	HLA-B*27:07	101	IRGWIFGTTL	0,8223
B27	HLA-B*27:07	235	ITRFQTLLAL	0,6211
B27	HLA-B*27:07	236	TRFQTLLAL	0,9877
B27	HLA-B*27:07	318	FRVQPTESI	0,8291
B27	HLA-B*27:07	325	SIVRFPNITNL	0,8921
B27	HLA-B*27:07	326	IVRFPNITNL	0,8934
B27	HLA-B*27:07	327	VRFPNITNL	0,9922
B27	HLA-B*27:07	327	VRFPNITNLCPF	0,7232
B27	HLA-B*27:07	344	ATRFASVYAW	0,5458
B27	HLA-B*27:07	345	TRFASVYAW	0,8735
B27	HLA-B*27:07	453	YRLFRKSNL	0,6719
B27	HLA-B*27:07	557	KKFLPFQQF	0,6105
B27	HLA-B*27:07	684	ARSVASQSI	0,577
B27	HLA-B*27:07	764	NRALTGIAV	0,5708
B27	HLA-B*27:07	814	KRSFIEDLL	0,9203
B27	HLA-B*27:07	814	KRSFIEDLLF	0,8818
B27	HLA-B*27:07	904	YRFNGIGV	0,614
B27	HLA-B*27:07	904	YRFNGIGVTQNV	0,5884
B27	HLA-B*27:07	999	GRLQSLQTYV	0,526
B27	HLA-B*27:07	1038	KRVDFCGKGYHL	0,8228
B27	HLA-B*27:07	1106	QRNFYEPQI	0,7313
B27	HLA-B*27:09	77	KRFDNPVL	0,7057
B27	HLA-B*27:09	77	KRFDNPVLPF	0,9931
B27	HLA-B*27:09	101	IRGWIFGTTL	0,8159
B27	HLA-B*27:09	235	ITRFQTLLAL	0,6732

B27	HLA-B*27:09	236	TRFQTLLAL	0,9861
B27	HLA-B*27:09	318	FRVQPTESI	0,8298
B27	HLA-B*27:09	325	SIVRFPNITNL	0,8994
B27	HLA-B*27:09	326	IVRFPNITNL	0,9018
B27	HLA-B*27:09	327	VRFPNITNL	0,9903
B27	HLA-B*27:09	327	VRFPNITNLCPF	0,7706
B27	HLA-B*27:09	344	ATRFASVYAW	0,6784
B27	HLA-B*27:09	345	TRFASVYAW	0,904
B27	HLA-B*27:09	453	YRLFRKSNL	0,6207
B27	HLA-B*27:09	764	NRALTGIAV	0,51
B27	HLA-B*27:09	814	KRSFIEDLL	0,9093
B27	HLA-B*27:09	814	KRSFIEDLLF	0,925
B27	HLA-B*27:09	846	ARDLICAQKF	0,5004
B27	HLA-B*27:09	904	YRFNGIGV	0,6307
B27	HLA-B*27:09	904	YRFNGIGVT	0,5198
B27	HLA-B*27:09	904	YRFNGIGVTQNV	0,6738
B27	HLA-B*27:09	999	GRLQSLQTYV	0,5888
B27	HLA-B*27:09	1038	KRVDFCGKGYHL	0,8281
B27	HLA-B*27:09	1106	QRNFYEPQI	0,7026
B27	HLA-B*27:10	77	KRFDNPVLPF	0,9542
B27	HLA-B*27:10	236	TRFQTLLAL	0,9226
B27	HLA-B*27:10	453	YRLFRKSNL	0,5229
B27	HLA-B*27:13	77	KRFDNPVLPF	0,9904
B27	HLA-B*27:13	101	IRGWIFGTTL	0,6849
B27	HLA-B*27:13	235	ITRFQTLLAL	0,5227
B27	HLA-B*27:13	236	TRFQTLLAL	0,9782
B27	HLA-B*27:13	318	FRVQPTESI	0,6722
B27	HLA-B*27:13	327	VRFPNITNL	0,9818
B27	HLA-B*27:13	344	ATRFASVYAW	0,7783
B27	HLA-B*27:13	345	TRFASVYAW	0,9414
B27	HLA-B*27:13	356	KRISNCVADY	0,6725
B27	HLA-B*27:13	453	YRLFRKSNL	0,6377
B27	HLA-B*27:13	814	KRSFIEDLL	0,8024
B27	HLA-B*27:13	814	KRSFIEDLLF	0,9257
B27	HLA-B*27:13	846	ARDLICAQKF	0,5398
B27	HLA-B*27:13	904	YRFNGIGVTQ	0,7685
B27	HLA-B*27:13	999	GRLQSLQTY	0,9825
B27	HLA-B*27:13	1038	KRVDFCGKGY	0,8924
B27	HLA-B*27:13	1038	KRVDFCGKGYHL	0,663
B27	HLA-B*27:15	77	KRFDNPVLPF	0,9827
B27	HLA-B*27:15	235	ITRFQTLLAL	0,5206
B27	HLA-B*27:15	236	TRFQTLLAL	0,9763
B27	HLA-B*27:15	327	VRFPNITNL	0,9756
B27	HLA-B*27:15	453	YRLFRKSNL	0,6738
B27	HLA-B*27:15	814	KRSFIEDLLF	0,8178
B27	HLA-B*27:15	904	YRFNGIGVT	0,6068
B27	HLA-B*27:15	999	GRLQSLQTY	0,9613
B27	HLA-B*27:17	20	TRTQLPPAY	0,9
B27	HLA-B*27:17	77	KRFDNPVL	0,65
B27	HLA-B*27:17	77	KRFDNPVLPF	0,9915

B27	HLA-B*27:17	101	IRGWIFGTTL	0,7045
B27	HLA-B*27:17	235	ITRFQTLLAL	0,545
B27	HLA-B*27:17	236	TRFQTLLAL	0,9819
B27	HLA-B*27:17	318	FRVQPTESI	0,7209
B27	HLA-B*27:17	325	SIVRFPNITNL	0,8395
B27	HLA-B*27:17	326	IVRFPNITNL	0,8595
B27	HLA-B*27:17	327	VRFPNITNL	0,9862
B27	HLA-B*27:17	327	VRFPNITNLCPF	0,7682
B27	HLA-B*27:17	344	ATRFASVYAW	0,782
B27	HLA-B*27:17	345	TRFASVYAW	0,9514
B27	HLA-B*27:17	356	KRISNCVADY	0,6994
B27	HLA-B*27:17	453	YRLFRKSNL	0,6674
B27	HLA-B*27:17	508	YRVVVLSF	0,6075
B27	HLA-B*27:17	814	KRSFIEDLL	0,8281
B27	HLA-B*27:17	814	KRSFIEDLLF	0,9299
B27	HLA-B*27:17	846	ARDLICAQKF	0,5844
B27	HLA-B*27:17	904	YRFNGIGV	0,5341
B27	HLA-B*27:17	904	YRFNGIGVT	0,5417
B27	HLA-B*27:17	904	YRFNGIGVTQ	0,802
B27	HLA-B*27:17	998	TGRLQSLQTY	0,6318
B27	HLA-B*27:17	999	GRLQSLQTY	0,9859
B27	HLA-B*27:17	1038	KRVDFCGKGY	0,9087
B27	HLA-B*27:17	1038	KRVDFCGKGYHL	0,6876
B27	HLA-B*27:17	1106	QRNFYEPQI	0,5937
B27	HLA-B*27:25	77	KRFDNPVLPF	0,8627
B27	HLA-B*27:25	236	TRFQTLLAL	0,8437
B27	HLA-B*27:28	77	KRFDNPVLPF	0,9752
B27	HLA-B*27:28	101	IRGWIFGTTL	0,6664
B27	HLA-B*27:28	236	TRFQTLLAL	0,9591
B27	HLA-B*27:28	327	VRFPNITNL	0,961
B27	HLA-B*27:28	344	ATRFASVYAW	0,754
B27	HLA-B*27:28	345	TRFASVYAW	0,9368
B27	HLA-B*27:28	356	KRISNCVADY	0,6169
B27	HLA-B*27:28	453	YRLFRKSNL	0,6569
B27	HLA-B*27:28	508	YRVVVLSF	0,6768
B27	HLA-B*27:28	814	KRSFIEDLLF	0,8481
B27	HLA-B*27:28	846	ARDLICAQKF	0,5402
B27	HLA-B*27:28	904	YRFNGIGV	0,5641
B27	HLA-B*27:28	904	YRFNGIGVTQ	0,7159
B27	HLA-B*27:28	999	GRLQSLQTY	0,9569
B27	HLA-B*27:28	1038	KRVDFCGKGY	0,8473
B27	HLA-B*38:01	236	TRFQTLLAL	0,761
B27	HLA-B*38:05	236	TRFQTLLAL	0,761
B27	HLA-B*38:09	236	TRFQTLLAL	0,761
B27	HLA-B*38:11	236	TRFQTLLAL	0,761
B27	HLA-B*39:01	236	TRFQTLLAL	0,9488
B27	HLA-B*39:01	318	FRVQPTESI	0,8358
B27	HLA-B*39:01	505	YQPYRVVVL	0,5961
B27	HLA-B*39:01	576	VRDPQTLEI	0,7426
B27	HLA-B*39:01	689	SQSIIAYTM	0,557

B27	HLA-B*39:01	764	NRALTGIAV	0,8592
B27	HLA-B*39:01	853	QKFNGLTVL	0,7764
B27	HLA-B*39:01	1158	NHTSPDVDL	0,7288
B27	HLA-B*39:02	168	FEYVSQPFL	0,6813
B27	HLA-B*39:02	236	TRFQTLLAL	0,7521
B27	HLA-B*39:02	318	FRVQPTESI	0,588
B27	HLA-B*39:02	464	FERDISTEI	0,7304
B27	HLA-B*39:02	505	YQPYRVVVL	0,7112
B27	HLA-B*39:02	689	SQSIIAYTM	0,6777
B27	HLA-B*39:02	852	AQKFNGLTV	0,5032
B27	HLA-B*39:02	852	AQKFNGLTVL	0,6857
B27	HLA-B*39:02	853	QKFNGLTVL	0,8116
B27	HLA-B*39:02	894	LQIPFAMQM	0,6107
B27	HLA-B*39:02	1016	AEIRASANL	0,7178
B27	HLA-B*39:02	1181	KEIDRLNEV	0,7871
B27	HLA-B*39:04	236	TRFQTLLAL	0,9488
B27	HLA-B*39:04	318	FRVQPTESI	0,8358
B27	HLA-B*39:04	505	YQPYRVVVL	0,5961
B27	HLA-B*39:04	576	VRDPQTLEI	0,7426
B27	HLA-B*39:04	689	SQSIIAYTM	0,557
B27	HLA-B*39:04	764	NRALTGIAV	0,8592
B27	HLA-B*39:04	853	QKFNGLTVL	0,7764
B27	HLA-B*39:04	1158	NHTSPDVDL	0,7288
B27	HLA-B*39:07	20	TRTQLPPAY	0,7614
B27	HLA-B*39:07	236	TRFQTLLAL	0,7603
B27	HLA-B*39:07	345	TRFASVYAW	0,7796
B27	HLA-B*39:07	465	ERDISTEIY	0,6857
B27	HLA-B*39:07	689	SQSIIAYTM	0,5704
B27	HLA-B*39:07	794	IKDFGGFNF	0,5866
B27	HLA-B*39:07	853	QKFNGLTVL	0,5625
B27	HLA-B*39:07	894	LQIPFAMQM	0,5211
B27	HLA-B*39:07	1087	AHFPREGVF	0,6804
B27	HLA-B*39:09	48	LHSTQDLFL	0,5309
B27	HLA-B*39:09	236	TRFQTLLAL	0,9121
B27	HLA-B*39:09	269	YLQPRTFLL	0,5194
B27	HLA-B*39:09	318	FRVQPTESI	0,7813
B27	HLA-B*39:09	505	YQPYRVVVL	0,812
B27	HLA-B*39:09	689	SQSIIAYTM	0,6287
B27	HLA-B*39:09	764	NRALTGIAV	0,7776
B27	HLA-B*39:09	853	QKFNGLTVL	0,766
B27	HLA-B*39:09	1158	NHTSPDVDL	0,7704
B27	HLA-B*39:14	236	TRFQTLLAL	0,9724
B27	HLA-B*39:14	318	FRVQPTESI	0,8722
B27	HLA-B*39:14	327	VRFPNITNL	0,9443
B27	HLA-B*39:14	453	YRLFRKSNL	0,7017
B27	HLA-B*39:14	505	YQPYRVVVL	0,7017
B27	HLA-B*39:14	576	VRDPQTLEI	0,769
B27	HLA-B*39:14	689	SQSIIAYTM	0,5743
B27	HLA-B*39:14	764	NRALTGIAV	0,865
B27	HLA-B*39:14	853	QKFNGLTVL	0,8612

B27	HLA-B*39:14	1158	NHTSPDVDL	0,6977
B27	HLA-B*39:18	236	TRFQTLLAL	0,7919
B27	HLA-B*39:18	764	NRALTGIAV	0,7027
B27	HLA-B*39:23	168	FEYVSQPFL	0,6813
B27	HLA-B*39:23	236	TRFQTLLAL	0,7521
B27	HLA-B*39:23	318	FRVQPTESI	0,588
B27	HLA-B*39:23	464	FERDISTEI	0,7304
B27	HLA-B*39:23	505	YQPYRVVVL	0,7112
B27	HLA-B*39:23	689	SQSIIAYTM	0,6777
B27	HLA-B*39:23	852	AQKFNGLTV	0,5032
B27	HLA-B*39:23	852	AQKFNGLTVL	0,6857
B27	HLA-B*39:23	853	QKFNGLTVL	0,8116
B27	HLA-B*39:23	894	LQIPFAMQM	0,6107
B27	HLA-B*39:23	1016	AEIRASANL	0,7178
B27	HLA-B*39:23	1181	KEIDRLNEV	0,7871
B27	HLA-B*39:26	236	TRFQTLLAL	0,9488
B27	HLA-B*39:26	318	FRVQPTESI	0,8358
B27	HLA-B*39:26	505	YQPYRVVVL	0,5961
B27	HLA-B*39:26	576	VRDPQTLEI	0,7426
B27	HLA-B*39:26	689	SQSIIAYTM	0,557
B27	HLA-B*39:26	764	NRALTGIAV	0,8592
B27	HLA-B*39:26	853	QKFNGLTVL	0,7764
B27	HLA-B*39:26	1158	NHTSPDVDL	0,7288
B27	HLA-B*39:27	236	TRFQTLLAL	0,9298
B27	HLA-B*39:27	318	FRVQPTESI	0,7781
B27	HLA-B*39:27	327	VRFPNITNL	0,8349
B27	HLA-B*39:27	505	YQPYRVVVL	0,5535
B27	HLA-B*39:27	576	VRDPQTLEI	0,726
B27	HLA-B*39:27	764	NRALTGIAV	0,8242
B27	HLA-B*39:27	853	QKFNGLTVL	0,6621
B27	HLA-B*39:27	1158	NHTSPDVDL	0,5984
B27	HLA-B*39:29	236	TRFQTLLAL	0,9638
B27	HLA-B*39:29	318	FRVQPTESI	0,8376
B27	HLA-B*39:29	327	VRFPNITNL	0,9218
B27	HLA-B*39:29	453	YRLFRKSNL	0,6499
B27	HLA-B*39:29	505	YQPYRVVVL	0,5823
B27	HLA-B*39:29	764	NRALTGIAV	0,8145
B27	HLA-B*39:29	853	QKFNGLTVL	0,8131
B27	HLA-B*39:29	1158	NHTSPDVDL	0,5748
B27	HLA-B*39:30	236	TRFQTLLAL	0,8953
B27	HLA-B*39:30	318	FRVQPTESI	0,6956
B27	HLA-B*39:30	764	NRALTGIAV	0,7463
B27	HLA-B*39:30	853	QKFNGLTVL	0,6386
B27	HLA-B*39:30	1158	NHTSPDVDL	0,6332
B27	HLA-B*39:32	236	TRFQTLLAL	0,8554
B27	HLA-B*39:32	505	YQPYRVVVL	0,5179
B27	HLA-B*39:32	764	NRALTGIAV	0,7295
B27	HLA-B*39:32	1158	NHTSPDVDL	0,5368
B27	HLA-B*40:12	168	FEYVSQPFL	0,5354
B27	HLA-B*40:12	689	SQSIIAYTM	0,5826

B27	HLA-B*40:12	852	AQKFNGLTVL	0,6787
B27	HLA-B*48:01	852	AQKFNGLTVL	0,6838
B27	HLA-B*48:02	22	TQLPPAYTNSF	0,5241
B27	HLA-B*48:02	77	KRFDNPVLPF	0,5598
B27	HLA-B*48:02	339	GEVFNATRF	0,7713
B27	HLA-B*48:02	345	TRFASVYAW	0,5657
B27	HLA-B*48:02	369	YNSASFSTF	0,5533
B27	HLA-B*48:02	413	GQTGKIADY	0,6026
B27	HLA-B*48:02	443	SKVGGNYNY	0,8498
B27	HLA-B*48:02	464	FERDISTEIY	0,6629
B27	HLA-B*48:02	497	FQPTNGVGY	0,6828
B27	HLA-B*48:02	557	KKFLPFQQF	0,7916
B27	HLA-B*48:02	687	VASQSIIAY	0,6788
B27	HLA-B*48:02	689	SQSIIAYTM	0,6856
B27	HLA-B*48:02	789	YKTPPIKDF	0,5888
B27	HLA-B*48:02	794	IKDFGGFNF	0,6372
B27	HLA-B*48:02	853	QKFNGLTVL	0,5314
B27	HLA-B*48:02	894	LQIPFAMQM	0,7465
B27	HLA-B*48:02	919	NQKLIANQF	0,7202
B27	HLA-B*48:02	999	GRLQSLQTY	0,6453
B27	HLA-B*48:02	1054	QSAPHGVVF	0,5772
B27	HLA-B*48:02	1087	AHFPREGVF	0,5026
B27	HLA-B*48:02	1201	QELGKYEQY	0,6932
B27	HLA-B*48:03	168	FEYVSQPFL	0,5354
B27	HLA-B*48:03	689	SQSIIAYTM	0,5826
B27	HLA-B*48:03	852	AQKFNGLTVL	0,6787
B27	HLA-B*48:04	852	AQKFNGLTVL	0,6838
B27	HLA-B*48:05	852	AQKFNGLTV	0,5272
B27	HLA-B*48:05	852	AQKFNGLTVL	0,5881
B27	HLA-B*48:09	852	AQKFNGLTVL	0,6838
B27	HLA-B*48:10	168	FEYVSQPFL	0,5246
B27	HLA-B*48:10	852	AQKFNGLTVL	0,6418
B27	HLA-B*48:10	853	QKFNGLTVL	0,614
B27	HLA-B*48:10	894	LQIPFAMQM	0,5173
B27	HLA-B*48:12	168	FEYVSQPFL	0,5246
B27	HLA-B*48:12	852	AQKFNGLTVL	0,6418
B27	HLA-B*48:12	853	QKFNGLTVL	0,614
B27	HLA-B*48:12	894	LQIPFAMQM	0,5173
B27	HLA-B*48:13	168	FEYVSQPFL	0,5246
B27	HLA-B*48:13	852	AQKFNGLTVL	0,6418
B27	HLA-B*48:13	853	QKFNGLTVL	0,614
B27	HLA-B*48:13	894	LQIPFAMQM	0,5173
B27	HLA-B*73:01	236	TRFQTLLAL	0,8088
B27	HLA-B*73:01	764	NRALTGIAV	0,6815
B44	HLA-B*15:53	153	MESEFRVY	0,5616
B44	HLA-B*15:53	339	GEVFNATRF	0,8089
B44	HLA-B*15:53	369	YNSASFSTF	0,5654
B44	HLA-B*15:53	413	GQTGKIADY	0,7749
B44	HLA-B*15:53	443	SKVGGNYNY	0,7828
B44	HLA-B*15:53	464	FERDISTEI	0,5031

B44	HLA-B*15:53	464	FERDISTEIY	0,8541
B44	HLA-B*15:53	497	FQPTNGVGY	0,7516
B44	HLA-B*15:53	557	KKFLPFQQF	0,6864
B44	HLA-B*15:53	634	RVYSTGSNVF	0,5726
B44	HLA-B*15:53	653	AEHVNNSY	0,6845
B44	HLA-B*15:53	687	VASQSIIAY	0,6214
B44	HLA-B*15:53	689	SQSIIAYTM	0,6354
B44	HLA-B*15:53	852	AQKFNGLTV	0,5366
B44	HLA-B*15:53	852	AQKFNGLTVL	0,5324
B44	HLA-B*15:53	894	LQIPFAMQM	0,7568
B44	HLA-B*15:53	919	NQKLIANQF	0,8277
B44	HLA-B*15:53	1016	AEIRASANL	0,5785
B44	HLA-B*15:53	1054	QSAPHGVVF	0,6409
B44	HLA-B*15:53	1264	VLKGVKLHY	0,583
B44	HLA-B*18:01	153	MESEFRVY	0,8458
B44	HLA-B*18:01	168	FEYVSQPF	0,5811
B44	HLA-B*18:01	464	FERDISTEIY	0,7821
B44	HLA-B*18:01	724	TEILPVSM	0,6734
B44	HLA-B*18:01	1201	QELGKYEQY	0,8379
B44	HLA-B*18:03	153	MESEFRVY	0,5628
B44	HLA-B*18:03	464	FERDISTEI	0,5105
B44	HLA-B*18:03	464	FERDISTEIY	0,5603
B44	HLA-B*18:05	153	MESEFRVY	0,8458
B44	HLA-B*18:05	168	FEYVSQPF	0,5811
B44	HLA-B*18:05	464	FERDISTEIY	0,7821
B44	HLA-B*18:05	724	TEILPVSM	0,6734
B44	HLA-B*18:05	1201	QELGKYEQY	0,8379
B44	HLA-B*18:06	153	MESEFRVY	0,6821
B44	HLA-B*18:06	464	FERDISTEIY	0,5613
B44	HLA-B*18:10	153	MESEFRVY	0,6833
B44	HLA-B*18:10	339	GEVFNATRF	0,6957
B44	HLA-B*18:10	464	FERDISTEIY	0,7407
B44	HLA-B*18:10	724	TEILPVSM	0,6043
B44	HLA-B*18:10	1201	QELGKYEQY	0,7825
B44	HLA-B*18:11	153	MESEFRVY	0,7741
B44	HLA-B*18:11	339	GEVFNATRF	0,7018
B44	HLA-B*18:11	464	FERDISTEI	0,5685
B44	HLA-B*18:11	464	FERDISTEIY	0,799
B44	HLA-B*18:11	724	TEILPVSM	0,6215
B44	HLA-B*18:11	1201	QELGKYEQY	0,834
B44	HLA-B*18:13	153	MESEFRVY	0,8429
B44	HLA-B*18:13	168	FEYVSQPF	0,5593
B44	HLA-B*18:13	388	NDLCFTNVY	0,5383
B44	HLA-B*18:13	464	FERDISTEIY	0,7752
B44	HLA-B*18:13	1201	QELGKYEQY	0,7889
B44	HLA-B*18:13	1206	YEQYIKWPW	0,5226
B44	HLA-B*18:15	153	MESEFRVY	0,7703
B44	HLA-B*18:15	168	FEYVSQPF	0,5051
B44	HLA-B*18:15	464	FERDISTEIY	0,7513
B44	HLA-B*18:15	896	IPFAMQMAY	0,5009

B44	HLA-B*18:19	153	MESEFRVY	0,6571
B44	HLA-B*18:19	464	FERDISTEIY	0,725
B44	HLA-B*18:20	153	MESEFRVY	0,8458
B44	HLA-B*18:20	168	FEYVSQPF	0,5811
B44	HLA-B*18:20	464	FERDISTEIY	0,7821
B44	HLA-B*18:20	724	TEILPVSM	0,6734
B44	HLA-B*18:20	1201	QELGKYEQY	0,8379
B44	HLA-B*37:01	168	FEYVSQPFL	0,6847
B44	HLA-B*37:01	1181	KEIDRLNEV	0,8589
B44	HLA-B*37:04	168	FEYVSQPFL	0,6712
B44	HLA-B*40:01	168	FEYVSQPFL	0,9485
B44	HLA-B*40:01	168	FEYVSQPFLM	0,5398
B44	HLA-B*40:01	339	GEVFNATRF	0,8888
B44	HLA-B*40:01	464	FERDISTEI	0,9094
B44	HLA-B*40:01	660	YECDIPIGAGI	0,5931
B44	HLA-B*40:01	818	IEDLLFNKV	0,639
B44	HLA-B*40:01	987	VEAEVQIDRL	0,8709
B44	HLA-B*40:01	989	AEVQIDRLI	0,9071
B44	HLA-B*40:01	1016	AEIRASANL	0,9748
B44	HLA-B*40:01	1181	KEIDRLNEV	0,906
B44	HLA-B*40:01	1194	NESLIDLQEL	0,5405
B44	HLA-B*40:01	1256	FDEDDSEPVL	0,5944
B44	HLA-B*40:01	1261	SEPVLKGVKL	0,736
B44	HLA-B*40:02	155	SEFRVYSSA	0,652
B44	HLA-B*40:02	168	FEYVSQPFL	0,8998
B44	HLA-B*40:02	190	REFVFKNI	0,6104
B44	HLA-B*40:02	339	GEVFNATRF	0,776
B44	HLA-B*40:02	464	FERDISTEI	0,8995
B44	HLA-B*40:02	660	YECDIPIGA	0,512
B44	HLA-B*40:02	724	TEILPVSM	0,8512
B44	HLA-B*40:02	818	IEDLLFNKV	0,6624
B44	HLA-B*40:02	987	VEAEVQIDRL	0,7072
B44	HLA-B*40:02	989	AEVQIDRL	0,7511
B44	HLA-B*40:02	989	AEVQIDRLI	0,8417
B44	HLA-B*40:02	1016	AEIRASANL	0,9365
B44	HLA-B*40:02	1181	KEIDRLNEV	0,9443
B44	HLA-B*40:02	1261	SEPVLKGVKL	0,654
B44	HLA-B*40:05	155	SEFRVYSSA	0,5629
B44	HLA-B*40:05	168	FEYVSQPFL	0,7104
B44	HLA-B*40:05	464	FERDISTEI	0,8023
B44	HLA-B*40:05	1016	AEIRASANL	0,8349
B44	HLA-B*40:05	1181	KEIDRLNEV	0,879
B44	HLA-B*40:06	155	SEFRVYSSA	0,7601
B44	HLA-B*40:06	168	FEYVSQPFL	0,7587
B44	HLA-B*40:06	464	FERDISTEI	0,7272
B44	HLA-B*40:06	618	TEVPVAIHA	0,7591
B44	HLA-B*40:06	660	YECDIPIGA	0,6484
B44	HLA-B*40:06	818	IEDLLFNKV	0,6059
B44	HLA-B*40:06	1016	AEIRASANL	0,6441
B44	HLA-B*40:06	1181	KEIDRLNEV	0,765

B44	HLA-B*40:11	168	FEYVSQPFL	0,905
B44	HLA-B*40:11	339	GEVFNATRF	0,8092
B44	HLA-B*40:11	464	FERDISTEI	0,8897
B44	HLA-B*40:11	618	TEVPVAIHA	0,6448
B44	HLA-B*40:11	724	TEILPVSM	0,7985
B44	HLA-B*40:11	818	IEDLLFNKV	0,6927
B44	HLA-B*40:11	987	VEAEVQIDRL	0,7437
B44	HLA-B*40:11	989	AEVQIDRL	0,7049
B44	HLA-B*40:11	989	AEVQIDRLI	0,8855
B44	HLA-B*40:11	1016	AEIRASANL	0,9391
B44	HLA-B*40:11	1181	KEIDRLNEV	0,9057
B44	HLA-B*40:11	1256	FDEDDSEPVL	0,5382
B44	HLA-B*40:11	1261	SEPVLKGVKL	0,6019
B44	HLA-B*40:14	168	FEYVSQPFL	0,905
B44	HLA-B*40:14	339	GEVFNATRF	0,8092
B44	HLA-B*40:14	464	FERDISTEI	0,8897
B44	HLA-B*40:14	618	TEVPVAIHA	0,6448
B44	HLA-B*40:14	724	TEILPVSM	0,7985
B44	HLA-B*40:14	818	IEDLLFNKV	0,6927
B44	HLA-B*40:14	987	VEAEVQIDRL	0,7437
B44	HLA-B*40:14	989	AEVQIDRL	0,7049
B44	HLA-B*40:14	989	AEVQIDRLI	0,8855
B44	HLA-B*40:14	1016	AEIRASANL	0,9391
B44	HLA-B*40:14	1181	KEIDRLNEV	0,9057
B44	HLA-B*40:14	1256	FDEDDSEPVL	0,5382
B44	HLA-B*40:14	1261	SEPVLKGVKL	0,6019
B44	HLA-B*40:15	155	SEFRVYSSA	0,5585
B44	HLA-B*40:15	168	FEYVSQPFL	0,7239
B44	HLA-B*40:15	214	RDLPQGFSAL	0,5122
B44	HLA-B*40:15	464	FERDISTEI	0,8506
B44	HLA-B*40:15	1016	AEIRASANL	0,8988
B44	HLA-B*40:15	1181	KEIDRLNEV	0,8892
B44	HLA-B*40:16	155	SEFRVYSSA	0,5585
B44	HLA-B*40:16	168	FEYVSQPFL	0,7239
B44	HLA-B*40:16	214	RDLPQGFSAL	0,5122
B44	HLA-B*40:16	464	FERDISTEI	0,8506
B44	HLA-B*40:16	1016	AEIRASANL	0,8988
B44	HLA-B*40:16	1181	KEIDRLNEV	0,8892
B44	HLA-B*40:20	153	MESEFRVY	0,5689
B44	HLA-B*40:20	168	FEYVSQPFL	0,55
B44	HLA-B*40:20	190	REFVFKNIDGY	0,5832
B44	HLA-B*40:20	339	GEVFNATRF	0,906
B44	HLA-B*40:20	464	FERDISTEI	0,6148
B44	HLA-B*40:20	464	FERDISTEIY	0,806
B44	HLA-B*40:20	653	AEHVNNSY	0,7263
B44	HLA-B*40:20	724	TEILPVSM	0,5311
B44	HLA-B*40:20	989	AEVQIDRLI	0,6133
B44	HLA-B*40:20	1016	AEIRASANL	0,7146
B44	HLA-B*40:20	1181	KEIDRLNEV	0,6384
B44	HLA-B*40:20	1201	QELGKYEQY	0,8567

B44	HLA-B*40:26	155	SEFRVYSSA	0,5119
B44	HLA-B*40:26	168	FEYVSQPFL	0,7736
B44	HLA-B*40:26	339	GEVFNATRF	0,7063
B44	HLA-B*40:26	464	FERDISTEI	0,8764
B44	HLA-B*40:26	724	TEILPVSM	0,6548
B44	HLA-B*40:26	989	AEVQIDRLI	0,7297
B44	HLA-B*40:26	1016	AEIRASANL	0,9027
B44	HLA-B*40:26	1181	KEIDRLNEV	0,9114
B44	HLA-B*40:29	155	SEFRVYSSA	0,6037
B44	HLA-B*40:29	168	FEYVSQPFL	0,838
B44	HLA-B*40:29	190	REFVFKNI	0,5309
B44	HLA-B*40:29	339	GEVFNATRF	0,7589
B44	HLA-B*40:29	464	FERDISTEI	0,8407
B44	HLA-B*40:29	724	TEILPVSM	0,764
B44	HLA-B*40:29	818	IEDLLFNKV	0,5439
B44	HLA-B*40:29	987	VEAEVQIDRL	0,6192
B44	HLA-B*40:29	989	AEVQIDRL	0,6685
B44	HLA-B*40:29	989	AEVQIDRLI	0,7966
B44	HLA-B*40:29	1016	AEIRASANL	0,9162
B44	HLA-B*40:29	1181	KEIDRLNEV	0,9308
B44	HLA-B*40:29	1261	SEPVLKGVKL	0,5302
B44	HLA-B*40:35	155	SEFRVYSSA	0,652
B44	HLA-B*40:35	168	FEYVSQPFL	0,8998
B44	HLA-B*40:35	190	REFVFKNI	0,6104
B44	HLA-B*40:35	339	GEVFNATRF	0,776
B44	HLA-B*40:35	464	FERDISTEI	0,8995
B44	HLA-B*40:35	660	YECDIPIGA	0,512
B44	HLA-B*40:35	724	TEILPVSM	0,8512
B44	HLA-B*40:35	818	IEDLLFNKV	0,6624
B44	HLA-B*40:35	987	VEAEVQIDRL	0,7072
B44	HLA-B*40:35	989	AEVQIDRL	0,7511
B44	HLA-B*40:35	989	AEVQIDRLI	0,8417
B44	HLA-B*40:35	1016	AEIRASANL	0,9365
B44	HLA-B*40:35	1181	KEIDRLNEV	0,9443
B44	HLA-B*40:35	1261	SEPVLKGVKL	0,654
B44	HLA-B*40:39	155	SEFRVYSSA	0,667
B44	HLA-B*40:39	168	FEYVSQPFL	0,8825
B44	HLA-B*40:39	190	REFVFKNI	0,6464
B44	HLA-B*40:39	339	GEVFNATRF	0,7646
B44	HLA-B*40:39	464	FERDISTEI	0,911
B44	HLA-B*40:39	660	YECDIPIGA	0,5358
B44	HLA-B*40:39	724	TEILPVSM	0,8562
B44	HLA-B*40:39	818	IEDLLFNKV	0,6636
B44	HLA-B*40:39	987	VEAEVQIDRL	0,6426
B44	HLA-B*40:39	989	AEVQIDRL	0,757
B44	HLA-B*40:39	989	AEVQIDRLI	0,8286
B44	HLA-B*40:39	1016	AEIRASANL	0,9217
B44	HLA-B*40:39	1181	KEIDRLNEV	0,944
B44	HLA-B*40:39	1261	SEPVLKGVKL	0,621
B44	HLA-B*40:40	168	FEYVSQPFL	0,7627

B44	HLA-B*40:40	464	FERDISTEI	0,7123
B44	HLA-B*40:40	852	AQKFNGLTVL	0,5879
B44	HLA-B*40:40	1016	AEIRASANL	0,7906
B44	HLA-B*40:40	1181	KEIDRLNEV	0,8178
B44	HLA-B*40:49	168	FEYVSQPFL	0,7985
B44	HLA-B*40:49	689	SQSIIAYTM	0,587
B44	HLA-B*40:49	852	AQKFNGLTVL	0,6038
B44	HLA-B*40:49	1016	AEIRASANL	0,8824
B44	HLA-B*40:50	168	FEYVSQPFL	0,7931
B44	HLA-B*40:50	464	FERDISTEI	0,8322
B44	HLA-B*40:50	724	TEILPVSM	0,5961
B44	HLA-B*40:50	989	AEVQIDRLI	0,69
B44	HLA-B*40:50	1016	AEIRASANL	0,8418
B44	HLA-B*40:50	1181	KEIDRLNEV	0,9058
B44	HLA-B*40:53	155	SEFRVYSSA	0,7601
B44	HLA-B*40:53	168	FEYVSQPFL	0,7587
B44	HLA-B*40:53	464	FERDISTEI	0,7272
B44	HLA-B*40:53	618	TEVPVAIHA	0,7591
B44	HLA-B*40:53	660	YECDIPIGA	0,6484
B44	HLA-B*40:53	818	IEDLLFNKV	0,6059
B44	HLA-B*40:53	1016	AEIRASANL	0,6441
B44	HLA-B*40:53	1181	KEIDRLNEV	0,765
B44	HLA-B*40:54	168	FEYVSQPFL	0,9485
B44	HLA-B*40:54	168	FEYVSQPFLM	0,5398
B44	HLA-B*40:54	339	GEVFNATRF	0,8888
B44	HLA-B*40:54	464	FERDISTEI	0,9094
B44	HLA-B*40:54	660	YECDIPIGAGI	0,5931
B44	HLA-B*40:54	818	IEDLLFNKV	0,639
B44	HLA-B*40:54	987	VEAEVQIDRL	0,8709
B44	HLA-B*40:54	989	AEVQIDRLI	0,9071
B44	HLA-B*40:54	1016	AEIRASANL	0,9748
B44	HLA-B*40:54	1181	KEIDRLNEV	0,906
B44	HLA-B*40:54	1194	NESLIDLQEL	0,5405
B44	HLA-B*40:54	1256	FDEDDSEPVL	0,5944
B44	HLA-B*40:54	1261	SEPVLKGVKL	0,736
B44	HLA-B*40:55	168	FEYVSQPFL	0,9485
B44	HLA-B*40:55	168	FEYVSQPFLM	0,5398
B44	HLA-B*40:55	339	GEVFNATRF	0,8888
B44	HLA-B*40:55	464	FERDISTEI	0,9094
B44	HLA-B*40:55	660	YECDIPIGAGI	0,5931
B44	HLA-B*40:55	818	IEDLLFNKV	0,639
B44	HLA-B*40:55	987	VEAEVQIDRL	0,8709
B44	HLA-B*40:55	989	AEVQIDRLI	0,9071
B44	HLA-B*40:55	1016	AEIRASANL	0,9748
B44	HLA-B*40:55	1181	KEIDRLNEV	0,906
B44	HLA-B*40:55	1194	NESLIDLQEL	0,5405
B44	HLA-B*40:55	1256	FDEDDSEPVL	0,5944
B44	HLA-B*40:55	1261	SEPVLKGVKL	0,736
B44	HLA-B*40:56	155	SEFRVYSSA	0,652
B44	HLA-B*40:56	168	FEYVSQPFL	0,8998

B44	HLA-B*40:56	190	REFVFKNI	0,6104
B44	HLA-B*40:56	339	GEVFNATRF	0,776
B44	HLA-B*40:56	464	FERDISTEI	0,8995
B44	HLA-B*40:56	660	YECDIPIGA	0,512
B44	HLA-B*40:56	724	TEILPVSM	0,8512
B44	HLA-B*40:56	818	IEDLLFNKV	0,6624
B44	HLA-B*40:56	987	VEAEVQIDRL	0,7072
B44	HLA-B*40:56	989	AEVQIDRL	0,7511
B44	HLA-B*40:56	989	AEVQIDRLI	0,8417
B44	HLA-B*40:56	1016	AEIRASANL	0,9365
B44	HLA-B*40:56	1181	KEIDRLNEV	0,9443
B44	HLA-B*40:56	1261	SEPVLKGVKL	0,654
B44	HLA-B*40:57	155	SEFRVYSSA	0,652
B44	HLA-B*40:57	168	FEYVSQPFL	0,8998
B44	HLA-B*40:57	190	REFVFKNI	0,6104
B44	HLA-B*40:57	339	GEVFNATRF	0,776
B44	HLA-B*40:57	464	FERDISTEI	0,8995
B44	HLA-B*40:57	660	YECDIPIGA	0,512
B44	HLA-B*40:57	724	TEILPVSM	0,8512
B44	HLA-B*40:57	818	IEDLLFNKV	0,6624
B44	HLA-B*40:57	987	VEAEVQIDRL	0,7072
B44	HLA-B*40:57	989	AEVQIDRL	0,7511
B44	HLA-B*40:57	989	AEVQIDRLI	0,8417
B44	HLA-B*40:57	1016	AEIRASANL	0,9365
B44	HLA-B*40:57	1181	KEIDRLNEV	0,9443
B44	HLA-B*40:57	1261	SEPVLKGVKL	0,654
B44	HLA-B*41:02	155	SEFRVYSSA	0,6407
B44	HLA-B*41:02	168	FEYVSQPFL	0,8406
B44	HLA-B*41:02	190	REFVFKNI	0,6992
B44	HLA-B*41:02	339	GEVFNATRF	0,7419
B44	HLA-B*41:02	464	FERDISTEI	0,9149
B44	HLA-B*41:02	724	TEILPVSM	0,8338
B44	HLA-B*41:02	818	IEDLLFNKV	0,7103
B44	HLA-B*41:02	987	VEAEVQIDRL	0,6717
B44	HLA-B*41:02	989	AEVQIDRL	0,7643
B44	HLA-B*41:02	989	AEVQIDRLI	0,847
B44	HLA-B*41:02	1016	AEIRASANL	0,9273
B44	HLA-B*41:02	1181	KEIDRLNEV	0,9555
B44	HLA-B*41:02	1261	SEPVLKGVKL	0,7634
B44	HLA-B*41:03	168	FEYVSQPFL	0,8113
B44	HLA-B*41:03	339	GEVFNATRF	0,7437
B44	HLA-B*41:03	464	FERDISTEI	0,8985
B44	HLA-B*41:03	724	TEILPVSM	0,7399
B44	HLA-B*41:03	818	IEDLLFNKV	0,706
B44	HLA-B*41:03	987	VEAEVQIDRL	0,6522
B44	HLA-B*41:03	989	AEVQIDRL	0,6693
B44	HLA-B*41:03	989	AEVQIDRLI	0,8767
B44	HLA-B*41:03	1016	AEIRASANL	0,9094
B44	HLA-B*41:03	1181	KEIDRLNEV	0,9207
B44	HLA-B*41:03	1261	SEPVLKGVKL	0,6936

B44	HLA-B*44:02	95	TEKSNIIRGW	0,9813
B44	HLA-B*44:02	153	MESEFRVY	0,5131
B44	HLA-B*44:02	297	SETKCTLKSF	0,7692
B44	HLA-B*44:02	339	GEVFNATRF	0,8968
B44	HLA-B*44:02	779	QEVFAQVKQIY	0,7113
B44	HLA-B*44:02	989	AEVQIDRLI	0,9059
B44	HLA-B*44:02	1016	AEIRASANL	0,8002
B44	HLA-B*44:02	1091	REGVFVSNGTHW	0,792
B44	HLA-B*44:02	1201	QELGKYEQY	0,9415
B44	HLA-B*44:02	1201	QELGKYEQYIKW	0,7097
B44	HLA-B*44:02	1206	YEQYIKWPW	0,7519
B44	HLA-B*44:03	95	TEKSNIIRGW	0,9553
B44	HLA-B*44:03	153	MESEFRVY	0,5502
B44	HLA-B*44:03	297	SETKCTLKSF	0,7221
B44	HLA-B*44:03	339	GEVFNATRF	0,9332
B44	HLA-B*44:03	747	TECSNLLLQY	0,6025
B44	HLA-B*44:03	779	QEVFAQVKQIY	0,7054
B44	HLA-B*44:03	989	AEVQIDRLI	0,922
B44	HLA-B*44:03	1016	AEIRASANL	0,8443
B44	HLA-B*44:03	1091	REGVFVSNGTHW	0,7134
B44	HLA-B*44:03	1201	QELGKYEQY	0,9687
B44	HLA-B*44:03	1201	QELGKYEQYIKW	0,6195
B44	HLA-B*44:03	1206	YEQYIKWPW	0,671
B44	HLA-B*44:04	95	TEKSNIIRGW	0,9072
B44	HLA-B*44:04	297	SETKCTLKSF	0,6094
B44	HLA-B*44:04	339	GEVFNATRF	0,8061
B44	HLA-B*44:04	747	TECSNLLLQY	0,5668
B44	HLA-B*44:04	989	AEVQIDRLI	0,7773
B44	HLA-B*44:04	1016	AEIRASANL	0,6706
B44	HLA-B*44:04	1091	REGVFVSNGTHW	0,6043
B44	HLA-B*44:04	1201	QELGKYEQY	0,8836
B44	HLA-B*44:04	1206	YEQYIKWPW	0,6802
B44	HLA-B*44:07	95	TEKSNIIRGW	0,9553
B44	HLA-B*44:07	153	MESEFRVY	0,5502
B44	HLA-B*44:07	297	SETKCTLKSF	0,7221
B44	HLA-B*44:07	339	GEVFNATRF	0,9332
B44	HLA-B*44:07	747	TECSNLLLQY	0,6025
B44	HLA-B*44:07	779	QEVFAQVKQIY	0,7054
B44	HLA-B*44:07	989	AEVQIDRLI	0,922
B44	HLA-B*44:07	1016	AEIRASANL	0,8443
B44	HLA-B*44:07	1091	REGVFVSNGTHW	0,7134
B44	HLA-B*44:07	1201	QELGKYEQY	0,9687
B44	HLA-B*44:07	1201	QELGKYEQYIKW	0,6195
B44	HLA-B*44:07	1206	YEQYIKWPW	0,671
B44	HLA-B*44:13	95	TEKSNIIRGW	0,9553
B44	HLA-B*44:13	153	MESEFRVY	0,5502
B44	HLA-B*44:13	297	SETKCTLKSF	0,7221
B44	HLA-B*44:13	339	GEVFNATRF	0,9332
B44	HLA-B*44:13	747	TECSNLLLQY	0,6025
B44	HLA-B*44:13	779	QEVFAQVKQIY	0,7054

B44	HLA-B*44:13	989	AEVQIDRLI	0,922
B44	HLA-B*44:13	1016	AEIRASANL	0,8443
B44	HLA-B*44:13	1091	REGVFVSNGTHW	0,7134
B44	HLA-B*44:13	1201	QELGKYEQY	0,9687
B44	HLA-B*44:13	1201	QELGKYEQYIKW	0,6195
B44	HLA-B*44:13	1206	YEQYIKWPW	0,671
B44	HLA-B*44:16	95	TEKSNIIRGW	0,8679
B44	HLA-B*44:16	153	MESEFRVY	0,5269
B44	HLA-B*44:16	190	REFVFKNIDGY	0,5278
B44	HLA-B*44:16	297	SETKCTLKSF	0,5822
B44	HLA-B*44:16	339	GEVFNATRF	0,8995
B44	HLA-B*44:16	464	FERDISTEIY	0,6219
B44	HLA-B*44:16	653	AEHVNNSY	0,6553
B44	HLA-B*44:16	747	TECSNLLLQY	0,5015
B44	HLA-B*44:16	779	QEVFAQVKQIY	0,5029
B44	HLA-B*44:16	829	ADAGFIKQY	0,8245
B44	HLA-B*44:16	917	YENQKLIANQF	0,5047
B44	HLA-B*44:16	989	AEVQIDRLI	0,7927
B44	HLA-B*44:16	1016	AEIRASANL	0,745
B44	HLA-B*44:16	1091	REGVFVSNGTHW	0,7416
B44	HLA-B*44:16	1181	KEIDRLNEV	0,6515
B44	HLA-B*44:16	1201	QELGKYEQY	0,8678
B44	HLA-B*44:16	1201	QELGKYEQYIKW	0,5106
B44	HLA-B*44:16	1206	YEQYIKWPW	0,6551
B44	HLA-B*44:21	95	TEKSNIIRGW	0,94
B44	HLA-B*44:21	297	SETKCTLKSF	0,6607
B44	HLA-B*44:21	339	GEVFNATRF	0,8236
B44	HLA-B*44:21	989	AEVQIDRLI	0,8099
B44	HLA-B*44:21	1016	AEIRASANL	0,6996
B44	HLA-B*44:21	1091	REGVFVSNGTHW	0,682
B44	HLA-B*44:21	1201	QELGKYEQY	0,8862
B44	HLA-B*44:21	1201	QELGKYEQYIKW	0,6123
B44	HLA-B*44:21	1206	YEQYIKWPW	0,6936
B44	HLA-B*44:22	95	TEKSNIIRGW	0,9813
B44	HLA-B*44:22	153	MESEFRVY	0,5131
B44	HLA-B*44:22	297	SETKCTLKSF	0,7692
B44	HLA-B*44:22	339	GEVFNATRF	0,8968
B44	HLA-B*44:22	779	QEVFAQVKQIY	0,7113
B44	HLA-B*44:22	989	AEVQIDRLI	0,9059
B44	HLA-B*44:22	1016	AEIRASANL	0,8002
B44	HLA-B*44:22	1091	REGVFVSNGTHW	0,792
B44	HLA-B*44:22	1201	QELGKYEQY	0,9415
B44	HLA-B*44:22	1201	QELGKYEQYIKW	0,7097
B44	HLA-B*44:22	1206	YEQYIKWPW	0,7519
B44	HLA-B*44:24	95	TEKSNIIRGW	0,9813
B44	HLA-B*44:24	153	MESEFRVY	0,5131
B44	HLA-B*44:24	297	SETKCTLKSF	0,7692
B44	HLA-B*44:24	339	GEVFNATRF	0,8968
B44	HLA-B*44:24	779	QEVFAQVKQIY	0,7113
B44	HLA-B*44:24	989	AEVQIDRLI	0,9059

B44	HLA-B*44:24	1016	AEIRASANL	0,8002
B44	HLA-B*44:24	1091	REGVFVSNGTHW	0,792
B44	HLA-B*44:24	1201	QELGKYEQY	0,9415
B44	HLA-B*44:24	1201	QELGKYEQYIKW	0,7097
B44	HLA-B*44:24	1206	YEQYIKWPW	0,7519
B44	HLA-B*44:26	95	TEKSNIIRGW	0,9553
B44	HLA-B*44:26	153	MESEFRVY	0,5502
B44	HLA-B*44:26	297	SETKCTLKSF	0,7221
B44	HLA-B*44:26	339	GEVFNATRF	0,9332
B44	HLA-B*44:26	747	TECSNLLLQY	0,6025
B44	HLA-B*44:26	779	QEVFAQVKQIY	0,7054
B44	HLA-B*44:26	989	AEVQIDRLI	0,922
B44	HLA-B*44:26	1016	AEIRASANL	0,8443
B44	HLA-B*44:26	1091	REGVFVSNGTHW	0,7134
B44	HLA-B*44:26	1201	QELGKYEQY	0,9687
B44	HLA-B*44:26	1201	QELGKYEQYIKW	0,6195
B44	HLA-B*44:26	1206	YEQYIKWPW	0,671
B44	HLA-B*44:27	95	TEKSNIIRGW	0,9813
B44	HLA-B*44:27	153	MESEFRVY	0,5131
B44	HLA-B*44:27	297	SETKCTLKSF	0,7692
B44	HLA-B*44:27	339	GEVFNATRF	0,8968
B44	HLA-B*44:27	779	QEVFAQVKQIY	0,7113
B44	HLA-B*44:27	989	AEVQIDRLI	0,9059
B44	HLA-B*44:27	1016	AEIRASANL	0,8002
B44	HLA-B*44:27	1091	REGVFVSNGTHW	0,792
B44	HLA-B*44:27	1201	QELGKYEQY	0,9415
B44	HLA-B*44:27	1201	QELGKYEQYIKW	0,7097
B44	HLA-B*44:27	1206	YEQYIKWPW	0,7519
B44	HLA-B*44:28	95	TEKSNIIRGW	0,9464
B44	HLA-B*44:28	297	SETKCTLKSF	0,6716
B44	HLA-B*44:28	339	GEVFNATRF	0,8558
B44	HLA-B*44:28	747	TECSNLLLQY	0,5764
B44	HLA-B*44:28	989	AEVQIDRLI	0,8322
B44	HLA-B*44:28	1016	AEIRASANL	0,7302
B44	HLA-B*44:28	1091	REGVFVSNGTHW	0,6683
B44	HLA-B*44:28	1201	QELGKYEQY	0,92
B44	HLA-B*44:28	1201	QELGKYEQYIKW	0,5544
B44	HLA-B*44:28	1206	YEQYIKWPW	0,7125
B44	HLA-B*44:29	95	TEKSNIIRGW	0,9629
B44	HLA-B*44:29	153	MESEFRVY	0,6747
B44	HLA-B*44:29	190	REFVFKNIDGY	0,5841
B44	HLA-B*44:29	297	SETKCTLKSF	0,8275
B44	HLA-B*44:29	339	GEVFNATRF	0,9564
B44	HLA-B*44:29	464	FERDISTEIY	0,7133
B44	HLA-B*44:29	653	AEHVNNSY	0,8118
B44	HLA-B*44:29	747	TECSNLLLQY	0,7163
B44	HLA-B*44:29	779	QEVFAQVKQIY	0,8073
B44	HLA-B*44:29	829	ADAGFIKQY	0,8988
B44	HLA-B*44:29	917	YENQKLIANQF	0,5716
B44	HLA-B*44:29	989	AEVQIDRLI	0,9447

B44	HLA-B*44:29	1016	AEIRASANL	0,8969
B44	HLA-B*44:29	1091	REGVFVSNGTHW	0,8063
B44	HLA-B*44:29	1201	QELGKYEQY	0,9796
B44	HLA-B*44:29	1201	QELGKYEQYIKW	0,6973
B44	HLA-B*44:29	1206	YEQYIKWPW	0,7407
B44	HLA-B*44:30	95	TEKSNIIRGW	0,9553
B44	HLA-B*44:30	153	MESEFRVY	0,5502
B44	HLA-B*44:30	297	SETKCTLKSF	0,7221
B44	HLA-B*44:30	339	GEVFNATRF	0,9332
B44	HLA-B*44:30	747	TECSNLLLQY	0,6025
B44	HLA-B*44:30	779	QEVFAQVKQIY	0,7054
B44	HLA-B*44:30	989	AEVQIDRLI	0,922
B44	HLA-B*44:30	1016	AEIRASANL	0,8443
B44	HLA-B*44:30	1091	REGVFVSNGTHW	0,7134
B44	HLA-B*44:30	1201	QELGKYEQY	0,9687
B44	HLA-B*44:30	1201	QELGKYEQYIKW	0,6195
B44	HLA-B*44:30	1206	YEQYIKWPW	0,671
B44	HLA-B*44:32	95	TEKSNIIRGW	0,938
B44	HLA-B*44:32	297	SETKCTLKSF	0,6498
B44	HLA-B*44:32	339	GEVFNATRF	0,8844
B44	HLA-B*44:32	747	TECSNLLLQY	0,513
B44	HLA-B*44:32	779	QEVFAQVKQIY	0,6238
B44	HLA-B*44:32	989	AEVQIDRLI	0,8801
B44	HLA-B*44:32	1016	AEIRASANL	0,796
B44	HLA-B*44:32	1091	REGVFVSNGTHW	0,7563
B44	HLA-B*44:32	1201	QELGKYEQY	0,9492
B44	HLA-B*44:32	1201	QELGKYEQYIKW	0,5458
B44	HLA-B*44:32	1206	YEQYIKWPW	0,6216
B44	HLA-B*44:33	95	TEKSNIIRGW	0,9813
B44	HLA-B*44:33	153	MESEFRVY	0,5131
B44	HLA-B*44:33	297	SETKCTLKSF	0,7692
B44	HLA-B*44:33	339	GEVFNATRF	0,8968
B44	HLA-B*44:33	779	QEVFAQVKQIY	0,7113
B44	HLA-B*44:33	989	AEVQIDRLI	0,9059
B44	HLA-B*44:33	1016	AEIRASANL	0,8002
B44	HLA-B*44:33	1091	REGVFVSNGTHW	0,792
B44	HLA-B*44:33	1201	QELGKYEQY	0,9415
B44	HLA-B*44:33	1201	QELGKYEQYIKW	0,7097
B44	HLA-B*44:33	1206	YEQYIKWPW	0,7519
B44	HLA-B*44:35	95	TEKSNIIRGW	0,975
B44	HLA-B*44:35	153	MESEFRVY	0,5617
B44	HLA-B*44:35	297	SETKCTLKSF	0,7874
B44	HLA-B*44:35	339	GEVFNATRF	0,9155
B44	HLA-B*44:35	747	TECSNLLLQY	0,5932
B44	HLA-B*44:35	779	QEVFAQVKQIY	0,7582
B44	HLA-B*44:35	989	AEVQIDRLI	0,9053
B44	HLA-B*44:35	1016	AEIRASANL	0,8196
B44	HLA-B*44:35	1091	REGVFVSNGTHW	0,782
B44	HLA-B*44:35	1201	QELGKYEQY	0,9534
B44	HLA-B*44:35	1201	QELGKYEQYIKW	0,6824

B44	HLA-B*44:35	1206	YEQYIKWPW	0,7492
B44	HLA-B*44:36	95	TEKSNIIRGW	0,9553
B44	HLA-B*44:36	153	MESEFRVY	0,5502
B44	HLA-B*44:36	297	SETKCTLKSF	0,7221
B44	HLA-B*44:36	339	GEVFNATRF	0,9332
B44	HLA-B*44:36	747	TECSNLLLQY	0,6025
B44	HLA-B*44:36	779	QEVFAQVKQIY	0,7054
B44	HLA-B*44:36	989	AEVQIDRLI	0,922
B44	HLA-B*44:36	1016	AEIRASANL	0,8443
B44	HLA-B*44:36	1091	REGVFVSNGTHW	0,7134
B44	HLA-B*44:36	1201	QELGKYEQY	0,9687
B44	HLA-B*44:36	1201	QELGKYEQYIKW	0,6195
B44	HLA-B*44:36	1206	YEQYIKWPW	0,671
B44	HLA-B*44:37	95	TEKSNIIRGW	0,826
B44	HLA-B*44:37	153	MESEFRVY	0,5576
B44	HLA-B*44:37	190	REFVFKNIDGY	0,5135
B44	HLA-B*44:37	339	GEVFNATRF	0,9143
B44	HLA-B*44:37	464	FERDISTEIY	0,7083
B44	HLA-B*44:37	829	ADAGFIKQY	0,8697
B44	HLA-B*44:37	989	AEVQIDRLI	0,7629
B44	HLA-B*44:37	1016	AEIRASANL	0,7138
B44	HLA-B*44:37	1091	REGVFVSNGTHW	0,6777
B44	HLA-B*44:37	1201	QELGKYEQY	0,902
B44	HLA-B*44:37	1206	YEQYIKWPW	0,5676
B44	HLA-B*44:38	95	TEKSNIIRGW	0,9553
B44	HLA-B*44:38	153	MESEFRVY	0,5502
B44	HLA-B*44:38	297	SETKCTLKSF	0,7221
B44	HLA-B*44:38	339	GEVFNATRF	0,9332
B44	HLA-B*44:38	747	TECSNLLLQY	0,6025
B44	HLA-B*44:38	779	QEVFAQVKQIY	0,7054
B44	HLA-B*44:38	989	AEVQIDRLI	0,922
B44	HLA-B*44:38	1016	AEIRASANL	0,8443
B44	HLA-B*44:38	1091	REGVFVSNGTHW	0,7134
B44	HLA-B*44:38	1201	QELGKYEQY	0,9687
B44	HLA-B*44:38	1201	QELGKYEQYIKW	0,6195
B44	HLA-B*44:38	1206	YEQYIKWPW	0,671
B44	HLA-B*45:01	153	MESEFRVYS	0,6419
B44	HLA-B*45:01	155	SEFRVYSSA	0,8907
B44	HLA-B*45:01	280	NENGTITDA	0,7132
B44	HLA-B*45:01	618	TEVPVAIHA	0,9036
B44	HLA-B*45:01	867	DEMIAQYTSA	0,5083
B44	HLA-B*45:01	1016	AEIRASANL	0,678
B44	HLA-B*45:01	1016	AEIRASANLA	0,8233
B44	HLA-B*45:01	1016	AEIRASANLAA	0,6215
B44	HLA-B*45:01	1071	QEKNFTTAP	0,5423
B44	HLA-B*45:01	1071	QEKNFTTAPA	0,5531
B44	HLA-B*45:01	1181	KEIDRLNEV	0,7707
B44	HLA-B*45:01	1181	KEIDRLNEVA	0,6837
B44	HLA-B*45:03	153	MESEFRVYS	0,6419
B44	HLA-B*45:03	155	SEFRVYSSA	0,8907

B44	HLA-B*45:03	280	NENGTITDA	0,7132
B44	HLA-B*45:03	618	TEVPVAIHA	0,9036
B44	HLA-B*45:03	867	DEMIAQYTSA	0,5083
B44	HLA-B*45:03	1016	AEIRASANL	0,678
B44	HLA-B*45:03	1016	AEIRASANLA	0,8233
B44	HLA-B*45:03	1016	AEIRASANLAA	0,6215
B44	HLA-B*45:03	1071	QEKNFTTAP	0,5423
B44	HLA-B*45:03	1071	QEKNFTTAPA	0,5531
B44	HLA-B*45:03	1181	KEIDRLNEV	0,7707
B44	HLA-B*45:03	1181	KEIDRLNEVA	0,6837
B44	HLA-B*45:04	153	MESEFRVYS	0,6668
B44	HLA-B*45:04	155	SEFRVYSSA	0,8728
B44	HLA-B*45:04	168	FEYVSQPFL	0,6477
B44	HLA-B*45:04	280	NENGTITDA	0,6169
B44	HLA-B*45:04	339	GEVFNATRFA	0,5181
B44	HLA-B*45:04	464	FERDISTEI	0,7909
B44	HLA-B*45:04	515	FELLHAPAT	0,6115
B44	HLA-B*45:04	618	TEVPVAIHA	0,9037
B44	HLA-B*45:04	660	YECDIPIGA	0,7754
B44	HLA-B*45:04	724	TEILPVSMT	0,6427
B44	HLA-B*45:04	818	IEDLLFNKV	0,696
B44	HLA-B*45:04	989	AEVQIDRLI	0,7778
B44	HLA-B*45:04	1016	AEIRASANL	0,7431
B44	HLA-B*45:04	1016	AEIRASANLA	0,7358
B44	HLA-B*45:04	1016	AEIRASANLAA	0,6111
B44	HLA-B*45:04	1181	KEIDRLNEV	0,8925
B44	HLA-B*45:04	1181	KEIDRLNEVA	0,783
B44	HLA-B*45:05	153	MESEFRVYS	0,7073
B44	HLA-B*45:05	155	SEFRVYSSA	0,887
B44	HLA-B*45:05	280	NENGTITDA	0,7827
B44	HLA-B*45:05	280	NENGTITDAV	0,5501
B44	HLA-B*45:05	339	GEVFNATRFA	0,5214
B44	HLA-B*45:05	618	TEVPVAIHA	0,9095
B44	HLA-B*45:05	660	YECDIPIGA	0,6478
B44	HLA-B*45:05	867	DEMIAQYTS	0,5654
B44	HLA-B*45:05	867	DEMIAQYTSA	0,6146
B44	HLA-B*45:05	989	AEVQIDRLI	0,7684
B44	HLA-B*45:05	1016	AEIRASANL	0,6972
B44	HLA-B*45:05	1016	AEIRASANLA	0,8381
B44	HLA-B*45:05	1016	AEIRASANLAA	0,6471
B44	HLA-B*45:05	1071	QEKNFTTAP	0,638
B44	HLA-B*45:05	1071	QEKNFTTAPA	0,6366
B44	HLA-B*45:05	1181	KEIDRLNEV	0,7759
B44	HLA-B*45:05	1181	KEIDRLNEVA	0,7116
B44	HLA-B*45:07	153	MESEFRVYS	0,6419
B44	HLA-B*45:07	155	SEFRVYSSA	0,8907
B44	HLA-B*45:07	280	NENGTITDA	0,7132
B44	HLA-B*45:07	618	TEVPVAIHA	0,9036
B44	HLA-B*45:07	867	DEMIAQYTSA	0,5083
B44	HLA-B*45:07	1016	AEIRASANL	0,678

B44	HLA-B*45:07	1016	AEIRASANLA	0,8233
B44	HLA-B*45:07	1016	AEIRASANLAA	0,6215
B44	HLA-B*45:07	1071	QEKNFTTAP	0,5423
B44	HLA-B*45:07	1071	QEKNFTTAPA	0,5531
B44	HLA-B*45:07	1181	KEIDRLNEV	0,7707
B44	HLA-B*45:07	1181	KEIDRLNEVA	0,6837
B44	HLA-B*49:04	339	GEVFNATRF	0,7684
B44	HLA-B*49:04	464	FERDISTEI	0,6468
B44	HLA-B*49:04	989	AEVQIDRLI	0,7729
B44	HLA-B*50:01	153	MESEFRVYS	0,5279
B44	HLA-B*50:01	155	SEFRVYSSA	0,9313
B44	HLA-B*50:01	168	FEYVSQPFL	0,5682
B44	HLA-B*50:01	464	FERDISTEI	0,8164
B44	HLA-B*50:01	515	FELLHAPAT	0,5692
B44	HLA-B*50:01	618	TEVPVAIHA	0,8958
B44	HLA-B*50:01	660	YECDIPIGA	0,7228
B44	HLA-B*50:01	1016	AEIRASANL	0,635
B44	HLA-B*50:01	1016	AEIRASANLA	0,6321
B44	HLA-B*50:01	1181	KEIDRLNEV	0,8625
B44	HLA-B*50:01	1181	KEIDRLNEVA	0,7661
B44	HLA-B*50:02	155	SEFRVYSSA	0,8534
B44	HLA-B*50:02	280	NENGTITDA	0,5939
B44	HLA-B*50:02	618	TEVPVAIHA	0,8364
B44	HLA-B*50:02	1016	AEIRASANLA	0,6537
B44	HLA-B*50:02	1181	KEIDRLNEVA	0,5821
B44	HLA-B*50:04	153	MESEFRVYS	0,5279
B44	HLA-B*50:04	155	SEFRVYSSA	0,9313
B44	HLA-B*50:04	168	FEYVSQPFL	0,5682
B44	HLA-B*50:04	464	FERDISTEI	0,8164
B44	HLA-B*50:04	515	FELLHAPAT	0,5692
B44	HLA-B*50:04	618	TEVPVAIHA	0,8958
B44	HLA-B*50:04	660	YECDIPIGA	0,7228
B44	HLA-B*50:04	1016	AEIRASANL	0,635
B44	HLA-B*50:04	1016	AEIRASANLA	0,6321
B44	HLA-B*50:04	1181	KEIDRLNEV	0,8625
B44	HLA-B*50:04	1181	KEIDRLNEVA	0,7661
B58	HLA-B*15:16	34	RGVYYPDKVF	0,6031
B58	HLA-B*15:16	50	STQDLFLPF	0,5575
B58	HLA-B*15:16	160	YSSANNCTF	0,7436
B58	HLA-B*15:16	258	WTAGAAAYY	0,6769
B58	HLA-B*15:16	267	VGYLQPRTF	0,6255
B58	HLA-B*15:16	304	KSFTVEKGI	0,6627
B58	HLA-B*15:16	344	ATRFASVYAW	0,5424
B58	HLA-B*15:16	372	ASFSTFKCY	0,5797
B58	HLA-B*15:16	392	FTNVYADSF	0,6268
B58	HLA-B*15:16	590	CSFGGVSVI	0,5898
B58	HLA-B*15:16	604	TSNQVAVLY	0,7291
B58	HLA-B*15:16	634	RVYSTGSNV	0,5284
B58	HLA-B*15:16	634	RVYSTGSNVF	0,7452
B58	HLA-B*15:16	685	RSVASQSII	0,7268

B58	HLA-B*15:16	687	VASQSIIAY	0,7282
B58	HLA-B*15:16	710	NSIAIPTNF	0,7782
B58	HLA-B*15:16	712	IAIPTNFTI	0,8768
B58	HLA-B*15:16	718	FTISVTTEI	0,7002
B58	HLA-B*15:16	733	KTSVDCTMY	0,5204
B58	HLA-B*15:16	814	KRSFIEDLLF	0,6401
B58	HLA-B*15:16	815	RSFIEDLLF	0,9347
B58	HLA-B*15:16	825	KVTLADAGF	0,5684
B58	HLA-B*15:16	865	LTDEMIAQY	0,7522
B58	HLA-B*15:16	878	LAGTITSGW	0,5789
B58	HLA-B*15:16	879	AGTITSGWTF	0,542
B58	HLA-B*15:16	880	GTITSGWTF	0,8294
B58	HLA-B*15:16	886	WTFGAGAAL	0,5705
B58	HLA-B*15:16	898	FAMQMAYRF	0,655
B58	HLA-B*15:16	923	IANQFNSAI	0,5744
B58	HLA-B*15:16	1005	QTYVTQQLI	0,6943
B58	HLA-B*15:16	1054	QSAPHGVVF	0,8856
B58	HLA-B*15:16	1086	KAHFPREGVF	0,5268
B58	HLA-B*15:16	1093	GVFVSNGTHW	0,5731
B58	HLA-B*15:17	21	RTQLPPAY	0,729
B58	HLA-B*15:17	28	YTNSFTRGVY	0,712
B58	HLA-B*15:17	29	TNSFTRGVYY	0,6428
B58	HLA-B*15:17	30	NSFTRGVYY	0,8886
B58	HLA-B*15:17	34	RGVYYPDKVF	0,7986
B58	HLA-B*15:17	35	GVYYPDKVF	0,8911
B58	HLA-B*15:17	50	STQDLFLPF	0,7926
B58	HLA-B*15:17	158	RVYSSANNCTF	0,6804
B58	HLA-B*15:17	160	YSSANNCTF	0,8168
B58	HLA-B*15:17	192	FVFKNIDGY	0,8376
B58	HLA-B*15:17	204	YSKHTPINL	0,7655
B58	HLA-B*15:17	212	LVRDLPQGF	0,867
B58	HLA-B*15:17	257	GWTAGAAAYY	0,6825
B58	HLA-B*15:17	258	WTAGAAAYY	0,9236
B58	HLA-B*15:17	261	GAAAYYVGY	0,8951
B58	HLA-B*15:17	267	VGYLQPRTF	0,7451
B58	HLA-B*15:17	304	KSFTVEKGI	0,6634
B58	HLA-B*15:17	304	KSFTVEKGIY	0,7603
B58	HLA-B*15:17	310	KGIYQTSNF	0,719
B58	HLA-B*15:17	344	ATRFASVYAW	0,7702
B58	HLA-B*15:17	361	CVADYSVLY	0,6496
B58	HLA-B*15:17	366	SVLYNSASF	0,8204
B58	HLA-B*15:17	372	ASFSTFKCY	0,828
B58	HLA-B*15:17	392	FTNVYADSF	0,7806
B58	HLA-B*15:17	604	TSNQVAVLY	0,9455
B58	HLA-B*15:17	625	HADQLTPTW	0,846
B58	HLA-B*15:17	634	RVYSTGSNV	0,6546
B58	HLA-B*15:17	634	RVYSTGSNVF	0,9115
B58	HLA-B*15:17	685	RSVASQSII	0,7537
B58	HLA-B*15:17	687	VASQSIIAY	0,9477
B58	HLA-B*15:17	710	NSIAIPTNF	0,9195

B58	HLA-B*15:17	712	IAIPTNFTI	0,867
B58	HLA-B*15:17	718	FTISVTTEI	0,7184
B58	HLA-B*15:17	733	KTSVDCTMY	0,8517
B58	HLA-B*15:17	814	KRSFIEDLLF	0,7282
B58	HLA-B*15:17	815	RSFIEDLLF	0,9846
B58	HLA-B*15:17	825	KVTLADAGF	0,8775
B58	HLA-B*15:17	865	LTDEMIAQY	0,9166
B58	HLA-B*15:17	878	LAGTITSGW	0,7922
B58	HLA-B*15:17	879	AGTITSGWTF	0,7247
B58	HLA-B*15:17	880	GTITSGWTF	0,9585
B58	HLA-B*15:17	886	WTFGAGAAL	0,7745
B58	HLA-B*15:17	892	AALQIPFAM	0,6696
B58	HLA-B*15:17	898	FAMQMAYRF	0,7486
B58	HLA-B*15:17	940	STASALGKL	0,7384
B58	HLA-B*15:17	962	LVKQLSSNF	0,7867
B58	HLA-B*15:17	1005	QTYVTQQLI	0,7371
B58	HLA-B*15:17	1021	SANLAATKM	0,6738
B58	HLA-B*15:17	1054	QSAPHGVVF	0,9815
B58	HLA-B*15:17	1086	KAHFPREGVF	0,7038
B58	HLA-B*15:17	1093	GVFVSNGTHW	0,7853
B58	HLA-B*15:17	1095	FVSNGTHWF	0,6814
B58	HLA-B*15:67	34	RGVYYPDKVF	0,6031
B58	HLA-B*15:67	50	STQDLFLPF	0,5575
B58	HLA-B*15:67	160	YSSANNCTF	0,7436
B58	HLA-B*15:67	258	WTAGAAAYY	0,6769
B58	HLA-B*15:67	267	VGYLQPRTF	0,6255
B58	HLA-B*15:67	304	KSFTVEKGI	0,6627
B58	HLA-B*15:67	344	ATRFASVYAW	0,5424
B58	HLA-B*15:67	372	ASFSTFKCY	0,5797
B58	HLA-B*15:67	392	FTNVYADSF	0,6268
B58	HLA-B*15:67	590	CSFGGVSVI	0,5898
B58	HLA-B*15:67	604	TSNQVAVLY	0,7291
B58	HLA-B*15:67	634	RVYSTGSNV	0,5284
B58	HLA-B*15:67	634	RVYSTGSNVF	0,7452
B58	HLA-B*15:67	685	RSVASQSII	0,7268
B58	HLA-B*15:67	687	VASQSIIAY	0,7282
B58	HLA-B*15:67	710	NSIAIPTNF	0,7782
B58	HLA-B*15:67	712	IAIPTNFTI	0,8768
B58	HLA-B*15:67	718	FTISVTTEI	0,7002
B58	HLA-B*15:67	733	KTSVDCTMY	0,5204
B58	HLA-B*15:67	814	KRSFIEDLLF	0,6401
B58	HLA-B*15:67	815	RSFIEDLLF	0,9347
B58	HLA-B*15:67	825	KVTLADAGF	0,5684
B58	HLA-B*15:67	865	LTDEMIAQY	0,7522
B58	HLA-B*15:67	878	LAGTITSGW	0,5789
B58	HLA-B*15:67	879	AGTITSGWTF	0,542
B58	HLA-B*15:67	880	GTITSGWTF	0,8294
B58	HLA-B*15:67	886	WTFGAGAAL	0,5705
B58	HLA-B*15:67	898	FAMQMAYRF	0,655
B58	HLA-B*15:67	923	IANQFNSAI	0,5744

B58	HLA-B*15:67	1005	QTYVTQQLI	0,6943
B58	HLA-B*15:67	1054	QSAPHGVVF	0,8856
B58	HLA-B*15:67	1086	KAHFPREGVF	0,5268
B58	HLA-B*15:67	1093	GVFVSNGTHW	0,5731
B58	HLA-B*15:95	34	RGVYYPDKVF	0,5969
B58	HLA-B*15:95	50	STQDLFLPF	0,5282
B58	HLA-B*15:95	158	RVYSSANNCTF	0,5058
B58	HLA-B*15:95	160	YSSANNCTF	0,7058
B58	HLA-B*15:95	258	WTAGAAAYY	0,6382
B58	HLA-B*15:95	267	VGYLQPRTF	0,6579
B58	HLA-B*15:95	304	KSFTVEKGI	0,6907
B58	HLA-B*15:95	304	KSFTVEKGIY	0,5666
B58	HLA-B*15:95	310	KGIYQTSNF	0,5569
B58	HLA-B*15:95	344	ATRFASVYAW	0,5798
B58	HLA-B*15:95	372	ASFSTFKCY	0,6011
B58	HLA-B*15:95	392	FTNVYADSF	0,5426
B58	HLA-B*15:95	590	CSFGGVSVI	0,5003
B58	HLA-B*15:95	604	TSNQVAVLY	0,7366
B58	HLA-B*15:95	625	HADQLTPTW	0,7356
B58	HLA-B*15:95	634	RVYSTGSNVF	0,7402
B58	HLA-B*15:95	685	RSVASQSII	0,7531
B58	HLA-B*15:95	687	VASQSIIAY	0,7264
B58	HLA-B*15:95	710	NSIAIPTNF	0,7623
B58	HLA-B*15:95	712	IAIPTNFTI	0,8481
B58	HLA-B*15:95	718	FTISVTTEI	0,5787
B58	HLA-B*15:95	733	KTSVDCTMY	0,5963
B58	HLA-B*15:95	814	KRSFIEDLLF	0,6782
B58	HLA-B*15:95	815	RSFIEDLLF	0,9594
B58	HLA-B*15:95	825	KVTLADAGF	0,5677
B58	HLA-B*15:95	878	LAGTITSGW	0,5751
B58	HLA-B*15:95	879	AGTITSGWTF	0,5289
B58	HLA-B*15:95	880	GTITSGWTF	0,8368
B58	HLA-B*15:95	898	FAMQMAYRF	0,5752
B58	HLA-B*15:95	923	IANQFNSAI	0,5088
B58	HLA-B*15:95	1005	QTYVTQQLI	0,6101
B58	HLA-B*15:95	1054	QSAPHGVVF	0,8744
B58	HLA-B*15:95	1086	KAHFPREGVF	0,5522
B58	HLA-B*15:95	1093	GVFVSNGTHW	0,6012
B58	HLA-B*57:01	97	KSNIIRGW	0,8688
B58	HLA-B*57:01	304	KSFTVEKGI	0,5385
B58	HLA-B*57:01	344	ATRFASVYAW	0,7634
B58	HLA-B*57:01	622	VAIHADQLTPTW	0,5506
B58	HLA-B*57:01	625	HADQLTPTW	0,8783
B58	HLA-B*57:01	712	IAIPTNFTI	0,5812
B58	HLA-B*57:01	815	RSFIEDLLF	0,868
B58	HLA-B*57:01	878	LAGTITSGW	0,7806
B58	HLA-B*57:01	880	GTITSGWTF	0,7834
B58	HLA-B*57:01	1093	GVFVSNGTHW	0,7168
B58	HLA-B*57:02	160	YSSANNCTF	0,5613
B58	HLA-B*57:02	249	LTPGDSSSGW	0,5051

B58	HLA-B*57:02	344	ATRFASVYAW	0,6112
B58	HLA-B*57:02	622	VAIHADQLTPTW	0,6009
B58	HLA-B*57:02	624	IHADQLTPTW	0,6898
B58	HLA-B*57:02	625	HADQLTPTW	0,9339
B58	HLA-B*57:02	712	IAIPTNFTI	0,6703
B58	HLA-B*57:02	815	RSFIEDLLF	0,8365
B58	HLA-B*57:02	878	LAGTITSGW	0,7731
B58	HLA-B*57:02	880	GTITSGWTF	0,7332
B58	HLA-B*57:02	1054	QSAPHGVVF	0,8599
B58	HLA-B*57:03	97	KSNIIRGW	0,5712
B58	HLA-B*57:03	160	YSSANNCTF	0,52
B58	HLA-B*57:03	304	KSFTVEKGI	0,5693
B58	HLA-B*57:03	344	ATRFASVYAW	0,6947
B58	HLA-B*57:03	622	VAIHADQLTPTW	0,6476
B58	HLA-B*57:03	624	IHADQLTPTW	0,6328
B58	HLA-B*57:03	625	HADQLTPTW	0,9284
B58	HLA-B*57:03	634	RVYSTGSNVF	0,5104
B58	HLA-B*57:03	685	RSVASQSII	0,56
B58	HLA-B*57:03	710	NSIAIPTNF	0,7756
B58	HLA-B*57:03	712	IAIPTNFTI	0,8166
B58	HLA-B*57:03	814	KRSFIEDLLF	0,5259
B58	HLA-B*57:03	815	RSFIEDLLF	0,9134
B58	HLA-B*57:03	825	KVTLADAGF	0,5236
B58	HLA-B*57:03	878	LAGTITSGW	0,816
B58	HLA-B*57:03	880	GTITSGWTF	0,8295
B58	HLA-B*57:03	898	FAMQMAYRF	0,5657
B58	HLA-B*57:03	1054	QSAPHGVVF	0,8674
B58	HLA-B*57:03	1093	GVFVSNGTHW	0,6139
B58	HLA-B*57:07	344	ATRFASVYAW	0,5647
B58	HLA-B*57:07	625	HADQLTPTW	0,8776
B58	HLA-B*57:07	712	IAIPTNFTI	0,6961
B58	HLA-B*57:07	815	RSFIEDLLF	0,7639
B58	HLA-B*57:07	878	LAGTITSGW	0,6899
B58	HLA-B*57:07	880	GTITSGWTF	0,6913
B58	HLA-B*57:07	898	FAMQMAYRF	0,5331
B58	HLA-B*57:08	97	KSNIIRGW	0,8688
B58	HLA-B*57:08	304	KSFTVEKGI	0,5385
B58	HLA-B*57:08	344	ATRFASVYAW	0,7634
B58	HLA-B*57:08	622	VAIHADQLTPTW	0,5506
B58	HLA-B*57:08	625	HADQLTPTW	0,8783
B58	HLA-B*57:08	712	IAIPTNFTI	0,5812
B58	HLA-B*57:08	815	RSFIEDLLF	0,868
B58	HLA-B*57:08	878	LAGTITSGW	0,7806
B58	HLA-B*57:08	880	GTITSGWTF	0,7834
B58	HLA-B*57:08	1093	GVFVSNGTHW	0,7168
B58	HLA-B*57:09	344	ATRFASVYAW	0,5174
B58	HLA-B*57:09	634	RVYSTGSNVF	0,591
B58	HLA-B*57:09	712	IAIPTNFTI	0,5816
B58	HLA-B*57:09	815	RSFIEDLLF	0,7555
B58	HLA-B*57:09	878	LAGTITSGW	0,5423

B58	HLA-B*57:09	880	GTITSGWTF	0,7045
B58	HLA-B*57:09	1054	QSAPHGVVF	0,7826
B58	HLA-B*57:09	1086	KAHFPREGVF	0,5053
B58	HLA-B*58:01	97	KSNIIRGW	0,6994
B58	HLA-B*58:01	160	YSSANNCTF	0,5597
B58	HLA-B*58:01	344	ATRFASVYAW	0,7778
B58	HLA-B*58:01	604	TSNQVAVLY	0,7554
B58	HLA-B*58:01	622	VAIHADQLTPTW	0,7607
B58	HLA-B*58:01	624	IHADQLTPTW	0,7759
B58	HLA-B*58:01	625	HADQLTPTW	0,9737
B58	HLA-B*58:01	687	VASQSIIAY	0,6802
B58	HLA-B*58:01	710	NSIAIPTNF	0,8133
B58	HLA-B*58:01	712	IAIPTNFTI	0,8079
B58	HLA-B*58:01	814	KRSFIEDLLF	0,5656
B58	HLA-B*58:01	815	RSFIEDLLF	0,9373
B58	HLA-B*58:01	878	LAGTITSGW	0,8666
B58	HLA-B*58:01	880	GTITSGWTF	0,879
B58	HLA-B*58:01	898	FAMQMAYRF	0,6403
B58	HLA-B*58:01	1054	QSAPHGVVF	0,8091
B58	HLA-B*58:01	1093	GVFVSNGTHW	0,7252
B58	HLA-B*58:02	815	RSFIEDLLF	0,6532
B58	HLA-B*58:04	56	LPFFSNVTW	0,5448
B58	HLA-B*58:04	97	KSNIIRGW	0,6084
B58	HLA-B*58:04	160	YSSANNCTF	0,5817
B58	HLA-B*58:04	344	ATRFASVYAW	0,7355
B58	HLA-B*58:04	604	TSNQVAVLY	0,7164
B58	HLA-B*58:04	622	VAIHADQLTPTW	0,7105
B58	HLA-B*58:04	624	IHADQLTPTW	0,7479
B58	HLA-B*58:04	625	HADQLTPTW	0,9681
B58	HLA-B*58:04	687	VASQSIIAY	0,648
B58	HLA-B*58:04	710	NSIAIPTNF	0,7658
B58	HLA-B*58:04	712	IAIPTNFTI	0,7552
B58	HLA-B*58:04	814	KRSFIEDLLF	0,5733
B58	HLA-B*58:04	815	RSFIEDLLF	0,9452
B58	HLA-B*58:04	878	LAGTITSGW	0,801
B58	HLA-B*58:04	880	GTITSGWTF	0,8598
B58	HLA-B*58:04	892	AALQIPFAM	0,5093
B58	HLA-B*58:04	898	FAMQMAYRF	0,6552
B58	HLA-B*58:04	1054	QSAPHGVVF	0,808
B58	HLA-B*58:04	1093	GVFVSNGTHW	0,6243
B58	HLA-B*58:06	815	RSFIEDLLF	0,7373
B58	HLA-B*58:08	62	VTWFHAIHV	0,5966
B58	HLA-B*58:08	304	KSFTVEKGI	0,7929
B58	HLA-B*58:08	590	CSFGGVSVI	0,664
B58	HLA-B*58:08	685	RSVASQSI	0,5963
B58	HLA-B*58:08	685	RSVASQSII	0,7492
B58	HLA-B*58:08	697	MSLGAENSV	0,5739
B58	HLA-B*58:08	711	SIAIPTNFTI	0,6417
B58	HLA-B*58:08	712	IAIPTNFTI	0,9331
B58	HLA-B*58:08	718	FTISVTTEI	0,7015

B58	HLA-B*58:08	815	RSFIEDLLF	0,7859
B58	HLA-B*58:08	923	IANQFNSAI	0,6908
B58	HLA-B*58:08	1005	QTYVTQQLI	0,8005
B58	HLA-B*58:09	56	LPFFSNVTW	0,6098
B58	HLA-B*58:09	97	KSNIIRGW	0,7131
B58	HLA-B*58:09	160	YSSANNCTF	0,5758
B58	HLA-B*58:09	258	WTAGAAAYY	0,5718
B58	HLA-B*58:09	304	KSFTVEKGIY	0,5095
B58	HLA-B*58:09	344	ATRFASVYAW	0,7902
B58	HLA-B*58:09	604	TSNQVAVLY	0,8181
B58	HLA-B*58:09	622	VAIHADQLTPTW	0,8017
B58	HLA-B*58:09	623	AIHADQLTPTW	0,5232
B58	HLA-B*58:09	624	IHADQLTPTW	0,8068
B58	HLA-B*58:09	625	HADQLTPTW	0,969
B58	HLA-B*58:09	687	VASQSIIAY	0,7266
B58	HLA-B*58:09	710	NSIAIPTNF	0,8466
B58	HLA-B*58:09	712	IAIPTNFTI	0,8112
B58	HLA-B*58:09	733	KTSVDCTMY	0,5183
B58	HLA-B*58:09	814	KRSFIEDLLF	0,5561
B58	HLA-B*58:09	815	RSFIEDLLF	0,8962
B58	HLA-B*58:09	878	LAGTITSGW	0,8826
B58	HLA-B*58:09	879	AGTITSGWTF	0,5228
B58	HLA-B*58:09	880	GTITSGWTF	0,86
B58	HLA-B*58:09	892	AALQIPFAM	0,5678
B58	HLA-B*58:09	898	FAMQMAYRF	0,7372
B58	HLA-B*58:09	1054	QSAPHGVVF	0,8091
B58	HLA-B*58:09	1093	GVFVSNGTHW	0,7325
B58	HLA-B*58:11	97	KSNIIRGW	0,6994
B58	HLA-B*58:11	160	YSSANNCTF	0,5597
B58	HLA-B*58:11	344	ATRFASVYAW	0,7778
B58	HLA-B*58:11	604	TSNQVAVLY	0,7554
B58	HLA-B*58:11	622	VAIHADQLTPTW	0,7607
B58	HLA-B*58:11	624	IHADQLTPTW	0,7759
B58	HLA-B*58:11	625	HADQLTPTW	0,9737
B58	HLA-B*58:11	687	VASQSIIAY	0,6802
B58	HLA-B*58:11	710	NSIAIPTNF	0,8133
B58	HLA-B*58:11	712	IAIPTNFTI	0,8079
B58	HLA-B*58:11	814	KRSFIEDLLF	0,5656
B58	HLA-B*58:11	815	RSFIEDLLF	0,9373
B58	HLA-B*58:11	878	LAGTITSGW	0,8666
B58	HLA-B*58:11	880	GTITSGWTF	0,879
B58	HLA-B*58:11	898	FAMQMAYRF	0,6403
B58	HLA-B*58:11	1054	QSAPHGVVF	0,8091
B58	HLA-B*58:11	1093	GVFVSNGTHW	0,7252
B62	HLA-B*15:01	35	GVYYPDKVF	0,677
B62	HLA-B*15:01	47	VLHSTQDLF	0,5635
B62	HLA-B*15:01	192	FVFKNIDGY	0,678
B62	HLA-B*15:01	212	LVRDLPQGF	0,7136
B62	HLA-B*15:01	240	TLLALHRSY	0,6805
B62	HLA-B*15:01	258	WTAGAAAYY	0,5072

B62	HLA-B*15:01	261	GAAAYYVGY	0,52
B62	HLA-B*15:01	366	SVLYNSASF	0,5995
B62	HLA-B*15:01	413	GQTGKIADY	0,764
B62	HLA-B*15:01	497	FQPTNGVGY	0,7039
B62	HLA-B*15:01	628	QLTPTWRVY	0,6565
B62	HLA-B*15:01	634	RVYSTGSNVF	0,8491
B62	HLA-B*15:01	686	SVASQSIIAY	0,5924
B62	HLA-B*15:01	687	VASQSIIAY	0,7822
B62	HLA-B*15:01	698	SLGAENSVAY	0,6839
B62	HLA-B*15:01	880	GTITSGWTF	0,6048
B62	HLA-B*15:01	894	LQIPFAMQM	0,6403
B62	HLA-B*15:01	919	NQKLIANQF	0,7035
B62	HLA-B*15:01	962	LVKQLSSNF	0,7084
B62	HLA-B*15:01	1000	RLQSLQTY	0,5767
B62	HLA-B*15:01	1054	QSAPHGVVF	0,7211
B62	HLA-B*15:01	1113	QIITTDNTF	0,6229
B62	HLA-B*15:01	1264	VLKGVKLHY	0,8811
B62	HLA-B*15:02	84	LPFNDGVYF	0,5012
B62	HLA-B*15:02	192	FVFKNIDGY	0,6067
B62	HLA-B*15:02	212	LVRDLPQGF	0,5599
B62	HLA-B*15:02	240	TLLALHRSY	0,6225
B62	HLA-B*15:02	366	SVLYNSASF	0,593
B62	HLA-B*15:02	628	QLTPTWRVY	0,6554
B62	HLA-B*15:02	687	VASQSIIAY	0,8247
B62	HLA-B*15:02	698	SLGAENSVAY	0,593
B62	HLA-B*15:02	699	LGAENSVAY	0,5304
B62	HLA-B*15:02	896	IPFAMQMAY	0,6693
B62	HLA-B*15:02	1054	QSAPHGVVF	0,678
B62	HLA-B*15:02	1113	QIITTDNTF	0,6078
B62	HLA-B*15:05	192	FVFKNIDGY	0,568
B62	HLA-B*15:05	240	TLLALHRSY	0,5065
B62	HLA-B*15:05	413	GQTGKIADY	0,5265
B62	HLA-B*15:05	497	FQPTNGVGY	0,5981
B62	HLA-B*15:05	634	RVYSTGSNVF	0,5053
B62	HLA-B*15:05	687	VASQSIIAY	0,7516
B62	HLA-B*15:05	689	SQSIIAYTM	0,5439
B62	HLA-B*15:05	880	GTITSGWTF	0,603
B62	HLA-B*15:05	894	LQIPFAMQM	0,5925
B62	HLA-B*15:05	1054	QSAPHGVVF	0,588
B62	HLA-B*15:05	1113	QIITTDNTF	0,5456
B62	HLA-B*15:12	628	QLTPTWRVY	0,5076
B62	HLA-B*15:12	634	RVYSTGSNVF	0,5866
B62	HLA-B*15:12	687	VASQSIIAY	0,588
B62	HLA-B*15:12	1054	QSAPHGVVF	0,5578
B62	HLA-B*15:14	634	RVYSTGSNVF	0,5315
B62	HLA-B*15:15	192	FVFKNIDGY	0,6152
B62	HLA-B*15:15	212	LVRDLPQGF	0,6015
B62	HLA-B*15:15	240	TLLALHRSY	0,5454
B62	HLA-B*15:15	366	SVLYNSASF	0,5295
B62	HLA-B*15:15	628	QLTPTWRVY	0,5711

B62	HLA-B*15:15	634	RVYSTGSNVF	0,5219
B62	HLA-B*15:15	686	SVASQSIIAY	0,5315
B62	HLA-B*15:15	687	VASQSIIAY	0,7424
B62	HLA-B*15:15	698	SLGAENSVAY	0,6339
B62	HLA-B*15:15	699	LGAENSVAY	0,5063
B62	HLA-B*15:15	919	NQKLIANQF	0,6734
B62	HLA-B*15:15	962	LVKQLSSNF	0,5773
B62	HLA-B*15:15	1054	QSAPHGVVF	0,6835
B62	HLA-B*15:15	1113	QIITTDNTF	0,6196
B62	HLA-B*15:19	628	QLTPTWRVY	0,5076
B62	HLA-B*15:19	634	RVYSTGSNVF	0,5866
B62	HLA-B*15:19	687	VASQSIIAY	0,588
B62	HLA-B*15:19	1054	QSAPHGVVF	0,5578
B62	HLA-B*15:20	192	FVFKNIDGY	0,6015
B62	HLA-B*15:20	240	TLLALHRSY	0,5374
B62	HLA-B*15:20	497	FQPTNGVGY	0,5717
B62	HLA-B*15:20	628	QLTPTWRVY	0,5008
B62	HLA-B*15:20	687	VASQSIIAY	0,764
B62	HLA-B*15:20	689	SQSIIAYTM	0,525
B62	HLA-B*15:20	698	SLGAENSVAY	0,5119
B62	HLA-B*15:20	880	GTITSGWTF	0,5777
B62	HLA-B*15:20	894	LQIPFAMQM	0,5729
B62	HLA-B*15:20	1054	QSAPHGVVF	0,5705
B62	HLA-B*15:20	1113	QIITTDNTF	0,55
B62	HLA-B*15:25	30	NSFTRGVYY	0,5102
B62	HLA-B*15:25	35	GVYYPDKVF	0,7166
B62	HLA-B*15:25	47	VLHSTQDLF	0,5497
B62	HLA-B*15:25	152	WMESEFRVY	0,5406
B62	HLA-B*15:25	192	FVFKNIDGY	0,6774
B62	HLA-B*15:25	212	LVRDLPQGF	0,6749
B62	HLA-B*15:25	240	TLLALHRSY	0,7448
B62	HLA-B*15:25	261	GAAAYYVGY	0,5614
B62	HLA-B*15:25	366	SVLYNSASF	0,6588
B62	HLA-B*15:25	413	GQTGKIADY	0,6454
B62	HLA-B*15:25	497	FQPTNGVGY	0,6173
B62	HLA-B*15:25	628	QLTPTWRVY	0,7063
B62	HLA-B*15:25	634	RVYSTGSNVF	0,8174
B62	HLA-B*15:25	686	SVASQSIIAY	0,5343
B62	HLA-B*15:25	687	VASQSIIAY	0,8425
B62	HLA-B*15:25	698	SLGAENSVAY	0,6499
B62	HLA-B*15:25	699	LGAENSVAY	0,5125
B62	HLA-B*15:25	880	GTITSGWTF	0,6109
B62	HLA-B*15:25	894	LQIPFAMQM	0,6635
B62	HLA-B*15:25	962	LVKQLSSNF	0,5897
B62	HLA-B*15:25	1000	RLQSLQTY	0,5751
B62	HLA-B*15:25	1054	QSAPHGVVF	0,6973
B62	HLA-B*15:25	1113	QIITTDNTF	0,6015
B62	HLA-B*15:25	1264	VLKGVKLHY	0,8551
B62	HLA-B*15:28	35	GVYYPDKVF	0,677
B62	HLA-B*15:28	47	VLHSTQDLF	0,5635

B62	HLA-B*15:28	192	FVFKNIDGY	0,678
B62	HLA-B*15:28	212	LVRDLPQGF	0,7136
B62	HLA-B*15:28	240	TLLALHRSY	0,6805
B62	HLA-B*15:28	258	WTAGAAAYY	0,5072
B62	HLA-B*15:28	261	GAAAYYVGY	0,52
B62	HLA-B*15:28	366	SVLYNSASF	0,5995
B62	HLA-B*15:28	413	GQTGKIADY	0,764
B62	HLA-B*15:28	497	FQPTNGVGY	0,7039
B62	HLA-B*15:28	628	QLTPTWRVY	0,6565
B62	HLA-B*15:28	634	RVYSTGSNVF	0,8491
B62	HLA-B*15:28	686	SVASQSIIAY	0,5924
B62	HLA-B*15:28	687	VASQSIIAY	0,7822
B62	HLA-B*15:28	698	SLGAENSVAY	0,6839
B62	HLA-B*15:28	880	GTITSGWTF	0,6048
B62	HLA-B*15:28	894	LQIPFAMQM	0,6403
B62	HLA-B*15:28	919	NQKLIANQF	0,7035
B62	HLA-B*15:28	962	LVKQLSSNF	0,7084
B62	HLA-B*15:28	1000	RLQSLQTY	0,5767
B62	HLA-B*15:28	1054	QSAPHGVVF	0,7211
B62	HLA-B*15:28	1113	QIITTDNTF	0,6229
B62	HLA-B*15:28	1264	VLKGVKLHY	0,8811
B62	HLA-B*15:31	84	LPFNDGVYF	0,5604
B62	HLA-B*15:31	192	FVFKNIDGY	0,5375
B62	HLA-B*15:31	687	VASQSIIAY	0,7264
B62	HLA-B*15:31	896	IPFAMQMAY	0,5929
B62	HLA-B*15:31	1054	QSAPHGVVF	0,5241
B62	HLA-B*15:31	1113	QIITTDNTF	0,5135
B62	HLA-B*15:33	35	GVYYPDKVF	0,677
B62	HLA-B*15:33	47	VLHSTQDLF	0,5635
B62	HLA-B*15:33	192	FVFKNIDGY	0,678
B62	HLA-B*15:33	212	LVRDLPQGF	0,7136
B62	HLA-B*15:33	240	TLLALHRSY	0,6805
B62	HLA-B*15:33	258	WTAGAAAYY	0,5072
B62	HLA-B*15:33	261	GAAAYYVGY	0,52
B62	HLA-B*15:33	366	SVLYNSASF	0,5995
B62	HLA-B*15:33	413	GQTGKIADY	0,764
B62	HLA-B*15:33	497	FQPTNGVGY	0,7039
B62	HLA-B*15:33	628	QLTPTWRVY	0,6565
B62	HLA-B*15:33	634	RVYSTGSNVF	0,8491
B62	HLA-B*15:33	686	SVASQSIIAY	0,5924
B62	HLA-B*15:33	687	VASQSIIAY	0,7822
B62	HLA-B*15:33	698	SLGAENSVAY	0,6839
B62	HLA-B*15:33	880	GTITSGWTF	0,6048
B62	HLA-B*15:33	894	LQIPFAMQM	0,6403
B62	HLA-B*15:33	919	NQKLIANQF	0,7035
B62	HLA-B*15:33	962	LVKQLSSNF	0,7084
B62	HLA-B*15:33	1000	RLQSLQTY	0,5767
B62	HLA-B*15:33	1054	QSAPHGVVF	0,7211
B62	HLA-B*15:33	1113	QIITTDNTF	0,6229
B62	HLA-B*15:33	1264	VLKGVKLHY	0,8811

B62	HLA-B*15:34	35	GVYYPDKVF	0,677
B62	HLA-B*15:34	47	VLHSTQDLF	0,5635
B62	HLA-B*15:34	192	FVFKNIDGY	0,678
B62	HLA-B*15:34	212	LVRDLPQGF	0,7136
B62	HLA-B*15:34	240	TLLALHRSY	0,6805
B62	HLA-B*15:34	258	WTAGAAAYY	0,5072
B62	HLA-B*15:34	261	GAAAYYVGY	0,52
B62	HLA-B*15:34	366	SVLYNSASF	0,5995
B62	HLA-B*15:34	413	GQTGKIADY	0,764
B62	HLA-B*15:34	497	FQPTNGVGY	0,7039
B62	HLA-B*15:34	628	QLTPTWRVY	0,6565
B62	HLA-B*15:34	634	RVYSTGSNVF	0,8491
B62	HLA-B*15:34	686	SVASQSIIAY	0,5924
B62	HLA-B*15:34	687	VASQSIIAY	0,7822
B62	HLA-B*15:34	698	SLGAENSVAY	0,6839
B62	HLA-B*15:34	880	GTITSGWTF	0,6048
B62	HLA-B*15:34	894	LQIPFAMQM	0,6403
B62	HLA-B*15:34	919	NQKLIANQF	0,7035
B62	HLA-B*15:34	962	LVKQLSSNF	0,7084
B62	HLA-B*15:34	1000	RLQSLQTY	0,5767
B62	HLA-B*15:34	1054	QSAPHGVVF	0,7211
B62	HLA-B*15:34	1113	QIITTDNTF	0,6229
B62	HLA-B*15:34	1264	VLKGVKLHY	0,8811
B62	HLA-B*15:38	30	NSFTRGVYY	0,5586
B62	HLA-B*15:38	152	WMESEFRVY	0,5815
B62	HLA-B*15:38	192	FVFKNIDGY	0,6613
B62	HLA-B*15:38	212	LVRDLPQGF	0,6736
B62	HLA-B*15:38	240	TLLALHRSY	0,7304
B62	HLA-B*15:38	261	GAAAYYVGY	0,5782
B62	HLA-B*15:38	366	SVLYNSASF	0,5604
B62	HLA-B*15:38	413	GQTGKIADY	0,7293
B62	HLA-B*15:38	464	FERDISTEIY	0,5752
B62	HLA-B*15:38	497	FQPTNGVGY	0,705
B62	HLA-B*15:38	628	QLTPTWRVY	0,676
B62	HLA-B*15:38	634	RVYSTGSNVF	0,7511
B62	HLA-B*15:38	686	SVASQSIIAY	0,5671
B62	HLA-B*15:38	687	VASQSIIAY	0,7799
B62	HLA-B*15:38	689	SQSIIAYTM	0,5356
B62	HLA-B*15:38	698	SLGAENSVAY	0,6347
B62	HLA-B*15:38	699	LGAENSVAY	0,5322
B62	HLA-B*15:38	880	GTITSGWTF	0,5811
B62	HLA-B*15:38	894	LQIPFAMQM	0,6945
B62	HLA-B*15:38	919	NQKLIANQF	0,8136
B62	HLA-B*15:38	962	LVKQLSSNF	0,7271
B62	HLA-B*15:38	1000	RLQSLQTY	0,5657
B62	HLA-B*15:38	1054	QSAPHGVVF	0,711
B62	HLA-B*15:38	1113	QIITTDNTF	0,5803
B62	HLA-B*15:38	1264	VLKGVKLHY	0,8714
B62	HLA-B*15:39	35	GVYYPDKVF	0,7375
B62	HLA-B*15:39	47	VLHSTQDLF	0,5828

B62	HLA-B*15:39	83	VLPFNDGVYF	0,5256
B62	HLA-B*15:39	152	WMESEFRVY	0,5416
B62	HLA-B*15:39	192	FVFKNIDGY	0,6491
B62	HLA-B*15:39	212	LVRDLPQGF	0,6773
B62	HLA-B*15:39	240	TLLALHRSY	0,7211
B62	HLA-B*15:39	261	GAAAYYVGY	0,5543
B62	HLA-B*15:39	366	SVLYNSASF	0,6531
B62	HLA-B*15:39	413	GQTGKIADY	0,6727
B62	HLA-B*15:39	497	FQPTNGVGY	0,6539
B62	HLA-B*15:39	628	QLTPTWRVY	0,6869
B62	HLA-B*15:39	634	RVYSTGSNVF	0,8327
B62	HLA-B*15:39	686	SVASQSIIAY	0,5034
B62	HLA-B*15:39	687	VASQSIIAY	0,8337
B62	HLA-B*15:39	689	SQSIIAYTM	0,5045
B62	HLA-B*15:39	698	SLGAENSVAY	0,6247
B62	HLA-B*15:39	699	LGAENSVAY	0,5064
B62	HLA-B*15:39	815	RSFIEDLLF	0,5266
B62	HLA-B*15:39	880	GTITSGWTF	0,6421
B62	HLA-B*15:39	894	LQIPFAMQM	0,6884
B62	HLA-B*15:39	919	NQKLIANQF	0,5508
B62	HLA-B*15:39	962	LVKQLSSNF	0,5971
B62	HLA-B*15:39	1000	RLQSLQTY	0,582
B62	HLA-B*15:39	1054	QSAPHGVVF	0,7223
B62	HLA-B*15:39	1113	QIITTDNTF	0,6116
B62	HLA-B*15:39	1264	VLKGVKLHY	0,8466
B62	HLA-B*15:40	634	RVYSTGSNVF	0,6651
B62	HLA-B*15:40	687	VASQSIIAY	0,5923
B62	HLA-B*15:40	894	LQIPFAMQM	0,564
B62	HLA-B*15:40	1264	VLKGVKLHY	0,65
B62	HLA-B*15:50	192	FVFKNIDGY	0,5522
B62	HLA-B*15:50	212	LVRDLPQGF	0,5998
B62	HLA-B*15:50	240	TLLALHRSY	0,595
B62	HLA-B*15:50	413	GQTGKIADY	0,6557
B62	HLA-B*15:50	628	QLTPTWRVY	0,567
B62	HLA-B*15:50	634	RVYSTGSNVF	0,7616
B62	HLA-B*15:50	687	VASQSIIAY	0,7011
B62	HLA-B*15:50	698	SLGAENSVAY	0,5791
B62	HLA-B*15:50	880	GTITSGWTF	0,5108
B62	HLA-B*15:50	894	LQIPFAMQM	0,5936
B62	HLA-B*15:50	919	NQKLIANQF	0,635
B62	HLA-B*15:50	962	LVKQLSSNF	0,5684
B62	HLA-B*15:50	1000	RLQSLQTY	0,504
B62	HLA-B*15:50	1054	QSAPHGVVF	0,6031
B62	HLA-B*15:50	1264	VLKGVKLHY	0,8458
B62	HLA-B*15:60	35	GVYYPDKVF	0,677
B62	HLA-B*15:60	47	VLHSTQDLF	0,5635
B62	HLA-B*15:60	192	FVFKNIDGY	0,678
B62	HLA-B*15:60	212	LVRDLPQGF	0,7136
B62	HLA-B*15:60	240	TLLALHRSY	0,6805
B62	HLA-B*15:60	258	WTAGAAAYY	0,5072

B62	HLA-B*15:60	261	GAAAYYVGY	0,52
B62	HLA-B*15:60	366	SVLYNSASF	0,5995
B62	HLA-B*15:60	413	GQTGKIADY	0,764
B62	HLA-B*15:60	497	FQPTNGVGY	0,7039
B62	HLA-B*15:60	628	QLTPTWRVY	0,6565
B62	HLA-B*15:60	634	RVYSTGSNVF	0,8491
B62	HLA-B*15:60	686	SVASQSIIAY	0,5924
B62	HLA-B*15:60	687	VASQSIIAY	0,7822
B62	HLA-B*15:60	698	SLGAENSVAY	0,6839
B62	HLA-B*15:60	880	GTITSGWTF	0,6048
B62	HLA-B*15:60	894	LQIPFAMQM	0,6403
B62	HLA-B*15:60	919	NQKLIANQF	0,7035
B62	HLA-B*15:60	962	LVKQLSSNF	0,7084
B62	HLA-B*15:60	1000	RLQSLQTY	0,5767
B62	HLA-B*15:60	1054	QSAPHGVVF	0,7211
B62	HLA-B*15:60	1113	QIITTDNTF	0,6229
B62	HLA-B*15:60	1264	VLKGVKLHY	0,8811
B62	HLA-B*15:65	35	GVYYPDKVF	0,5822
B62	HLA-B*15:65	47	VLHSTQDLF	0,6073
B62	HLA-B*15:65	152	WMESEFRVY	0,5864
B62	HLA-B*15:65	192	FVFKNIDGY	0,5038
B62	HLA-B*15:65	212	LVRDLPQGF	0,5359
B62	HLA-B*15:65	240	TLLALHRSY	0,6159
B62	HLA-B*15:65	366	SVLYNSASF	0,6187
B62	HLA-B*15:65	584	ILDITPCSF	0,5073
B62	HLA-B*15:65	628	QLTPTWRVY	0,6602
B62	HLA-B*15:65	634	RVYSTGSNVF	0,7599
B62	HLA-B*15:65	686	SVASQSIIAY	0,5527
B62	HLA-B*15:65	687	VASQSIIAY	0,792
B62	HLA-B*15:65	698	SLGAENSVAY	0,6847
B62	HLA-B*15:65	880	GTITSGWTF	0,5709
B62	HLA-B*15:65	1000	RLQSLQTY	0,5594
B62	HLA-B*15:65	1054	QSAPHGVVF	0,7159
B62	HLA-B*15:65	1113	QIITTDNTF	0,5676
B62	HLA-B*15:65	1264	VLKGVKLHY	0,7435
B62	HLA-B*15:70	192	FVFKNIDGY	0,5037
B62	HLA-B*15:70	212	LVRDLPQGF	0,5805
B62	HLA-B*15:70	413	GQTGKIADY	0,5593
B62	HLA-B*15:70	634	RVYSTGSNVF	0,6634
B62	HLA-B*15:70	687	VASQSIIAY	0,563
B62	HLA-B*15:70	962	LVKQLSSNF	0,5026
B62	HLA-B*15:70	1054	QSAPHGVVF	0,5214
B62	HLA-B*15:70	1264	VLKGVKLHY	0,7409
B62	HLA-B*15:75	35	GVYYPDKVF	0,6355
B62	HLA-B*15:75	47	VLHSTQDLF	0,524
B62	HLA-B*15:75	192	FVFKNIDGY	0,652
B62	HLA-B*15:75	212	LVRDLPQGF	0,6707
B62	HLA-B*15:75	240	TLLALHRSY	0,6492
B62	HLA-B*15:75	366	SVLYNSASF	0,5638
B62	HLA-B*15:75	413	GQTGKIADY	0,7207

B62	HLA-B*15:75	497	FQPTNGVGY	0,656
B62	HLA-B*15:75	628	QLTPTWRVY	0,6233
B62	HLA-B*15:75	634	RVYSTGSNVF	0,8275
B62	HLA-B*15:75	686	SVASQSIIAY	0,5578
B62	HLA-B*15:75	687	VASQSIIAY	0,7575
B62	HLA-B*15:75	698	SLGAENSVAY	0,6521
B62	HLA-B*15:75	880	GTITSGWTF	0,5821
B62	HLA-B*15:75	894	LQIPFAMQM	0,6094
B62	HLA-B*15:75	919	NQKLIANQF	0,6621
B62	HLA-B*15:75	962	LVKQLSSNF	0,6585
B62	HLA-B*15:75	1000	RLQSLQTY	0,533
B62	HLA-B*15:75	1054	QSAPHGVVF	0,6833
B62	HLA-B*15:75	1113	QIITTDNTF	0,5867
B62	HLA-B*15:75	1264	VLKGVKLHY	0,8622
B62	HLA-B*15:78	35	GVYYPDKVF	0,677
B62	HLA-B*15:78	47	VLHSTQDLF	0,5635
B62	HLA-B*15:78	192	FVFKNIDGY	0,678
B62	HLA-B*15:78	212	LVRDLPQGF	0,7136
B62	HLA-B*15:78	240	TLLALHRSY	0,6805
B62	HLA-B*15:78	258	WTAGAAAYY	0,5072
B62	HLA-B*15:78	261	GAAAYYVGY	0,52
B62	HLA-B*15:78	366	SVLYNSASF	0,5995
B62	HLA-B*15:78	413	GQTGKIADY	0,764
B62	HLA-B*15:78	497	FQPTNGVGY	0,7039
B62	HLA-B*15:78	628	QLTPTWRVY	0,6565
B62	HLA-B*15:78	634	RVYSTGSNVF	0,8491
B62	HLA-B*15:78	686	SVASQSIIAY	0,5924
B62	HLA-B*15:78	687	VASQSIIAY	0,7822
B62	HLA-B*15:78	698	SLGAENSVAY	0,6839
B62	HLA-B*15:78	880	GTITSGWTF	0,6048
B62	HLA-B*15:78	894	LQIPFAMQM	0,6403
B62	HLA-B*15:78	919	NQKLIANQF	0,7035
B62	HLA-B*15:78	962	LVKQLSSNF	0,7084
B62	HLA-B*15:78	1000	RLQSLQTY	0,5767
B62	HLA-B*15:78	1054	QSAPHGVVF	0,7211
B62	HLA-B*15:78	1113	QIITTDNTF	0,6229
B62	HLA-B*15:78	1264	VLKGVKLHY	0,8811
B62	HLA-B*15:81	35	GVYYPDKVF	0,677
B62	HLA-B*15:81	47	VLHSTQDLF	0,5635
B62	HLA-B*15:81	192	FVFKNIDGY	0,678
B62	HLA-B*15:81	212	LVRDLPQGF	0,7136
B62	HLA-B*15:81	240	TLLALHRSY	0,6805
B62	HLA-B*15:81	258	WTAGAAAYY	0,5072
B62	HLA-B*15:81	261	GAAAYYVGY	0,52
B62	HLA-B*15:81	366	SVLYNSASF	0,5995
B62	HLA-B*15:81	413	GQTGKIADY	0,764
B62	HLA-B*15:81	497	FQPTNGVGY	0,7039
B62	HLA-B*15:81	628	QLTPTWRVY	0,6565
B62	HLA-B*15:81	634	RVYSTGSNVF	0,8491
B62	HLA-B*15:81	686	SVASQSIIAY	0,5924

B62	HLA-B*15:81	687	VASQSIIAY	0,7822
B62	HLA-B*15:81	698	SLGAENSVAY	0,6839
B62	HLA-B*15:81	880	GTITSGWTF	0,6048
B62	HLA-B*15:81	894	LQIPFAMQM	0,6403
B62	HLA-B*15:81	919	NQKLIANQF	0,7035
B62	HLA-B*15:81	962	LVKQLSSNF	0,7084
B62	HLA-B*15:81	1000	RLQSLQTY	0,5767
B62	HLA-B*15:81	1054	QSAPHGVVF	0,7211
B62	HLA-B*15:81	1113	QIITTDNTF	0,6229
B62	HLA-B*15:81	1264	VLKGVKLHY	0,8811
B62	HLA-B*15:82	35	GVYYPDKVF	0,677
B62	HLA-B*15:82	47	VLHSTQDLF	0,5635
B62	HLA-B*15:82	192	FVFKNIDGY	0,678
B62	HLA-B*15:82	212	LVRDLPQGF	0,7136
B62	HLA-B*15:82	240	TLLALHRSY	0,6805
B62	HLA-B*15:82	258	WTAGAAAYY	0,5072
B62	HLA-B*15:82	261	GAAAYYVGY	0,52
B62	HLA-B*15:82	366	SVLYNSASF	0,5995
B62	HLA-B*15:82	413	GQTGKIADY	0,764
B62	HLA-B*15:82	497	FQPTNGVGY	0,7039
B62	HLA-B*15:82	628	QLTPTWRVY	0,6565
B62	HLA-B*15:82	634	RVYSTGSNVF	0,8491
B62	HLA-B*15:82	686	SVASQSIIAY	0,5924
B62	HLA-B*15:82	687	VASQSIIAY	0,7822
B62	HLA-B*15:82	698	SLGAENSVAY	0,6839
B62	HLA-B*15:82	880	GTITSGWTF	0,6048
B62	HLA-B*15:82	894	LQIPFAMQM	0,6403
B62	HLA-B*15:82	919	NQKLIANQF	0,7035
B62	HLA-B*15:82	962	LVKQLSSNF	0,7084
B62	HLA-B*15:82	1000	RLQSLQTY	0,5767
B62	HLA-B*15:82	1054	QSAPHGVVF	0,7211
B62	HLA-B*15:82	1113	QIITTDNTF	0,6229
B62	HLA-B*15:82	1264	VLKGVKLHY	0,8811
B62	HLA-B*15:85	35	GVYYPDKVF	0,6525
B62	HLA-B*15:85	47	VLHSTQDLF	0,5234
B62	HLA-B*15:85	192	FVFKNIDGY	0,6835
B62	HLA-B*15:85	212	LVRDLPQGF	0,6903
B62	HLA-B*15:85	240	TLLALHRSY	0,6924
B62	HLA-B*15:85	258	WTAGAAAYY	0,5057
B62	HLA-B*15:85	261	GAAAYYVGY	0,5182
B62	HLA-B*15:85	366	SVLYNSASF	0,5905
B62	HLA-B*15:85	413	GQTGKIADY	0,7286
B62	HLA-B*15:85	497	FQPTNGVGY	0,6701
B62	HLA-B*15:85	628	QLTPTWRVY	0,6639
B62	HLA-B*15:85	634	RVYSTGSNVF	0,8172
B62	HLA-B*15:85	686	SVASQSIIAY	0,6057
B62	HLA-B*15:85	687	VASQSIIAY	0,7768
B62	HLA-B*15:85	698	SLGAENSVAY	0,6849
B62	HLA-B*15:85	880	GTITSGWTF	0,5734
B62	HLA-B*15:85	894	LQIPFAMQM	0,5976

B62	HLA-B*15:85	919	NQKLIANQF	0,6783
B62	HLA-B*15:85	962	LVKQLSSNF	0,6864
B62	HLA-B*15:85	1000	RLQSLQTY	0,5531
B62	HLA-B*15:85	1054	QSAPHGVVF	0,6958
B62	HLA-B*15:85	1113	QIITTDNTF	0,608
B62	HLA-B*15:85	1264	VLKGVKLHY	0,8756
B62	HLA-B*15:88	84	LPFNDGVYF	0,5189
B62	HLA-B*15:88	192	FVFKNIDGY	0,5876
B62	HLA-B*15:88	240	TLLALHRSY	0,5087
B62	HLA-B*15:88	628	QLTPTWRVY	0,53
B62	HLA-B*15:88	687	VASQSIIAY	0,7704
B62	HLA-B*15:88	698	SLGAENSVAY	0,5239
B62	HLA-B*15:88	699	LGAENSVAY	0,5042
B62	HLA-B*15:88	896	IPFAMQMAY	0,6381
B62	HLA-B*15:88	1054	QSAPHGVVF	0,5587
B62	HLA-B*15:88	1113	QIITTDNTF	0,5554
B62	HLA-B*15:92	35	GVYYPDKVF	0,677
B62	HLA-B*15:92	47	VLHSTQDLF	0,5635
B62	HLA-B*15:92	192	FVFKNIDGY	0,678
B62	HLA-B*15:92	212	LVRDLPQGF	0,7136
B62	HLA-B*15:92	240	TLLALHRSY	0,6805
B62	HLA-B*15:92	258	WTAGAAAYY	0,5072
B62	HLA-B*15:92	261	GAAAYYVGY	0,52
B62	HLA-B*15:92	366	SVLYNSASF	0,5995
B62	HLA-B*15:92	413	GQTGKIADY	0,764
B62	HLA-B*15:92	497	FQPTNGVGY	0,7039
B62	HLA-B*15:92	628	QLTPTWRVY	0,6565
B62	HLA-B*15:92	634	RVYSTGSNVF	0,8491
B62	HLA-B*15:92	686	SVASQSIIAY	0,5924
B62	HLA-B*15:92	687	VASQSIIAY	0,7822
B62	HLA-B*15:92	698	SLGAENSVAY	0,6839
B62	HLA-B*15:92	880	GTITSGWTF	0,6048
B62	HLA-B*15:92	894	LQIPFAMQM	0,6403
B62	HLA-B*15:92	919	NQKLIANQF	0,7035
B62	HLA-B*15:92	962	LVKQLSSNF	0,7084
B62	HLA-B*15:92	1000	RLQSLQTY	0,5767
B62	HLA-B*15:92	1054	QSAPHGVVF	0,7211
B62	HLA-B*15:92	1113	QIITTDNTF	0,6229
B62	HLA-B*15:92	1264	VLKGVKLHY	0,8811
B62	HLA-B*15:96	35	GVYYPDKVF	0,677
B62	HLA-B*15:96	47	VLHSTQDLF	0,5635
B62	HLA-B*15:96	192	FVFKNIDGY	0,678
B62	HLA-B*15:96	212	LVRDLPQGF	0,7136
B62	HLA-B*15:96	240	TLLALHRSY	0,6805
B62	HLA-B*15:96	258	WTAGAAAYY	0,5072
B62	HLA-B*15:96	261	GAAAYYVGY	0,52
B62	HLA-B*15:96	366	SVLYNSASF	0,5995
B62	HLA-B*15:96	413	GQTGKIADY	0,764
B62	HLA-B*15:96	497	FQPTNGVGY	0,7039
B62	HLA-B*15:96	628	QLTPTWRVY	0,6565

B62	HLA-B*15:96	634	RVYSTGSNVF	0,8491
B62	HLA-B*15:96	686	SVASQSIIAY	0,5924
B62	HLA-B*15:96	687	VASQSIIAY	0,7822
B62	HLA-B*15:96	698	SLGAENSVAY	0,6839
B62	HLA-B*15:96	880	GTITSGWTF	0,6048
B62	HLA-B*15:96	894	LQIPFAMQM	0,6403
B62	HLA-B*15:96	919	NQKLIANQF	0,7035
B62	HLA-B*15:96	962	LVKQLSSNF	0,7084
B62	HLA-B*15:96	1000	RLQSLQTY	0,5767
B62	HLA-B*15:96	1054	QSAPHGVVF	0,7211
B62	HLA-B*15:96	1113	QIITTDNTF	0,6229
B62	HLA-B*15:96	1264	VLKGVKLHY	0,8811
B62	HLA-B*15:97	35	GVYYPDKVF	0,677
B62	HLA-B*15:97	47	VLHSTQDLF	0,5635
B62	HLA-B*15:97	192	FVFKNIDGY	0,678
B62	HLA-B*15:97	212	LVRDLPQGF	0,7136
B62	HLA-B*15:97	240	TLLALHRSY	0,6805
B62	HLA-B*15:97	258	WTAGAAAYY	0,5072
B62	HLA-B*15:97	261	GAAAYYVGY	0,52
B62	HLA-B*15:97	366	SVLYNSASF	0,5995
B62	HLA-B*15:97	413	GQTGKIADY	0,764
B62	HLA-B*15:97	497	FQPTNGVGY	0,7039
B62	HLA-B*15:97	628	QLTPTWRVY	0,6565
B62	HLA-B*15:97	634	RVYSTGSNVF	0,8491
B62	HLA-B*15:97	686	SVASQSIIAY	0,5924
B62	HLA-B*15:97	687	VASQSIIAY	0,7822
B62	HLA-B*15:97	698	SLGAENSVAY	0,6839
B62	HLA-B*15:97	880	GTITSGWTF	0,6048
B62	HLA-B*15:97	894	LQIPFAMQM	0,6403
B62	HLA-B*15:97	919	NQKLIANQF	0,7035
B62	HLA-B*15:97	962	LVKQLSSNF	0,7084
B62	HLA-B*15:97	1000	RLQSLQTY	0,5767
B62	HLA-B*15:97	1054	QSAPHGVVF	0,7211
B62	HLA-B*15:97	1113	QIITTDNTF	0,6229
B62	HLA-B*15:97	1264	VLKGVKLHY	0,8811
B62	HLA-B*35:28	192	FVFKNIDGY	0,6199
B62	HLA-B*35:28	240	TLLALHRSY	0,5051
B62	HLA-B*35:28	261	GAAAYYVGY	0,5209
B62	HLA-B*35:28	339	GEVFNATRF	0,6317
B62	HLA-B*35:28	413	GQTGKIADY	0,5514
B62	HLA-B*35:28	443	SKVGGNYNY	0,6002
B62	HLA-B*35:28	464	FERDISTEIY	0,5471
B62	HLA-B*35:28	497	FQPTNGVGY	0,6463
B62	HLA-B*35:28	687	VASQSIIAY	0,7882
B62	HLA-B*35:28	689	SQSIIAYTM	0,6456
B62	HLA-B*35:28	699	LGAENSVAY	0,546
B62	HLA-B*35:28	815	RSFIEDLLF	0,5236
B62	HLA-B*35:28	880	GTITSGWTF	0,5709
B62	HLA-B*35:28	894	LQIPFAMQM	0,7173
B62	HLA-B*35:28	919	NQKLIANQF	0,5854

B62	HLA-B*35:28	1054	QSAPHGVVF	0,6134
B62	HLA-B*35:28	1113	QIITTDNTF	0,5096
B62	HLA-B*52:01	712	IAIPTNFTI	0,7144
B62	HLA-B*52:02	712	IAIPTNFTI	0,7268
B62	HLA-B*52:03	712	IAIPTNFTI	0,7225
B62	HLA-B*52:04	712	IAIPTNFTI	0,7144
B62	HLA-B*52:05	712	IAIPTNFTI	0,7144
B62	HLA-B*52:07	712	IAIPTNFTI	0,7144

Note: The epitopes conserved among Khosta-2 and SARS-CoV-2 are indicated in red
