# Supplementary material for: Conservation of HLA Spike Protein Epitopes Supports T Cell Cross-Protection in SARS-CoV-2 Vaccinated Individuals against the Potentially Zoonotic Coronavirus Khosta-2
Source: Int J Mol Sci. 2024 May 31;25(11):6087. doi: 10.3390/ijms25116087 (PMC11172828; doi:10.3390/ijms25116087)
Supplement: Supplementary file 1 [file ijms-25-06087-s001.zip › Supplemental Table 3.pdf]

Supplemental Table 3

HLA-C01:02

Pos	ALLELE	Peptide	Score_EL
1055	HLA-C*01:02	SAPHGVVFL	0.9666610
505	HLA-C*01:02	YQPYRVVVL	0.8731390
382	HLA-C*01:02	VSPTKLNDL	0.8376700
215	HLA-C*01:02	DLPQGFSAL	0.6968300
109	HLA-C*01:02	TLDSKTQSL	0.5960200
525	HLA-C*01:02	CGPKKSTNL	0.5443170
214	HLA-C*01:02	RDLPQGFSAL	0.5212360
269	HLA-C*01:02	YLQPRTFLL	0.5189250
410	HLA-C*01:02	IAPGQTGKI	0.5153670
1137	HLA-C*01:02	VYDPLQPEL	0.4161170
213	HLA-C*01:02	VRDLPQGFSAL	0.4148090
951	HLA-C*01:02	VVNQNAQAL	0.3742980
860	HLA-C*01:02	VLPPLLTDEM	0.3736260
679	HLA-C*01:02	NSPRRARSV	0.3523060
1067	HLA-C*01:02	YVPAQEKNF	0.3389740
23	HLA-C*01:02	QLPPAYTNSF	0.2985700
869	HLA-C*01:02	MIAQYTSAL	0.2683710
937	HLA-C*01:02	SLSSTASAL	0.2588260
1054	HLA-C*01:02	QSAPHGVVFL	0.2440060
204	HLA-C*01:02	YSKHTPINL	0.2375180
691	HLA-C*01:02	SIIAYTMSL	0.2244470
433	HLA-C*01:02	VIAWNSNNL	0.2141610
976	HLA-C*01:02	VLNDILSRL	0.1983210
24	HLA-C*01:02	LPPAYTNSF	0.1967130
870	HLA-C*01:02	IAQYTSALL	0.1923890
523	HLA-C*01:02	TVCGPKKSTNL	0.1907930
524	HLA-C*01:02	VCGPKKSTNL	0.1870380

HLA-C*02:02

Pos	ALLELE	Peptide	Score_EL
687	HLA-C*02:02	VASQSIIAY	0.8367210
1054	HLA-C*02:02	QSAPHGVVF	0.7402120
718	HLA-C*02:02	FTISVTTEI	0.6044010
1095	HLA-C*02:02	FVSNGTHWF	0.5905600
898	HLA-C*02:02	FAMQMAYRF	0.5782790
30	HLA-C*02:02	NSFTRGVYY	0.5773140
204	HLA-C*02:02	YSKHTPINL	0.5668790
604	HLA-C*02:02	TSNQVAVLY	0.5185190
212	HLA-C*02:02	LVRDLPQGF	0.4939540
894	HLA-C*02:02	LQIPFAMQM	0.4869870
710	HLA-C*02:02	NSIAIPTNF	0.4774030
162	HLA-C*02:02	SANNCTFEY	0.4683060
192	HLA-C*02:02	FVFKNIDGY	0.4590910
865	HLA-C*02:02	LTDEMIAQY	0.4234830
1052	HLA-C*02:02	FPQSAPHGVVF	0.4065470
1021	HLA-C*02:02	SANLAATKM	0.3781410
712	HLA-C*02:02	IAIPTNFTI	0.3543880
160	HLA-C*02:02	YSSANNCTF	0.3415160
962	HLA-C*02:02	LVKQLSSNF	0.3394020
258	HLA-C*02:02	WTAGAAAYY	0.3299060
372	HLA-C*02:02	ASFSTFKCY	0.3294680
976	HLA-C*02:02	VLNDILSRL	0.3270210
269	HLA-C*02:02	YLQPRTFLL	0.3141330
392	HLA-C*02:02	FTNVYADSF	0.2998190
361	HLA-C*02:02	CVADYSVLY	0.2956830
1060	HLA-C*02:02	VVFLHVTYV	0.2931170
892	HLA-C*02:02	AALQIPFAM	0.2925790
1264	HLA-C*02:02	VLKGVKLHY	0.2916610
267	HLA-C*02:02	VGYLQPRTF	0.2887620
343	HLA-C*02:02	NATRFASVY	0.2758410

1055	HLA-C*02:02	SAPHGVVFL	0.2719190
1189	HLA-C*02:02	VAKNLNESL	0.2599260
28	HLA-C*02:02	YTNSFTRGV	0.2496170
50	HLA-C*02:02	STQDLFLPF	0.2427770
625	HLA-C*02:02	HADQLTPTW	0.2351690
505	HLA-C*02:02	YQPYRVVVL	0.2332730
28	HLA-C*02:02	YTNSFTRGVY	0.2262420
261	HLA-C*02:02	GAAAYYVGY	0.2235930
951	HLA-C*02:02	VVNQNAQAL	0.2227700

HLA-C03:03

Pos	ALLELE	Peptide	Score_EL
951	HLA-C*03:03	VVNQNAQAL	0.7995950
221	HLA-C*03:03	SALEPLVDL	0.7647860
1054	HLA-C*03:03	QSAPHGVVF	0.7121150
712	HLA-C*03:03	IAIPTNFTI	0.6853900
204	HLA-C*03:03	YSKHTPINL	0.6720250
886	HLA-C*03:03	WTFGAGAAL	0.6281400
718	HLA-C*03:03	FTISVTTEI	0.5996190
1021	HLA-C*03:03	SANLAATKM	0.5732640
892	HLA-C*03:03	AALQIPFAM	0.5659050
870	HLA-C*03:03	IAQYTSALL	0.5617850
869	HLA-C*03:03	MIAQYTSAL	0.5568900
1189	HLA-C*03:03	VAKNLNESL	0.5519470
923	HLA-C*03:03	IANQFNSAI	0.5163940
1055	HLA-C*03:03	SAPHGVVFL	0.4879120
691	HLA-C*03:03	SIIAYTMSL	0.4311350
898	HLA-C*03:03	FAMQMAYRF	0.4075460
109	HLA-C*03:03	TLDSKTQSL	0.3813330
269	HLA-C*03:03	YLQPRTFLL	0.3793590
929	HLA-C*03:03	SAIGKIQDSL	0.3737030
160	HLA-C*03:03	YSSANNCTF	0.3576430
759	HLA-C*03:03	FCTQLNRAL	0.3494590
1136	HLA-C*03:03	TVYDPLQPEL	0.2994230
894	HLA-C*03:03	LQIPFAMQM	0.2991940
505	HLA-C*03:03	YQPYRVVVL	0.2990200
1137	HLA-C*03:03	VYDPLQPEL	0.2815730
1052	HLA-C*03:03	FPQSAPHGVVF	0.2717310
1225	HLA-C*03:03	IAIVMVTIM	0.2558520
1095	HLA-C*03:03	FVSNGTHWF	0.2486660
996	HLA-C*03:03	LITGRLQSL	0.2376540
262	HLA-C*03:03	AAAYYVGYL	0.2354450

HLA-C03:04

Pos	ALLELE	Peptide	Score_EL
951	HLA-C*03:04	VVNQNAQAL	0.7995950
221	HLA-C*03:04	SALEPLVDL	0.7647860
1054	HLA-C*03:04	QSAPHGVVF	0.7121150
712	HLA-C*03:04	IAIPTNFTI	0.6853900
204	HLA-C*03:04	YSKHTPINL	0.6720250
886	HLA-C*03:04	WTFGAGAAL	0.6281400
718	HLA-C*03:04	FTISVTTEI	0.5996190
1021	HLA-C*03:04	SANLAATKM	0.5732640
892	HLA-C*03:04	AALQIPFAM	0.5659050
870	HLA-C*03:04	IAQYTSALL	0.5617850
869	HLA-C*03:04	MIAQYTSAL	0.5568900
1189	HLA-C*03:04	VAKNLNESL	0.5519470
923	HLA-C*03:04	IANQFNSAI	0.5163940
1055	HLA-C*03:04	SAPHGVVFL	0.4879120
691	HLA-C*03:04	SIIAYTMSL	0.4311350

898	HLA-C*03:04	FAMQMAYRF	0.4075460
109	HLA-C*03:04	TLDSKTQSL	0.3813330
269	HLA-C*03:04	YLQPRTFLL	0.3793590
929	HLA-C*03:04	SAIGKIQDSL	0.3737030
160	HLA-C*03:04	YSSANNCTF	0.3576430
759	HLA-C*03:04	FCTQLNRAL	0.3494590
1136	HLA-C*03:04	TVYDPLQPEL	0.2994230
894	HLA-C*03:04	LQIPFAMQM	0.2991940
505	HLA-C*03:04	YQPYRVVVL	0.2990200
1137	HLA-C*03:04	VYDPLQPEL	0.2815730
1052	HLA-C*03:04	FPQSAPHGVVF	0.2717310
1225	HLA-C*03:04	IAIVMVTIM	0.2558520
1095	HLA-C*03:04	FVSNGTHWF	0.2486660
996	HLA-C*03:04	LITGRLQSL	0.2376540
262	HLA-C*03:04	AAAYYVGYL	0.2354450

HLA-C04:01

Pos	ALLELE	Peptide	Score_EL
1137	HLA-C*04:01	VYDPLQPEL	0.9890100
78	HLA-C*04:01	RFDNPVLPF	0.8293790
109	HLA-C*04:01	TLDSKTQSL	0.7862160
1136	HLA-C*04:01	TVYDPLQPEL	0.5944180
584	HLA-C*04:01	ILDITPCSF	0.5314790
576	HLA-C*04:01	VRDPQTLEI	0.4066310
269	HLA-C*04:01	YLQPRTFLL	0.3954270
1255	HLA-C*04:01	KFDEDDSEPVL	0.3477740
1135	HLA-C*04:01	NTVYDPLQPEL	0.3302140
1137	HLA-C*04:01	VYDPLQPELD	0.2535060
1144	HLA-C*04:01	ELDSFKEEL	0.2395180
1109	HLA-C*04:01	FYEPQIITT	0.2119110
167	HLA-C*04:01	TFEYVSQPF	0.2057660
976	HLA-C*04:01	VLNDILSRL	0.2050430
612	HLA-C*04:01	YQDVNCTEV	0.1977010
625	HLA-C*04:01	HADQLTPTW	0.1921350
489	HLA-C*04:01	YFPLQSYGF	0.1725160
635	HLA-C*04:01	VYSTGSNVF	0.1638710
77	HLA-C*04:01	KRFDNPVLPF	0.1535710
38	HLA-C*04:01	YPDKVFRSSVL	0.1526820
51	HLA-C*04:01	TQDLFLPFF	0.1443490
321	HLA-C*04:01	QPTESIVRF	0.1396060
865	HLA-C*04:01	LTDEMIAQY	0.1332980
983	HLA-C*04:01	RLDKVEAEV	0.1321000
1052	HLA-C*04:01	FPQSAPHGV	0.1277050
417	HLA-C*04:01	KIADYNYKL	0.1226590

HLA-C05:01

Pos	ALLELE	Peptide	Score_EL
109	HLA-C*05:01	TLDSKTQSL	0.9771740
584	HLA-C*05:01	ILDITPCSF	0.8135600
983	HLA-C*05:01	RLDKVEAEV	0.7846700
1137	HLA-C*05:01	VYDPLQPEL	0.7347040
285	HLA-C*05:01	ITDAVDCAL	0.7082610
612	HLA-C*05:01	YQDVNCTEV	0.6348130
269	HLA-C*05:01	YLQPRTFLL	0.6032370
108	HLA-C*05:01	TTLDSKTQSL	0.5593770
1136	HLA-C*05:01	TVYDPLQPEL	0.5513400
625	HLA-C*05:01	HADQLTPTW	0.5479000
109	HLA-C*05:01	TLDSKTQSLL	0.5384810
1144	HLA-C*05:01	ELDSFKEEL	0.5189920

575	HLA-C*05:01	AVRDPQTLEI	0.5157620
865	HLA-C*05:01	LTDEMIAQY	0.4319490
51	HLA-C*05:01	TQDLFLPFF	0.4234120
572	HLA-C*05:01	TTDAVRDPQTL	0.4025970
976	HLA-C*05:01	VLNDILSRL	0.3933470
773	HLA-C*05:01	EQDKNTQEV	0.3851750
78	HLA-C*05:01	RFDNPVLPF	0.3425920
841	HLA-C*05:01	LGDIAARDL	0.3154370
417	HLA-C*05:01	KIADYNYKL	0.3137220
576	HLA-C*05:01	VRDPQTLEI	0.2978880
723	HLA-C*05:01	TTEILPVSM	0.2754910

HLA-C06:02

Pos	ALLELE	Peptide	Score_EL
327	HLA-C*06:02	VRFPNITNL	0.9755830
402	HLA-C*06:02	IRGDEVRQI	0.8345460
236	HLA-C*06:02	TRFQTLLAL	0.7989610
318	HLA-C*06:02	FRVQPTESI	0.6729480
576	HLA-C*06:02	VRDPQTLEI	0.5813300
453	HLA-C*06:02	YRLFRKSNL	0.5742470
77	HLA-C*06:02	KRFDNPVLPF	0.4117640
456	HLA-C*06:02	FRKSNLKPF	0.4084910
505	HLA-C*06:02	YQPYRVVVL	0.4061410
789	HLA-C*06:02	YKTPPIKDF	0.3863800
20	HLA-C*06:02	TRTQLPPAY	0.3733780
326	HLA-C*06:02	IVRFPNITNL	0.3623830
28	HLA-C*06:02	YTNSFTRGV	0.3323260
269	HLA-C*06:02	YLQPRTFLL	0.3280120
684	HLA-C*06:02	ARSVASQSI	0.3213900
205	HLA-C*06:02	SKHTPINLV	0.3083280
204	HLA-C*06:02	YSKHTPINL	0.2895340
43	HLA-C*06:02	FRSSVLHST	0.2664440
999	HLA-C*06:02	GRLQSLQTY	0.2585500
325	HLA-C*06:02	SIVRFPNITNL	0.2449380
1106	HLA-C*06:02	QRNFYEPQI	0.2423860
894	HLA-C*06:02	LQIPFAMQM	0.2348460
1055	HLA-C*06:02	SAPHGVVFL	0.2346700
718	HLA-C*06:02	FTISVTTEI	0.2338950
345	HLA-C*06:02	TRFASVYAW	0.2309230
1137	HLA-C*06:02	VYDPLQPEL	0.2298050
557	HLA-C*06:02	KKFLPFQQF	0.2202210
267	HLA-C*06:02	VGYLQPRTF	0.2094980
30	HLA-C*06:02	NSFTRGVYY	0.2066080
1060	HLA-C*06:02	VVFLHVTYV	0.1764500
503	HLA-C*06:02	VGYQPYRVV	0.1723020
1208	HLA-C*06:02	QYIKWPWYI	0.1717120

HLA-C07:01

Pos	ALLELE	Peptide	Score_EL
327	HLA-C*07:01	VRFPNITNL	0.8666960
236	HLA-C*07:01	TRFQTLLAL	0.6120630
20	HLA-C*07:01	TRTQLPPAY	0.4734090
576	HLA-C*07:01	VRDPQTLEI	0.4719160
1137	HLA-C*07:01	VYDPLQPEL	0.4702010
77	HLA-C*07:01	KRFDNPVLPF	0.4667750
999	HLA-C*07:01	GRLQSLQTY	0.4316250
318	HLA-C*07:01	FRVQPTESI	0.2742130
402	HLA-C*07:01	IRGDEVRQI	0.2296020
453	HLA-C*07:01	YRLFRKSNL	0.2212920
269	HLA-C*07:01	YLQPRTFLL	0.1816100

456	HLA-C*07:01	FRKSNLKPF	0.1762480
345	HLA-C*07:01	TRFASVYAW	0.1651620
789	HLA-C*07:01	YKTPPIKDF	0.1572890
30	HLA-C*07:01	NSFTRGVYY	0.1443930
557	HLA-C*07:01	KKFLPFQQF	0.1326580
325	HLA-C*07:01	SIVRFPNITNL	0.1035180
687	HLA-C*07:01	VASQSIIAY	0.1013260
1054	HLA-C*07:01	QSAPHGVVF	0.0947600
505	HLA-C*07:01	YQPYRVVVL	0.0947460
576	HLA-C*07:01	VRDPQTLEIL	0.0931460
326	HLA-C*07:01	IVRFPNITNL	0.0905020
448	HLA-C*07:01	NYNYLYRLF	0.0821690
814	HLA-C*07:01	KRSFIEDLL	0.0811000
204	HLA-C*07:01	YSKHTPINL	0.0785890
635	HLA-C*07:01	VYSTGSNVF	0.0775030
604	HLA-C*07:01	TSNQVAVLY	0.0769270
684	HLA-C*07:01	ARSVASQSI	0.0733560
781	HLA-C*07:01	VFAQVKQIY	0.0719540
1087	HLA-C*07:01	AHFPREGVF	0.0691880
1208	HLA-C*07:01	QYIKWPWYI	0.0666490

HLA-C07:02

Pos	ALLELE	Peptide	Score_EL
1137	HLA-C*07:02	VYDPLQPEL	0.8713540
327	HLA-C*07:02	VRFPNITNL	0.8433440
20	HLA-C*07:02	TRTQLPPAY	0.6510680
236	HLA-C*07:02	TRFQTLLAL	0.5824180
999	HLA-C*07:02	GRLQSLQTY	0.5372510
576	HLA-C*07:02	VRDPQTLEI	0.4786820
635	HLA-C*07:02	VYSTGSNVF	0.4713910
505	HLA-C*07:02	YQPYRVVVL	0.3771270
77	HLA-C*07:02	KRFDNPVLPF	0.3644700
269	HLA-C*07:02	YLQPRTFLL	0.3380540
448	HLA-C*07:02	NYNYLYRLF	0.3174210
318	HLA-C*07:02	FRVQPTESI	0.3134040
78	HLA-C*07:02	RFDNPVLPF	0.2786520
456	HLA-C*07:02	FRKSNLKPF	0.2779230
789	HLA-C*07:02	YKTPPIKDF	0.2653460
265	HLA-C*07:02	YYVGYLQPRTF	0.2573370
144	HLA-C*07:02	YYHKNNKSW	0.2100920
781	HLA-C*07:02	VFAQVKQIY	0.2084450
453	HLA-C*07:02	YRLFRKSNL	0.2021140
169	HLA-C*07:02	EYVSQPFLM	0.1863110
268	HLA-C*07:02	GYLQPRTFL	0.1814490
489	HLA-C*07:02	YFPLQSYGF	0.1811300
1087	HLA-C*07:02	AHFPREGVF	0.1718990
557	HLA-C*07:02	KKFLPFQQF	0.1578890
1208	HLA-C*07:02	QYIKWPWYI	0.1505290
345	HLA-C*07:02	TRFASVYAW	0.1492090
576	HLA-C*07:02	VRDPQTLEIL	0.1458630
497	HLA-C*07:02	FQPTNGVGY	0.1308060
186	HLA-C*07:02	FKNLREFVF	0.1273710
402	HLA-C*07:02	IRGDEVRQI	0.1259990
894	HLA-C*07:02	LQIPFAMQM	0.1258130
109	HLA-C*07:02	TLDSKTQSL	0.1250860
1136	HLA-C*07:02	TVYDPLQPEL	0.1229770
788	HLA-C*07:02	IYKTPPIKDF	0.1202020

HLA-C08:01

Pos	ALLELE	Peptide	Score_EL
109	HLA-C*08:01	TLDSKTQSL	0.5658800

1137	HLA-C*08:01	VYDPLQPEL	0.4418680
221	HLA-C*08:01	SALEPLVDL	0.3799280
712	HLA-C*08:01	IAIPTNFTI	0.3129130
269	HLA-C*08:01	YLQPRTFLL	0.2805940
285	HLA-C*08:01	ITDAVDCAL	0.2784720
951	HLA-C*08:01	VVNQNAQAL	0.2467780
625	HLA-C*08:01	HADQLTPTW	0.2426230
718	HLA-C*08:01	FTISVTTEI	0.2425530
1055	HLA-C*08:01	SAPHGVVFL	0.2354820
1054	HLA-C*08:01	QSAPHGVVF	0.2199890
612	HLA-C*08:01	YQDVNCTEV	0.2198090
1021	HLA-C*08:01	SANLAATKM	0.2048310
584	HLA-C*08:01	ILDITPCSF	0.1903110
923	HLA-C*08:01	IANQFNSAI	0.1752770
892	HLA-C*08:01	AALQIPFAM	0.1741640
417	HLA-C*08:01	KIADYNYKL	0.1441640
505	HLA-C*08:01	YQPYRVVVL	0.1425360
870	HLA-C*08:01	IAQYTSALL	0.1387480
1136	HLA-C*08:01	TVYDPLQPEL	0.1270010
773	HLA-C*08:01	EQDKNTQEV	0.1089260

HLA-C08:02

Pos	ALLELE	Peptide	Score_EL
109	HLA-C*08:02	TLDSKTQSL	0.9705090
285	HLA-C*08:02	ITDAVDCAL	0.7479830
1137	HLA-C*08:02	VYDPLQPEL	0.7445000
584	HLA-C*08:02	ILDITPCSF	0.7303380
612	HLA-C*08:02	YQDVNCTEV	0.6418140
1144	HLA-C*08:02	ELDSFKEEL	0.5766540
1136	HLA-C*08:02	TVYDPLQPEL	0.5470820
108	HLA-C*08:02	TTLDSKTQSL	0.5104370
983	HLA-C*08:02	RLDKVEAEV	0.4849050
625	HLA-C*08:02	HADQLTPTW	0.4555270
841	HLA-C*08:02	LGDIAARDL	0.4328330
109	HLA-C*08:02	TLDSKTQSLL	0.4276660
773	HLA-C*08:02	EQDKNTQEV	0.3981320
572	HLA-C*08:02	TTDAVRDPQTL	0.3291510
78	HLA-C*08:02	RFDNPVLPF	0.3203470
269	HLA-C*08:02	YLQPRTFLL	0.3135080
51	HLA-C*08:02	TQDLFLPFF	0.2869890
951	HLA-C*08:02	VVNQNAQAL	0.2860300
723	HLA-C*08:02	TTEILPVSM	0.2850680
575	HLA-C*08:02	AVRDPQTLEI	0.2820630
865	HLA-C*08:02	LTDEMIAQY	0.2542320
1135	HLA-C*08:02	NTVYDPLQPEL	0.2244430

HLA-C12:03

Pos	ALLELE	Peptide	Score_EL
687	HLA-C*12:03	VASQSIIAY	0.7730470
30	HLA-C*12:03	NSFTRGVYY	0.6720350
204	HLA-C*12:03	YSKHTPINL	0.6283700
718	HLA-C*12:03	FTISVTTEI	0.6082460
1054	HLA-C*12:03	QSAPHGVVF	0.6072830
712	HLA-C*12:03	IAIPTNFTI	0.4975140
28	HLA-C*12:03	YTNSFTRGV	0.4235380
122	HLA-C*12:03	NATNVVIKV	0.3745740
343	HLA-C*12:03	NATRFASVY	0.3531300
1055	HLA-C*12:03	SAPHGVVFL	0.3364220
1021	HLA-C*12:03	SANLAATKM	0.3232800
1060	HLA-C*12:03	VVFLHVTYV	0.3213390

267	HLA-C*12:03	VGYLQPRTF	0.3170570
898	HLA-C*12:03	FAMQMAYRF	0.3149780
894	HLA-C*12:03	LQIPFAMQM	0.3132070
1189	HLA-C*12:03	VAKNLNESL	0.3086180
505	HLA-C*12:03	YQPYRVVVL	0.3041170
604	HLA-C*12:03	TSNQVAVLY	0.3004760
162	HLA-C*12:03	SANNCTFEY	0.2984870
923	HLA-C*12:03	IANQFNSAI	0.2968270
892	HLA-C*12:03	AALQIPFAM	0.2931450
372	HLA-C*12:03	ASFSTFKCY	0.2813160
710	HLA-C*12:03	NSIAIPTNF	0.2780310
221	HLA-C*12:03	SALEPLVDL	0.2403750
943	HLA-C*12:03	SALGKLQDV	0.2359520
92	HLA-C*12:03	FASTEKSNI	0.2341030
262	HLA-C*12:03	AAAYYVGYL	0.1902180
929	HLA-C*12:03	SAIGKIQDSL	0.1852030

HLA-C14:02

Pos	ALLELE	Peptide	Score_EL
635	HLA-C*14:02	VYSTGSNVF	0.9423850
1137	HLA-C*14:02	VYDPLQPEL	0.9023700
781	HLA-C*14:02	VFAQVKQIY	0.8433820
489	HLA-C*14:02	YFPLQSYGF	0.7241080
268	HLA-C*14:02	GYLQPRTFL	0.6603000
78	HLA-C*14:02	RFDNPVLPF	0.5954090
144	HLA-C*14:02	YYHKNNKSW	0.5706230
265	HLA-C*14:02	YYVGYLQPRTF	0.5687450
706	HLA-C*14:02	AYSNNSIAI	0.5350600
167	HLA-C*14:02	TFEYVSQPF	0.5153070
969	HLA-C*14:02	NFGAISSVL	0.5103720
1101	HLA-C*14:02	HWFVTQRNF	0.5043790
144	HLA-C*14:02	YYHKNNKSWM	0.5039240
448	HLA-C*14:02	NYNYLYRLF	0.4892330
193	HLA-C*14:02	VFKNIDGYF	0.4846810
1102	HLA-C*14:02	WFVTQRNFY	0.4783250
368	HLA-C*14:02	LYNSASFSTF	0.4577280
159	HLA-C*14:02	VYSSANNCTF	0.4555730
504	HLA-C*14:02	GYQPYRVVV	0.4060140
755	HLA-C*14:02	QYGSFCTQL	0.3929240
1147	HLA-C*14:02	SFKEELDKY	0.3899710
1094	HLA-C*14:02	VFVSNGTHW	0.3697360
379	HLA-C*14:02	CYGVSPTKL	0.3394690
505	HLA-C*14:02	YQPYRVVVL	0.3382200
169	HLA-C*14:02	EYVSQPFLM	0.3368910
788	HLA-C*14:02	IYKTPPIKDF	0.3282130
642	HLA-C*14:02	VFQTRAGCL	0.3240530
634	HLA-C*14:02	RVYSTGSNVF	0.3232280

HLA-C15:02

Pos	ALLELE	Peptide	Score_EL
718	HLA-C*15:02	FTISVTTEI	0.8099620
28	HLA-C*15:02	YTNSFTRGV	0.8076240
204	HLA-C*15:02	YSKHTPINL	0.8013210
1096	HLA-C*15:02	VSNGTHWFV	0.6809840
171	HLA-C*15:02	VSQPFLMDL	0.6777380
1060	HLA-C*15:02	VVFLHVTYV	0.6690310
712	HLA-C*15:02	IAIPTNFTI	0.6280920
62	HLA-C*15:02	VTWFHAIHV	0.5490730
634	HLA-C*15:02	RVYSTGSNV	0.5091970

269	HLA-C*15:02	YLQPRTFLL	0.5012370
417	HLA-C*15:02	KIADYNYKL	0.4782510
1054	HLA-C*15:02	QSAPHGVVF	0.4662000
122	HLA-C*15:02	NATNVVIKV	0.4134190
221	HLA-C*15:02	SALEPLVDL	0.4075260
943	HLA-C*15:02	SALGKLQDV	0.4055910
691	HLA-C*15:02	SIIAYTMSL	0.4048960
894	HLA-C*15:02	LQIPFAMQM	0.3769380
1175	HLA-C*15:02	SVVNIQKEI	0.3722220
968	HLA-C*15:02	SNFGAISSV	0.3703380
1005	HLA-C*15:02	QTYVTQQLI	0.3621440
892	HLA-C*15:02	AALQIPFAM	0.3446720
195	HLA-C*15:02	KNIDGYFKI	0.3438870
777	HLA-C*15:02	NTQEVFAQV	0.3401370
940	HLA-C*15:02	STASALGKL	0.3389230
135	HLA-C*15:02	FCNDPFLGV	0.3269320
976	HLA-C*15:02	VLNDILSRL	0.3160940
262	HLA-C*15:02	AAAYYVGYL	0.3139630
93	HLA-C*15:02	ASTEKSNII	0.3083780
923	HLA-C*15:02	IANQFNSAI	0.3031780
233	HLA-C*15:02	INITRFQTL	0.3017440
1055	HLA-C*15:02	SAPHGVVFL	0.3003080
304	HLA-C*15:02	KSFTVEKGI	0.2994430
1021	HLA-C*15:02	SANLAATKM	0.2989090
202	HLA-C*15:02	KIYSKHTPI	0.2968020

HLA-C16:01

Pos	ALLELE	Peptide	Score_EL
1054	HLA-C*16:01	QSAPHGVVF	0.9155490
687	HLA-C*16:01	VASQSIIAY	0.9081000
30	HLA-C*16:01	NSFTRGVYY	0.8073520
204	HLA-C*16:01	YSKHTPINL	0.7482230
1021	HLA-C*16:01	SANLAATKM	0.6740910
343	HLA-C*16:01	NATRFASVY	0.6206200
1189	HLA-C*16:01	VAKNLNESL	0.5673400
162	HLA-C*16:01	SANNCTFEY	0.5420610
160	HLA-C*16:01	YSSANNCTF	0.5397300
267	HLA-C*16:01	VGYLQPRTF	0.5362580
892	HLA-C*16:01	AALQIPFAM	0.5293490
604	HLA-C*16:01	TSNQVAVLY	0.5051130
898	HLA-C*16:01	FAMQMAYRF	0.4361040
712	HLA-C*16:01	IAIPTNFTI	0.4105260
923	HLA-C*16:01	IANQFNSAI	0.3950200
372	HLA-C*16:01	ASFSTFKCY	0.3920010
1055	HLA-C*16:01	SAPHGVVFL	0.3824000
699	HLA-C*16:01	LGAENSVAY	0.3783070
951	HLA-C*16:01	VVNQNAQAL	0.3589760
221	HLA-C*16:01	SALEPLVDL	0.3564080
710	HLA-C*16:01	NSIAIPTNF	0.3498980
269	HLA-C*16:01	YLQPRTFLL	0.3264340
870	HLA-C*16:01	IAQYTSALL	0.3255700
718	HLA-C*16:01	FTISVTTEI	0.3206320
1052	HLA-C*16:01	FPQSAPHGVVF	0.2924260

HLA-C17:01

Pos	ALLELE	Peptide	Score_EL
269	HLA-C*17:01	YLQPRTFLL	0.8724580
718	HLA-C*17:01	FTISVTTEI	0.8518340
712	HLA-C*17:01	IAIPTNFTI	0.7804690
221	HLA-C*17:01	SALEPLVDL	0.7746260
109	HLA-C*17:01	TLDSKTQSL	0.7705340

```
 976 HLA-C*17:01      VLNDILSRL 0.7539870
1055 HLA-C*17:01      SAPHGVVFL 0.7138380
 204 HLA-C*17:01      YSKHTPINL 0.6995850
 417 HLA-C*17:01      KIADYNYKL 0.6874430
 691 HLA-C*17:01      SIIAYTMSL 0.5871280
1136 HLA-C*17:01     TVYDPLQPEL 0.5568080
 951 HLA-C*17:01      VVNQNAQAL 0.5438950
1137 HLA-C*17:01      VYDPLQPEL 0.5344550
 892 HLA-C*17:01      AALQIPFAM 0.5216840
 262 HLA-C*17:01      AAAYYVGYL 0.5133210
 870 HLA-C*17:01      IAQYTSALL 0.4969470
 171 HLA-C*17:01      VSQPFLMDL 0.4905060
 923 HLA-C*17:01      IANQFNSAI 0.4507970
1060 HLA-C*17:01      VVFLHVTYV 0.4413040
1054 HLA-C*17:01      QSAPHGVVF 0.4376900
1095 HLA-C*17:01      FVSNGTHWF 0.4245460
 894 HLA-C*17:01      LQIPFAMQM 0.4150410
 505 HLA-C*17:01      YQPYRVVVL 0.3991670
 888 HLA-C*17:01      FGAGAALQI 0.3912130
 869 HLA-C*17:01      MIAQYTSAL 0.3900610
 886 HLA-C*17:01      WTFGAGAAL 0.3744180
-------------------------------------------
```

Note: The epitopes conserved among Khosta-2 and SARS-CoV-2 are indicated in red.
