# Supplementary material for: Conservation of HLA Spike Protein Epitopes Supports T Cell Cross-Protection in SARS-CoV-2 Vaccinated Individuals against the Potentially Zoonotic Coronavirus Khosta-2
Source: Int J Mol Sci. 2024 May 31;25(11):6087. doi: 10.3390/ijms25116087 (PMC11172828; doi:10.3390/ijms25116087)
Supplement: Supplementary file 1 [file ijms-25-06087-s001.zip › Supplemental Table 4.pdf]

Supplemental Table 4

Allele: DRB1_0101

Pos	MHC	Peptide	Of	Core	Core_Rel	Score_EL
540	DRB1_0101	NFNFNGLTGTGVLTE	3	FNGLTGTGV	1.000	0.938016
512	DRB1_0101	VLSFELLHAPATVCG	3	FELLHAPAT	0.967	0.811818
314	DRB1_0101	QTSNFRVQPTESIVR	4	FRVQPTESI	1.000	0.781642
492	DRB1_0101	LQSYGFQPTNGVGYQ	3	YGFQPTNGV	0.993	0.718623
514	DRB1_0101	SFELLHAPATVCGPK	3	LLHAPATVC	0.927	0.717202
303	DRB1_0101	LKSFTVEKGIYQTSN	3	FTVEKGIYQ	1.000	0.709921
1057	DRB1_0101	PHGVVFLHVTYVPAQ	3	VVFLHVTYV	0.993	0.692500
197	DRB1_0101	IDGYFKIYSKHTPIN	3	YFKIYSKHT	1.000	0.607851

Allele: DRB1_0401

Pos	MHC	Peptide	Of	Core	Core_Rel	Score_EL
33	DRB1_0401	TRGVYYPDKVFRSSV	4	YYPDKVFRS	1.000	0.905995
314	DRB1_0401	QTSNFRVQPTESIVR	4	FRVQPTESI	1.000	0.898292
1111	DRB1_0401	EPQIITTDNTFVSGN	4	ITTDNTFVS	0.927	0.821410
802	DRB1_0401	FSQILPDPSKPSKRS	3	ILPDPSKPS	1.000	0.819922
1014	DRB1_0401	RAAEIRASANLAATK	4	IRASANLAA	1.000	0.802608
492	DRB1_0401	LQSYGFQPTNGVGYQ	3	YGFQPTNGV	1.000	0.759073
86	DRB1_0401	FNDGVYFASTEKSNI	4	VYFASTEKS	1.000	0.749776
362	DRB1_0401	VADYSVLYNSASFST	3	YSVLYNSAS	1.000	0.665981
303	DRB1_0401	LKSFTVEKGIYQTSN	3	FTVEKGIYQ	1.000	0.587244
1099	DRB1_0401	GTHWFVTQRNFYEPQ	4	FVTQRNFYE	0.987	0.556554
207	DRB1_0401	HTPINLVRDLPQGFS	3	INLVRDLPQ	0.993	0.522253

Allele: DRB1_0404

Pos	MHC	Peptide	Of	Core	Core_Rel	Score_EL
207	DRB1_0404	HTPINLVRDLPQGFS	3	INLVRDLPQ	1.000	0.953780
1014	DRB1_0404	RAAEIRASANLAATK	4	IRASANLAA	1.000	0.923993
232	DRB1_0404	GINITRFQTLLALHR	3	ITRFQTLLA	1.000	0.895299
86	DRB1_0404	FNDGVYFASTEKSNI	4	VYFASTEKS	1.000	0.852579
928	DRB1_0404	NSAIGKIQDSLSSTA	3	IGKIQDSLS	1.000	0.848992
960	DRB1_0404	NTLVKQLSSNFGAIS	3	VKQLSSNFG	1.000	0.812821
1111	DRB1_0404	EPQIITTDNTFVSGN	4	ITTDNTFVS	0.720	0.761306
802	DRB1_0404	FSQILPDPSKPSKRS	3	ILPDPSKPS	1.000	0.723057
944	DRB1_0404	ALGKLQDVVNQNAQA	4	LQDVVNQNA	1.000	0.668202
702	DRB1_0404	ENSVAYSNNSIAIPT	3	VAYSNNSIA	1.000	0.663927
323	DRB1_0404	TESIVRFPNITNLCP	3	IVRFPNITN	1.000	0.655964

690	DRB1_0404	QSIIAYTMSLGAENS	3	IAYTMSLGA	0.973	0.638258
113	DRB1_0404	KTQSLLIVNNATNVV	4	LLIVNNATN	0.993	0.614084
300	DRB1_0404	KCTLKSFTVEKGIYQ	3	LKSFTVEKG	1.000	0.600123
767	DRB1_0404	LTGIAVEQDKNTQEV	3	IAVEQDKNT	0.967	0.597703
1126	DRB1_0404	CDVVIGIVNNTVYDP	3	VIGIVNNTV	0.800	0.589583

Allele: DRB1_0701

Pos	MHC	Peptide	Of	Core	Core_Rel	Score_EL
309	DRB1_0701	EKGIYQTSNFRVQPT	3	IYQTSNFRV	1.000	0.912889
714	DRB1_0701	IPTNFTISVTTEILP	4	FTISVTTEI	1.000	0.896225
494	DRB1_0701	SYGFQPTNGVGYQPY	3	FQPTNGVGY	1.000	0.821964
632	DRB1_0701	TWRVYSTGSNVFQTR	3	VYSTGSNVF	1.000	0.716526
690	DRB1_0701	QSIIAYTMSLGAENS	3	IAYTMSLGA	0.973	0.681335
1153	DRB1_0701	DKYFKNHTSPDVDLG	3	FKNHTSPDV	0.973	0.639460
25	DRB1_0701	PPAYTNSFTRGVYYP	3	YTNSFTRGV	1.000	0.637973
1111	DRB1_0701	EPQIITTDNTFVSGN	3	IITTDNTFV	0.993	0.568150
201	DRB1_0701	FKIYSKHTPINLVRD	3	YSKHTPINL	0.960	0.531197
681	DRB1_0701	PRRARSVASQSIIAY	3	ARSVASQSI	0.993	0.524826
797	DRB1_0701	FGGFNFSQILPDPSK	3	FNFSQILPD	0.960	0.476780

Allele: DRB1_0802

Pos	MHC	Peptide	Of	Core	Core_Rel	Score_EL
1014	DRB1_0802	RAAEIRASANLAATK	4	IRASANLAA	0.960	0.603367
61	DRB1_0802	NVTWFHAIHVSGTNG	3	WFHAIHVSG	0.480	0.553878
272	DRB1_0802	PRTFLLKYNENGTIT	3	FLLKYNENG	0.993	0.546261
1009	DRB1_0802	TQQLIRAAEIRASAN	4	IRAAEIRAS	0.567	0.534196
446	DRB1_0802	GGNYNYLYRLFRKSN	3	YNYLYRLFR	0.940	0.524253
978	DRB1_0802	NDILSRLDKVEAEVQ	3	LSRLDKVEA	0.980	0.523589

Allele: DRB1_0901

Pos	MHC	Peptide	Of	Core	Core_Rel	Score_EL
714	DRB1_0901	IPTNFTISVTTEILP	4	FTISVTTEI	1.000	0.845552
883	DRB1_0901	TSGWTFGAGAALQIP	3	WTFGAGAAL	0.553	0.780244
885	DRB1_0901	GWTFGAGAALQIPFA	3	FGAGAALQI	0.953	0.722468
25	DRB1_0901	PPAYTNSFTRGVYYP	3	YTNSFTRGV	0.987	0.656755
493	DRB1_0901	QSYGFQPTNGVGYQP	4	FQPTNGVGY	0.793	0.655929
255	DRB1_0901	SSGWTAGAAAYYVGY	3	WTAGAAAYY	0.907	0.629042
309	DRB1_0901	EKGIYQTSNFRVQPT	3	IYQTSNFRV	0.967	0.618192

690	DRB1_0901	QSIIAYTMSLGAENS	3	IAYTMSLGA	0.640	0.586207
923	DRB1_0901	IANQFNSAIGKIQDS	4	FNSAIGKIQ	0.980	0.565579
797	DRB1_0901	FGGFNFSQILPDPSK	3	FNFSQILPD	0.913	0.550854
60	DRB1_0901	SNVTWFHAIHVSGTN	2	VTWFHAIHV	0.427	0.538172
540	DRB1_0901	NFNFNGLTGTGVLTE	3	FNGLTGTGV	1.000	0.538096
1064	DRB1_0901	HVTYVPAQEKNFTTA	3	YVPAQEKNF	0.993	0.533012
87	DRB1_0901	NDGVYFASTEKSNII	4	YFASTEKSN	0.460	0.520388

Allele: DRB1_1001

Pos	MHC	Peptide	Of	Core	Core_Rel	Score_EL
1135	DRB1_1001	NTVYDPLQPELDSFK	3	YDPLQPELD	1.000	0.890783
216	DRB1_1001	LPQGFSALEPLVDLP	4	FSALEPLVD	1.000	0.879496
966	DRB1_1001	LSSNFGAISSVLNDI	4	FGAISSVLN	1.000	0.855589
314	DRB1_1001	QTSNFRVQPTESIVR	4	FRVQPTESI	1.000	0.787133
799	DRB1_1001	GFNFSQILPDPSKPS	3	FSQILPDPS	0.980	0.731931
362	DRB1_1001	VADYSVLYNSASFST	3	YSVLYNSAS	0.980	0.708692
62	DRB1_1001	VTWFHAIHVSGTNGT	3	FHAIHVSGT	0.973	0.698477
492	DRB1_1001	LQSYGFQPTNGVGYQ	3	YGFQPTNGV	0.987	0.626730
87	DRB1_1001	NDGVYFASTEKSNII	3	VYFASTEKS	0.867	0.623790
692	DRB1_1001	IIAYTMSLGAENSVA	3	YTMSLGAEN	0.993	0.616190
303	DRB1_1001	LKSFTVEKGIYQTSN	3	FTVEKGIYQ	1.000	0.608513
512	DRB1_1001	VLSFELLHAPATVCG	3	FELLHAPAT	0.953	0.597462

Allele: DRB1_1101

Pos	MHC	Peptide	Of	Core	Core_Rel	Score_EL
978	DRB1_1101	NDILSRLDKVEAEVQ	3	LSRLDKVEA	1.000	0.806222
955	DRB1_1101	NAQALNTLVKQLSSN	4	LNTLVKQLS	1.000	0.741440
446	DRB1_1101	GGNYNYLYRLFRKSN	3	YNYLYRLFR	1.000	0.722800

Allele: DRB1_1302

Pos	MHC	Peptide	Of	Core	Core_Rel	Score_EL
115	DRB1_1302	QSLLIVNNATNVVIK	3	LIVNNATNV	0.507	0.768378
116	DRB1_1302	SLLIVNNATNVVIKV	3	IVNNATNVV	0.740	0.722566
309	DRB1_1302	EKGIYQTSNFRVQPT	3	IYQTSNFRV	1.000	0.682084
1014	DRB1_1302	RAAEIRASANLAATK	4	IRASANLAA	0.993	0.615121
1092	DRB1_1302	EGVFVSNGTHWFVTQ	3	FVSNGTHWF	0.987	0.606284
912	DRB1_1302	TQNVLYENQKLIANQ	4	LYENQKLIA	0.713	0.583838

818	DRB1_1302	IEDLLFNKVTLADAG	3	LLFNKVTLA	0.967	0.583772
274	DRB1_1302	TFLLKYNENGTITDA	3	LKYNENGTI	0.973	0.582775
802	DRB1_1302	FSQILPDPSKPSKRS	3	ILPDPSKPS	1.000	0.538356
314	DRB1_1302	QTSNFRVQPTESIVR	4	FRVQPTESI	0.987	0.535912
1111	DRB1_1302	EPQIITTDNTFVSGN	3	IITTDNTFV	0.627	0.526440
430	DRB1_1302	TGCVIAWNSNNLDSK	4	IAWNSNNLD	0.493	0.517543
778	DRB1_1302	TQEVFAQVKQIYKTP	3	VFAQVKQIY	0.893	0.501246
1099	DRB1_1302	GTHWFVTQRNFYEPQ	3	WFVTQRNFY	0.853	0.472552
346	DRB1_1302	RFASVYAWNRKRISN	4	VYAWNRKRI	0.507	0.450526
1153	DRB1_1302	DKYFKNHTSPDVDLG	3	FKNHTSPDV	0.753	0.443493
919	DRB1_1302	NQKLIANQFNSAIGK	3	LIANQFNSA	0.740	0.438946
142	DRB1_1302	GVYYHKNNKSWMESE	3	YHKNNKSWM	0.913	0.437505
33	DRB1_1302	TRGVYYPDKVFRSSV	4	YYPDKVFRS	0.933	0.425745
947	DRB1_1302	KLQDVVNQNAQALNT	4	VVNQNAQAL	0.707	0.425418

Allele: DRB1_1501

Pos	MHC	Peptide	Of	Core	Core_Rel	Score_EL
867	DRB1_1501	DEMIAQYTSALLAGT	3	IAQYTSALL	1.000	0.989184
430	DRB1_1501	TGCVIAWNSNNLDSK	3	VIAWNSNNL	1.000	0.966005
309	DRB1_1501	EKGIYQTSNFRVQPT	3	IYQTSNFRV	1.000	0.922581
232	DRB1_1501	GINITRFQTLLALHR	3	ITRFQTLLA	1.000	0.857700
59	DRB1_1501	FSNVTWFHAIHVSGT	3	VTWFHAIHV	1.000	0.835947
1001	DRB1_1501	LQSLQTYVTQQLIRA	3	LQTYVTQQL	1.000	0.820364
790	DRB1_1501	KTPPIKDFGGFNFSQ	4	IKDFGGFNF	1.000	0.818158
323	DRB1_1501	TESIVRFPNITNLCP	3	IVRFPNITN	1.000	0.796209
831	DRB1_1501	AGFIKQYGDCLGDIA	3	IKQYGDCLG	1.000	0.567814
1014	DRB1_1501	RAAEIRASANLAATK	4	IRASANLAA	1.000	0.559269
1126	DRB1_1501	CDVVIGIVNNTVYDP	3	VIGIVNNTV	0.847	0.540038
689	DRB1_1501	SQSIIAYTMSLGAEN	3	IIAYTMSLG	0.813	0.533005
239	DRB1_1501	QTLLALHRSYLTPGD	3	LALHRSYLT	0.967	0.526434
911	DRB1_1501	VTQNVLYENQKLIAN	3	NVLYENQKL	0.973	0.468846

Allele: DRB1_1602

Pos	MHC	Peptide	Of	Core	Core_Rel	Score_EL
867	DRB1_1602	DEMIAQYTSALLAGT	3	IAQYTSALL	1.000	0.825191
309	DRB1_1602	EKGIYQTSNFRVQPT	3	IYQTSNFRV	1.000	0.776902
430	DRB1_1602	TGCVIAWNSNNLDSK	3	VIAWNSNNL	0.973	0.679206
314	DRB1_1602	QTSNFRVQPTESIVR	4	FRVQPTESI	1.000	0.643355
1153	DRB1_1602	DKYFKNHTSPDVDLG	3	FKNHTSPDV	0.933	0.587700
59	DRB1_1602	FSNVTWFHAIHVSGT	3	VTWFHAIHV	0.987	0.573085
343	DRB1_1602	NATRFASVYAWNRKR	4	FASVYAWNR	0.993	0.552323

197	DRB1_1602	IDGYFKIYSKHTPIN	3	YFKIYSKHT	0.907	0.540575
153	DRB1_1602	MESEFRVYSSANNCT	4	FRVYSSANN	1.000	0.534802
232	DRB1_1602	GINITRFQTLLALHR	3	ITRFQTLLA	0.973	0.528208
1111	DRB1_1602	EPQIITTDNTFVSGN	3	IITTDNTFV	0.920	0.490496
556	DRB1_1602	NKKFLPFQQFGRDIA	3	FLPFQQFGR	0.987	0.480254
502	DRB1_1602	GVGYQPYRVVVLSFE	3	YQPYRVVVL	1.000	0.473389
790	DRB1_1602	KTPPIKDFGGFNFSQ	4	IKDFGGFNF	1.000	0.458410

Allele: DRB3_0301

Pos	MHC	Peptide	Of	Core	Core_Rel	Score_EL
115	DRB3_0301	QSLLIVNNATNVVIK	4	IVNNATNVV	0.493	0.717182
1111	DRB3_0301	EPQIITTDNTFVSGN	3	IITTDNTFV	0.640	0.667030
114	DRB3_0301	TQSLLIVNNATNVVI	4	LIVNNATNV	0.533	0.638262
1014	DRB3_0301	RAAEIRASANLAATK	4	IRASANLAA	0.987	0.626741
309	DRB3_0301	EKGIYQTSNFRVQPT	3	IYQTSNFRV	1.000	0.622539
818	DRB3_0301	IEDLLFNKVTLADAG	3	LLFNKVTLA	0.993	0.607752
274	DRB3_0301	TFLLKYNENGTITDA	3	LKYNENGTI	0.927	0.579236
802	DRB3_0301	FSQILPDPSKPSKRS	3	ILPDPSKPS	1.000	0.573097
399	DRB3_0301	SFVIRGDEVRQIAPG	3	IRGDEVRQI	0.993	0.549064
430	DRB3_0301	TGCVIAWNSNNLDSK	4	IAWNSNNLD	0.827	0.534940
702	DRB3_0301	ENSVAYSNNSIAIPT	3	VAYSNNSIA	0.667	0.506589
314	DRB3_0301	QTSNFRVQPTESIVR	4	FRVQPTESI	0.953	0.482288
1127	DRB3_0301	DVVIGIVNNTVYDPL	2	VIGIVNNTV	0.467	0.471003
1126	DRB3_0301	CDVVIGIVNNTVYDP	4	IGIVNNTVY	0.487	0.467142
1092	DRB3_0301	EGVFVSNGTHWFVTQ	3	FVSNGTHWF	0.900	0.460335
947	DRB3_0301	KLQDVVNQNAQALNT	4	VVNQNAQAL	0.607	0.451712
530	DRB3_0301	STNLVKNKCVNFNFN	3	LVKNKCVNF	0.993	0.432644
65	DRB3_0301	FHAIHVSGTNGTKRF	3	IHVSGTNGT	0.980	0.423558
364	DRB3_0301	DYSVLYNSASFSTFK	3	VLYNSASFS	0.867	0.421713
906	DRB3_0301	FNGIGVTQNVLYENQ	3	IGVTQNVLY	0.853	0.419595
703	DRB3_0301	NSVAYSNNSIAIPTN	3	AYSNNSIAI	0.533	0.413595
1185	DRB3_0301	RLNEVAKNLNESLID	4	VAKNLNESL	1.000	0.411978

Allele: DRB4_0101

Pos	MHC	Peptide	Of	Core	Core_Rel	Score_EL
1190	DRB4_0101	AKNLNESLIDLQELG	3	LNESLIDLQ	1.000	0.373391
1009	DRB4_0101	TQQLIRAAEIRASAN	4	IRAAEIRAS	0.800	0.372095
1014	DRB4_0101	RAAEIRASANLAATK	4	IRASANLAA	0.973	0.344867
86	DRB4_0101	FNDGVYFASTEKSNI	4	VYFASTEKS	1.000	0.333224
355	DRB4_0101	RKRISNCVADYSVLY	3	ISNCVADYS	1.000	0.319595
314	DRB4_0101	QTSNFRVQPTESIVR	4	FRVQPTESI	0.987	0.318780
988	DRB4_0101	EAEVQIDRLITGRLQ	3	VQIDRLITG	0.953	0.303910

39	DRB4_0101	PDKVFRSSVLHSTQD	3	VFRSSVLHS	0.927	0.294615
799	DRB4_0101	GFNFSQILPDPSKPS	3	FSQILPDPS	0.960	0.294276
207	DRB4_0101	HTPINLVRDLPQGFS	3	INLVRDLPQ	0.927	0.274844
457	DRB4_0101	RKSNLKPFERDISTE	4	LKPFERDIS	0.993	0.268178
1003	DRB4_0101	SLQTYVTQQLIRAAE	4	YVTQQLIRA	0.733	0.259812
802	DRB4_0101	FSQILPDPSKPSKRS	3	ILPDPSKPS	1.000	0.259521
1005	DRB4_0101	QTYVTQQLIRAAEIR	3	VTQQLIRAA	0.547	0.239215
764	DRB4_0101	NRALTGIAVEQDKNT	3	LTGIAVEQD	0.973	0.231179
1168	DRB4_0101	DISGINASVVNIQKE	4	INASVVNIQ	1.000	0.227370

Allele: DRB4_0103

Pos	MHC	Peptide	Of	Core	Core_Rel	Score_EL
1190	DRB4_0103	AKNLNESLIDLQELG	3	LNESLIDLQ	1.000	0.373391
1009	DRB4_0103	TQQLIRAAEIRASAN	4	IRAAEIRAS	0.800	0.372095
1014	DRB4_0103	RAAEIRASANLAATK	4	IRASANLAA	0.973	0.344867
86	DRB4_0103	FNDGVYFASTEKSNI	4	VYFASTEKS	1.000	0.333224
355	DRB4_0103	RKRISNCVADYSVLY	3	ISNCVADYS	1.000	0.319595
314	DRB4_0103	QTSNFRVQPTESIVR	4	FRVQPTESI	0.987	0.318780
988	DRB4_0103	EAEVQIDRLITGRLQ	3	VQIDRLITG	0.953	0.303910
39	DRB4_0103	PDKVFRSSVLHSTQD	3	VFRSSVLHS	0.927	0.294615
799	DRB4_0103	GFNFSQILPDPSKPS	3	FSQILPDPS	0.960	0.294276
207	DRB4_0103	HTPINLVRDLPQGFS	3	INLVRDLPQ	0.927	0.274844
457	DRB4_0103	RKSNLKPFERDISTE	4	LKPFERDIS	0.993	0.268178
1003	DRB4_0103	SLQTYVTQQLIRAAE	4	YVTQQLIRA	0.733	0.259812
802	DRB4_0103	FSQILPDPSKPSKRS	3	ILPDPSKPS	1.000	0.259521
1005	DRB4_0103	QTYVTQQLIRAAEIR	3	VTQQLIRAA	0.547	0.239215
764	DRB4_0103	NRALTGIAVEQDKNT	3	LTGIAVEQD	0.973	0.231179
1168	DRB4_0103	DISGINASVVNIQKE	4	INASVVNIQ	1.000	0.227370

Allele: DRB5_0101

Pos	MHC	Peptide	Of	Core	Core_Rel	Score_EL
343	DRB5_0101	NATRFASVYAWNRKR	4	FASVYAWNR	1.000	0.924594
672	DRB5_0101	ASYQTQTNSPRRARS	2	YQTQTNSPR	0.993	0.839527
779	DRB5_0101	QEVFAQVKQIYKTPP	3	FAQVKQIYK	0.993	0.811819
234	DRB5_0101	NITRFQTLLALHRSY	4	FQTLLALHR	1.000	0.811567
191	DRB5_0101	EFVFKNIDGYFKIYS	3	FKNIDGYFK	1.000	0.727983
1062	DRB5_0101	FLHVTYVPAQEKNFT	3	VTYVPAQEK	0.980	0.691341
303	DRB5_0101	LKSFTVEKGIYQTSN	3	FTVEKGIYQ	1.000	0.677725
556	DRB5_0101	NKKFLPFQQFGRDIA	3	FLPFQQFGR	1.000	0.612992
315	DRB5_0101	TSNFRVQPTESIVRF	3	FRVQPTESI	0.887	0.604856

Allele: HLA-DPA10103-DPB10201

Pos	MHC	Peptide	Of	Core	Core_Rel	Score_EL
338	HLA-DPA10103-DPB10201	FGEVFNATRFASVYA	4	FNATRFASV	1.000	0.958081
137	HLA-DPA10103-DPB10201	NDPFLGVYYHKNNKS	3	FLGVYYHKN	1.000	0.927370
448	HLA-DPA10103-DPB10201	NYNYLYRLFRKSNLK	3	YLYRLFRKS	1.000	0.900380
919	HLA-DPA10103-DPB10201	NQKLIANQFNSAIGK	3	LIANQFNSA	1.000	0.854497
310	HLA-DPA10103-DPB10201	KGIYQTSNFRVQPTE	3	YQTSNFRVQ	1.000	0.798770
1059	HLA-DPA10103-DPB10201	GVVFLHVTYVPAQEK	3	FLHVTYVPA	0.993	0.752146
39	HLA-DPA10103-DPB10201	PDKVFRSSVLHSTQD	4	FRSSVLHST	1.000	0.751483
229	HLA-DPA10103-DPB10201	LPIGINITRFQTLLA	4	INITRFQTL	1.000	0.740551
500	HLA-DPA10103-DPB10201	TNGVGYQPYRVVVLS	3	VGYQPYRVV	0.987	0.596417
186	HLA-DPA10103-DPB10201	FKNLREFVFKNIDGY	3	LREFVFKNI	1.000	0.556008
794	HLA-DPA10103-DPB10201	IKDFGGFNFSQILPD	3	FGGFNFSQI	0.980	0.542803
489	HLA-DPA10103-DPB10201	YFPLQSYGFQPTNGV	3	LQSYGFQPT	1.000	0.524831
1039	HLA-DPA10103-DPB10201	RVDFCGKGYHLMSFP	3	FCGKGYHLM	1.000	0.522161
167	HLA-DPA10103-DPB10201	TFEYVSQPFLMDLEG	3	YVSQPFLMD	1.000	0.490941
815	HLA-DPA10103-DPB10201	RSFIEDLLFNKVTLA	3	IEDLLFNKV	1.000	0.474460
1044	HLA-DPA10103-DPB10201	GKGYHLMSFPQSAPH	3	YHLMSFPQS	0.973	0.374533
149	HLA-DPA10103-DPB10201	NKSWMESEFRVYSSA	3	WMESEFRVY	0.993	0.341433

Allele: HLA-DPA10103-DPB10301

Pos	MHC	Peptide	Of	Core	Core_Rel	Score_EL
679	HLA-DPA10103-DPB10301	NSPRRARSVASQSII	4	RARSVASQS	0.680	0.828726
154	HLA-DPA10103-DPB10301	ESEFRVYSSANNCTF	4	RVYSSANNC	0.693	0.733745
1182	HLA-DPA10103-DPB10301	EIDRLNEVAKNLNES	3	RLNEVAKNL	0.700	0.657668
762	HLA-DPA10103-DPB10301	QLNRALTGIAVEQDK	3	RALTGIAVE	0.760	0.657035
1016	HLA-DPA10103-DPB10301	AEIRASANLAATKMS	3	RASANLAAT	0.867	0.643581
17	HLA-DPA10103-DPB10301	NLTTRTQLPPAYTNS	4	RTQLPPAYT	0.700	0.624748
234	HLA-DPA10103-DPB10301	NITRFQTLLALHRSY	3	RFQTLLALH	0.753	0.617181
630	HLA-DPA10103-DPB10301	TPTWRVYSTGSNVFQ	4	RVYSTGSNV	0.707	0.597643
343	HLA-DPA10103-DPB10301	NATRFASVYAWNRKR	3	RFASVYAWN	0.747	0.541066
404	HLA-DPA10103-DPB10301	GDEVRQIAPGQTGKI	4	RQIAPGQTG	0.680	0.540501
243	HLA-DPA10103-DPB10301	ALHRSYLTPGDSSSG	3	RSYLTPGDS	0.733	0.535052
1087	HLA-DPA10103-DPB10301	AHFPREGVFVSNGTH	4	REGVFVSNG	0.700	0.514743

Allele: HLA-DPA10103-DPB10401

Pos	MHC	Peptide	Of	Core	Core_Rel	Score_EL

338	HLA-DPA10103-DPB10401	FGEVFNATRFASVYA	4	FNATRFASV	1.000	0.940954
167	HLA-DPA10103-DPB10401	TFEYVSQPFLMDLEG	3	YVSQPFLMD	1.000	0.648400
229	HLA-DPA10103-DPB10401	LPIGINITRFQTLLA	4	INITRFQTL	1.000	0.613948
137	HLA-DPA10103-DPB10401	NDPFLGVYYHKNNKS	3	FLGVYYHKN	0.993	0.595645
310	HLA-DPA10103-DPB10401	KGIYQTSNFRVQPTE	3	YQTSNFRVQ	0.980	0.515109
919	HLA-DPA10103-DPB10401	NQKLIANQFNSAIGK	3	LIANQFNSA	1.000	0.473444
1059	HLA-DPA10103-DPB10401	GVVFLHVTYVPAQEK	3	FLHVTYVPA	1.000	0.470661
39	HLA-DPA10103-DPB10401	PDKVFRSSVLHSTQD	4	FRSSVLHST	0.993	0.353789
815	HLA-DPA10103-DPB10401	RSFIEDLLFNKVTLA	3	IEDLLFNKV	0.993	0.308022
794	HLA-DPA10103-DPB10401	IKDFGGFNFSQILPD	3	FGGFNFSQI	0.933	0.301512
465	HLA-DPA10103-DPB10401	ERDISTEIYQAGSTP	3	ISTEIYQAG	1.000	0.281516
797	HLA-DPA10103-DPB10401	FGGFNFSQILPDPSK	3	FNFSQILPD	0.940	0.200222
1044	HLA-DPA10103-DPB10401	GKGYHLMSFPQSAPH	3	YHLMSFPQS	0.980	0.194480
149	HLA-DPA10103-DPB10401	NKSWMESEFRVYSSA	3	WMESEFRVY	0.980	0.193790
364	HLA-DPA10103-DPB10401	DYSVLYNSASFSTFK	5	YNSASFSTF	0.967	0.157613
500	HLA-DPA10103-DPB10401	TNGVGYQPYRVVVLS	3	VGYQPYRVV	0.920	0.146939
507	HLA-DPA10103-DPB10401	PYRVVVLSFELLHAP	3	VVVLSFELL	0.920	0.142047
448	HLA-DPA10103-DPB10401	NYNYLYRLFRKSNLK	3	YLYRLFRKS	0.873	0.133866

Allele: HLA-DPA10103-DPB10601

Pos	MHC	Peptide	Of	Core	Core_Rel	Score_EL
679	HLA-DPA10103-DPB10601	NSPRRARSVASQSII	3	RRARSVASQ	0.407	0.820258
680	HLA-DPA10103-DPB10601	SPRRARSVASQSIIA	3	RARSVASQS	0.633	0.793414
154	HLA-DPA10103-DPB10601	ESEFRVYSSANNCTF	4	RVYSSANNC	0.693	0.692347
343	HLA-DPA10103-DPB10601	NATRFASVYAWNRKR	3	RFASVYAWN	0.807	0.623829
1181	HLA-DPA10103-DPB10601	KEIDRLNEVAKNLNE	4	RLNEVAKNL	0.687	0.609740
234	HLA-DPA10103-DPB10601	NITRFQTLLALHRSY	3	RFQTLLALH	0.733	0.562497
630	HLA-DPA10103-DPB10601	TPTWRVYSTGSNVFQ	4	RVYSTGSNV	0.687	0.556733
1016	HLA-DPA10103-DPB10601	AEIRASANLAATKMS	3	RASANLAAT	0.807	0.548163
17	HLA-DPA10103-DPB10601	NLTTRTQLPPAYTNS	4	RTQLPPAYT	0.707	0.541266
1180	HLA-DPA10103-DPB10601	QKEIDRLNEVAKNLN	4	DRLNEVAKN	0.320	0.499970
243	HLA-DPA10103-DPB10601	ALHRSYLTPGDSSSG	3	RSYLTPGDS	0.727	0.457338
762	HLA-DPA10103-DPB10601	QLNRALTGIAVEQDK	3	RALTGIAVE	0.780	0.449189
404	HLA-DPA10103-DPB10601	GDEVRQIAPGQTGKI	4	RQIAPGQTG	0.680	0.424062

Allele: HLA-DPA10201-DPB10101

Pos	MHC	Peptide	Of	Core	Core_Rel	Score_EL
338	HLA-DPA10201-DPB10101	FGEVFNATRFASVYA	4	FNATRFASV	1.000	0.621325
137	HLA-DPA10201-DPB10101	NDPFLGVYYHKNNKS	3	FLGVYYHKN	0.993	0.352920
310	HLA-DPA10201-DPB10101	KGIYQTSNFRVQPTE	3	YQTSNFRVQ	0.860	0.337032

167	HLA-DPA10201-DPB10101	TFEYVSQPFLMDLEG	3	YVSQPFLMD	0.973	0.297948
39	HLA-DPA10201-DPB10101	PDKVFRSSVLHSTQD	4	FRSSVLHST	0.900	0.282963
919	HLA-DPA10201-DPB10101	NQKLIANQFNSAIGK	3	LIANQFNSA	0.967	0.252460
1059	HLA-DPA10201-DPB10101	GVVFLHVTYVPAQEK	3	FLHVTYVPA	0.840	0.242960
229	HLA-DPA10201-DPB10101	LPIGINITRFQTLLA	4	INITRFQTL	0.973	0.238437
797	HLA-DPA10201-DPB10101	FGGFNFSQILPDPSK	3	FNFSQILPD	0.927	0.213300
794	HLA-DPA10201-DPB10101	IKDFGGFNFSQILPD	3	FGGFNFSQI	0.753	0.184192
465	HLA-DPA10201-DPB10101	ERDISTEIYQAGSTP	3	ISTEIYQAG	0.880	0.176552
813	HLA-DPA10201-DPB10101	SKRSFIEDLLFNKVT	5	IEDLLFNKV	0.767	0.159134
364	HLA-DPA10201-DPB10101	DYSVLYNSASFSTFK	5	YNSASFSTF	0.747	0.137097
315	HLA-DPA10201-DPB10101	TSNFRVQPTESIVRF	3	FRVQPTESI	0.860	0.134168

Allele: HLA-DPA10201-DPB10501

Pos	MHC	Peptide	Of	Core	Core_Rel	Score_EL
338	HLA-DPA10201-DPB10501	FGEVFNATRFASVYA	4	FNATRFASV	0.953	0.389147
137	HLA-DPA10201-DPB10501	NDPFLGVYYHKNNKS	3	FLGVYYHKN	0.933	0.306581
310	HLA-DPA10201-DPB10501	KGIYQTSNFRVQPTE	3	YQTSNFRVQ	0.820	0.228629
343	HLA-DPA10201-DPB10501	NATRFASVYAWNRKR	4	FASVYAWNR	0.513	0.194153
554	HLA-DPA10201-DPB10501	ESNKKFLPFQQFGRD	3	KKFLPFQQF	0.620	0.187663
39	HLA-DPA10201-DPB10501	PDKVFRSSVLHSTQD	4	FRSSVLHST	0.853	0.183076
919	HLA-DPA10201-DPB10501	NQKLIANQFNSAIGK	3	LIANQFNSA	0.947	0.165449
1059	HLA-DPA10201-DPB10501	GVVFLHVTYVPAQEK	3	FLHVTYVPA	0.800	0.156823
555	HLA-DPA10201-DPB10501	SNKKFLPFQQFGRDI	4	FLPFQQFGR	0.500	0.133289
234	HLA-DPA10201-DPB10501	NITRFQTLLALHRSY	4	FQTLLALHR	0.473	0.129145
446	HLA-DPA10201-DPB10501	GGNYNYLYRLFRKSN	5	YLYRLFRKS	0.453	0.127654
167	HLA-DPA10201-DPB10501	TFEYVSQPFLMDLEG	3	YVSQPFLMD	0.907	0.108624
465	HLA-DPA10201-DPB10501	ERDISTEIYQAGSTP	3	ISTEIYQAG	0.907	0.108208
912	HLA-DPA10201-DPB10501	TQNVLYENQKLIANQ	3	VLYENQKLI	0.647	0.105018
778	HLA-DPA10201-DPB10501	TQEVFAQVKQIYKTP	3	VFAQVKQIY	0.467	0.104289
1044	HLA-DPA10201-DPB10501	GKGYHLMSFPQSAPH	3	YHLMSFPQS	0.867	0.099378
779	HLA-DPA10201-DPB10501	QEVFAQVKQIYKTPP	3	FAQVKQIYK	0.587	0.096005
445	HLA-DPA10201-DPB10501	VGGNYNYLYRLFRKS	4	YNYLYRLFR	0.513	0.093976

Allele: HLA-DPA10201-DPB11401

Pos	MHC	Peptide	Of	Core	Core_Rel	Score_EL
155	HLA-DPA10201-DPB11401	SEFRVYSSANNCTFE	3	RVYSSANNC	0.693	0.287575
680	HLA-DPA10201-DPB11401	SPRRARSVASQSIIA	3	RARSVASQS	0.673	0.266303
630	HLA-DPA10201-DPB11401	TPTWRVYSTGSNVFQ	4	RVYSTGSNV	0.713	0.250729
1181	HLA-DPA10201-DPB11401	KEIDRLNEVAKNLNE	3	DRLNEVAKN	0.380	0.228405
343	HLA-DPA10201-DPB11401	NATRFASVYAWNRKR	3	RFASVYAWN	0.493	0.213586

1015	HLA-DPA10201-DPB11401	AAEIRASANLAATKM	3	IRASANLAA	0.407	0.207001
338	HLA-DPA10201-DPB11401	FGEVFNATRFASVYA	4	FNATRFASV	0.947	0.205779
1182	HLA-DPA10201-DPB11401	EIDRLNEVAKNLNES	3	RLNEVAKNL	0.620	0.195790
1016	HLA-DPA10201-DPB11401	AEIRASANLAATKMS	3	RASANLAAT	0.647	0.193496
404	HLA-DPA10201-DPB11401	GDEVRQIAPGQTGKI	3	VRQIAPGQT	0.407	0.187268
39	HLA-DPA10201-DPB11401	PDKVFRSSVLHSTQD	4	FRSSVLHST	0.720	0.179055
405	HLA-DPA10201-DPB11401	DEVRQIAPGQTGKIA	3	RQIAPGQTG	0.640	0.178759
677	HLA-DPA10201-DPB11401	QTNSPRRARSVASQS	4	PRRARSVAS	0.273	0.142119
1070	HLA-DPA10201-DPB11401	AQEKNFTTAPAICHD	3	KNFTTAPAI	0.633	0.132628

Allele: HLA-DPA10301-DPB10402

Pos	MHC	Peptide	Of	Core	Core_Rel	Score_EL
338	HLA-DPA10301-DPB10402	FGEVFNATRFASVYA	4	FNATRFASV	1.000	0.799477
137	HLA-DPA10301-DPB10402	NDPFLGVYYHKNNKS	3	FLGVYYHKN	0.967	0.534338
919	HLA-DPA10301-DPB10402	NQKLIANQFNSAIGK	3	LIANQFNSA	0.993	0.466164
310	HLA-DPA10301-DPB10402	KGIYQTSNFRVQPTE	3	YQTSNFRVQ	0.920	0.463023
39	HLA-DPA10301-DPB10402	PDKVFRSSVLHSTQD	4	FRSSVLHST	0.940	0.442875
167	HLA-DPA10301-DPB10402	TFEYVSQPFLMDLEG	3	YVSQPFLMD	0.980	0.435622
1059	HLA-DPA10301-DPB10402	GVVFLHVTYVPAQEK	3	FLHVTYVPA	0.933	0.413981
229	HLA-DPA10301-DPB10402	LPIGINITRFQTLLA	4	INITRFQTL	0.993	0.384669
465	HLA-DPA10301-DPB10402	ERDISTEIYQAGSTP	3	ISTEIYQAG	0.980	0.332258
797	HLA-DPA10301-DPB10402	FGGFNFSQILPDPSK	3	FNFSQILPD	0.953	0.278486
815	HLA-DPA10301-DPB10402	RSFIEDLLFNKVTLA	3	IEDLLFNKV	0.913	0.248253
1044	HLA-DPA10301-DPB10402	GKGYHLMSFPQSAPH	3	YHLMSFPQS	0.953	0.241450
1003	HLA-DPA10301-DPB10402	SLQTYVTQQLIRAAE	4	YVTQQLIRA	0.827	0.213494
794	HLA-DPA10301-DPB10402	IKDFGGFNFSQILPD	3	FGGFNFSQI	0.833	0.210810
315	HLA-DPA10301-DPB10402	TSNFRVQPTESIVRF	3	FRVQPTESI	0.953	0.204325
912	HLA-DPA10301-DPB10402	TQNVLYENQKLIANQ	3	VLYENQKLI	0.633	0.197556

Allele: HLA-DQA10101-DQB10501

Pos	MHC	Peptide	Of	Core	Core_Rel	Score_EL
207	HLA-DQA10101-DQB10501	HTPINLVRDLPQGFS	3	INLVRDLPQ	0.920	0.050726
80	HLA-DQA10101-DQB10501	DNPVLPFNDGVYFAS	3	VLPFNDGVY	0.940	0.041026
1141	HLA-DQA10101-DQB10501	LQPELDSFKEELDKY	4	LDSFKEELD	0.947	0.037753
430	HLA-DQA10101-DQB10501	TGCVIAWNSNNLDSK	3	VIAWNSNNL	0.773	0.036926
867	HLA-DQA10101-DQB10501	DEMIAQYTSALLAGT	3	IAQYTSALL	0.887	0.035834
59	HLA-DQA10101-DQB10501	FSNVTWFHAIHVSGT	3	VTWFHAIHV	0.813	0.033282
309	HLA-DQA10101-DQB10501	EKGIYQTSNFRVQPT	3	IYQTSNFRV	0.727	0.030139
764	HLA-DQA10101-DQB10501	NRALTGIAVEQDKNT	3	LTGIAVEQD	0.887	0.029183
1130	HLA-DQA10101-DQB10501	IGIVNNTVYDPLQPE	4	NNTVYDPLQ	0.600	0.024867
220	HLA-DQA10101-DQB10501	FSALEPLVDLPIGIN	3	LEPLVDLPI	0.980	0.023354

618	HLA-DQA10101-DQB10501	TEVPVAIHADQLTPT	4	VAIHADQLT	0.560	0.022634

Allele: HLA-DQA10102-DQB10502

Pos	MHC	Peptide	Of	Core	Core_Rel	Score_EL
207	HLA-DQA10102-DQB10502	HTPINLVRDLPQGFS	3	INLVRDLPQ	0.973	0.130955
764	HLA-DQA10102-DQB10502	NRALTGIAVEQDKNT	3	LTGIAVEQD	0.967	0.102791
1141	HLA-DQA10102-DQB10502	LQPELDSFKEELDKY	4	LDSFKEELD	0.980	0.073965
1150	HLA-DQA10102-DQB10502	EELDKYFKNHTSPDV	3	DKYFKNHTS	0.840	0.059777
1130	HLA-DQA10102-DQB10502	IGIVNNTVYDPLQPE	4	NNTVYDPLQ	0.733	0.055147
80	HLA-DQA10102-DQB10502	DNPVLPFNDGVYFAS	3	VLPFNDGVY	0.893	0.048192
618	HLA-DQA10102-DQB10502	TEVPVAIHADQLTPT	4	VAIHADQLT	0.707	0.045908
797	HLA-DQA10102-DQB10502	FGGFNFSQILPDPSK	3	FNFSQILPD	0.773	0.043116

Allele: HLA-DQA10102-DQB10602

Pos	MHC	Peptide	Of	Core	Core_Rel	Score_EL
1009	HLA-DQA10102-DQB10602	TQQLIRAAEIRASAN	4	IRAAEIRAS	1.000	0.942370
1168	HLA-DQA10102-DQB10602	DISGINASVVNIQKE	4	INASVVNIQ	1.000	0.732077
634	HLA-DQA10102-DQB10602	RVYSTGSNVFQTRAG	3	STGSNVFQT	1.000	0.672240
602	HLA-DQA10102-DQB10602	TNTSNQVAVLYQDVN	4	NQVAVLYQD	0.567	0.543760
764	HLA-DQA10102-DQB10602	NRALTGIAVEQDKNT	3	LTGIAVEQD	0.993	0.526578
950	HLA-DQA10102-DQB10602	DVVNQNAQALNTLVK	3	NQNAQALNT	0.980	0.514267
39	HLA-DQA10102-DQB10602	PDKVFRSSVLHSTQD	4	FRSSVLHST	0.633	0.503702

Allele: HLA-DQA10103-DQB10603

Pos	MHC	Peptide	Of	Core	Core_Rel	Score_EL
1009	HLA-DQA10103-DQB10603	TQQLIRAAEIRASAN	4	IRAAEIRAS	0.927	0.561329
207	HLA-DQA10103-DQB10603	HTPINLVRDLPQGFS	3	INLVRDLPQ	0.987	0.542747
1130	HLA-DQA10103-DQB10603	IGIVNNTVYDPLQPE	4	NNTVYDPLQ	0.900	0.494898
1168	HLA-DQA10103-DQB10603	DISGINASVVNIQKE	4	INASVVNIQ	0.913	0.410685
566	HLA-DQA10103-DQB10603	GRDIADTTDAVRDPQ	3	IADTTDAVR	0.767	0.375269
764	HLA-DQA10103-DQB10603	NRALTGIAVEQDKNT	3	LTGIAVEQD	0.947	0.354426
1014	HLA-DQA10103-DQB10603	RAAEIRASANLAATK	4	IRASANLAA	0.820	0.315340
278	HLA-DQA10103-DQB10603	KYNENGTITDAVDCA	4	NGTITDAVD	0.627	0.267493
569	HLA-DQA10103-DQB10603	IADTTDAVRDPQTLE	4	TDAVRDPQT	0.893	0.255403
86	HLA-DQA10103-DQB10603	FNDGVYFASTEKSNI	4	VYFASTEKS	0.933	0.250487
1060	HLA-DQA10103-DQB10603	VVFLHVTYVPAQEKN	3	LHVTYVPAQ	0.833	0.250228
602	HLA-DQA10103-DQB10603	TNTSNQVAVLYQDVN	3	SNQVAVLYQ	0.620	0.241920

Allele: HLA-DQA10104-DQB10503

Pos	MHC	Peptide	Of	Core	Core_Rel	Score_EL
207	HLA-DQA10104-DQB10503	HTPINLVRDLPQGFS	3	INLVRDLPQ	0.973	0.082710
232	HLA-DQA10104-DQB10503	GINITRFQTLLALHR	3	ITRFQTLLA	0.900	0.065833
1150	HLA-DQA10104-DQB10503	EELDKYFKNHTSPDV	3	DKYFKNHTS	0.893	0.061784
1009	HLA-DQA10104-DQB10503	TQQLIRAAEIRASAN	4	IRAAEIRAS	0.773	0.054256
1014	HLA-DQA10104-DQB10503	RAAEIRASANLAATK	4	IRASANLAA	0.713	0.048466
1097	HLA-DQA10104-DQB10503	SNGTHWFVTQRNFYE	3	THWFVTQRN	0.787	0.045070
430	HLA-DQA10104-DQB10503	TGCVIAWNSNNLDSK	3	VIAWNSNNL	0.747	0.041535
1130	HLA-DQA10104-DQB10503	IGIVNNTVYDPLQPE	4	NNTVYDPLQ	0.640	0.039739
252	HLA-DQA10104-DQB10503	GDSSSGWTAGAAAYY	3	SSGWTAGAA	0.820	0.039244
764	HLA-DQA10104-DQB10503	NRALTGIAVEQDKNT	3	LTGIAVEQD	0.867	0.039010
300	HLA-DQA10104-DQB10503	KCTLKSFTVEKGIYQ	3	LKSFTVEKG	0.960	0.037340
831	HLA-DQA10104-DQB10503	AGFIKQYGDCLGDIA	3	IKQYGDCLG	0.893	0.036251
59	HLA-DQA10104-DQB10503	FSNVTWFHAIHVSGT	3	VTWFHAIHV	0.673	0.035830
867	HLA-DQA10104-DQB10503	DEMIAQYTSALLAGT	3	IAQYTSALL	0.700	0.034883
618	HLA-DQA10104-DQB10503	TEVPVAIHADQLTPT	4	VAIHADQLT	0.673	0.034649
1141	HLA-DQA10104-DQB10503	LQPELDSFKEELDKY	4	LDSFKEELD	0.947	0.033889

Allele: HLA-DQA10201-DQB10202

Pos	MHC	Peptide	Of	Core	Core_Rel	Score_EL
617	HLA-DQA10201-DQB10202	CTEVPVAIHADQLTP	4	PVAIHADQL	0.993	0.692045
566	HLA-DQA10201-DQB10202	GRDIADTTDAVRDPQ	4	ADTTDAVRD	0.847	0.657678
716	HLA-DQA10201-DQB10202	TNFTISVTTEILPVS	3	TISVTTEIL	0.773	0.545931
255	HLA-DQA10201-DQB10202	SSGWTAGAAAYYVGY	3	WTAGAAAYY	0.520	0.512220
1111	HLA-DQA10201-DQB10202	EPQIITTDNTFVSGN	4	ITTDNTFVS	0.840	0.488437
575	HLA-DQA10201-DQB10202	AVRDPQTLEILDITP	4	PQTLEILDI	0.993	0.456695
87	HLA-DQA10201-DQB10202	NDGVYFASTEKSNII	3	VYFASTEKS	0.767	0.454872
797	HLA-DQA10201-DQB10202	FGGFNFSQILPDPSK	3	FNFSQILPD	0.940	0.433096
88	HLA-DQA10201-DQB10202	DGVYFASTEKSNIIR	5	ASTEKSNII	0.520	0.420900
763	HLA-DQA10201-DQB10202	LNRALTGIAVEQDKN	4	LTGIAVEQD	0.853	0.419571

Allele: HLA-DQA10201-DQB10301

Pos	MHC	Peptide	Of	Core	Core_Rel	Score_EL
255	HLA-DQA10201-DQB10301	SSGWTAGAAAYYVGY	3	WTAGAAAYY	0.540	0.819672
256	HLA-DQA10201-DQB10301	SGWTAGAAAYYVGYL	3	TAGAAAYYV	0.547	0.741956
87	HLA-DQA10201-DQB10301	NDGVYFASTEKSNII	3	VYFASTEKS	0.900	0.722439
1168	HLA-DQA10201-DQB10301	DISGINASVVNIQKE	4	INASVVNIQ	1.000	0.714041

1009	HLA-DQA10201-DQB10301	TQQLIRAAEIRASAN	4	IRAAEIRAS	0.980	0.686205
39	HLA-DQA10201-DQB10301	PDKVFRSSVLHSTQD	4	FRSSVLHST	0.760	0.668791
883	HLA-DQA10201-DQB10301	TSGWTFGAGAALQIP	5	FGAGAALQI	0.500	0.659181
663	HLA-DQA10201-DQB10301	DIPIGAGICASYQTQ	3	IGAGICASY	0.707	0.598714
407	HLA-DQA10201-DQB10301	VRQIAPGQTGKIADY	3	IAPGQTGKI	1.000	0.595225
797	HLA-DQA10201-DQB10301	FGGFNFSQILPDPSK	3	FNFSQILPD	0.973	0.546661

Allele: HLA-DQA10201-DQB10303

Pos	MHC	Peptide	Of	Core	Core_Rel	Score_EL
86	HLA-DQA10201-DQB10303	FNDGVYFASTEKSNI	4	VYFASTEKS	0.973	0.291270
1009	HLA-DQA10201-DQB10303	TQQLIRAAEIRASAN	4	IRAAEIRAS	0.913	0.267392
1168	HLA-DQA10201-DQB10303	DISGINASVVNIQKE	4	INASVVNIQ	0.900	0.267024
255	HLA-DQA10201-DQB10303	SSGWTAGAAAYYVGY	3	WTAGAAAYY	0.580	0.253003
39	HLA-DQA10201-DQB10303	PDKVFRSSVLHSTQD	3	VFRSSVLHS	0.500	0.227355
618	HLA-DQA10201-DQB10303	TEVPVAIHADQLTPT	3	PVAIHADQL	0.767	0.212020
426	HLA-DQA10201-DQB10303	PDDFTGCVIAWNSNN	3	FTGCVIAWN	0.887	0.208378
566	HLA-DQA10201-DQB10303	GRDIADTTDAVRDPQ	4	ADTTDAVRD	0.593	0.195030
763	HLA-DQA10201-DQB10303	LNRALTGIAVEQDKN	4	LTGIAVEQD	0.747	0.192315
797	HLA-DQA10201-DQB10303	FGGFNFSQILPDPSK	3	FNFSQILPD	0.820	0.178888
256	HLA-DQA10201-DQB10303	SGWTAGAAAYYVGYL	3	TAGAAAYYV	0.527	0.172867
1014	HLA-DQA10201-DQB10303	RAAEIRASANLAATK	4	IRASANLAA	0.473	0.158596
40	HLA-DQA10201-DQB10303	DKVFRSSVLHSTQDL	3	FRSSVLHST	0.613	0.153906
1166	HLA-DQA10201-DQB10303	LGDISGINASVVNIQ	3	ISGINASVV	0.660	0.152610

Allele: HLA-DQA10201-DQB10402

Pos	MHC	Peptide	Of	Core	Core_Rel	Score_EL
1009	HLA-DQA10201-DQB10402	TQQLIRAAEIRASAN	4	IRAAEIRAS	0.833	0.472338
39	HLA-DQA10201-DQB10402	PDKVFRSSVLHSTQD	4	FRSSVLHST	0.533	0.402738
60	HLA-DQA10201-DQB10402	SNVTWFHAIHVSGTN	5	FHAIHVSGT	0.453	0.381454
87	HLA-DQA10201-DQB10402	NDGVYFASTEKSNII	3	VYFASTEKS	0.773	0.381117
1014	HLA-DQA10201-DQB10402	RAAEIRASANLAATK	4	IRASANLAA	0.507	0.338449
797	HLA-DQA10201-DQB10402	FGGFNFSQILPDPSK	3	FNFSQILPD	0.753	0.337318
88	HLA-DQA10201-DQB10402	DGVYFASTEKSNIIR	3	YFASTEKSN	0.333	0.319097
764	HLA-DQA10201-DQB10402	NRALTGIAVEQDKNT	3	LTGIAVEQD	0.800	0.318981
343	HLA-DQA10201-DQB10402	NATRFASVYAWNRKR	4	FASVYAWNR	0.720	0.314884
59	HLA-DQA10201-DQB10402	FSNVTWFHAIHVSGT	3	VTWFHAIHV	0.440	0.312393
618	HLA-DQA10201-DQB10402	TEVPVAIHADQLTPT	3	PVAIHADQL	0.647	0.312056
255	HLA-DQA10201-DQB10402	SSGWTAGAAAYYVGY	3	WTAGAAAYY	0.553	0.310478
403	HLA-DQA10201-DQB10402	RGDEVRQIAPGQTGK	4	VRQIAPGQT	0.860	0.303128
309	HLA-DQA10201-DQB10402	EKGIYQTSNFRVQPT	3	IYQTSNFRV	0.487	0.297633
310	HLA-DQA10201-DQB10402	KGIYQTSNFRVQPTE	3	YQTSNFRVQ	0.453	0.291809

1168	HLA-DQA10201-DQB10402	DISGINASVVNIQKE	4	INASVVNIQ	0.800	0.289663

Allele: HLA-DQA10301-DQB10302

Pos	MHC	Peptide	Of	Core	Core_Rel	Score_EL
764	HLA-DQA10301-DQB10302	NRALTGIAVEQDKNT	3	LTGIAVEQD	0.967	0.063847
797	HLA-DQA10301-DQB10302	FGGFNFSQILPDPSK	3	FNFSQILPD	0.813	0.045522
566	HLA-DQA10301-DQB10302	GRDIADTTDAVRDPQ	4	ADTTDAVRD	0.607	0.037359
1111	HLA-DQA10301-DQB10302	EPQIITTDNTFVSGN	4	ITTDNTFVS	0.747	0.032300
86	HLA-DQA10301-DQB10302	FNDGVYFASTEKSNI	4	VYFASTEKS	0.860	0.027269
1168	HLA-DQA10301-DQB10302	DISGINASVVNIQKE	4	INASVVNIQ	0.673	0.020080
690	HLA-DQA10301-DQB10302	QSIIAYTMSLGAENS	3	IAYTMSLGA	0.287	0.019576
255	HLA-DQA10301-DQB10302	SSGWTAGAAAYYVGY	4	TAGAAAYYV	0.460	0.019110
426	HLA-DQA10301-DQB10302	PDDFTGCVIAWNSNN	3	FTGCVIAWN	0.780	0.019060
207	HLA-DQA10301-DQB10302	HTPINLVRDLPQGFS	3	INLVRDLPQ	0.900	0.018051
1009	HLA-DQA10301-DQB10302	TQQLIRAAEIRASAN	4	IRAAEIRAS	0.793	0.017722
618	HLA-DQA10301-DQB10302	TEVPVAIHADQLTPT	3	PVAIHADQL	0.667	0.017692
254	HLA-DQA10301-DQB10302	SSSGWTAGAAAYYVG	4	WTAGAAAYY	0.387	0.017133
1170	HLA-DQA10301-DQB10302	SGINASVVNIQKEID	4	ASVVNIQKE	0.400	0.016798

Allele: HLA-DQA10303-DQB10402

Pos	MHC	Peptide	Of	Core	Core_Rel	Score_EL
39	HLA-DQA10303-DQB10402	PDKVFRSSVLHSTQD	4	FRSSVLHST	0.600	0.231686
1009	HLA-DQA10303-DQB10402	TQQLIRAAEIRASAN	4	IRAAEIRAS	0.900	0.230195
797	HLA-DQA10303-DQB10402	FGGFNFSQILPDPSK	3	FNFSQILPD	0.787	0.209469
200	HLA-DQA10303-DQB10402	YFKIYSKHTPINLVR	3	IYSKHTPIN	0.647	0.203023
818	HLA-DQA10303-DQB10402	IEDLLFNKVTLADAG	4	LFNKVTLAD	0.513	0.202665
1151	HLA-DQA10303-DQB10402	ELDKYFKNHTSPDVD	4	YFKNHTSPD	0.353	0.193426
197	HLA-DQA10303-DQB10402	IDGYFKIYSKHTPIN	3	YFKIYSKHT	0.507	0.182090
1150	HLA-DQA10303-DQB10402	EELDKYFKNHTSPDV	3	DKYFKNHTS	0.607	0.177460
630	HLA-DQA10303-DQB10402	TPTWRVYSTGSNVFQ	3	WRVYSTGSN	0.740	0.160456
976	HLA-DQA10303-DQB10402	VLNDILSRLDKVEAE	4	ILSRLDKVE	0.533	0.159555

Allele: HLA-DQA10401-DQB10402

Pos	MHC	Peptide	Of	Core	Core_Rel	Score_EL
764	HLA-DQA10401-DQB10402	NRALTGIAVEQDKNT	3	LTGIAVEQD	0.927	0.292678
797	HLA-DQA10401-DQB10402	FGGFNFSQILPDPSK	3	FNFSQILPD	0.893	0.261907
255	HLA-DQA10401-DQB10402	SSGWTAGAAAYYVGY	4	TAGAAAYYV	0.487	0.214183
1009	HLA-DQA10401-DQB10402	TQQLIRAAEIRASAN	4	IRAAEIRAS	0.893	0.198262

254	HLA-DQA10401-DQB10402	SSSGWTAGAAAYYVG	4	WTAGAAAYY	0.433	0.191532
566	HLA-DQA10401-DQB10402	GRDIADTTDAVRDPQ	4	ADTTDAVRD	0.660	0.187894
86	HLA-DQA10401-DQB10402	FNDGVYFASTEKSNI	4	VYFASTEKS	0.807	0.185829
1168	HLA-DQA10401-DQB10402	DISGINASVVNIQKE	4	INASVVNIQ	0.740	0.174254
618	HLA-DQA10401-DQB10402	TEVPVAIHADQLTPT	3	PVAIHADQL	0.727	0.159260
39	HLA-DQA10401-DQB10402	PDKVFRSSVLHSTQD	4	FRSSVLHST	0.587	0.157120
1141	HLA-DQA10401-DQB10402	LQPELDSFKEELDKY	4	LDSFKEELD	0.933	0.150451
216	HLA-DQA10401-DQB10402	LPQGFSALEPLVDLP	4	FSALEPLVD	0.807	0.145024
1170	HLA-DQA10401-DQB10402	SGINASVVNIQKEID	4	ASVVNIQKE	0.467	0.144171

Allele: HLA-DQA10501-DQB10201

Pos	MHC	Peptide	Of	Core	Core_Rel	Score_EL
617	HLA-DQA10501-DQB10201	CTEVPVAIHADQLTP	4	PVAIHADQL	0.993	0.680372
566	HLA-DQA10501-DQB10201	GRDIADTTDAVRDPQ	4	ADTTDAVRD	0.687	0.513351
1111	HLA-DQA10501-DQB10201	EPQIITTDNTFVSGN	4	ITTDNTFVS	0.860	0.497346
255	HLA-DQA10501-DQB10201	SSGWTAGAAAYYVGY	3	WTAGAAAYY	0.560	0.491967
87	HLA-DQA10501-DQB10201	NDGVYFASTEKSNII	3	VYFASTEKS	0.927	0.458878
764	HLA-DQA10501-DQB10201	NRALTGIAVEQDKNT	3	LTGIAVEQD	0.960	0.441353
797	HLA-DQA10501-DQB10201	FGGFNFSQILPDPSK	3	FNFSQILPD	0.920	0.440875
716	HLA-DQA10501-DQB10201	TNFTISVTTEILPVS	3	TISVTTEIL	0.780	0.389275

Allele: HLA-DQA10501-DQB10301

Pos	MHC	Peptide	Of	Core	Core_Rel	Score_EL
255	HLA-DQA10501-DQB10301	SSGWTAGAAAYYVGY	3	WTAGAAAYY	0.620	0.821014
256	HLA-DQA10501-DQB10301	SGWTAGAAAYYVGYL	3	TAGAAAYYV	0.533	0.724726
87	HLA-DQA10501-DQB10301	NDGVYFASTEKSNII	3	VYFASTEKS	0.940	0.710684
1009	HLA-DQA10501-DQB10301	TQQLIRAAEIRASAN	4	IRAAEIRAS	0.987	0.705335
1168	HLA-DQA10501-DQB10301	DISGINASVVNIQKE	4	INASVVNIQ	1.000	0.674956
883	HLA-DQA10501-DQB10301	TSGWTFGAGAALQIP	5	FGAGAALQI	0.607	0.660836
39	HLA-DQA10501-DQB10301	PDKVFRSSVLHSTQD	4	FRSSVLHST	0.640	0.612066
407	HLA-DQA10501-DQB10301	VRQIAPGQTGKIADY	3	IAPGQTGKI	0.993	0.606718
663	HLA-DQA10501-DQB10301	DIPIGAGICASYQTQ	3	IGAGICASY	0.767	0.595821
797	HLA-DQA10501-DQB10301	FGGFNFSQILPDPSK	3	FNFSQILPD	0.973	0.477932

Allele: HLA-DQA10501-DQB10302

Pos	MHC	Peptide	Of	Core	Core_Rel	Score_EL
764	HLA-DQA10501-DQB10302	NRALTGIAVEQDKNT	3	LTGIAVEQD	0.920	0.151863

255	HLA-DQA10501-DQB10302	SSGWTAGAAAYYVGY	3	WTAGAAAYY	0.593	0.117918
1111	HLA-DQA10501-DQB10302	EPQIITTDNTFVSGN	4	ITTDNTFVS	0.707	0.116772
618	HLA-DQA10501-DQB10302	TEVPVAIHADQLTPT	3	PVAIHADQL	0.780	0.116630
797	HLA-DQA10501-DQB10302	FGGFNFSQILPDPSK	3	FNFSQILPD	0.833	0.112836
86	HLA-DQA10501-DQB10302	FNDGVYFASTEKSNI	4	VYFASTEKS	0.887	0.097817
566	HLA-DQA10501-DQB10302	GRDIADTTDAVRDPQ	4	ADTTDAVRD	0.527	0.089850
207	HLA-DQA10501-DQB10302	HTPINLVRDLPQGFS	3	INLVRDLPQ	0.847	0.078411
1168	HLA-DQA10501-DQB10302	DISGINASVVNIQKE	4	INASVVNIQ	0.807	0.073628
1009	HLA-DQA10501-DQB10302	TQQLIRAAEIRASAN	4	IRAAEIRAS	0.760	0.065519
426	HLA-DQA10501-DQB10302	PDDFTGCVIAWNSNN	3	FTGCVIAWN	0.860	0.062949
1014	HLA-DQA10501-DQB10302	RAAEIRASANLAATK	4	IRASANLAA	0.567	0.058167

Allele: HLA-DQA10501-DQB10303

Pos	MHC	Peptide	Of	Core	Core_Rel	Score_EL
86	HLA-DQA10501-DQB10303	FNDGVYFASTEKSNI	4	VYFASTEKS	0.967	0.281831
1009	HLA-DQA10501-DQB10303	TQQLIRAAEIRASAN	4	IRAAEIRAS	0.887	0.265950
1168	HLA-DQA10501-DQB10303	DISGINASVVNIQKE	4	INASVVNIQ	0.893	0.230350
255	HLA-DQA10501-DQB10303	SSGWTAGAAAYYVGY	3	WTAGAAAYY	0.627	0.227774
39	HLA-DQA10501-DQB10303	PDKVFRSSVLHSTQD	3	VFRSSVLHS	0.527	0.200066
426	HLA-DQA10501-DQB10303	PDDFTGCVIAWNSNN	3	FTGCVIAWN	0.913	0.189912
764	HLA-DQA10501-DQB10303	NRALTGIAVEQDKNT	3	LTGIAVEQD	0.867	0.161438
797	HLA-DQA10501-DQB10303	FGGFNFSQILPDPSK	3	FNFSQILPD	0.840	0.155641
1014	HLA-DQA10501-DQB10303	RAAEIRASANLAATK	4	IRASANLAA	0.533	0.151400
618	HLA-DQA10501-DQB10303	TEVPVAIHADQLTPT	3	PVAIHADQL	0.713	0.144207
40	HLA-DQA10501-DQB10303	DKVFRSSVLHSTQDL	3	FRSSVLHST	0.587	0.131615
883	HLA-DQA10501-DQB10303	TSGWTFGAGAALQIP	3	WTFGAGAAL	0.340	0.125802
1111	HLA-DQA10501-DQB10303	EPQIITTDNTFVSGN	4	ITTDNTFVS	0.800	0.124045
469	HLA-DQA10501-DQB10303	STEIYQAGSTPCNGV	3	IYQAGSTPC	0.713	0.119697
393	HLA-DQA10501-DQB10303	TNVYADSFVIRGDEV	3	YADSFVIRG	0.907	0.118041

Allele: HLA-DQA10501-DQB10402

Pos	MHC	Peptide	Of	Core	Core_Rel	Score_EL
1009	HLA-DQA10501-DQB10402	TQQLIRAAEIRASAN	4	IRAAEIRAS	0.827	0.497060
39	HLA-DQA10501-DQB10402	PDKVFRSSVLHSTQD	4	FRSSVLHST	0.493	0.413751
86	HLA-DQA10501-DQB10402	FNDGVYFASTEKSNI	4	VYFASTEKS	0.780	0.400926
61	HLA-DQA10501-DQB10402	NVTWFHAIHVSGTNG	4	FHAIHVSGT	0.673	0.385892
797	HLA-DQA10501-DQB10402	FGGFNFSQILPDPSK	3	FNFSQILPD	0.787	0.366966
403	HLA-DQA10501-DQB10402	RGDEVRQIAPGQTGK	4	VRQIAPGQT	0.900	0.342012
764	HLA-DQA10501-DQB10402	NRALTGIAVEQDKNT	3	LTGIAVEQD	0.853	0.338381
1014	HLA-DQA10501-DQB10402	RAAEIRASANLAATK	4	IRASANLAA	0.607	0.337765
343	HLA-DQA10501-DQB10402	NATRFASVYAWNRKR	4	FASVYAWNR	0.740	0.335467

59	HLA-DQA10501-DQB10402	FSNVTWFHAIHVSGT	3	VTWFHAIHV	0.473	0.322681
38	HLA-DQA10501-DQB10402	YPDKVFRSSVLHSTQ	4	VFRSSVLHS	0.433	0.319606
255	HLA-DQA10501-DQB10402	SSGWTAGAAAYYVGY	3	WTAGAAAYY	0.587	0.316422
618	HLA-DQA10501-DQB10402	TEVPVAIHADQLTPT	3	PVAIHADQL	0.653	0.314441
430	HLA-DQA10501-DQB10402	TGCVIAWNSNNLDSK	3	VIAWNSNNL	0.853	0.295448

Allele: HLA-DQA10601-DQB10402

Pos	MHC	Peptide	Of	Core	Core_Rel	Score_EL
1009	HLA-DQA10601-DQB10402	TQQLIRAAEIRASAN	4	IRAAEIRAS	0.860	0.302170
764	HLA-DQA10601-DQB10402	NRALTGIAVEQDKNT	3	LTGIAVEQD	0.887	0.258999
86	HLA-DQA10601-DQB10402	FNDGVYFASTEKSNI	4	VYFASTEKS	0.800	0.253918
255	HLA-DQA10601-DQB10402	SSGWTAGAAAYYVGY	3	WTAGAAAYY	0.493	0.240825
797	HLA-DQA10601-DQB10402	FGGFNFSQILPDPSK	3	FNFSQILPD	0.820	0.239470
39	HLA-DQA10601-DQB10402	PDKVFRSSVLHSTQD	4	FRSSVLHST	0.587	0.229841
618	HLA-DQA10601-DQB10402	TEVPVAIHADQLTPT	3	PVAIHADQL	0.660	0.216226
60	HLA-DQA10601-DQB10402	SNVTWFHAIHVSGTN	5	FHAIHVSGT	0.433	0.208136
252	HLA-DQA10601-DQB10402	GDSSSGWTAGAAAYY	3	SSGWTAGAA	0.560	0.203025
1168	HLA-DQA10601-DQB10402	DISGINASVVNIQKE	4	INASVVNIQ	0.800	0.190324
343	HLA-DQA10601-DQB10402	NATRFASVYAWNRKR	4	FASVYAWNR	0.687	0.189315
566	HLA-DQA10601-DQB10402	GRDIADTTDAVRDPQ	4	ADTTDAVRD	0.587	0.182640
1014	HLA-DQA10601-DQB10402	RAAEIRASANLAATK	4	IRASANLAA	0.493	0.180690
256	HLA-DQA10601-DQB10402	SGWTAGAAAYYVGYL	3	TAGAAAYYV	0.560	0.179288
1141	HLA-DQA10601-DQB10402	LQPELDSFKEELDKY	4	LDSFKEELD	0.920	0.176775
59	HLA-DQA10601-DQB10402	FSNVTWFHAIHVSGT	3	VTWFHAIHV	0.447	0.171340

Note: The epitopes conserved among Khosta-2 and SARS-CoV-2 are indicated in red.
